# Analysis of Fragmentation Pathways of Peptide Modified with Quaternary Ammonium and Phosphonium Group as Ionization Enhancers

**DOI:** 10.3390/molecules26226964

**Published:** 2021-11-18

**Authors:** Monika Kijewska, Dorota Gąszczyk, Remigiusz Bąchor, Piotr Stefanowicz, Zbigniew Szewczuk

**Affiliations:** Faculty of Chemistry, University of Wrocław, F. Joliot-Curie 14, 50-383 Wrocław, Poland; dorota.gaszczyk@chem.uni.wroc.pl (D.G.); piotr.stefanowicz@chem.uni.wroc.pl (P.S.); zbigniew.szewczuk@chem.uni.wroc.pl (Z.S.)

**Keywords:** derivatization, fixed charge tag, quaternary ammonium salt, mass spectrometry, electron-capture dissociation, collision induced dissociation

## Abstract

Peptide modification by a quaternary ammonium group containing a permanent positive charge is a promising method of increasing the ionization efficiency of the analyzed compounds, making ultra-sensitive detection even at the attomolar level possible. Charge-derivatized peptides may undergo both charge remote (ChR) and charge-directed (ChD) fragmentation. A series of model peptide conjugates derivatized with *N*,*N*,*N*-triethyloammonium (TEA), 1-azoniabicyclo[2.2.2]octane (ABCO), 2,4,6-triphenylopyridinium (TPP) and tris(2,4,6-trimetoxyphenylo)phosphonium (TMPP) groups were analyzed by their fragmentation pathways both in collision-induced dissociation (CID) and electron-capture dissociation (ECD) mode. The effect of the fixed-charge tag type and peptide sequence on the fragmentation pathways was investigated. We found that the aspartic acid effect plays a crucial role in the CID fragmentation of TPP and TEA peptide conjugates whereas it was not resolved for the peptides derivatized with the phosphonium group. ECD spectra are mostly dominated by c_n_ ions. ECD fragmentation of TMPP-modified peptides results in the formation of intense fragments derived from this fixed-charge tag, which may serve as reporter ion.

## 1. Introduction

Nowadays, tandem mass spectrometry is the common tool used in proteomic research. One of the most important applications of the MS/MS technique is the sequencing of peptides and proteins based on their fragmentation spectra. Peptides are molecules composed of amino acid residues connected by the amide bonds with similar energy, which can break down in many different ways, creating complex fragmentation spectra. Depending on the fragmentation method used, characteristic fragmentation pathways of the analyzed compound can be observed. Moreover, the value of the applied collision energy will be optimized by the length of the peptide chain, the presence of amino acid residues with functional groups in the side chain (e.g., carboxylic (D and E), amino (K), guanidino (R), amides (Q and N), and another basic, histidine (H) side chain), as well as the introduced modifications. The fragmentation of peptides by the CID (collision-induced dissociation) method mainly leads to the formation of b and y ion types, while for the ECD (electron-capture dissociation) method, mostly series of c and z ions are observed [1].

During MS/MS experiments, specific fragments of peptides may form, depending on the peptide sequence. Peptides containing aspartic or glutamic acid may form b ions, which may undergo cyclization on C-terminus. As a result of proton transfer from the carboxyl group to the nitrogen atom of the amide bond, a five-membered ring of succinimide anhydride is formed [2]. This effect results from the limitation of the mobility of mobile protons in the peptide chain [3] due to the presence of a strongly basic guanidine group from the side chain of arginine residues. Moreover, the introduction of a group containing a stable positive charge to a peptide molecule may cause the complete elimination of proton mobility [4]. Under CID conditions, fragmentation of protonated peptides containing a proline residue in the sequence leads to the presence of intense signals corresponding to the formation of the γ-type fragment ions, which are produced by dissociation of the amide bond at the proline residue [5]. The proline effect is explained by the high affinity of protons to the tertiary amide of the proline residue [6]. Moreover, computational studies have shown that during the fragmentation of protonated proline-containing peptides, unstable b fragments may also be formed. In this case, proline is at the C-terminus of the peptide chain and formed b ions have a bicyclic structure [7].

During the ESI-MS analysis, the functional groups in the peptide chain can be protonated, resulting in the formation of isomers characterized by different internal energy [8]. In order to acknowledge the processes taking place in the MS/MS experiment, a “mobile proton” model (ChD-charge directed fragmentation) has been developed [9]. This model assumes that under the collision energy, a proton may migrate to the other possible protonation sites, such as the guanidine group, the oxygen atom of the peptide bond, or to the α- and ε-amino groups. The protonation of the amide nitrogen can significantly weaken the peptide bond and therefore cause fragmentation. If proton mobility is not limited, peptides are fragmented according to the “mobile proton” mechanism, creating mainly b- and y-type fragmentation ions. The cleavage of the amide bond according to the ChD mechanism in peptides containing an arginine residue in sequence can be limited. It results from the strong proton binding by the basic guanidine group of the arginine residue. The resulting limitation of its mobility may hinder proton movement during MS/MS experiments. Due to the lack of a mobile proton and ion fragments formed during ChR (charge-remote fragmentation) [10], the mechanism is slightly different than those obtained from ChD fragmentation. The attachment of the positive charge to the *N*-terminal amino group of the peptide results in the formation of mainly a- and b-type fragments. The effect of arginine can be mimicked by the derivatization of a peptide with a fixed positive-charge-carrying molecule, including the quaternary ammonium group [11]. A stable positive charge can be introduced to a peptide molecule using the derivatization reaction to form quaternary ammonium [12,13,14,15,16,17], phosphonium [18,19,20], or sulfonium [21] groups. Moreover, the introduction of the ionization tag not only facilitates the fragmentation spectrum interpretation by generating a specific fragmentation path but significantly improves the ionization efficiency of derivatized compounds causing the analysis to find trace amounts of those substances. Recently, we have published a new and promising ionization enhancer based on pyridinium derivatives, which allowed us to detect the attomole amount of peptides by a mass spectrometer equipped with a standard ESI source and a triple quadrupole analyzer. The pyrylium moieties exhibit high selectivity towards the ε-amino group of the lysine moiety, which makes this approach particularly useful for tryptic peptides containing a lysine residue at the C-terminus [22,23]. Because the derivatization, depending on the concentration of the reagent used, may also take place at the N-terminus of some sterically non-hindered amino groups, it was important to analyze the effect of the ionization enhancer located at the N-terminal amino group [22]. Additionally, during MS/MS experiments, the derivatized peptide containing a triphenylpyridinium moiety reveals the characteristic reporter ion (2,4,6-triphenylpyridinium cation). This was used in MRM (Multiple Reaction Monitoring) analysis of biological samples as a predominant ion to investigate the presence of peptidic biomarkers of preeclampsia and kidney disease [24,25,26,27,28].

Herein, a fragmentation study on a series of model peptide conjugates derivatized with the quaternary ammonium group (*N*,*N*,*N*-triethylammoniumacetyl, 1-azoniabicyclo [2.2.2] octylammoniumacetyl, 2,4,6-triphenylopyridinium) and phosphonium-(tris(2,4,6-trimetoxyphenylo)phosphonium) was performed by tandem mass spectrometry using collision-induced dissociation and electron capture dissociation. In the present research, we examined the stability of the obtained analogues in CID and ECD experiments. Additionally, the impact of the peptide sequence including the effects of proline and aspartic acid on CID fragmentation of derivatized peptides were determined. The analyzed series of compounds, as presented by the obtained results, was sufficient to determine the fragmentation pattern in both the CID and ECD techniques. Although the analysis on the effect of the ionization tags on the CID spectra of the derivatized peptide has been already discussed in the literature, the ECD analysis presented in our manuscript has not been investigated yet.

## 2. Results and Discussion

**Figure 1:**
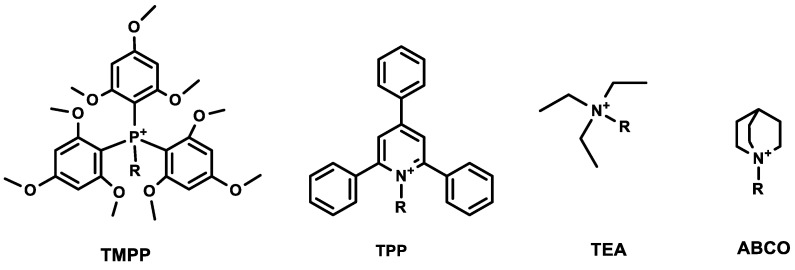
Structure of ionization tags (R—peptide sequence).

In our study, we examined the effect of both the quaternary ammonium (QAS)/phosphonium (QPS) group and the side chain of amino acid residues on the fragmentation pathway of a series of the model peptide with the H-GDGRTL-NH_2_ peptide analogues as an immunosuppressive ubiquitin fragment [29]. The major novelty of the presented manuscript is the presentation and comparison of the CID and ECD spectra for the peptide modified by different fixed-charge tags, which has not been presented before in the scientific literature. The obtained and presented data allowed for better insight into the fragmentation pattern of the charge-modified peptide. The model peptides were derivatized by different quaternary ammonium and phoshponium tags in the form of *N*,*N*,*N*-triethylammoniumacetyl (TEA), 1-azoniabicyclo[2.2.2]octylammoniumacetyl (ABCO), 2,4,6-triphenylpyridinium (TPP), and tris(2,4,6-trimethoxyphenyl) phosphonium (TMPP). The schematic presentation of chemical structures of ionization tags used in the experiments is presented in Figure 1. The analogues were designed using alanine scanning, where individual amino acid residues were exchanged for alanine to determine whether the side chain of a specific residue plays a significant role in the fragmentation pathway. Moreover, we examined the stability of ionization tags in tandem mass experiments and their influence on the possibility of peptide sequencing. We investigated the impact of the presence of proline and aspartic acid in peptides containing ionization tags on the formation of characteristic fragment ions.

**Scheme 1:**
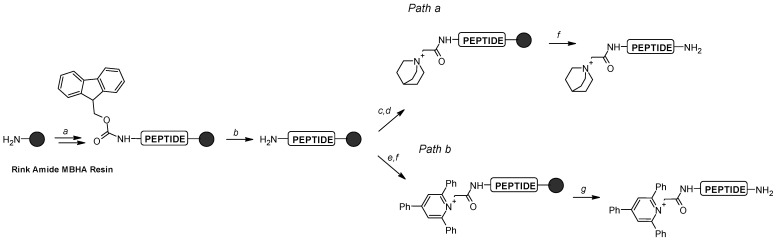
Synthetic approach: (**a**) SPPS-Aaa (3 eq), TBTU (3 eq), DIPEA (6 eq), 2 h, RT; (**b**) 25% piperidine in DMF (2 × 10 min), RT; (**c**) ICH_2_COOH (5 eq), DIC (5 eq), 3 × 30 min; (**d**) TEA (20 eq) or ABCO (20 eq) or TMPP (5 eq), 12 h; (**e**) Fmoc-Gly-OH (3 eq), DIPEA (6 eq), 2h, RT; (**f**) TPP (3 eq), TEA (3 eq), CH_3_COOH (3 eq), 2 h; (**g**) TFA:H_2_O:TIS (9.5:2.5:2.5; v:v:v), 2 h, RT).

Briefly, TPP salt was introduced by direct reaction of the amino group of the glycine residue with 2,4,6-triphenylpyrylium tetrafluoroborate in the presence of acetic acid as a catalyst (Scheme 1, path b) [19]. The TEA, ABCO, and TMPP groups were formed in a two-step reaction involving iodoacetylation of the amino group followed by the reaction with the appropriate tertiary amine or phosphine on the resin (Scheme 1, path a) [13,14]. After the synthesis, all analogues were removed from the resin and analyzed by mass spectrometry. Fragmentation experiments using both CID and ECD techniques were performed on quaternary ammonium and phosphonium salt conjugates (28 modified peptides) and seven unmodified peptides.

**Figure 2:**
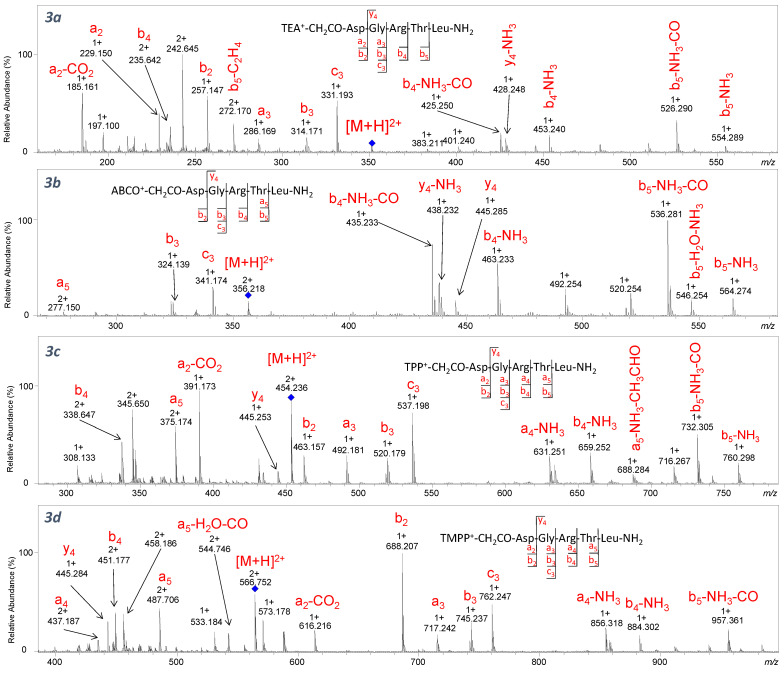
Representative ESI-CID-MS/MS spectra for [M+H]^2+^ ion of compounds **3a**–**3d** (for compounds: **3a**–**3c** collision energy: 20 eV; for compounds **3d**: 25 eV).

**Figure 3:**
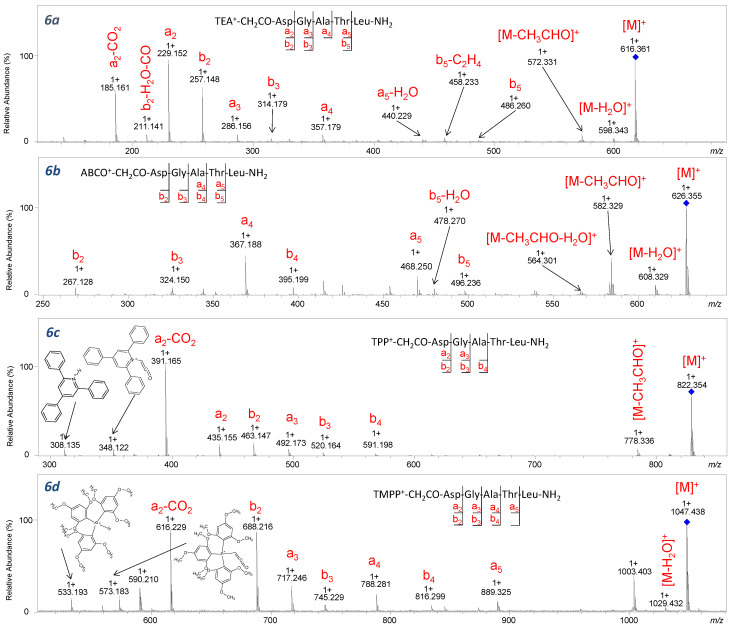
ESI-CID-MS/MS spectra for ion [M]^+^ of compounds **6a**–**6d** in collision energy: **6a**—35 eV, **6b**—38 eV, **6c**—35 eV, **6d**—50 eV.

In our analysis, the fragment of molecules containing the iodoacetic derivative of ammonium or pyridinium salts (QAS^+^-CH_2_CO^−^) was treated as the first amino acid residue, because betaine is considered to be an amino acid. For consistency and easier comparison of the obtained results, a similar assumption was made also for phosphonium salt (QPS^+^-CH_2_CO^−^). All identified fragments coming from the *N*-terminal part of the derivatized analogues contain ionization tags, therefore the series of ions *a*, *b*, *c* are not assigned additional ionization markers in MS/MS spectra. The series of fragmentation experiments ESI-CID-MS/MS for [M]^+^ and [M+H]^2+^ ions for all synthesized peptide derivatives were carried out and analytical data are presented in Table 1 and Table 2. Representative spectra of compounds **3a**–**3d** and **6a**–**6d **are presented in Figure 2 and Figure 3. In Supplementary Information (SI), we present the fragmentation spectra for all obtained peptides and their derivatized analogues (Figures S1–S70). For each compound, the tandem mass experiments were recorded in different collision energies. It was found that the relative abundances and numbers of the observed and identified fragment ions changed with the collisional energy. The fragmentation study revealed a series of intensive *a* and *b* ions for derivatized analogues formed in CID experiments. The observed results correlate well with the literature data described for other quaternary ammonium [13] and phosphonium derivatives [18] as well as for compounds containing the 2,4,6-triphenylpyridinium moiety [22]. The relative abundance of the precursor ion peak varies wildly from spectrum to spectrum, which indicates that many of the fragments (e.g., all *a*-type ions) come from secondary fragmentation. However, this does not impair the validity of our conclusions regarding the stability of QAS or the influence on the observed effects. The signals in the fragmentation spectra for derivatized analogues containing Arg, Asp, and Thr in the sequence are also accompanied by typical neutral losses such as a water molecule [M–18.011], ammonia [M–17.027], and an acetaldehyde molecule [M–44.026] (Figure 2 and Figure 3). Moreover, fragmentation of the side chain of Thr and loss of the neutral molecule of 44.026 Da was previously described by Wang et al. [30], which may occur in both protonated peptides and sodium adducts. In addition, in the case of *N*,*N*,*N*-triethylammoniumacetyl derivatives, an ion corresponding to a mass loss of 28.031 was noted (Figure 2a and Figure 3a), which indicates the elimination of one alkene (-C_2_H_4_) group from the *N*,*N*,*N*-triethylammonium group that appears as a result of the Hofmann elimination [31]. However, in our work, we noticed fewer ions from the Hofmann elimination, which can be explained by different sequences used in our fragmentation study. On the other hand, neutral losses observed for compounds **3b**–**d**–**7b**–**d** indicate the loss of the CO molecule [M–27.995] (Figure 2 and Figure 3). The fragmentation of QAS/QPS^+^-CH_2_CO-DGRTL-NH_2_ derivatives (Figure 2) mainly occurs between the third and fourth amino acid residues, forming *a*_3_, *b*_3_, and *c*_3_ fragment ions (the most abundant signal). Further analysis revealed that the signals corresponding to the *a*_4_, *a*_5_, *b*_4_, and *b*_5_ ions with a charge of 1+ are accompanied by the neutral losses. Moreover, in the spectra, only one signal corresponding to ion *y*_4_ formed from the C-terminus is observed.

**Figure 4:**
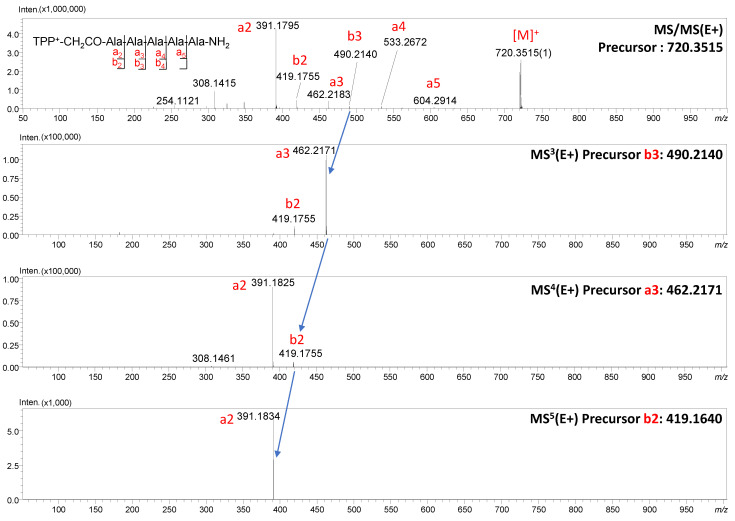
MS^5^ for TPP^+^-CH_2_CO-AAAAA-NH_2_ (**1c**) (in each step-collision energy 25 eV).

We noticed that with the increase in the value of the applied collision energy, the number of a-type ions increases, but also, in lower values of collision energy, intensive a ions are formed. To prove this occurrence, we performed MS^n^ multistage analysis, which provides means to link all product ions to specific precursor ions, hence enabling recursive reconstruction of fragmentation pathways that link specific sub-structures to complete molecular structures. The MS^5^ analysis for TPP^+^-CH_2_CO-AAAAA-NH_2_ is presented in Figure 4. The selected ions were fragmented in the next stages, which allows us to conclude the order in which the following fragments appear: *b*_3_ → *a*_3_ → *b*_2_ → *a*_2_. It is worth emphasizing that the a2 ion is dominant in the MS/MS spectrum ion, confirming the tendency of compounds containing salts at the *N*-terminus to generate a ions.

Figure 3 shows the spectra of the QAS/QPS^+^-CH_2_CO-GDGATL-NH_2_ analogues obtained after replacing the arginine residue with alanine at the third position. In the presented spectra, a series of *a* and *b* ions accompanied by low-intensity signals corresponding to the neutral losses are observed. For this sequence, the neutral loss of the CH_3_CHO molecule resulting from the fragmentation of the threonine residue side chain in each spectrum is observed. In spectra 6c and 6d, the signals from the fixed-charge tag dissociation are presented. Depending on the applied collision energy, different intensities of signals appearing after the dissociation of TPP at *m*/*z*: 391.165, 348.122, 320.131, and 308.135 are observed. The obtained results are similar to the conclusions proposed by Waliczek et al. [22]. The peak at *m*/*z* 320.131 mainly appears at collision energy higher than 35 eV (Figures S48, S50, S54 and S56). Moreover, higher collision energy results in an increase in the intensity of all signals from the ionization tag and a decrease in the signal intensity corresponding to the fragmentation of the peptide sequence. This phenomenon was used for the monitoring of unique reporter ions that formed after derivatization of the peptides present in trace amounts, with these being biomarkers of kidney disease and preeclampsia [24,26,27]. Similar behavior was noticed for phosphonium salt where signals at *m*/*z* 573.183, 616.229, 533.193, and 590.210 were observed, which confirms the charge remote fragmentation mechanism. For TMPP analogues containing an alanine residue as a first amino acid (1d, 2d, and 4d), fewer ions originating from phosphonium salt were observed (Figures S58, S60 and S64). However, regardless of the analyzed sequence, the fragmentation of peptides containing phosphonium salt (TMPP) leads to the formation of the abundant signal at *m*/*z* 616.229 (*a*_2_-CO_2_) and the low-intensity signal at *m*/*z* 573.183. The generation of the *a*_2_-CO_2_ ion for QAS/QPS^+^-peptide conjugates containing aspartic acid at the second position in the peptide may be explained by the impact of QAS/QPS on the decarboxylation (neutral loss of the CO_2_ molecule) taking place in the side chain of Asp. In our investigation, the Asp residue was introduced intentionally at the second position in the analyzed peptide sequence because a similar investigation, regarding the influence of the QAS group—trimethyl-, triethyl-, tripropyl-, and tributylammoniumacetyl (TMAA, TEAA, TPAA, and TBAA, respectively)—on the mechanism of peptide fragmentation was described. The authors proved that regardless of the QAS used (TMAA, TEAA, TPAA, TBAA), decarboxylation of the side chain of Asp residue was observed and the *a*_2_-CO_2_ ion appeared [13,31].

**Figure 5:**
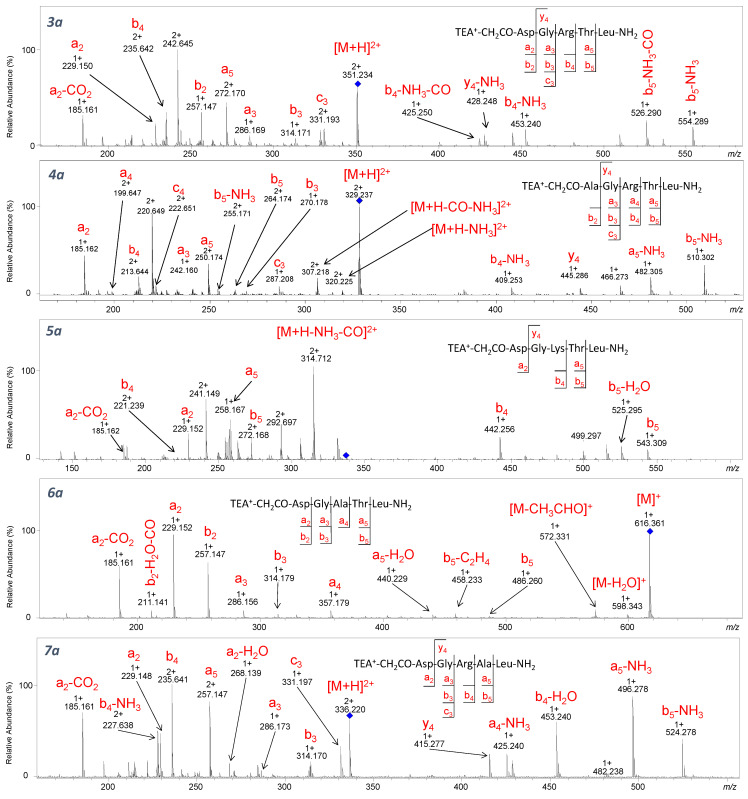
ESI-CID-MS/MS spectra for ions [M]^+^ and [M+H]^2+^ of compounds **3a**–**7a** in collision energy: **3a**—17 eV, **4a**—15 eV, **5a**—15 eV, **6a**—30 eV, **7a**—17 eV.

In the spectra of compounds **3a**–**7a** containing *N*,*N*,*N*-triethylammoniumacetyl group at the *N*-terminus (Figure 5), the neutral loss of a molecule of 28 Da is observed only for TEA^+^-CH_2_CO-Asp-Gly-Ala-Thr-Leu-NH_2_ conjugate. However, the obtained *m*/*z* values may correspond to both *b*_5_-C_2_H_4_ and *a*_5_ ions. We noticed that in the case of peptides containing arginine or lysine residue in their sequence, the fragmentation leads to the formation of the a5 ion, whereas for the peptide in which the arginine was replaced by alanine, the monoisotopic mass calculated for the *b*_5_-C_2_H_4_ ion (458.224 Da) corresponded to the *m*/*z* observed in the spectrum (458.233) and not with the mass calculated for the *a*_5_ ion (458.260 Da). This may suggest that the introduction of an additional proton-binding site in the peptide sequence (such as arginine or lysine residue) does not cause the loss of the alkene molecule from the quaternary ammonium group.

### 2.1. Effect of Proline

**Scheme 2:**
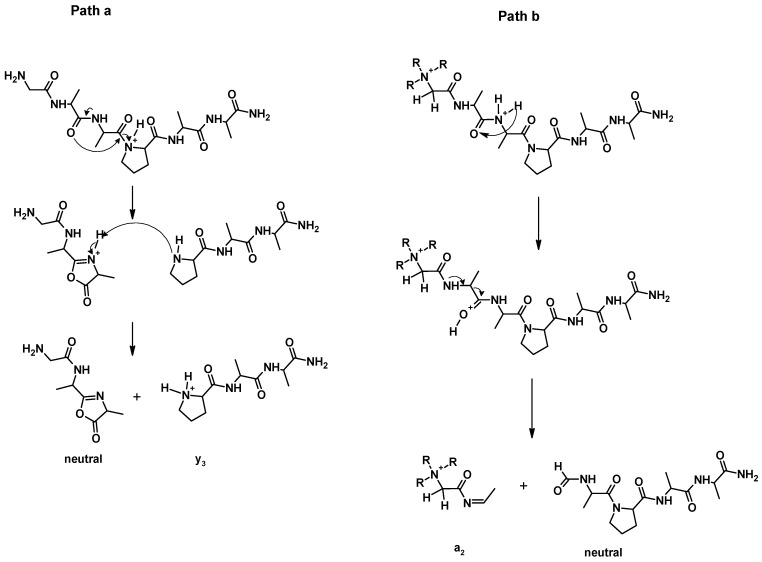
Mechanism proposal explaining: (**Path a**) Effect of proline for non-derivatized peptides containing Pro residue and (**Path b**) the formation of ions *a*_2_ on the example of QAS^+^-CH_2_CO-AAPAA-NH_2_ (**Path b**: Formation of *a*_2_ ions is independent of the peptide sequence containing QAS).

**Figure 6:**
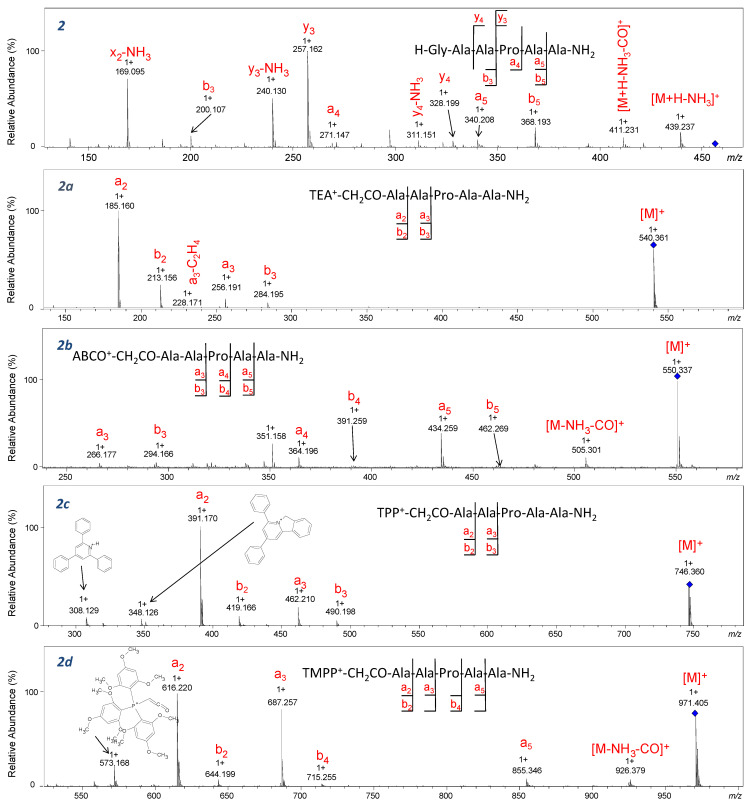
ESI-CID-MS/MS spectra for ions [M]^+^ of compounds **2a**–**2d** in collision energy: **2**—17 eV, **2a**—25 eV, **2b**—40 eV, **2c**—20 eV, **2d**—50 eV.

The proline effect was investigated for QAS/QPS^+^-CH_2_CO-GAAPAA-NH_2_, QAS/QPS^+^-CH_2_CO-GAAAAA-NH_2_, and their non-modified analogues. In CID experiments for H-GAAPAA-NH_2_ (Figure 6a), the fragmentation of protonated and non-modified peptides containing the proline residue in the sequence leads to the formation of an intense *y*_3_ ion, which is the consequence of dissociation of the amide bond at the proline residue. The proline effect is explained by the high affinity of the proton to the tertiary amide nitrogen atom of a proline residue [5,6]. In this case, proline is located at the C-terminus of the peptide chain, and the formed *b* ions have a bicyclic structure (Scheme 2, path a) [7]. In the ESI-MS/MS spectrum of H-GAAPAA-NH_2_ (2), the formation of C-terminal fragments (*x*_2_, *y*_3_, *y*_4_) was observed, and among the results, the *y*_3_ fragment ion (*m*/*z* 257.162) has the highest intensity, which proves the proline effect. In our study, we performed CID experiments for both [M]^+^ and [M+H]^2+^ ions corresponding to QAS/QPS^+^-peptides. For the derivatized analogues in which precursor ions [M]^+^ were fragmented, the characteristic signal corresponding to the *y*_3_ ion confirming the proline effect was not identified, resulting in inhibition of the proline effect. A similar observation was described for the ion [M+H]+ of peptides containing Arg residue subjected to CID experiments [32]. In mass spectra of TEA^+^-CH_2_CO-AAPAA-NH_2_ (2a) and TPP^+^-CH_2_CO-AAPAA-NH_2_ (2c), only the formation of ions at *m*/*z* corresponding to *a*_2_, *a*_3_, *b*_2_, and *b*_3_ resulting from the fragmentation of the second or third amide bond is observed. The formation of abundant *a*_2_ ion was observed for the majority of the investigated quaternary salts, therefore we assumed that this reaction is independent on the proline and is caused by the proximity of the positive charge located on the *N*-terminus. The mechanistic details of this reaction were not studied; however, based on Eckert’s [33] observation regarding the behavior of cyclic peptides in CID conditions, we postulate a proton transfer from (R)3N^+^-CH_2_-CO-peptide-NH_2_ to the carbonyl group of the next amino acid, and then dissociation of the bond with the formation of formyl in the neutral part of the molecule and an intense a_2_ ion (Scheme 2, path b). A similar mechanism was postulated by Rudowska et al. for oligoproline containing the quaternary ammonium group [34].

**Figure 7:**
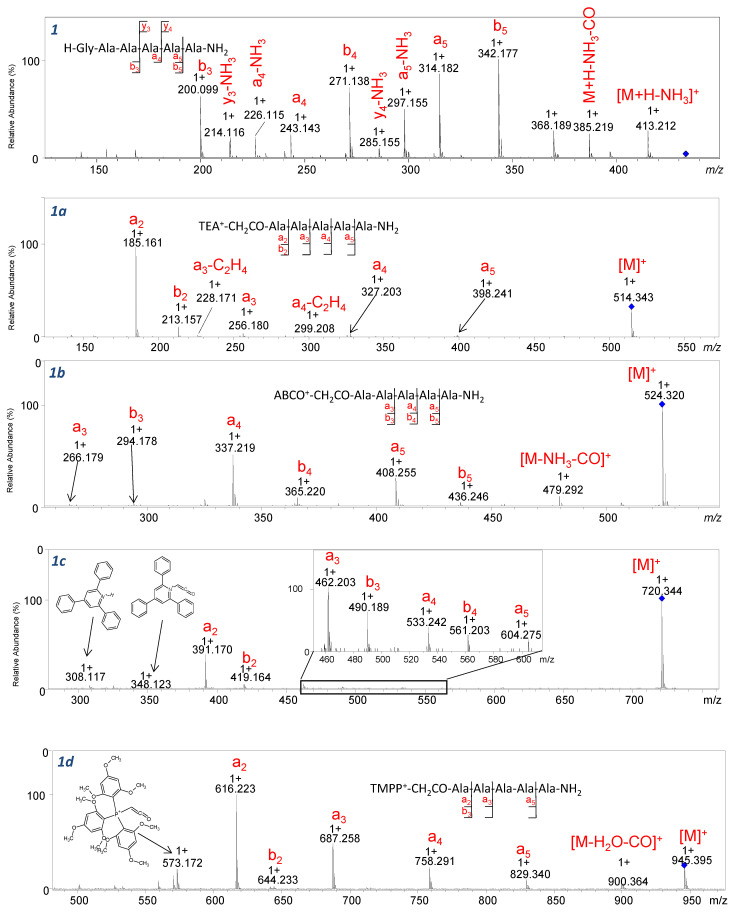
ESI-CID-MS/MS spectra for ions [M]^+^ of compounds **1a**–**1d** in collision energy: **1**—15 eV, **1a**—27 eV, **1b**—37 eV, **1c**—30 eV, **1d**—55 eV.

**Figure 8:**
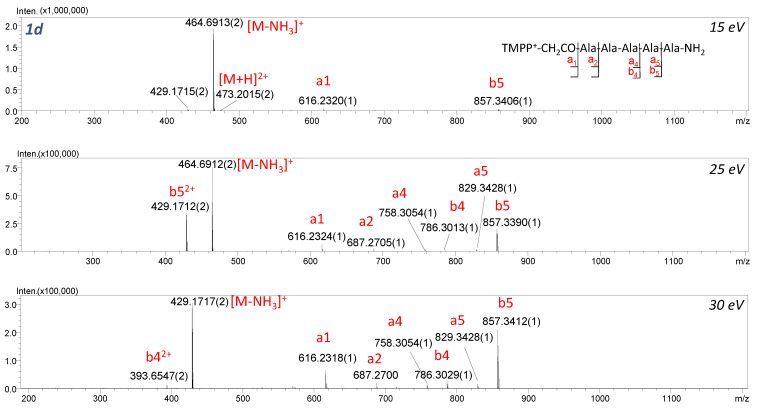
ESI-CID-MS/MS spectra of TMPP^+^-CH_2_CO-Ala-Ala-Ala-Ala-Ala-NH_2_ (**1d**). Precursor ion [M+H]^2+^ at *m*/*z* 473.2015.

**Figure 9:**
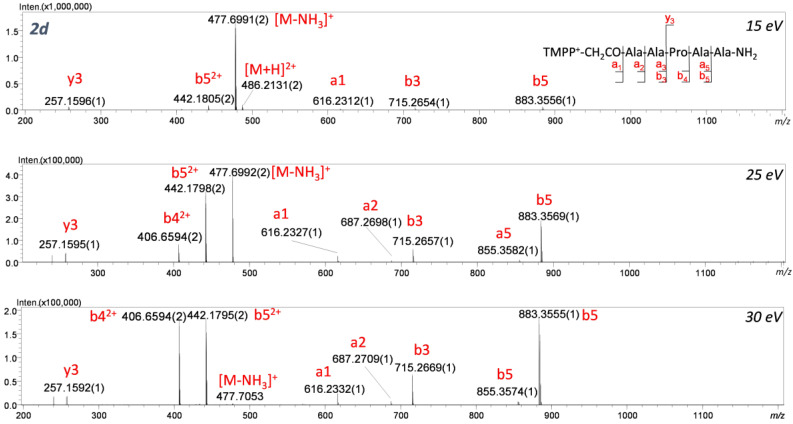
ESI-CID-MS/MS spectra of TMPP^+^-CH_2_CO-Ala-Ala-Pro-Ala-Ala-NH_2_ (**2d**). Precursor ion [M+H]^2+^ at *m*/*z* 486.208.

Going further, fragmentation of ABCO^+^-CH_2_CO-AAPAA-NH_2_ (2b) and TMPP^+^-CH_2_CO-AAPAA-NH_2_ (2d) compounds is accompanied by the formation of fragments of further peptide bonds. Comparing the spectra shown in Figure 6 to the spectra of compounds **1a**–**1d** (Figure 7), we can conclude that regardless of the presence of a proline residue in the analyzed sequences, a series of intense a-type ions was observed. For [M]+ ions of the peptide sequence conjugated with QAS/QPS fixed-charge tags subjected to CID analysis, the proline effect is inhibited. The fragmentation pathway observed for [M]+ occurs according to the ChR processes in which the charge is fixed on one particular atom (QAS) possessing high proton affinity. Our further study was performed on the parent ion [M+H]^2+^ isolated for TMPP^+^-CH_2_CO-AAAAA-NH_2_ (Figure 8) and TMPP^+^-CH_2_CO-AAPAA-NH_2_ (Figure 9) to determine the competition between ChR and ChD mechanisms. In ChD, a reaction occurs for the peptide containing a proton attached to an amino group, which can migrate from one position to another. The fragmentation spectra recorded in different collision energy for 2d are presented in Figure 9. The spectra are dominated by an abundant series of b and a-type ions but also a low-intensity y_3_ ion confirming the proline effect. It can be clearly stated that the ChR mechanism is strongly favored and the location of QAS at the *N*-terminus significantly weakens the proline effect. We can conclude that the analyzed ionization markers lead to similar effects and can be treated as Arg substitutes. Huang et al. [31] observed that for peptides with Arg in the sequence and charge 1+, the proline effect is not observed, whereas for peptides without Arg in their sequence and charge 2+, the proline effect was noticeable. Moreover, for peptides without Arg but possessing charge 1+ (e.g., because of the presence of Lys), the proline effect was also noted.

### 2.2. Effect of Aspartic Acid

Multiple protonated peptides, due to peptide bond breaking after the aspartic or glutamic acid residues, can form fragment b-type ions, which undergo cyclization at the C-terminus. As a result of proton transfer from the carboxylic group on the nitrogen atom of the amide bond, a five-membered ring of succinimide anhydride is formed [2]. The presence of a strongly basic guanidine group of arginine residue limits the mobility of mobile protons in a peptide chain [3]. In addition, introducing a stable positive charge into the peptide chain can eliminate proton mobility.

**Scheme 3:**
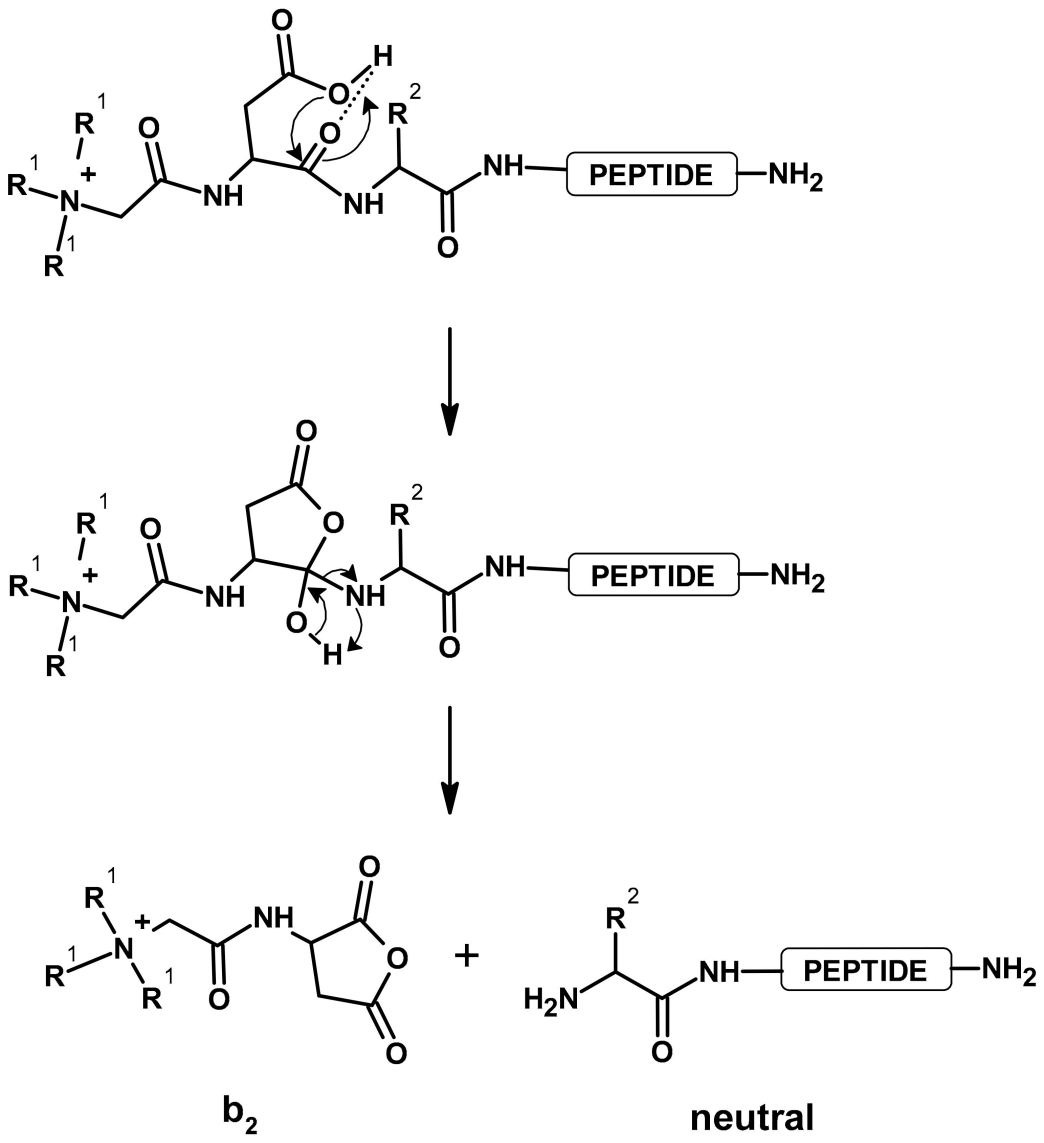
Proposed mechanism of formation of b_2_ ion in derivatized peptides containing Asp residue at position 2.

**Figure 10:**
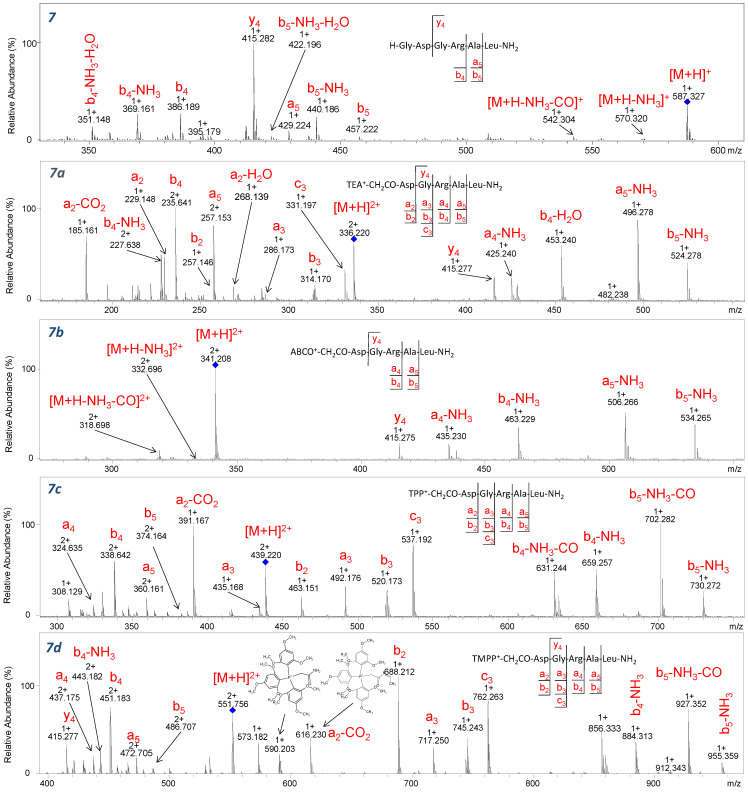
ESI-CID-MS/MS spectra for ion [M+H]^2+^ of compounds **7a**–**7d** in collision energy: **7**—30 eV, **7a**—17 eV, **7b**—15 eV, **7c**—20 eV, **7d**—25 eV.

In Figure 10, the fragmentation spectra of the H-GDGRAL-NH_2_ peptide and its derivatized analogues were presented. The influence of aspartic acid was investigated based on the intensity of characteristic *b*_2_ ions formed during experiments. The *b*_2_ ion was identified for peptides containing Asp residue and the following fixed-charge tags: TEA, TPP, and TMPP. The proposed mechanism of formation of the *b*_2_ ion for derivatized peptides, which has been already presented by Gu et al. [4], is shown in Scheme 3. In the case of analogues derivatized with ABCO, the *b*_2_ ion was only observed for the H-GDGATL-NH_2_ (Figure S40) sequence in which no arginine or lysine residue is present. The fragmentation spectra of peptides derivatized with the 2,4,6-triphenylpyridinium group (Figures S48, S52, S54 and S56) revealed the low-intensity signal corresponding to the *b*_2_ ion at *m*/*z* 463.151, proving slight effect of aspartic acid. In the case of peptides modified by the TMPP group, the *b*_2_ ion (*m*/*z* 688.212) is dominant in the spectra, which are shown in Figures S62, S68 and S70. Such an observation allows us to conclude unequivocally that the type of fixed-charge tag used has an impact on the presence of the aspartic acid effect. Moreover, a significant influence of the aspartic acid effect was observed for the modified peptides without basic amino acids in their sequence because the salt bridges are not formed and dissociation after aspartic acid can occur. Further analysis of the aspartic acid effect was performed on peptides containing Ala residue instead of Asp at position 2. It can be expected that the lack of aspartic acid in this position should inhibit the formation of the *b*_2_ fragment that was observed for AGRTL derivatized with TEA, ABCO, and TPP groups (Figures S22, S36 and S50). In the case of fragmentation of the TMPP^+^-CH_2_CO-AGRTL-NH_2_ derivative (Figure S64) beside the intensive *a*_2_ ion, the signal at *m*/*z* 644.221 corresponding to the *b*_2_ ion is observed. It is likely that the signal corresponding to this ion results from the preferences of the quaternary phosphonium group, and in order to unambiguously explain this, another series of analogues containing other amino acid residues at position 2 should be tested. Recently, we analyzed the fragmentation pathways of the model peptide derivatized by 5-azoniaspiro[4.4]nonyl or benzo-5-azoniaspiro[4.4]nonyl ionization enhancers at the N-terminus [35,36]. The obtained results indicate a similar tendency in the ESI-MS/MS spectra recorded for peptide derivatives in the form of ASN^+^-CO-VESYVPLFP-OH and BASN^+^-CO-VESYVPLFP-OH. Each of the analyzed azoniaspiro peptide derivatives produced an intense signal corresponding to the a_2_* and b_2_* ions, which may be used as characteristic ions in LC-ESI-MRM analysis. Therefore, it may be assumed that the fragmentation pathways are not predictable and depend on several factors, which make their detailed analysis important.

### 2.3. ECD Analysis

**Figure 11:**
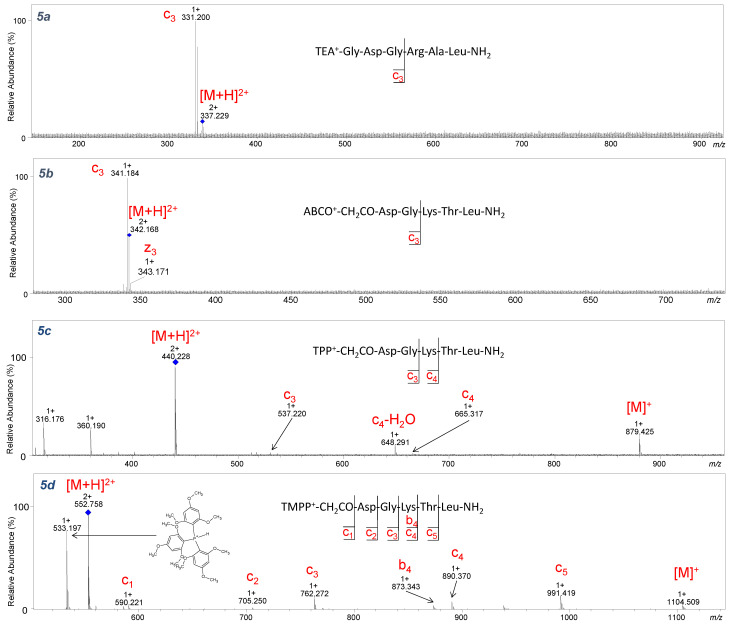
ECD-MS for the ion [M+H]^2+^ of compounds **5a**–**5d**.

**Figure 12:**
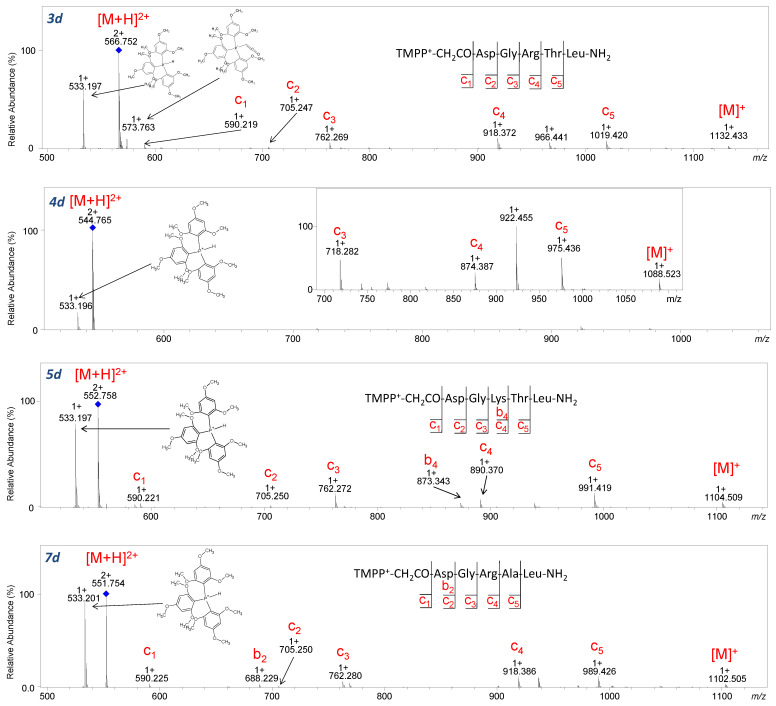
ECD-MS for the ion [M+H]^2+^ of compounds **3d**, **4d**, **5d** and **7d**.

During the ECD experiment, the charge of a peptide ion is reduced as a result of electron transfer. Therefore, only multiple-charged (z ≥ +2) molecules could be analyzed by this technique [37]. In our study, the ECD technique was used to check the impact of the introduced fixed-charge tag on the fragmentation pattern. The product ions identified in ECD fragmentation of the studied peptides (**3**–**5a**–**d**, **7a**–**7d**) are shown in Figure 11, Figure 12, and Figures S71–S86. During the analysis, one limitation was the relatively low *m*/*z* value (below 350 for charge 2+) and low intensity of the [M+H]^2+^ ion corresponding to peptides with TEA and ABCO groups, which impeded fragmentation (Figure 10 and Figures S71–S78). However, an intensive signal corresponding to the *c*_3_ ion at *m*/*z* 331.200 (**5a**–Figure 11) and *m*/*z* 341.182 (**5b**–Figure 11) was observed. This can be explained by the fact that basic amino acid residues such as arginine and lysine or an ionization tag may contribute to fragmentation. Attaching a proton to the basic amino acid residue detaches and neutralizes the peptide fragment, thereby generating *c*_3_ ion. Moreover, the low-intensity *z*_3_ ion from the C-terminus of the ABCO+-conjugate (**5b**–Figure 11, 3d–Figure S75, **5d**–Figure S77) was identified. A characteristic series of c ions covering the peptide sequence in ECD experiments was observed for peptides with TPP and TMPP (Figure 11) groups because the *m*/*z* values for those compounds were above 400 (charge 2+), allowing ion isolation and fragmentation.

It was surprising that for peptides with the TMPP group (Figure 12), a characteristic ion at *m*/*z* 533.197 from the decomposition of the tris(2,4,6-trimethoxyphenyl) phosphonium group appeared in both CID and ECD techniques. It is worth emphasizing that no other quaternary group produced ions during ECD fragmentation. The ECD spectra of the investigated peptides are less complicated compared to their CID spectra. The number of formed fragments is lower because the losses of small molecules are limited.

## 3. Materials and Methods

All details concerning applied reagents and equipment are presented in Supplementary Information.

### 3.1. Synthetic Protocol of QAS and QPS Peptide Conjugates

The model peptides were synthesized manually in polypropylene syringe reactors (Intavis AG) equipped with polyethylene filters, according to a standard Fmoc (9-fluorenylmethoxycarbonyl) solid phase synthesis procedure [38] using 30 mg of Rink Amide Resin (loading 0.68 mmol/g) for each synthesis. Coupling was performed using Fmoc-protected amino acid (3 eq), TBTU (2-(1*H*-Benzotriazole-1-yl)-1,1,3,3-tetramethylaminium tetrafluoroborate) (3 eq), and DIPEA (*N*,*N*-diisopropylethylamine) (6 eq) for 2 h at room temperature. Each coupling step was monitored by the Kaiser test [39]. After synthesis, the peptides were derivatized with ABCO, TEA, or TPP using protocols published by Cydzik et al. [13], Setner et al. [14], and Waliczek et al. [19], respectively. In order to attachment tris(2,4,6-trimetoxyphenylo)phosphane to the *N*-terminus, we used the same protocol as ABCO while applying 5 eq of reagent. After the reaction was completed, the resin was washed with DMF (7 × 1 min), DCM (3 × 1 min), and MeOH (3 × 1 min), and dried in vacuo. The last step consisted of drying a peptydyl resin in a vacuum desiccator for one day at room temperature. The side-chain deprotection and cleavage of the derivatized peptides from the resin were accomplished using a solution of TFA/H_2_O/TIS (95/2.5/2.5, *v*/*v*/*v*) at room temperature for 2 h. After evaporating trifluoracetic acid, the products were lyophilized and analyzed by analytical methods: ESI-MS, ESI-CID-MS/MS, and ESI-ECD-MS/MS.

### 3.2. ESI-MS Analysis

#### 3.2.1. CID

The CID experiments were performed using an Apex-Qe 7T Bruker instrument (Bremen, Germany) equipped with a standard ESI source. Spectra were recorded using aqueous solutions of acetonitrile (50%) and formic acid (0.1%), at the peptide concentration of 5 μM. The instrument’s parameters were as follows: Positive-ion mode, calibration with the Tunemix™ mixture (Agilent Technologies, Palo Alto, CA, USA), mass accuracy was better than 5 ppm, scan range: 50–1600 *m*/*z*; drying gas: Nitrogen; flow rate: 4.0 L/min, temperature: 200 °C; potential between the spray needle and the orifice: 4.5 kV, analyte was introduced to the ESI source by KDScientific pump (Holliston, MA, USA) with a flow rate: 3 µL/min).

In the MS/MS mode, the quadrupole was used to select the precursor ions, which were fragmented in the hexapole collision cell applying argon as the collision gas. The obtained fragments were subsequently analyzed by the ICR mass analyzer. For the MS/MS measurements, the voltage over the hexapole collision cell varied from 15 to 40 V.

#### 3.2.2. ECD

The ECD experiments were performed using an Apex-Qe 7T Bruker instrument (Bremen, Germany) equipped with the ESI source and a heated hollow cathode dispenser, using standard Bruker ECD settings. The quadrupole was used to select the precursor ions, which were fragmented in the ICR cell. The cathode dispenser was heated gradually to 1.7 A. The ECD pulse length was set at 300 ms., and ECD bias was 1 V. Analysis of the obtained spectra was carried out with Biotools (Bruker, Bremen, Germany) software.

#### 3.2.3. MS^n^ Analysis

The MS^n^ analysis was performed on the Shimadzu LCMS-IT-TOF system equipped with a Nexera X2 chromatographic module. The instruments’ parameters were as follows: Ion mode, calibration with the Tune Solution, LCMS-IT-TOF (Shimadzu Corporation, Kyoto, Japan), mass accuracy was better than 5 ppm, scan range: 50–2000 *m*/*z*; drying gas: nitrogen (flow rate: 1.5 L/min), temperature: 200 °C; solvents: Isocratic elution 50% B in A (A = 0.1% HCOOH in water; B = 0.1% HCOOH in MeCN), flow rate: 0.1 µL/min.

## 4. Conclusions

A comparative study of the influence of ionization tags (quaternary ammonium groups (TEA, ABCO, TPP) and one phosphonium group (TMPP)) and peptide sequences on the fragmentation of model peptides using CID and ECD experiments was performed. The series of peptides based on the H-DGRTL-NH_2_ sequence in which individual amino acid residue was exchanged for an alanine were synthesized on a solid support.

Analysis of the obtained peptides in the CID experiment showed that fragmentation proceeds with the loss of neutral molecules such as water, ammonia, carbon monoxide, as well as loss of the acetaldehyde molecule, which results from the fragmentation of the side chain of threonine residue. Moreover, aspartic acid or alanine residue located in the second position of peptides containing a positive charge at the *N*-terminus is privileged to generate abundant a_2_ or *a*_2_-CO_2_ ions.

In contrast to the ABCO group, which is stable under the applied conditions, for peptides with the TPP and TMPP groups, a characteristic series of fragments originating from the ionization tags appeared. The intensity of signals increased with increasing collision energy, and at the same time, the intensity of the fragments from the peptide sequence decreased. In the case of the aliphatic quaternary ammonium group (TEA), ions were formed due to the fragmentation of the ionization tag according to the Hofmann elimination, making the obtained fragmentation spectra analysis difficult but only for sequences without basic amino acid residues such as Arg or Lys.

We also analyzed the impact of a peptide sequence on the fragmentation pattern of peptides containing a stable positive charge—QAS or QPS. We observed that the effect of proline in the derivatized peptides is inconsequential in comparison to the *N*-modified peptides. The effect of the aspartic acid is evident for peptides possessing TPP and TEA groups. However, the effect of the aspartic acid was not resolved for the peptides derivatized with the phosphonium group because, in the case of the analogues where the Asp residue was replaced by alanine, an intense *b*_2_ ion also appeared, which may suggest the fragmentation preferences of the TMPP group.

ECD spectra of peptides containing TPP and TMPP groups are dominated by *c_n_* ions providing sufficient information for the sequencing of peptides. Very promising results were obtained for the ECD analysis of phosphonium group analogues because an intense fragment derived from the TMPP group was formed and it may be used as a reporter ion in LC-ECD-MS studies. Application of each of the presented fixed-charge tags may significantly improve the qualitative analysis of peptides and the final determination of their chemical structure by tandem mass spectrometry.

The presented detailed analysis of fragmentation patterns of the analyzed compounds may allow one to determine the final mechanism of fragmentation of fixed-charge tagged peptides. The obtained results allowed the discovery and comparison of the fragmentation patterns of analyzed compounds in CID and ECD modes, and therefore, they may be useful in the analysis of more complex mixtures facilitating MS/MS data interpretation.

## Figures and Tables

**Table 1:** Analytical data for model peptides.

Nr.	Peptide Sequence	[M+H]^+^ Found	[M+H]^+^ Calc.	[M+2H]^2+^ Found	[M+2H]^2+^ Calc.
**1**	H-GAAAAA-NH_2_	430.242	430.241	-	-
**2**	H-GAAPAA-NH_2_	456.257	456.257	-	-
**3**	H-GDGRTL-NH_2_	617.341	617.337	309.181	309.172
**4**	H-GAGRTL-NH_2_	573.340	573.347	287.176	287.177
**5**	H-GDGKTL-NH_2_	589.336	589.330	295.181	295.169
**6**	H-GDGATL-NH_2_	532.284	532.273	-	-
**7**	H-GDGRAL-NH_2_	587.327	587.326	-	-

**Table 2:** Analytical data for QAS and QPS peptide conjugates.

Nr.	Sequences of QAS and QPS Peptide Conjugates	[M]^+^ Found	[M]^+^ Calc.	[M+H]^2+^ Found	[M+H]^2+^ Calc.
**1a**	TEA^+^-CH_2_CO-AAAAA-NH_2_	514.343	514.335	-	-
**1b**	ABCO^+^-CH_2_CO-AAAAA-NH_2_	524.320	524.319	-	-
**1c**	TPP^+^-CH_2_CO-AAAAA-NH_2_	720.344	720.350	-	-
**1d**	TMPP^+^-CH_2_CO-AAAAA-NH_2_	945.395	945.401	473.205	473.204
**2a**	TEA^+^-CH_2_CO-AAPAA-NH_2_	540.361	540.350	-	-
**2b**	ABCO^+^-CH_2_CO-AAPAA-NH_2_	550.337	550.335	-	-
**2c**	TPP^+^-CH_2_CO-AAPAA-NH_2_	746.360	746.366	-	-
**2d**	TMPP^+^-CH_2_CO-AAPAA-NH_2_	971.405	971.416	486.213	486.212
**3a**	TEA^+^-CH_2_CO-DGRTL-NH_2_	701.446	701.431	351.234	351.219
**3b**	ABCO^+^-CH_2_CO-DGRTL-NH_2_	711.416	711.415	356.221	356.211
**3c**	TPP^+^-CH_2_CO-DGRTL-NH_2_	907.439	907.446	454.236	454.227
**3d**	TMPP^+^-CH_2_CO-DGRTL-NH_2_	1132.483	1132.494	566.752	566.752
**4a**	TEA^+^-CH_2_CO-AGRTL-NH_2_	657.458	657.441	329.237	329.224
**4b**	ABCO^+^-CH_2_CO-AGRTL-NH_2_	667.425	667.425	334.224	334.216
**4c**	TPP^+^-CH_2_CO-AGRTL-NH_2_	863.443	863.456	432.236	432.232
**4d**	TMPP^+^-CH_2_CO-AGRTL-NH_2_	1088.509	1088.506	544.765	544.757
**5a**	TEA^+^-CH_2_CO-DGKTL-NH_2_	673.439	673.424	337.229	337.216
**5b**	ABCO^+^-CH_2_CO-DGKTL-NH_2_	683.418	683.409	342.218	342.208
**5c**	TPP^+^-CH_2_CO-DGKTL-NH_2_	879.425	879.440	440.224	440.224
**5d**	TMPP^+^-CH_2_CO-DGKTL-NH_2_	1104.509	1104.490	552.758	552.749
**6a**	TEA^+^-CH_2_CO-DGATL-NH_2_	616.361	616.366	-	-
**6b**	ABCO^+^-CH_2_CO-DGATL-NH_2_	626.355	626.350	-	-
**6c**	TPP^+^-CH_2_CO-DGATL-NH_2_	822.361	822.382	-	-
**6d**	TMPP^+^-CH_2_CO-DGATL-NH_2_	1047.438	1047.440	-	-
**7a**	TEA^+^-CH_2_CO-DGRAL-NH_2_	671.409	671.420	336.220	336.214
**7b**	ABCO^+^-CH_2_CO-DGRAL-NH_2_	681.411	681.404	341.216	341.206
**7c**	TPP^+^-CH_2_CO-DGRAL-NH_2_	877.421	877.436	439.220	439.221
**7d**	TMPP^+^-CH_2_CO-DGRAL-NH_2_	1102.505	1102.486	551.754	551.745

## Data Availability

Not applicable.

## Supplementary Materials

The supplementary information is available online, Table S1: Analytical data for model peptides, Table S2: Analytical data for QAS and QPS peptide conjugates, Figure S1: ESI-MS spectrum of H-Gly-Ala-Ala-Ala-Ala-Ala-NH_2_ (**1**) Figure S2: ESI-CID-MS/MS spectra of H-Gly-Ala-Ala-Ala-Ala-Ala-NH_2_ (**1**). Precursor ion at *m/z* 430.242, Figure S3: ESI-MS spectrum of H-Gly-Ala-Ala-Pro-Ala-Ala-NH_2_ (**2**), Figure S4: ESI-CID-MS/MS spectra of H-Gly-Ala-Ala-Pro-Ala-Ala-NH_2_ (**2**). Precursor ion at *m/z* 456.257, Figure S5: ESI-MS spectrum of H-Gly-Asp-Gly-Arg-Thr-Leu-NH_2_ (**3**), Figure S6: ESI-CID-MS/MS spectra of H-Gly-Asp-Gly-Arg-Thr-Leu-NH2 (**3**). Precursor ion at *m/z* 309.181, Figure S7: ESI-MS spectrum of H-Gly-Ala-Gly-Arg-Thr-Leu-NH_2_ (**4**), Figure S8: ESI-CID-MS/MS spectra of H-Gly-Ala-Gly-Arg-Thr-Leu-NH_2_ (**4**). Precursor ion at *m/z* 573.340, Figure S9: ESI-MS spectrum of H-Gly-Asp-Gly-Lys-Thr-Leu-NH_2_ (**5**), Figure S10: ESI-CID-MS/MS spectra of H-Gly-Asp-Gly-Lys-Thr-Leu-NH_2_ (**5**). Precursor ion at *m/z* 589.336, Figure S11: ESI-MS spectrum of H-Gly-Asp-Gly-Ala-Thr-Leu-NH_2_ (**6**), Figure S12: ESI-CID-MS/MS spectra of H-Gly-Asp-Gly-Ala-Thr-Leu-NH_2_ (**6**). Precursor ion at *m/z* 532.294, Figure S13: ESI-MS spectrum of H-Gly-Asp-Gly-Lys-Ala-Leu-NH_2_ (**7**), Figure S14: ESI-CID-MS/MS spectra of H-Gly-Asp-Gly-Arg-Ala-Leu-NH_2_ (**7**). Precursor ion at *m/z* 587.327, Figure S15: ESI-MS spectrum of TEA^+^-CH_2_CO-Ala-Ala-Ala-Ala-Ala-NH_2_ (**1a**), Figure S16: ESI-CID-MS/MS spectra of TEA^+^-CH_2_CO-Ala-Ala-Ala-Ala-Ala-NH_2_ (**1a**). Precursor ion at *m/z* 514.323, Figure S17: ESI-MS spectrum of TEA^+^-CH_2_CO-Ala-Ala-Pro-Ala-Ala-NH_2_ (**2a**), Figure S18: ESI-CID-MS/MS spectra of TEA^+^-CH_2_CO-Ala-Ala-Pro-Ala-Ala-NH_2_ (**2a**). Precursor ion at *m/z* 540.361, Figure S19: ESI-MS spectrum of TEA^+^-CH_2_CO-Asp-Gly-Arg-Thr-Leu-NH_2_ (**3a**), Figure S20: ESI-CID-MS/MS spectra of TEA^+^-CH_2_CO-Asp-Gly-Arg-Thr-Leu-NH_2_ (**3a**). Precursor ion at *m/z* 351.234, Figure S21: ESI-MS spectrum of TEA^+^-CH_2_CO-Ala-Gly-Arg-Thr-Leu-NH_2_ (**4a**), Figure S22: ESI-CID-MS/MS spectra of TEA^+^-CH_2_CO-Ala-Gly-Arg-Thr-Leu-NH_2_ (**4a**). Precursor ion at *m/z* 329.237, Figure S23: ESI-MS spectrum of TEA^+^-CH_2_CO-Asp-Gly-Lys-Thr-Leu-NH_2_ (**5a**), Figure S24: ESI-CID-MS/MS spectra of TEA^+^-CH_2_CO-Asp-Gly-Lys-Thr-Leu-NH_2_ (**5a**). Precursor ion at *m/z* 337.229, Figure S25: ESI-MS spectrum of TEA^+^-CH_2_CO-Asp-Gly-Ala-Thr-Leu-NH_2_ (**6a**), Figure S26: ESI-CID-MS/MS spectra of TEA^+^-CH_2_CO-Asp-Gly-Ala-Thr-Leu-NH_2_ (**6a**). Precursor ion at *m/z* 616.361, Figure S27: ESI-MS spectrum of TEA^+^-CH_2_CO-Asp-Gly-Arg-Ala-Leu-NH_2_ (**7a**), Figure S28: ESI-CID-MS/MS spectra of TEA^+^-CH_2_CO-Asp-Gly-Arg-Ala-Leu-NH_2_ (**7a**). Precursor ion at *m/z* 336.221, Figure S29: ESI-MS spectrum of ABCO^+^-CH_2_CO-Ala-Ala-Ala-Ala-Ala-NH_2_ (**1b**), Figure S30: ESI-CID-MS/MS spectra of ABCO^+^-CH_2_CO-Ala-Ala-Ala-Ala-Ala-NH_2_ (**1b**). Precursor ion at *m/z* 524.313, Figure S31: ESI-MS spectrum of ABCO^+^-CH_2_CO-Ala-Ala-Pro-Ala-Ala-NH_2_ (**2b**), Figure S32: ESI-CID-MS/MS spectra of ABCO^+^-CH_2_CO-Ala-Ala-Pro-Ala-Ala-NH_2_ (**2b**). Precursor ion at *m/z* 550.339, Figure S33: ESI-MS spectrum of ABCO^+^-CH_2_CO-Asp-Gly-Arg-Thr-Leu-NH_2_ (**3b**), Figure S34: ESI-CID-MS/MS spectra of ABCO^+^-CH_2_CO-Asp-Gly-Arg-Thr-Leu-NH_2_ (**3b**). Precursor ion at *m/z* 356.218, Figure S35: ESI-MS spectrum of ABCO^+^-CH_2_CO-Ala-Gly-Arg-Thr-Leu-NH_2_ (**4b**), Figure S36: ESI-CID-MS/MS spectra of ABCO^+^-CH_2_CO-Ala-Gly-Arg-Thr-Leu-NH_2_ (**4b**). Precursor ion at *m/z* 334.224, Figure S37: ESI-MS spectrum of ABCO^+^-CH_2_CO-Asp-Gly-Lys-Thr-Leu-NH_2_ (**5b**), Figure S38: ESI-CID-MS/MS spectra of ABCO^+^-CH_2_CO-Asp-Gly-Lys-Thr-Leu-NH_2_ (**5b**). Precursor ion at *m/z* 342.214, Figure S39: ESI-MS spectrum of ABCO^+^-CH_2_CO-Asp-Gly-Ala-Thr-Leu-NH_2_ (**6b**), Figure S40: ESI-CID-MS/MS spectra of ABCO^+^-CH_2_CO-Asp-Gly-Ala-Thr-Leu-NH_2_ (**6b**). Precursor ion at *m/z* 626.355, Figure S41: ESI-MS spectrum ABCO^+^-CH_2_CO-Asp-Gly-Arg-Ala-Leu-NH_2_ (**7b**), Figure S42: ESI-CIDMS/MS spectra of ABCO^+^-CH_2_CO-Asp-Gly-Arg-Ala-Leu-NH_2_ (**7b**). Precursor ion at *m/z* 341.206, Figure S43: ESI-MS spectrum of TPP^+^-CH_2_CO-Ala-Ala-Ala-Ala-Ala-NH_2_ (**1c**), Figure S44. ESI-CID-MS/MS spectra of TPP^+^-CH_2_CO-Ala-Ala-Ala-Ala-Ala-NH_2_ (**1c**). Precursor ion at *m/z* 720.344, Figure S45: ESI-MS spectrum of TPP^+^-CH_2_CO-Ala-Ala-Pro-Ala-Ala-NH_2_ (**2c**), Figure S46: ESI-CID-MS/MS spectra of TPP^+^-CH_2_CO-Ala-Ala-Pro-Ala-Ala-NH_2_ (**2c**). Precursor ion at *m/z* 746.360, Figure S47: ESI-MS spectrum of TPP^+^-CH_2_CO-Asp-Gly-Arg-Thr-Leu-NH_2_ (**3c**), Figure S48: ESI-CID-MS/MS spectra of TPP^+^-CH_2_CO-Asp-Gly-Arg-Thr-Leu-NH_2_ (**3c**). Precursor ion at *m/z* 454.236, Figure S49: ESI-MS spectrum of TPP^+^-CH_2_CO-Ala-Gly-Arg-Thr-Leu-NH_2_ (**4c**), Figure S50: ESI-CID-MS/MS spectra of TPP^+^-CH_2_CO-Ala-Gly-Arg-Thr-Leu-NH_2_ (**4c**). Precursor ion at *m/z* 432.236, Figure S51: ESI-MS spectrum of TPP^+^-CH_2_CO-Asp-Gly-Lys-Thr-Leu-NH_2_ (**5c**), Figure S52. ESI-CID-MS/MS spectra of TPP^+^-CH_2_CO-Asp-Gly-Lys-Thr-Leu-NH_2_ (**5c**). Precursor ion at *m/z* 440.221, Figure S53: ESI-MS spectrum of TPP^+^-CH_2_CO-Asp-Gly-Ala-Thr-Leu-NH_2_ (**6c**), Figure S54: ESI-CID-MS/MS spectra of TPP^+^-CH_2_CO-Asp-Gly-Ala-Thr-Leu-NH_2_ (**6c**). Precursor ion at *m/z* 822.357, Figure S55: ESI-MS spectrum of TPP^+^-CH_2_CO-Asp-Gly-Arg-Ala-Leu-NH_2_ (**7c**), Figure S56: ESI-CID-MS/MS spectra of TPP^+^-CH_2_CO-Asp-Gly-Arg-Ala-Leu-NH_2_ (**7c**). Precursor ion at *m/z* 439.220, Figure S57: ESI-MS spectrum of TMPP^+^-CH_2_CO-Ala-Ala-Ala-Ala-Ala-NH_2_ (**1d**), Figure S58: ESI-CID-MS/MS spectra of TMPP^+^-CH_2_CO-Ala-Ala-Ala-Ala-Ala-NH_2_ (**1d**). Precursor ion at *m/z* 945.395, Figure S59: ESI-MS spectrum of TMPP^+^-CH_2_CO-Ala-Ala-Pro-Ala-Ala-NH_2_ (**2d**), Figure S60: ESI-CID-MS/MS spectra of TMPP^+^-CH_2_CO-Ala-Ala-Pro-Ala-Ala-NH_2_ (**2d**). Precursor ion at *m/z* 971.405, Figure S61: ESI-MS spectrum of TMPP^+^-CH_2_CO-Asp-Gly-Arg-Thr-Leu-NH_2_ (**3d**), Figure S62: ESI-CID-MS/MS spectra of TMPP^+^-CH_2_CO-Asp-Gly-Arg-Thr-Leu-NH_2_ (**3d**). Precursor ion at *m/z* 566.752, Figure S63: ESI-MS spectrum of TMPP^+^-CH_2_CO-Ala-Gly-Arg-Thr-Leu-NH_2_ (**4d**), Figure S64: ESI-CID-MS/MS spectra of TMPP^+^-CH_2_CO-Ala-Gly-Arg-Thr-Leu-NH_2_ (**4d**). Precursor ion at *m/z* 544.765, Figure S65: ESI-MS spectrum of TMPP^+^-CH_2_CO-Asp-Gly-Lys-Thr-Leu-NH_2_ (**5d**), Figure S66: ESI-CID-MS/MS spectra of TMPP^+^-CH_2_CO-Asp-Gly-Lys-Thr-Leu-NH_2_ (**5d**). Precursor ion at *m/z* 552.758, Figure S67: ESI-MS spectrum of TMPP^+^-CH_2_CO-Asp-Gly-Ala-Thr-Leu-NH_2_ (**6d**), Figure S68: ESI-CID-MS/MS spectra of TMPP^+^-CH_2_CO-Asp-Gly-Ala-Thr-Leu-NH_2_ (**6d**). Precursor ion at *m/z* 1047.438, Figure S69: ESI-MS spectrum of TMPP^+^-CH_2_CO-Asp-Gly-Arg-Ala-Leu-NH_2_ (**7d**), Figure S70: ESI-CID-MS/MS spectra of TMPP^+^-CH_2_CO-Asp-Gly-Arg-Ala-Leu-NH_2_ (**7d**). Precursor ion at *m/z* 551.756, Figure S71: ESI-ECD-MS/MS spectrum of TEA^+^-CH_2_CO-Asp-Gly-Arg-Thr-Leu-NH_2_ (**3a**), Figure S72: ESI-ECD-MS/MS spectrum of TEA^+^-CH_2_CO-Ala-Gly-Arg-Thr-Leu-NH_2_(**4a**), Figure S73: ESI-ECD-MS/MS spectrum of TEA^+^-CH_2_CO-Asp-Gly-Lys-Thr-Leu-NH_2_ (**5a**). Precursor ion at *m/z* 337.229, Figure S74: ESI-ECD-MS/MS spectrum of TEA^+^-CH_2_CO-Asp-Gly-Arg-Ala-Leu-NH_2_ (**7b**), Figure S75: ESI-ECD-MS/MS spectrum of ABCO^+^-CH_2_CO-Asp-Gly-Arg-Thr-Leu-NH_2_ (**3b**). Precursor ion at *m/z* 356.221, Figure S76: ESI-ECD-MS/MS spectrum of ABCO^+^-CH_2_CO-Ala-Gly-Arg-Thr-Leu-NH_2_ (**4b**), Figure S77: ESI-ECD-MS/MS spectrum of ABCO^+^-CH_2_CO-Asp-Gly-Lys-Thr-Leu-NH_2_ (**5b**). Precursor ion at *m/z* 342.218, Figure S78: ESI-ECD-MS/MS spectrum of ABCO^+^-CH_2_CO-Asp-Gly-Arg-Ala-Leu-NH_2_ (**7b**), Figure S79: ESI-ECD-MS/MS spectrum of TPP^+^-CH_2_CO-Asp-Gly-Arg-Thr-Leu-NH_2_ (**3d**). Precursor ion at *m/z* 454.236, Figure S80: ESI-ECD-MS/MS spectrum of TPP^+^-CH_2_CO-Ala-Gly-Arg-Thr-Leu-NH_2_ (**4c**). Precursor ion at *m/z* 432.236, Figure S81: ESI-ECD-MS/MS spectrum of TPP^+^-CH_2_CO-Asp-Gly-Lys-Thr-Leu-NH_2_ (**5c**). Precursor ion at *m/z* 440.228, Figure S82: ESI-ECD-MS/MS spectrum of TPP^+^-CH_2_CO-Asp-Gly-Arg-Ala-Leu-NH_2_ (**7c**). Precursor ion at *m/z* 439.220, Figure S83: ESI-ECD-MS/MS spectrum of TMPP^+^-CH_2_CO-Asp-Gly-Arg-Thr-Leu-NH_2_ (**3d**). Precursor ion at *m/z* 566.752, Figure S84: ESI-ECD-MS/MS spectrum of TMPP^+^-CH_2_CO-Ala-Gly-Arg-Thr-Leu-NH_2_ (**4d**). Precursor ion at *m/z* 544.765, Figure S85: ESI-ECD-MS/MS spectrum of TMPP^+^-CH_2_CO-Asp-Gly-Lys-Thr-Leu-NH_2_ (**5d**). Precursor ion at *m/z* 552.758, Figure S86: ESI-ECD-MS/MS spectrum of TMPP^+^-CH_2_CO-Asp-Gly-Arg-Ala-Leu-NH_2_ (**7d**). Precursor ion at *m/z* 551.754.

## Sample Availability

Samples of the analyzed compounds are available from the authors.
