# Supplementary material for: Analysis of Fragmentation Pathways of Peptide Modified with Quaternary Ammonium and Phosphonium Group as Ionization Enhancers
Source: Molecules. 2021 Nov 18;26(22):6964. doi: 10.3390/molecules26226964 (PMC8623324; doi:10.3390/molecules26226964)
Supplement: Supplementary file 1 [file molecules-26-06964-s001.zip › molecules-1460615-supplementary.pdf]

molecules

Article

Analysis of fragmentation pathways of peptide modified with quaternary ammonium and phosphonium group as ionization enhancers

Monika Kijewska *, Dorota Gąszczyk, Remigiusz Bąchor *, Piotr Stefanowicz and Zbigniew Szewczuk

Faculty of Chemistry, University of Wroclaw, F. Joliot-Curie 14, 50-383 Wroclaw, Poland
*Correspondence: monika.kijewska@chem.uni.wroc.pl (M.K.);
remigiusz.bachor@chem.uni.wroc.pl(R.B.);
Tel.: +48 71 375 7250; +48 71 375 7218; Fax: +48 71 328 2348

Table of content

1. Experimental section ... 3
1.1 Reagents ... 3
1.2 Analytical data for QAS and QPS peptide conjugates ... 3
1.2.1 H-Gly-Ala-Ala-Ala-Ala-Ala-NH_2 (1) ... 4
1.2.2 H-Gly-Ala-Ala-Pro-Ala-Ala-NH_2 (2) ... 5
1.2.3 H-Gly-Asp-Gly-Arg-Thr-Leu-NH_2 (3) ... 5
1.2.4 H-Gly-Ala-Gly-Arg-Thr-Leu-NH_2 (4) ... 6
1.2.5 H-Gly-Asp-Gly-Lys-Thr-Leu-NH_2 (5) ... 7
1.2.6 H-Gly-Asp-Gly-Ala-Thr-Leu-NH_2 (6) ... 7
1.2.7 H-Gly-Asp-Gly-Arg-Ala-Leu-NH_2 (7) ... 8
1.2.8 TEA^+-CH_2CO-Ala-Ala-Ala-Ala-Ala-NH_2 (1a) ... 8
1.2.9 TEA^+-CH_2CO-Ala-Ala-Pro-Ala-Ala-NH_2 (2a) ... 9
1.2.10 TEA^+-CH_2CO-Asp-Gly-Arg-Thr-Leu-NH_2 (3a) ... 9
1.2.11 TEA^+-CH_2CO-Ala-Gly-Arg-Thr-Leu-NH_2 (4a) ... 10
1.2.12 TEA^+-CH_2CO-Asp-Gly-Lys-Thr-Leu-NH_2 (5a) ... 10
1.2.13 TEA^+-CH_2CO-Asp-Gly-Ala-Thr-Leu-NH_2 (6a) ... 11
1.2.14 TEA^+-CH_2CO-Asp-Gly-Arg-Ala-Leu-NH_2 (7a) ... 11
1.2.15 $ABCO^+$-CH_2CO-Ala-Ala-Ala-Ala-Ala-NH_2 (1b) ... 12
1.2.16 $ABCO^+$-CH_2CO-Ala-Ala-Pro-Ala-Ala-NH_2 (2b) ... 12
1.2.17 ABCO $^+$-CH_2CO-Asp-Gly-Arg-Thr-Leu-NH_2 (3b) ... 13
1.2.18 $ABCO^+$-CH_2CO-Ala-Gly-Arg-Thr-Leu-NH_2 (4b) ... 13

1.2.19 $ABCO^+$-CH_2CO-Asp-Gly-Lys-Thr-Leu-NH_2 (5b) ... 14

1.2.20 $ABCO^+$-CH_2CO-Asp-Gly-Ala-Thr-Leu-NH_2 (6b) ... 14

1.2.21 $ABCO^+$-CH_2CO-Asp-Gly-Arg-Ala-Leu-NH_2 (7b) ... 15

1.2.22 TPP^+-CH_2CO-Ala-Ala-Ala-Ala-Ala-NH_2 (1c) ... 15

1.2.23 TPP^+-CH_2CO-Ala-Ala-Pro-Ala-Ala-NH_2 (2c) ... 16

1.2.24 TPP^+-CH_2CO-Asp-Gly-Arg-Thr-Leu-NH_2 (3c) ... 16

1.2.25 TPP^+-CH_2CO-Ala-Gly-Arg-Thr-Leu-NH_2 (4c) ... 17

1.2.26 TPP^+-CH_2CO-Asp-Gly-Lys-Thr-Leu-NH_2 (5c) ... 17

1.2.27 TPP^+-CH_2CO-Asp-Gly-Ala-Thr-Leu-NH_2 (6c) ... 18

1.2.28 TPP^+-CH_2CO-Asp-Gly-Arg-Ala-Leu-NH_2 (7c) ... 18

1.2.29 $TMPP^+$-CH_2CO-Ala-Ala-Ala-Ala-Ala-NH_2 (1d) ... 19

1.2.30 $TMPP^+$-CH_2CO-Ala-Ala-Pro-Ala-Ala-NH_2 (2d) ... 19

1.2.31 $TMPP^+$-CH_2CO-Asp-Gly-Arg-Thr-Leu-NH_2 (3d) ... 20

1.2.32 $TMPP^+$-CH_2CO-Ala-Gly-Arg-Thr-Leu-NH_2 (4d) ... 21

1.2.33 $TMPP^+$-CH_2CO-Asp-Gly-Lys-Thr-Leu-NH_2 (5d) ... 22

1.2.34 $TMPP^+$-CH_2CO-Asp-Gly-Ala-Thr-Leu-NH_2 (6d) ... 23

1.2.35 $TMPP^+$-CH_2CO-Asp-Gly-Arg-Ala-Leu-NH_2 (7d) ... 24

2. ESI-MS and ESI-MS/MS spectra ... 25

2.1 ESI-MS and ESI-CID-MS/MS spectra ... 25

2.2 ESI-ECD-MS/MS spectra ... 81

1. Experimental section

1.1 Reagents

The derivatives of amino acids for peptide synthesis and the coupling reagent O-(Benzotriazol-1-yl)-*N,N,N′,N′*-tetramethyluronium tetrafluoroborate (TBTU) were purchased from NovaBiochem. The Rink Amide Resin (0.68 mmol/g), *N,N,N*-triethylamine (TEA), 1-azabicyclo [2.2.2] octane (ABCO), 2,4,6-triphenylpyridinium tetrafluoroborate (TPP), tris(2,4,6-trimethoxyphenyl) phosphine (TMPP)) and iodoacetic acid were purchased from Sigma-Aldrich. The solvents for peptide synthesis (analytical grade) were obtained from Riedel de Haën (DMF) and J. T. Baker (methanol, acetonitrile). Other solvents used in this work were obtained from Aldrich. Other reagents used in this work were obtained from Aldrich: triisopropylsilane (TIS) and IrisBiotech: trifluoroacetic acid, *N,N*-diisopropylethylamine (DIPEA).

1.2 Analytical data for QAS and QPS peptide conjugates

Table S1. Analytical data for model peptides

Nr.	Peptide sequence	$[M+H]^+$ found	$[M+H]^+$ calc.	$[M+2H]^{2+}$ found	$[M+2H]^{2+}$ calc.
1	H-GAAAAA-NH_2	430.242	430.241	-	-
2	H-GAAPAA-NH_2	456.257	456.257	-	-
3	H-GDGRTL-NH_2	617.341	617.337	309.181	309.172
4	H-GAGRTL-NH_2	573.340	573.347	287.176	287.177
5	H-GDGKTL-NH_2	589.336	589.330	295.181	295.169
6	H-GDGATL-NH_2	532.284	532.273	-	-
7	H-GDGRAL-NH_2	587.327	587.326	-	-

Table S2. Analytical data for QAS and QPS peptide conjugates

Nr.	Sequences of QAS and QPS peptides	$[M]^+$ found	$[M]^+$ calc.	$[M+H]^{2+}$ found	$[M+H]^{2+}$ calc.
1a	TEA^+-CH_2CO-AAAAA-NH_2	514.343	514.335	-	-
1b	$ABCO^+$-CH_2CO-AAAAA-NH_2	524.320	524.319	-	-
1c	TPP^+-CH_2CO-AAAAA-NH_2	720.344	720.350	-	-
1d	$TMPP^+$-CH_2CO-AAAAA-NH_2	945.395	945.401	473.205	473.204
2a	TEA^+-CH_2CO-AAPAA-NH_2	540.361	540.350	-	-
2b	$ABCO^+$-CH_2CO-AAPAA-NH_2	550.337	550.335	-	-
2c	TPP^+-CH_2CO-AAPAA-NH_2	746.360	746.366	-	-

2d	TMPP⁺-CH₂CO-AAPAA-NH₂	971.405	971.416	486.213	
3a	TEA^+-CH_2CO-DGRTL-NH_2	701.446	701.431	351.234	351.219
3b	$ABCO^+$-CH_2CO-DGRTL-NH_2	711.416	711.415	356.221	356.211
3c	TPP^+-CH_2CO-DGRTL-NH_2	907.439	907.446	454.236	454.227
3d	$TMPP^+$-CH_2CO-DGRTL-NH_2	1132.483	1132.494	566.752	566.752
4a	TEA^+-CH_2CO-AGRTL-NH_2	657.458	657.441	329.237	329.224
4b	$ABCO^+$-CH_2CO-AGRTL-NH_2	667.425	667.425	334.224	334.216
4c	TPP^+-CH_2CO-AGRTL-NH_2	863.443	863.456	432.236	432.232
4d	$TMPP^+$-CH_2CO-AGRTL-NH_2	1088.509	1088.506	544.765	544.757
5a	TEA^+-CH_2CO-DGKTL-NH_2	673.439	673.424	337.229	337.216
5b	$ABCO^+$-CH_2CO-DGKTL-NH_2	683.418	683.409	342.218	342.208
5c	TPP^+-CH_2CO-DGKTL-NH_2	879.425	879.440	440.224	440.224
5d	$TMPP^+$-CH_2CO-DGKTL-NH_2	1104.509	1104.490	552.758	552.749
6a	TEA^+-CH_2CO-DGATL-NH_2	616.361	616.366	-	-
6b	$ABCO^+$-CH_2CO-DGATL-NH_2	626.355	626.350	-	-
6c	TPP^+-CH_2CO-DGATL-NH_2	822.361	822.382	-	-
6d	$TMPP^+$-CH_2CO-DGATL-NH_2	1047.438	1047.440	-	-
7a	TEA^+-CH_2CO-DGRAL-NH_2	671.409	671.420	336.220	336.214
7b	$ABCO^+$-CH_2CO-DGRAL-NH_2	681.411	681.404	341.216	341.206
7c	TPP^+-CH_2CO-DGRAL-NH_2	877.421	877.436	439.220	439.221
7d	$TMPP^+$-CH_2CO-DGRAL-NH_2	1102.505	1102.486	551.754	551.745

1.2.1 H-Gly-Ala-Ala-Ala-Ala-Ala-NH_2 (1)

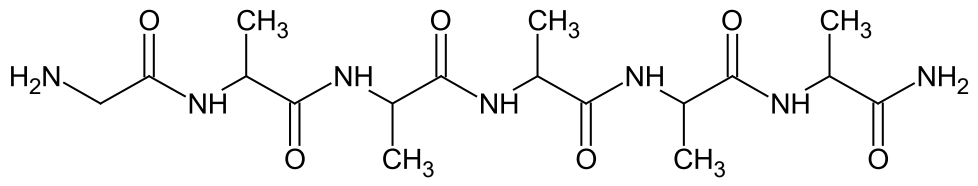

Analytical data:

ESI-MS: *m/z* $[M+H]^+$: 430.242 (calc. for $C_{17}H_{32}N_7O_6$ $[M+H]^+$: 430.241) (**SI Figure S1**)

ESI-MS/MS: precursor ion at *m/z* $[M+H]^+$: 430.242, collision energy 10 eV: 314.178 (calc. for a_5: 314.182); 243.139 (calc. for dla a_4: 243.145); 200.098 (calc. for b_3: 200.103); 271.135 (calc. for b_4: 271.140); 342.176 (calc. for b_5: 342,177) (**SI Figure S2**)

1.2.2 H-Gly-Ala-Ala-Pro-Ala-Ala-NH_2 (2)

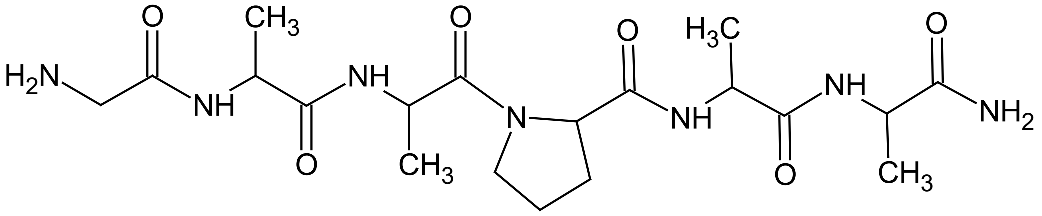

Analytical dada:

ESI-MS: *m/z* $[M+H]^+$: 456.257 (calc. for $C_{19}H_{34}N_7O_6$ $[M+H]^+$: 456.257) (**SI Figure S3**)

ESI-MS/MS: precursor ion at *m/z* $[M+H]^+$: 456.257, collision energy 12 eV: 340.208 (calc. for a_5: 340.198); 271.147 (calc. for a_4: 271.177); 200.107 (calc. for b_3: 200.103); 368.193 (calc. for b_5: 368.192); 257.162 (calc. for y_3: 257,161); 328.199 (calc. for y_4: 328.198) (**SI Figure S4**)

1.2.3 H-Gly-Asp-Gly-Arg-Thr-Leu-NH_2 (3)

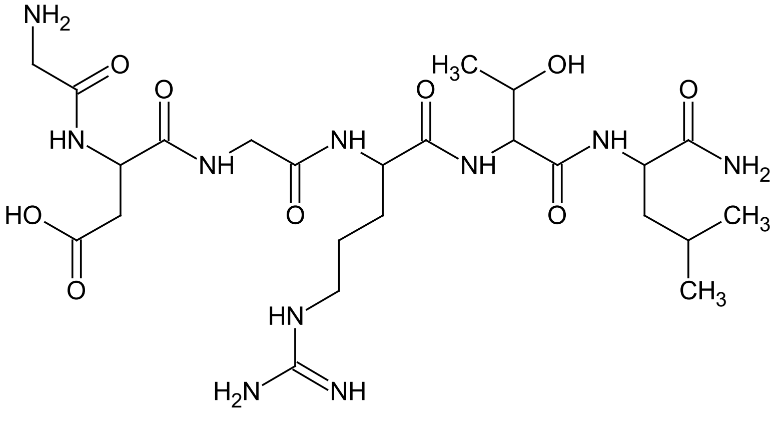

Analytical data:

ESI-MS: *m/z* $[M+H]^+$: 617.341 (calc. for $C_{24}H_{45}N_{10}O_9$ $[M+H]^+$: 617.337); *m/z* $[M+2H]^{2+}$: 309.181 (calc. for $C_{24}H_{46}N_{10}O_9$ $[M+2H]^{2+}$: 309.172) (**SI Figure S5**)

ESI-MS/MS: precursor ion at *m/z* $[M+2H]^{2+}$: 309.181, collision energy 10 eV: 145.062 (calc. for a_2: 145.061) 459.228 (calc. for a_5: 459.231); 386,178 (calc. for b_4: 386.383); 487.225 (calc. for b_5: 487.226); 173.054 (calc. for b_2: 173.146); 445,283 (calc. for y_4: 445.288) (**SI Figure S6**)

1.2.4 H-Gly-Ala-Gly-Arg-Thr-Leu-NH_2 (4)

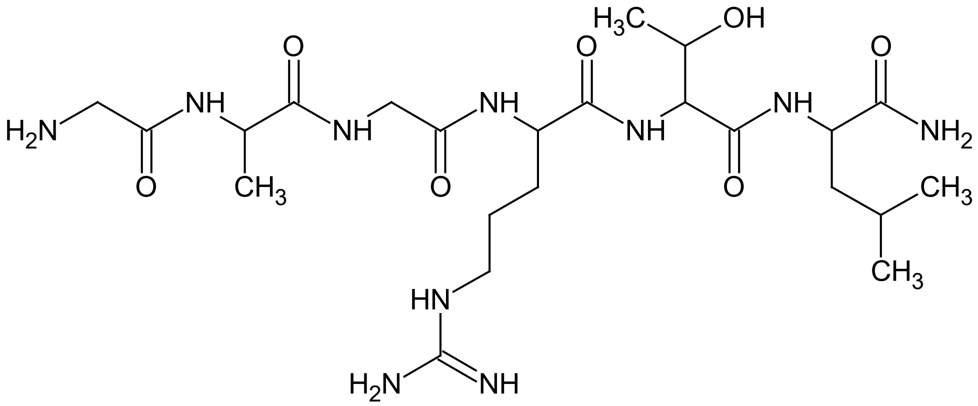

Analytical data:

ESI-MS: *m/z* $[M+H]^+$: 573.340 (calc. for $C_{23}H_{45}N_{10}O_7$ $[M+H]^+$: 573.347); *m/z* $[M+2H]^{2+}$: 287.176 (calc. for $C_{23}H_{46}N_{10}O_7$ $[M+2H]^{2+}$: 287.177) (**SI Figure S7**)

ESI-MS/MS: precursor ion at *m/z* $[M+H]^+$: 573.340, collision energy 30 eV: 314.195 (calc. for a_4: 314.194); 415.250 (calc. for a_5: 415.241); 342.202 (calc. for b_4: 342.188); 443.241 (calc. for b_5: 443.236) (**SI Figure S8**)

1.2.5 H-Gly-Asp-Gly-Lys-Thr-Leu-NH_2 (5)

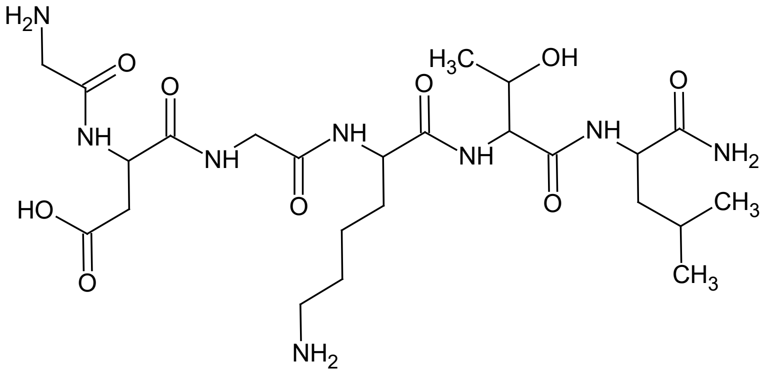

Analytical data:

ESI-MS: *m/z* $[M+H]^+$: 589.336 (calc. for $C_{24}H_{45}N_8O_9$ $[M+H]^+$: 589.330); *m/z* $[M+2H]^{2+}$: 295.181 (calc. for $C_{24}H_{46}N_8O_9$ $[M+2H]^{2+}$: 295.169) (**SI Figure S9**)

ESI-MS/MS: precursor ion at *m/z* $[M+H]^+$: 589.336, collision energy 20 eV: 358.181 (calc. for b_4: 358.172); 459.229 (calc. for b_5: 459.420); 417.288 (calc. for y_4: 417.282) (**SI Figure S10**)

1.2.6 H-Gly-Asp-Gly-Ala-Thr-Leu-NH_2 (6)

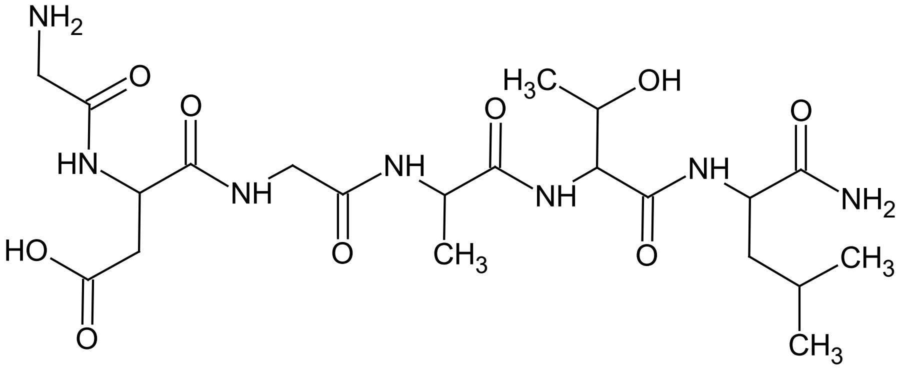

Analytical data:

ESI-MS: *m/z* $[M+H]^+$: 532.284 (calc. for $C_{21}H_{38}N_7O_9$ $[M+H]^+$: 532.273) (**SI Figure S11**)

ESI-MS/MS: precursor ion at *m/z* $[M+H]^+$: 532.284, collision energy 10 eV: 402.170 (calc. for b_5: 402.162); 515.255 (calc. for $[M-NH_3]^+$: 515.246); 497.245 (calc. for $[M-NH_3-H_2O]^+$: 497.235); 487.261 (calc. for $[M-NH_3-CO]^+$: 487.254) (**SI Figure S12**)

1.2.7 H-Gly-Asp-Gly-Arg-Ala-Leu-NH_2 (7)

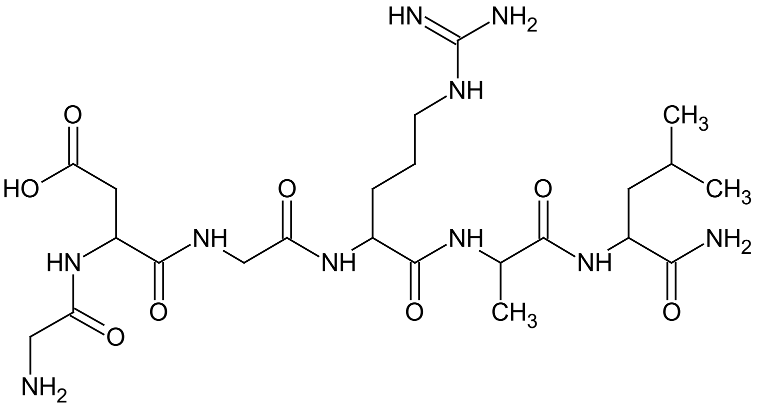

Analytical data:

ESI-MS: *m/z* $[M+H]^+$: 587.327 (calc. for $C_{23}H_{43}N_{10}O_8$ $[M+H]^+$: 587,326) (**SI Figure S13**)

ESI-MS/MS: precursor ion at *m/z* $[M+H]^+$: 587.327, collision energy 25 eV: 386,189 (calc. for b_4: 386.178); 457.222 (calc. for b_5: 457.215); 415.282 (calc. for y_4: 415.278) (**SI Figure S14**)

1.2.8 TEA^+-CH_2CO-Ala-Ala-Ala-Ala-Ala-NH_2 (1a)

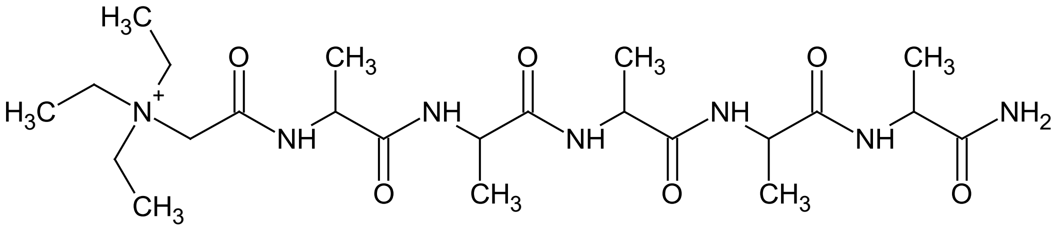

Analytical data:

ESI-MS: *m/z* $[M]^+$: 514.343; calc. for $C_{23}H_{44}N_7O_6$: $[M]^+$: 514.335 (**SI Figure S15**)

ESI-MS/MS: precursor ion at *m/z* $[M]^+$: 514.343, collision energy 25 eV: 185.161 (calc. for a_2: 185.165); 256.180 (calc. for a_3: 256.202); 327.203 (calc. for a_4: 327.239); 398.241 (calc. for a_5: 398.276); 213.157 (calc. for b_2: 213.160) (**SI Figure S16**)

1.2.9 TEA+-CH2CO-Ala-Ala-Pro-Ala-Ala-NH2 (2a)

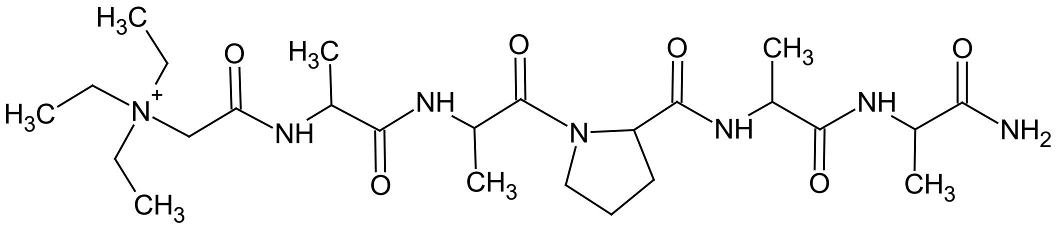

Analytical data:

ESI-MS: *m/z* [M]+: 540,361; calc. for $C_{25}H_{46}N_7O_6$: [M]+: 540.350 (**SI Figure S17**)

ESI-MS/MS: precursor ion at *m/z* [M]+: 540,361, collision energy 25 eV: 185.160 (calc. for a_2: 185.165); 256.191 (calc. for a_3: 256.202); 213.156 (calc. for b_2: 213.160); 284.195 (calc. for b_3: 284.197); 143.121 (calc. for z_2: 143.082) (**SI Figure S18**)

1.2.10 TEA+-CH2CO-Asp-Gly-Arg-Thr-Leu-NH2 (3a)

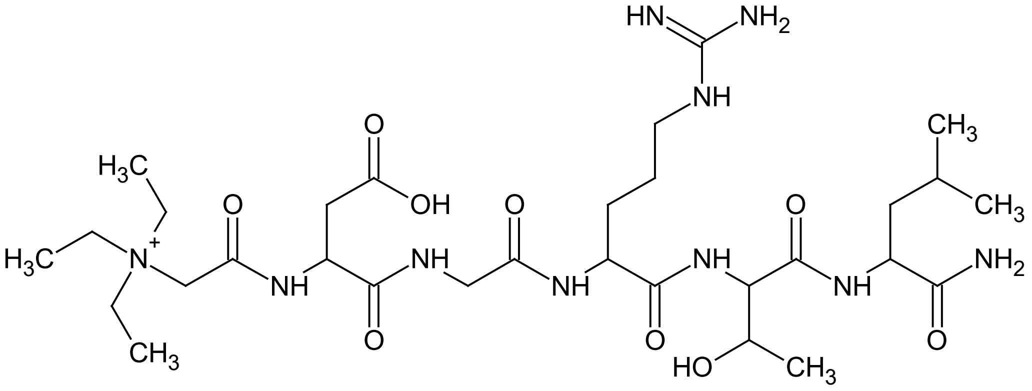

Analytical data:

ESI-MS: *m/z* [M]+: 701.446; calc. for $C_{30}H_{57}N_{10}O_9$: [M]+: 701.431, *m/z* [M+H]2+: 351.234; calc. for $C_{30}H_{58}N_{10}O_9$: [M+H]2+: 351.219 (**SI Figure S19**)

ESI-MS/MS: precursor ion at *m/z* [M+H]2+: 351.234, collision energy 17 eV: 229.150 (calc. for a_2: 229.155); 221.642 (calc. for a_4^{2+}: 221.642); 272.170 (calc. for a_5^{2+}: 272.166); 257.147 (calc. for b_2: 257.150); 314,171 (calc. for b_3: 314.171); 235.642 (calc. for b_4^{2+}: 235.640); 286.169 (calc. for b_5^{2+}: 286.164); 331.193 (calc. for c_3: 331.198); 244.646 (calc. for c_4^{2+}: 244.153) (**SI Figure S20**)

1.2.11 TEA$^+$-CH$_2$CO-Ala-Gly-Arg-Thr-Leu-NH$_2$ (4a)

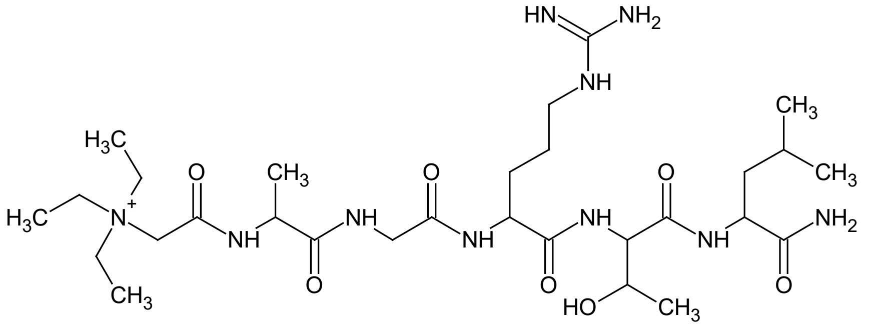

Analytical data:

ESI-MS: *m/z* [M]$^+$: 657,458; calc. for $C_{29}H_{57}N_{10}O_7$ [M]$^+$: 657.441, *m/z* [M+H]$^{2+}$: 329.237; calc. for $C_{29}H_{58}N_{10}O_7$ [M+H]$^{2+}$: 329.224 (**SI Figure S21**)

ESI-MS/MS: precursor ion at *m/z* [M+H]$^{2+}$: 329.237, collision energy 15 eV: 242.160 (calc. for a_3: 242.186); 199.647 (calc. for a_4^{2+}: 199.647); 250.174 (calc. for a_5^{2+}: 250.171); 270.178 (calc. for b_3: 270.181); 213.644 (calc. for b_4^{2+}: 213.645); 264.174 (calc. for b_5^{2+}: 264.169); 287.208 (calc. for c_3: 287.207); 222.651 (calc. for c_4^{2+}: 222.286); 445.286 (calc. for y_4: 445.288) (**SI Figure S22**)

1.2.12 TEA$^+$-CH$_2$CO-Asp-Gly-Lys-Thr-Leu-NH$_2$ (5a)

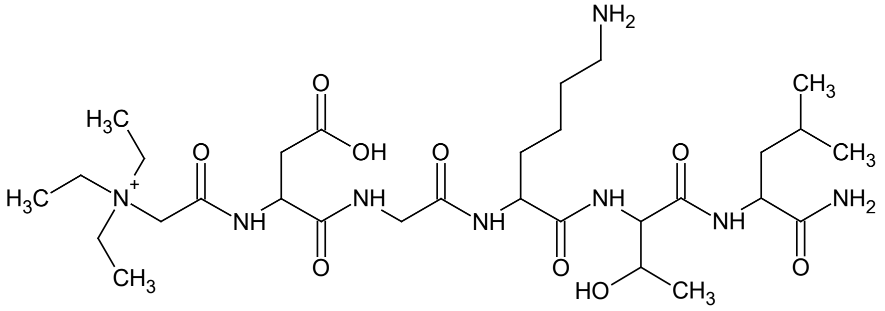

Analytical data:

ESI-MS: *m/z* [M]$^+$: 673.439; calc. for $C_{30}H_{57}N_8O_9$ [M]$^+$: 673.424, *m/z* [M+H]$^{2+}$: 337.229; calc. for $C_{30}H_{59}N_8O_9$ [M+H]$^{2+}$: 337.216 (**SI Figure S23**)

ESI-MS/MS: precursor ion at *m/z* [M+H]$^{2+}$: 337.229, collision energy 12 eV: 229.152 (calc. for a_2: 229.155); 515.309 (calc. for a_5: 515.318); 258.166 (calc. for a_5^{2+}: 258.163); 221.239 (calc. for b_4^{2+}: 221.637); 543.309 (calc. for b_5: 543.321); 272.168 (calc. for b_5^{2+}: 272.161) (**SI Figure S24**)

1.2.13 TEA$^+$-CH$_2$CO-Asp-Gly-Ala-Thr-Leu-NH$_2$ (6a)

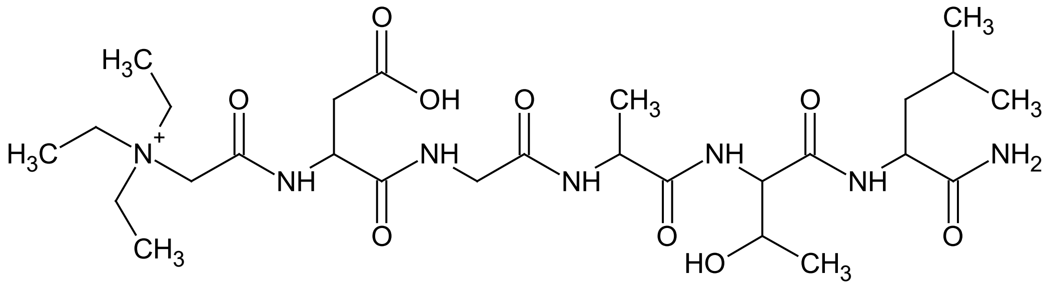

Analytical data:

ESI-MS: *m/z* [M]$^+$: 616,361; calc. for $C_{27}H_{50}N_7O_9$ [M]$^+$: 616.366 (**SI Figure S25**)

ESI-MS/MS: precursor ion at *m/z* [M]$^+$: 616.361, collision energy 30 eV: 229.152 (calc. for a_2: 229.155); 286.156 (calc. for a_3: 286.176); 357,179 (calc. for a_4: 357.213); 458.233 (calc. for a_5: 458.161); 257.148 (calc. for b_2: 257.148) 314,179 (calc. for b_3: 314.171) 385.179 (calc. for b_4: 385.208); 486.260 (calc. for b_5: 486.256) (**SI Figure S26**)

1.2.14 TEA$^+$-CH$_2$CO-Asp-Gly-Arg-Ala-Leu-NH$_2$ (7a)

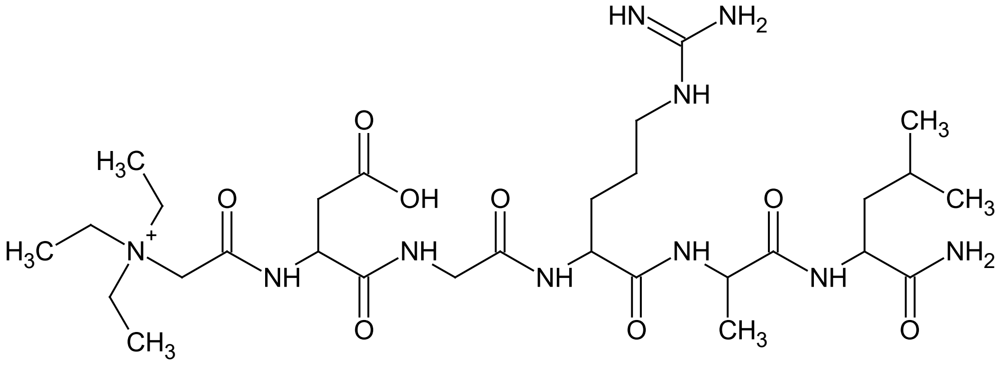

Analytical data:

ESI-MS: *m/z* [M]$^+$: 671.409; calc. for $C_{29}H_{55}N_{10}O_8$ [M]$^+$: 671.420, *m/z* [M+H]$^{2+}$: 336.220; calc. for $C_{29}H_{56}N_{10}O_8$ [M+H]$^{2+}$: 336.214 (**SI Figure S27**)

ESI-MS/MS: precursor ion at *m/z* [M+H]$^{2+}$: 336.220, collision energy 17 eV: 229.148 (calc. for a_2: 229.155); 286.173 (calc. for a_3: 286.176); 221.643 (calc. for a_4: 221.642); 257.303 (calc. for a_5^{2+}: 257.153); 314,170 (calc. for b_3: 314.171); 235.641 (calc. for b_4^{2+}: 235.640); 331.197 (calc. for c_3: 331.198) (**SI Figure S28**)

1.2.15 ABCO⁺-CH2CO-Ala-Ala-Ala-Ala-Ala-NH2 (1b)

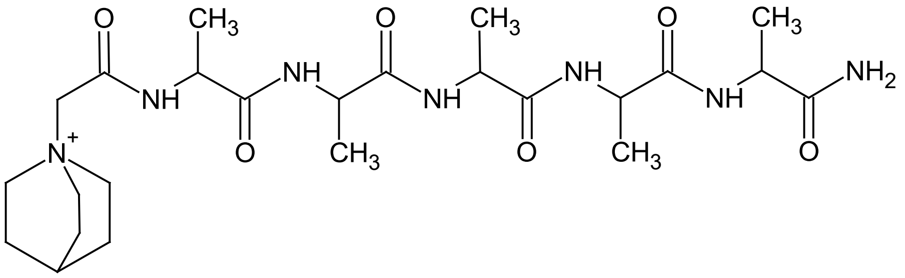

Analytical data:

ESI-MS: *m/z* $[M]^+$: 524.320; calc. for $C_{24}H_{42}N_7O_6$ $[M]^+$: 524.319 (**SI Figure S29**)

ESI-MS/MS: precursor ion at *m/z* $[M]^+$: 524.320, collision energy 37 eV: 266.179 (calc. for a_3: 266.186); 337.219 (calc. for a_4: 337.224); 408.255 (calc. for a_5: 408,261); 294.178 (calc. for b_3: 294.181); 365.208 (calc. for b_4: 365.218); 436.246 (calc. for b_5: 436.155) (**SI Figure S30**)

1.2.16 ABCO⁺-CH2CO-Ala-Ala-Pro-Ala-Ala-NH2 (2b)

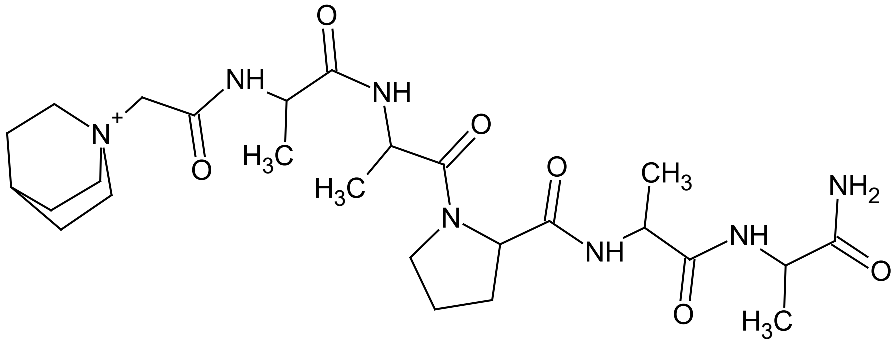

Analytical data:

ESI-MS: *m/z* $[M]^+$: 550.337; calc. for $C_{26}H_{44}N_7O_6$ $[M]^+$: 550.335 (**SI Figure S31**)

ESI-MS/MS: precursor ion at *m/z* $[M]^+$: 550.337, collision energy 40 eV: 266.177 (calc. for a_3: 266.186); 364.196 (calc. for a_4: 364.146); 434.259 (calc. for a_5: 434.276); 294.166 (calc. for b_3: 294.181); 391.259 (calc. for b_4: 391.2234); 462.269 (calc. for b_5: 462.271) (**SI Figure S32**)

1.2.17 ABCO $^{+}$-CH_2CO-Asp-Gly-Arg-Thr-Leu-NH_2 (3b)

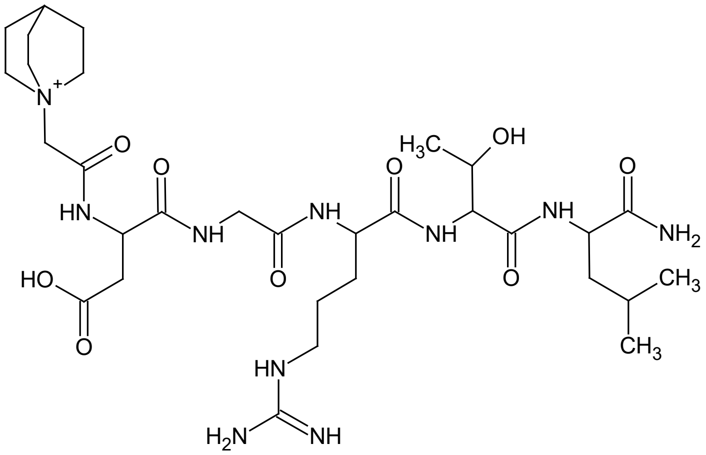

Analytical data:

ESI-MS: *m/z* $[M]^+$: 711.416; calc. for $C_{31}H_{55}N_{10}O_9$ $[M]^+$: 711.415, *m/z* $[M+H]^{2+}$: 356.221; calc. for $C_{31}H_{56}N_{10}O_9$ $[M+H]^{2+}$: 356.211 (**SI Figure S33**)

ESI-MS/MS: precursor ion at *m/z* $[M+H]^{2+}$: 356.221, collision energy 20 eV: 277.150 (calc. for a_5: 277.158); 324.139 (calc. for b_3: 324.155); 291.127 (calc. for dla b_5^{2+}: 291.127); 341.174 (calc. for c_3: 341.182); 445.285 (calc. for y_4: 445.288) (**SI Figure S34**)

1.2.18 ABCO^{+}-CH_2CO-Ala-Gly-Arg-Thr-Leu-NH_2 (4b)

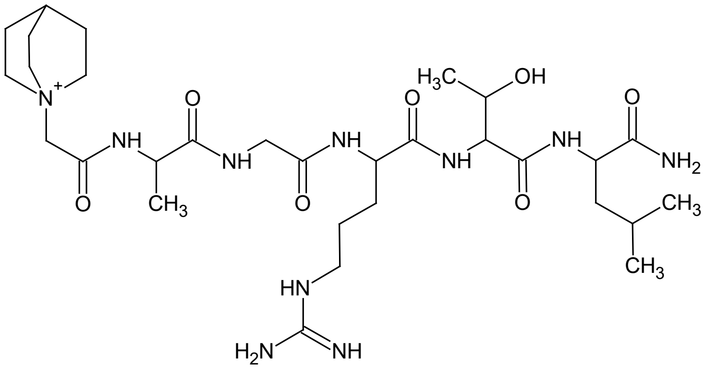

Analytical data:

ESI-MS: *m/z* $[M]^+$: 667.425; calc. for $C_{30}H_{55}N_{10}O_7$ $[M]^+$: 667.425; *m/z* $[M+H]^{2+}$: 334.224; calc. for $C_{30}H_{56}N_{10}O_7$ $[M+H]^{2+}$: 334.216 (**SI Figure S35**)

ESI-MS/MS: precursor ion at *m/z* $[M+H]^{2+}$: 334.224, collision energy 15 eV: 492.288 (calc. for a_5-NH_3: 492.293); 419.235 (calc. for y_3-NH_3: 419.241); 520.280 (calc. for b_5-NH_3: 520.288); 445.284 (calc. fory_4: 445.288) (**SI Figure S36**)

1.2.19 ABCO⁺-CH₂CO-Asp-Gly-Lys-Thr-Leu-NH₂ (5b)

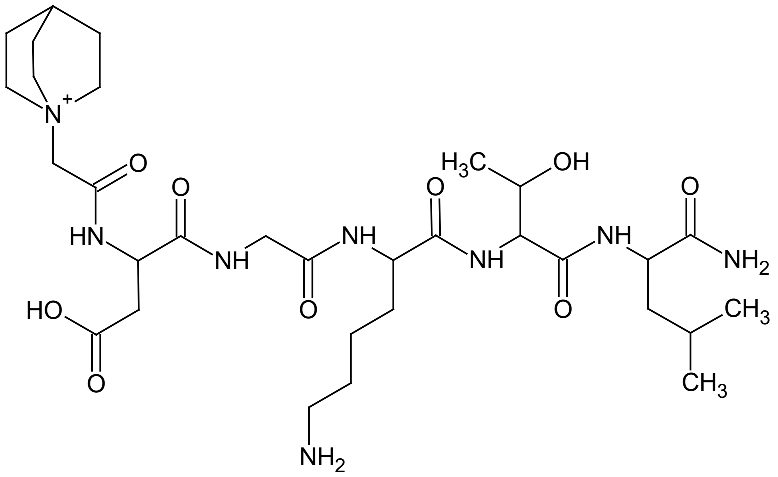

Analytical data:

ESI-MS: *m/z* $[M]^+$: 683.418; calc. for $C_{31}H_{55}N_8O_9$ $[M]^+$: 683.409; *m/z* $[M+H]^{2+}$: 342.218; calc. for $C_{31}H_{56}N_8O_9$ $[M+H]^{2+}$: 342.208 (**SI Figure S37**)

ESI-MS/MS: precursor ion at *m/z* $[M+H]^{2+}$: 342.218, collision energy 13 eV: 424.250 (calc. for a_4: 424.255); 525,299 (calc. for a_5: 525.303); 452.242 (calc. for b_4: 452.250); 553.295 (calc. for b_5: 553.298); 277.141 (calc. for b_5^{2+}: 277.153); 469.276 (calc. for c_4: 469.277) (**SI Figure S38**)

1.2.20 ABCO⁺-CH₂CO-Asp-Gly-Ala-Thr-Leu-NH₂ (6b)

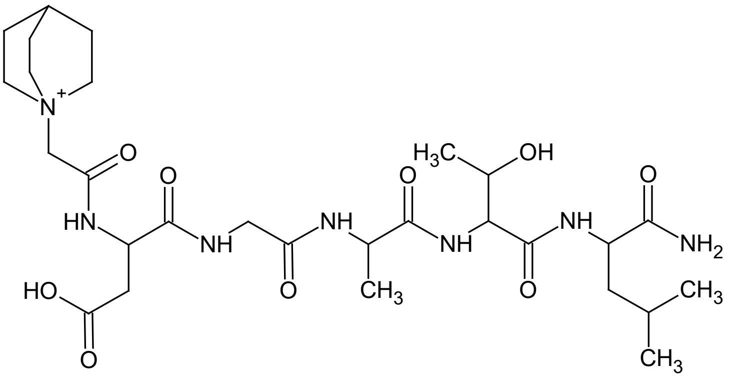

Analytical data:

ESI-MS: *m/z* $[M]^+$: 626.355; calc. for $C_{28}H_{48}N_7O_9$ $[M]^+$: 626.350 (**SI Figure S39**)

ESI-MS/MS: precursor ion at *m/z* $[M]^+$: 626.355, collision energy 40 eV: 296.153 (calc. for a_3: 296.160); 367.188 (calc. for a_4: 367.198); 468.250 (calc. for a_5: 468.245); 267.128 (calc. for b_2: 267.134); 324.150 (calc. for b_3: 324.155); 395.199 (calc. for b_4: 395.193); 496.236 (calc. for b_5: 496.240) (**SI Figure S40**)

1.2.21 ABCO+-CH2CO-Asp-Gly-Arg-Ala-Leu-NH2 (7b)

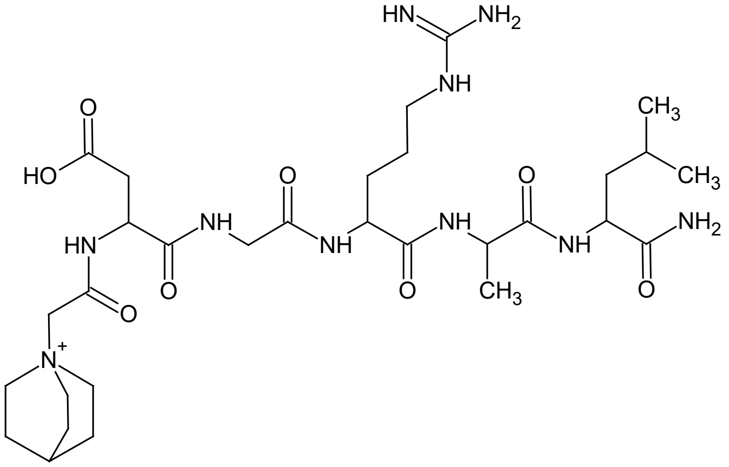

Analytical data:

ESI-MS: *m/z* $[M]^{+}$: 681.411; calc. for $C_{29}H_{55}N_{10}O_{8}$ $[M]^{+}$: 681.404; *m/z* $[M+H]^{2+}$: 341.216; calc. for $C_{31}H_{56}N_{8}O_{9}$ $[M+H]^{2+}$: 341.206 (**SI Figure S41**)

ESI-MS/MS: precursor ion at *m/z* $[M+H]^{2+}$: 341.216, collision energy 15 eV: 435.230 (calc. for a_4-NH_3: 435.236); 506.266 (calc. for a_5-NH_3: 506.273); 463,229 (calc. for b_4-NH_3: 463.231); 534.265 (calc. for b_5-NH_3: 534.268); 415.275 (calc. for y_4: 415.278) (**SI Figure S42**)

1.2.22 TPP+-CH2CO-Ala-Ala-Ala-Ala-Ala-NH2 (1c)

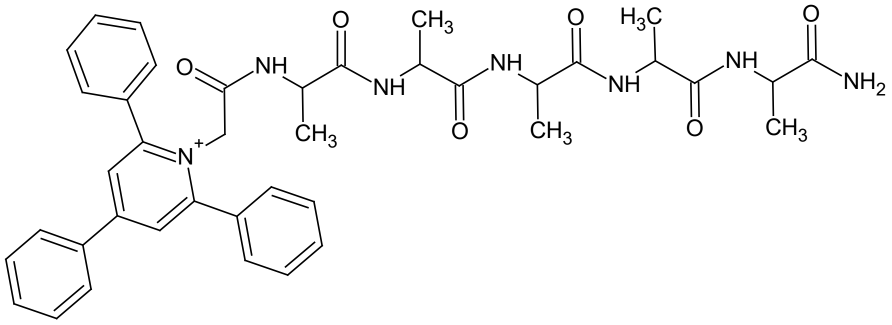

Analytical data:

ESI-MS: *m/z* $[M]^{+}$: 720.344; calc. for $C_{40}H_{46}N_{7}O_{6}$ $[M]^{+}$: 720.350 (**SI Figure S43**)

ESI-MS/MS: precursor ion at *m/z* $[M]^{+}$: 720.344, collision energy 30 eV: 391.170 (calc. for a_2: 391.181) 462.203 (calc. for a_3: 462.218); 533.242 (calc. for a_4: 533.255); 604.275 (calc. for a_5: 604.292); 419.164 (calc. for b_2: 419.175); 490.189 (calc. for b_3: 490.213); 561.203 (calc. for b_4: 561.250) (**SI Figure S44**)

1.2.23 TPP$^+$-CH$_2$CO-Ala-Ala-Pro-Ala-Ala-NH$_2$ (2c)

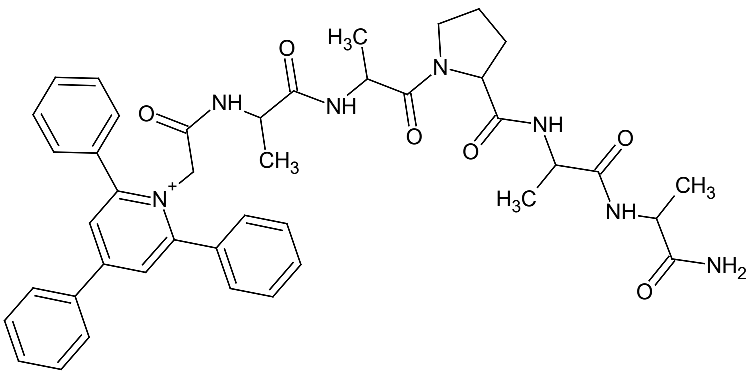

Analytical data:

ESI-MS: *m/z* [M]$^+$: 746.360; calc. for $C_{42}H_{46\backslash 8}N_7O_6$ [M]$^+$: 746.366 (**SI Figure S45**)

ESI-MS/MS: precursor ion at *m/z* [M]$^+$: 746.360, collision energy 15 eV: 391.170 (calc. for a_2: 391.181) 462.210 (calc. for a_3: 462.218); 419.166 (calc. for b_2: 419.175); 490.198 (calc. for b_3: 490.213) (**SI Figure S46**)

1.2.24 TPP$^+$-CH$_2$CO-Asp-Gly-Arg-Thr-Leu-NH$_2$ (3c)

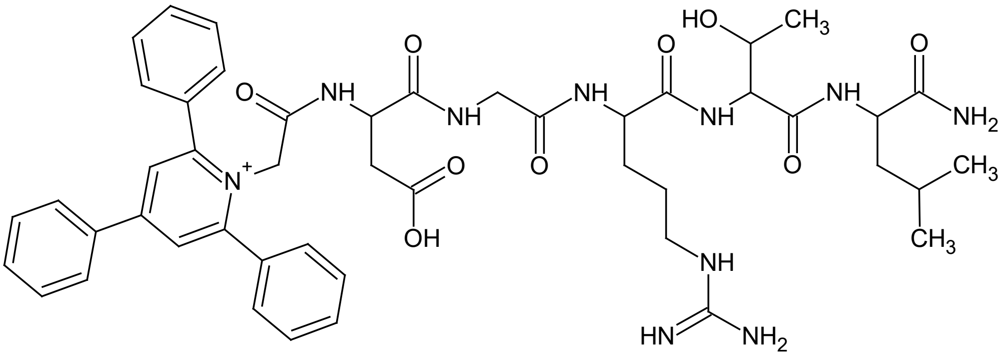

Analytical data:

ESI-MS: *m/z* [M]$^+$: 907.439; calc. for $C_{47}H_{59}N_{10}O_9$ [M]$^+$: 907.446; *m/z* [M+H]$^{2+}$: 454.236; calc. for $C_{47}H_{60}N_{10}O_9$ [M+H]$^{2+}$: 454.227 (**SI Figure S47**)

ESI-MS/MS: precursor ion at *m/z* [M+H]$^{2+}$: 454,236, collision energy 20 eV: 435.165 (calc. for a_2: 435.170); 492.181 (calc. for a_3: 492.192); 324.648 (calc. for a_4^{2+}: 324.650); 375,174 (calc. for a_5^{2+}: 375.174); 463.157 (calc. for b_2: 463.165); 520.179 (calc. for b_3: 520.187); 338.647 (calc. for b_4^{2+}: 338.648); 480.177 (calc. for c_2: 480.192); 537.198 (calc. for c_3: 537.213); 347.156 (calc. for c_4^{2+}: 347.161); 445.253 (calc. for y_4: 445.288) (**SI Figure S48**)

1.2.25 TPP$^+$-CH_2CO-Ala-Gly-Arg-Thr-Leu-NH_2 (4c)

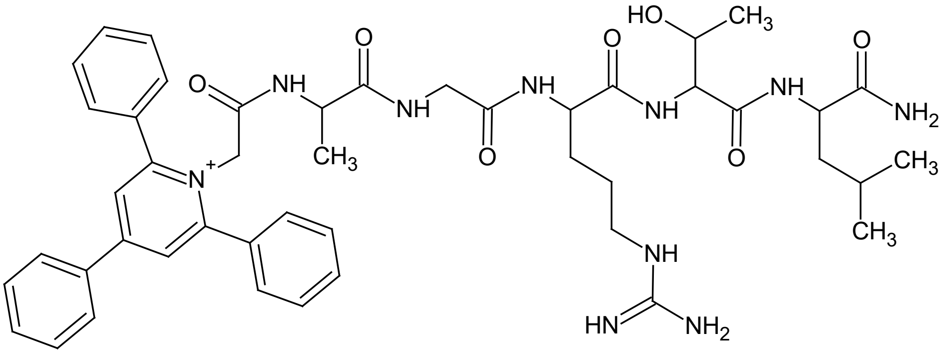

Analytical data:

ESI-MS: *m/z* $[M]^+$: 863.443; calc. for $C_{46}H_{59}N_{10}O_9$ $[M]^+$: 863.456; *m/z* $[M+H]^{2+}$: 432.236; calc. for $C_{46}H_{60}N_{10}O_9$ $[M+H]^{2+}$: 432.232 (**SI Figure S49**)

ESI-MS/MS: precursor ion at *m/z* $[M+H]^{2+}$: 432.236, collision energy 17 eV: 391.179 (calc. for a_2: 391.181); 448.191 (calc. for a_3: 448.202); 353.180 (calc. for a_5^{2+}: 353.179); 476.190 (calc. for b_3: 476.197); 316.650 (calc. for b_4^{2+}: 316.653); 367.176 (calc. for b_5^{2+}: 367.177) 493.208 (calc. for c_3: 493.223) (**SI Figure S50**)

1.2.26 TPP$^+$-CH_2CO-Asp-Gly-Lys-Thr-Leu-NH_2 (5c)

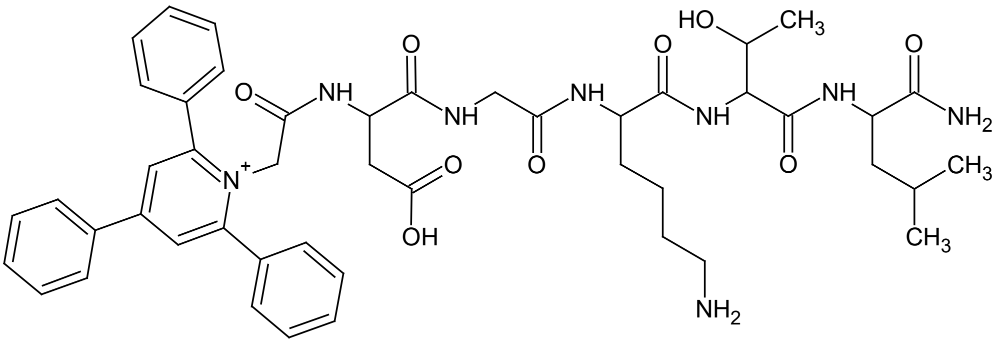

Analytical data:

ESI-MS: *m/z* $[M]^+$: 879.425; calc. for $C_{47}H_{59}N_8O_9$ $[M]^+$: 879.440; *m/z* $[M+H]^{2+}$: 440,224; calc. for $C_{47}H_{60}N_8O_9$ $[M+H]^{2+}$: 440.224 (**SI Figure S51**)

ESI-MS/MS: precursor ion at *m/z* $[M+H]^{2+}$: 440,224, collision energy 17 eV: 435.161 (calc. for a_2: 435.170); 492.182 (calc. for a_3: 492.192); 620.278 (calc. for a_4: 620.286); 361.169 (calc. for a_5^{2+}: 361.171); 721.315 (calc. for a_5: 721.334); 463.148 (calc. for a b_2: 463.165); 520.172 (calc. for b_3: 520.187); 324.639 (calc. for b_4^{2+}: 324.644); 648.265 (calc. for b_4: 648.282); 375.163 (calc. for b_5^{2+}: 375.168); 749.309 (calc. for b_5: 749.329); 537.193 (calc. for c_3: 537.213); 665.284 (calc. for c_4: 665.308) (**SI Figure S52**)

1.2.27 TPP+-CH2CO-Asp-Gly-Ala-Thr-Leu-NH2 (6c)

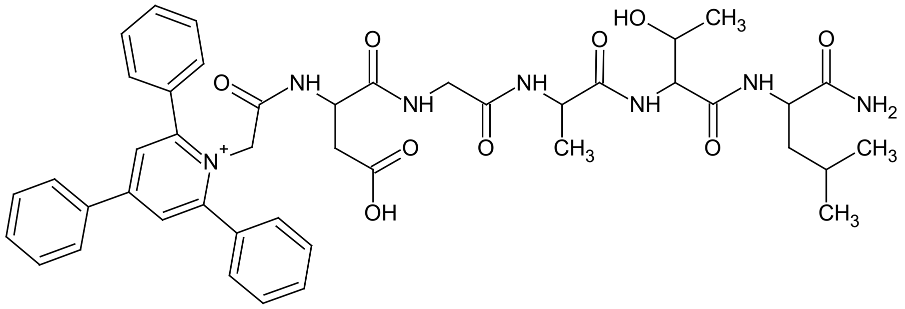

Analytical data:

ESI-MS: *m/z* $[M]^+$: 822,361; calc. for $C_{44}H_{52}N_7O_9$ $[M]^+$: 822.382 (**SI Figure S53**)

ESI-MS/MS: precursor ion at *m/z* $[M]^+$: 822.361, collision energy 35 eV: 435.155 (calc. for a_2: 435.170); 492.173 (calc. for a_3: 492.192); 563.203 (calc. for a_4: 563.229); 664.256 (calc. for a_5: 664.277); 463.147 (calc. for b_2: 463.165); 520.164 (calc. for b_3: 520.187); 591.198 (calc. for b_4: 591.224); 692.253 (calc. for b_5: 692.272) (**SI Figure S54**)

1.2.28 TPP+-CH2CO-Asp-Gly-Arg-Ala-Leu-NH2 (7c)

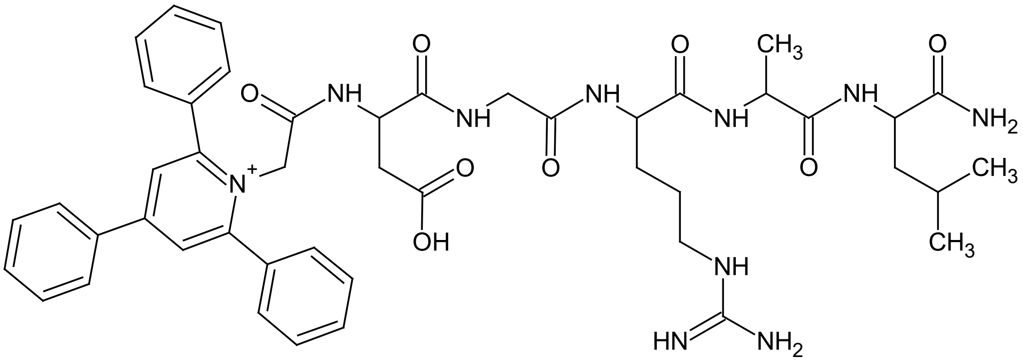

Analytical data:

ESI-MS: *m/z* $[M]^+$: 877.421; calc. for $C_{46}H_{57}N_{10}O_8$ $[M]^+$: 877.436; *m/z* $[M+H]^{2+}$: 439.220; calc. for $C_{46}H_{58}N_{10}O_{89}$ $[M+H]^{2+}$: 439.221(**SI Figure S55**)

ESI-MS/MS: precursor ion at *m/z* $[M+H]^{2+}$: 439.220, collision energy 35 eV: 435.168 (calc. for a_2: 435.170); 492,176 (calc. for a_3: 492.192); 324.635 (calc. for a_4^{2+}: 324.650); 360.161 (calc. for a_5: 760.169); 463.151 (calc. for b_2: 463.165); 520.173 (calc. for b_3: 520.187); 338.642 (calc. for b_4^{2+}: 338.648); 374.164 (calc. for b_5^{2+}: 374.166); 537.192 (calc. for c_3: 537.213); 415.263 (calc. for y_4: 415.278) (**SI Figure S56**)

1.2.29 TMPP^{+}-CH$_2$CO-Ala-Ala-Ala-Ala-Ala-NH$_2$ (1d)

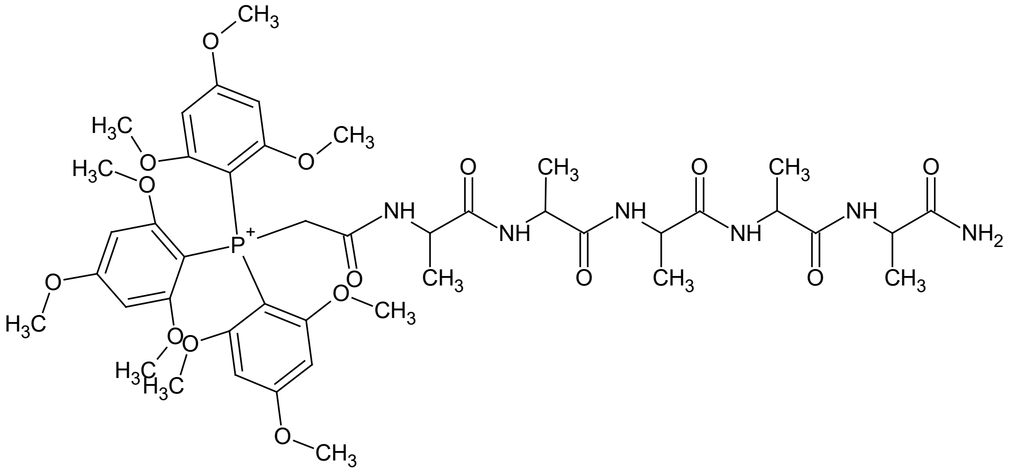

Analytical data:

ESI-MS: *m/z* [M]$^{+}$: 945.395; calc. for $C_{44}H_{62}N_6O_{15}P$ [M]$^{+}$: 945.401 (**SI Figure S57**)

ESI-MS/MS: precursor ion at *m/z* [M]$^{+}$: 945.395, collision energy 48 eV: 616.223 (calc. for a_1: 616.231); 687.258 (calc. for a_2: 687.268); 758.291 (calc. for a_3: 758.305); 829.340 (calc. for a_4: 829.341); 644.233 (calc. for b_1: 644.226); 715.237 (calc. for b_2: 715.263) (**SI Figure S58**)

1.2.30 TMPP^{+}-CH$_2$CO-Ala-Ala-Pro-Ala-Ala-NH$_2$ (2d)

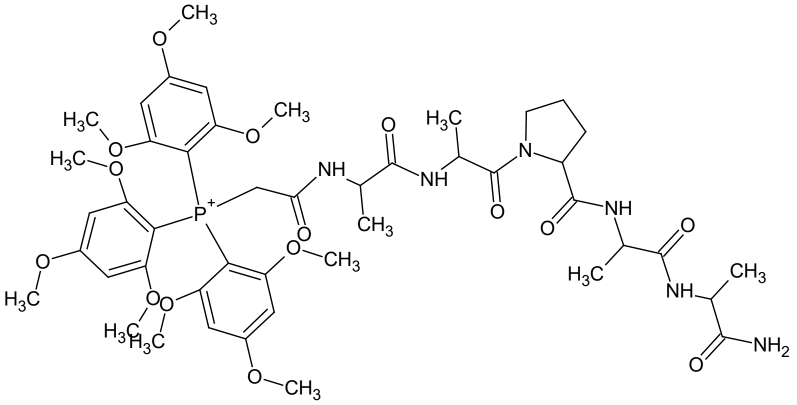

Analytical data:

ESI-MS: *m/z* [M]$^{+}$: 971.405; calc. for $C_{46}H_{64}N_6O_{15}P$ [M]$^{+}$: 971.416 (**SI Figure S59**)

ESI-MS/MS: precursor ion at *m/z* [M]$^{+}$: 971,405, collision energy 50 eV: 616.220 (calc. for a_1: 616.231); 687.257 (calc. for a_2: 687.268); 855.346 (calc. for a_4: 855.358); 644.199 (calc. for b_1: 644.226); 715.255 (calc. for b_2: 715.263) (**SI Figure S60**)

1.2.31 TMPP⁺-CH₂CO-Asp-Gly-Arg-Thr-Leu-NH₂ (3d)

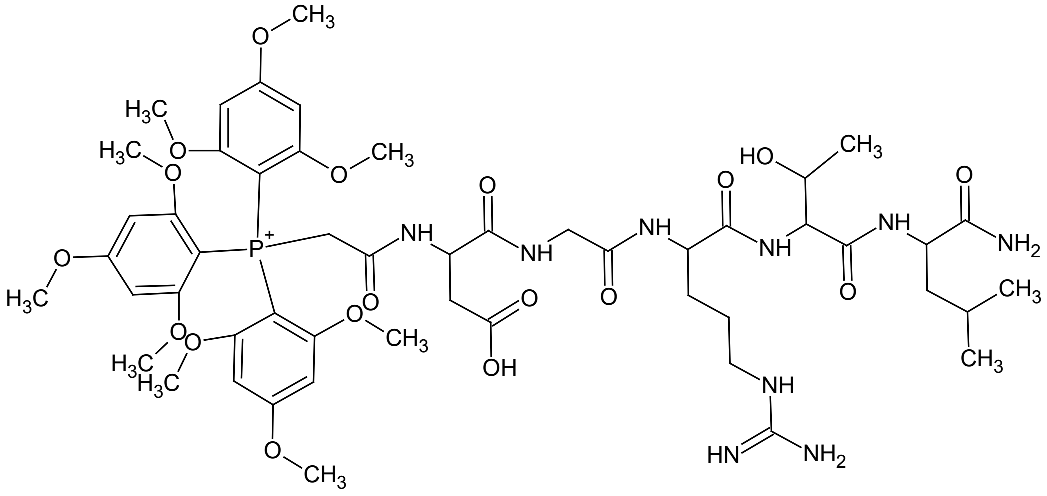

Analytical data:

ESI-MS: *m/z* [M]⁺: 1132.483; calc. for $C_{51}H_{75}N_9O_{18}P$ [M]⁺: 1132.494; *m/z* [M+H]²⁺: 566.752; calc. for $C_{51}H_{76}N_9O_{18}P$ [M+H]²⁺: 566.752 (**SI Figure S61**)

ESI-MS/MS: precursor ion at *m/z* [M+H]²⁺: 566.752, collision energy 25 eV: 717.242 (calc. for a_2: 717.242); 437.187 (calc. for a_3^{2+}: 437.175); 487.706 (calc. for a_4: 487.699); 688.207 (calc. for b_1: 688.215); 745.237 (calc. for b_2: 745.237); 451,177 (calc. for b_3^{2+}: 451.173); 501.203 (calc. for b_4^{2+}: 501.696); 762.248 (calc. for c_2: 762.263); 445.284 (calc. for y_4: 445.288) (**SI Figure S62**)

1.2.32 TMPP+-CH2CO-Ala-Gly-Arg-Thr-Leu-NH2 (4d)

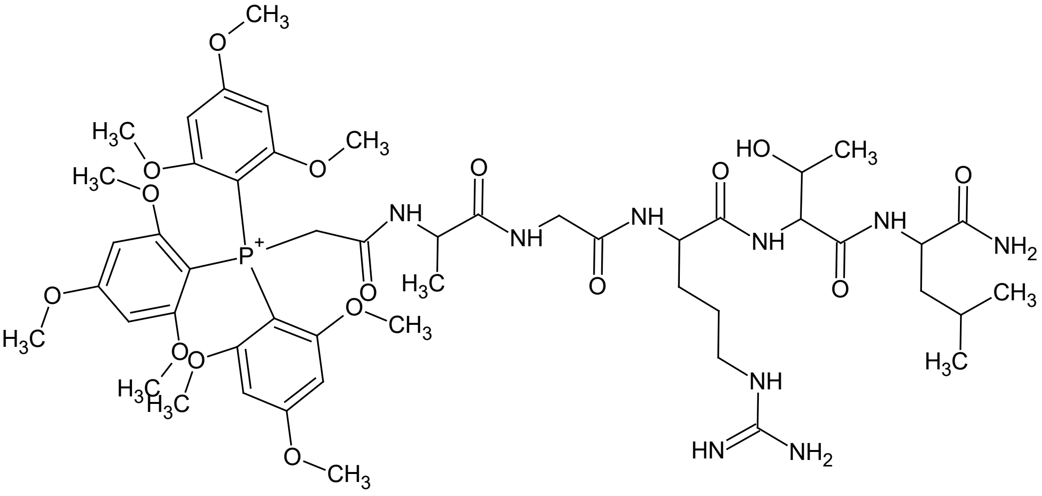

Analytical data:

ESI-MS: *m/z* $[M]^{+}$: 1088.509; calc. for $C_{50}H_{75}N_9O_{18}P$ $[M]^{+}$: 1088.506; *m/z* $[M+H]^{2+}$: 544.765; calc. for $C_{50}H_{76}N_9O_{18}P$ $[M+H]^{2+}$: 544.757 (**SI Figure S63**)

ESI-MS/MS: precursor ion at *m/z* $[M+H]^{2+}$: 566.752, collision energy 25 eV: 616.228 (calc. for a_1: 616.231); 673.245 (calc. for a_2: 673.252); 415.187 (calc. for a_3^{2+}: 415.180); 465.709 (calc. for a_4^{2+}: 465.704); 644.221 (calc. for b_1: 644.226); 701.254 (calc. for b_2: 701.247); 429.183 (calc. for b_3^{2+}: 429.178); 479.713 (calc. for b_5^{2+}: 479.702); 661.243 (calc. for c_1: 661.252) (**SI Figure S64**)

1.2.33 TMPP$^+$-CH_2CO-Asp-Gly-Lys-Thr-Leu-NH_2 (5d)

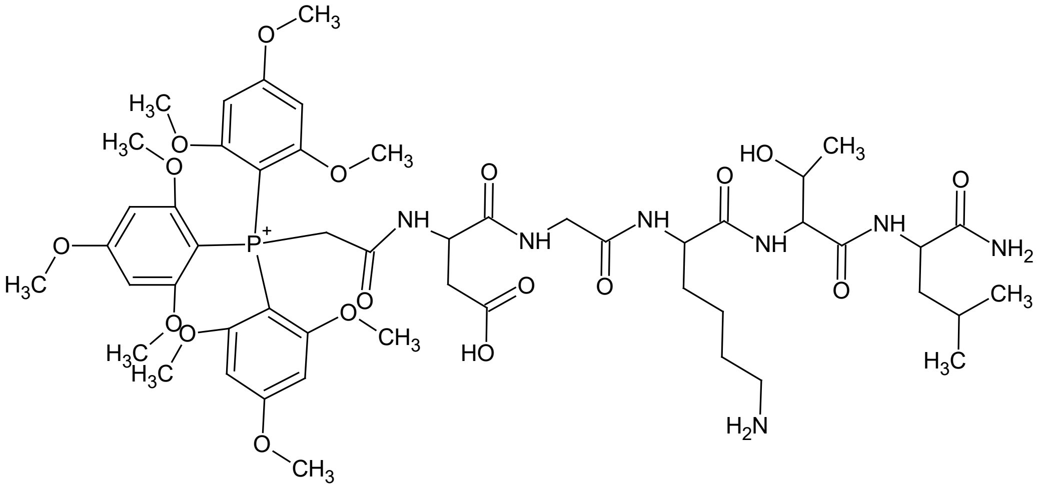

Analytical data:

ESI-MS: *m/z* $[M]^+$: 1104.509; calc. for $C_{51}H_{75}N_7O_{18}P$ $[M]^+$: 1104.490; *m/z* $[M+H]^{2+}$: 552.758; calc. for $C_{51}H_{76}N_7O_{18}P$ $[M+H]^{2+}$: 552.749 (**SI Figure S65**)

ESI-MS/MS: precursor ion at *m/z* $[M+H]^{2+}$: 552.758, collision energy 17 eV: 717.236 (calc. for a_2: 717.242); 473.704 (calc. for a_4^{2+}: 473.696); 946.381 (calc. for a_4: 946.385); 688.203 (calc. for b_1: 688.215); 745.232 (calc. for b_2: 745.237); 437.179 (calc. for b_3^{2+}: 437.197); 873.332 (calc. for b_3: 873.332); 974.373 (calc. for b_4: 974.380); 487.702 (calc. for b_4^{2+}: 487.702); 762.263 (calc. for c_1: 762.263) (**SI Figure S66**)

1.2.34 TMPP+-CH_2CO-Asp-Gly-Ala-Thr-Leu-NH_2 (6d)

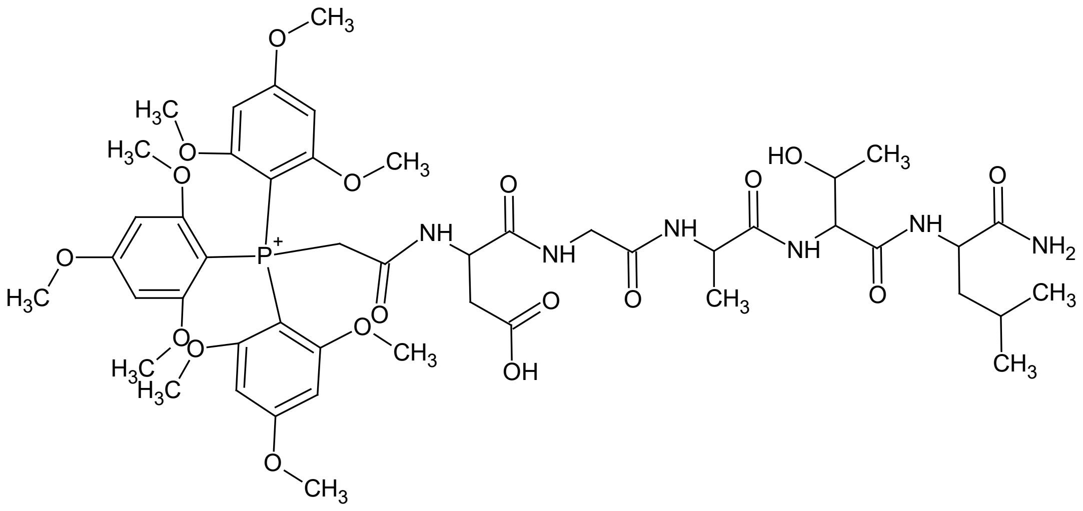

Analytical data:

ESI-MS: *m/z* $[M]^+$: 1047.438; calc. for $C_{48}H_{68}N_6O_{18}P$ $[M]^+$: 1047.440 (**SI Figure S67**)

ESI-MS/MS: precursor ion at *m/z* $[M]^+$: 1047.438, collision energy 50 eV: 660.209 (calc. for a_1: 660.220); 717.246 (calc. for a_2: 717.242); 788.281 (calc. for a_3: 788.279); 889.325 (calc. for a_4: 889.327); 688.216 (calc. for b_1: 688.215); 745.229 (calc. for b_2: 745.237); 816.299 (calc. for b_3: 816.274); 917.333 (calc. for b_4: 917.322) (**SI Figure S68**)

1.2.35 TMPP+-CH_2CO-Asp-Gly-Arg-Ala-Leu-NH_2 (7d)

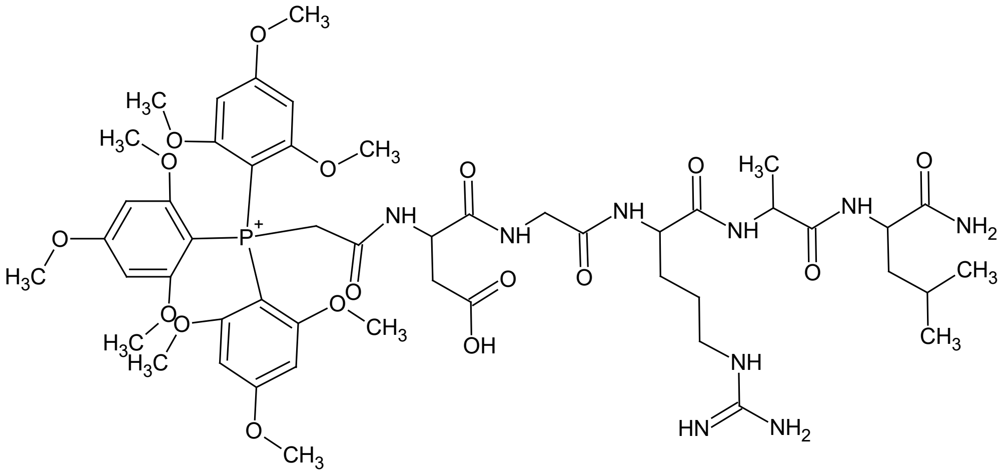

Analytical data:

ESI-MS: *m/z* $[M]^+$: 1102.505; calc. for $C_{51}H_{75}N_7O_{18}P$ $[M]^+$: 1102.486; *m/z* $[M+H]^{2+}$: 551.754; calc. for $C_{51}H_{76}N_7O_{18}P$ $[M+H]^{2+}$: 551,745 (**SI Figure S69**)

ESI-MS/MS: precursor ion at *m/z* $[M+H]^{2+}$: 551.,754, collision energy 25 eV: 660.217 (calc. for a_1: 660.220); 717.250 (calc. for a_2: 717.242); 437.175 (calc. for a_3^{2+}: 437.175); 472.705 (calc. for a_4^{2+}: 472.694); 688.212 (calc. for b_1: 688.215); 745.243 (calc. for b_2: 745.237); 451.183 (calc. for b_3^{2+}: 451.173); 486.707 (calc. for b_4^{2+}: 486.691); (calc. for c_1: 762.263); 415.277 (calc. for y_4: 415.278) (**SI Figure S70**)

2. ESI-MS and ESI-MS/MS spectra

2.1 ESI-MS and ESI-CID-MS/MS spectra

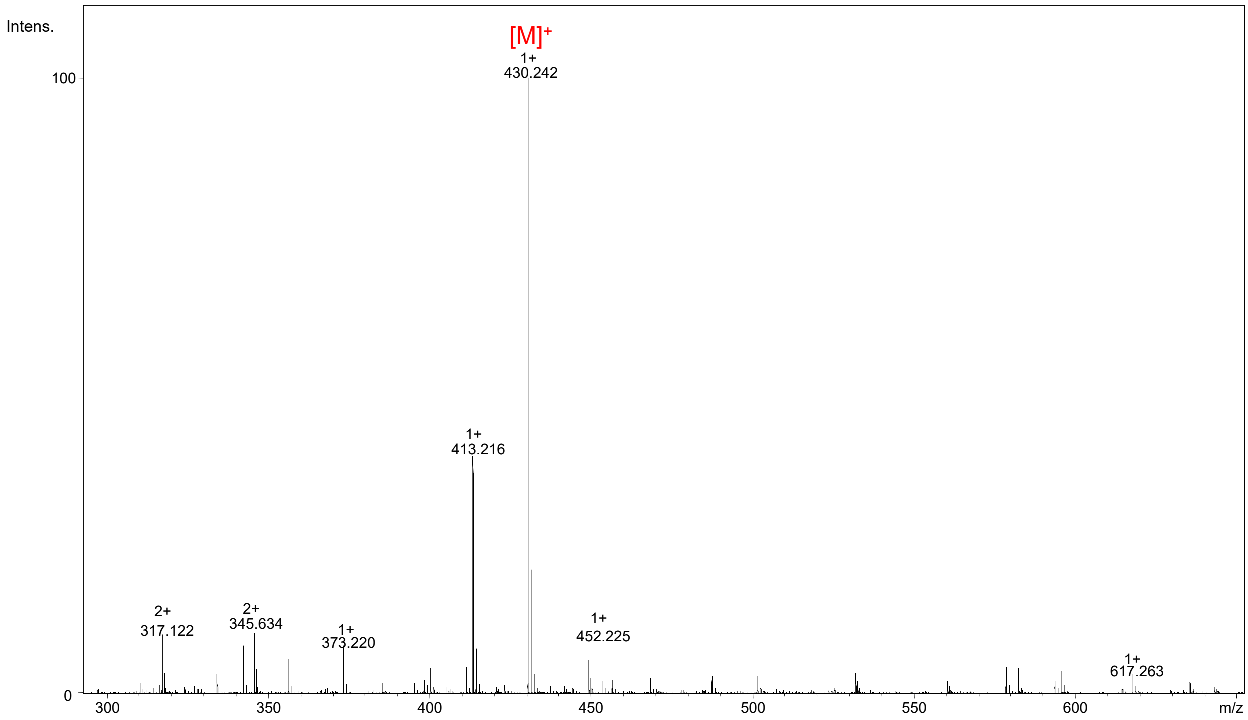

Figure S1. ESI-MS spectrum of H-Gly-Ala-Ala-Ala-Ala-Ala-NH_2 (1)

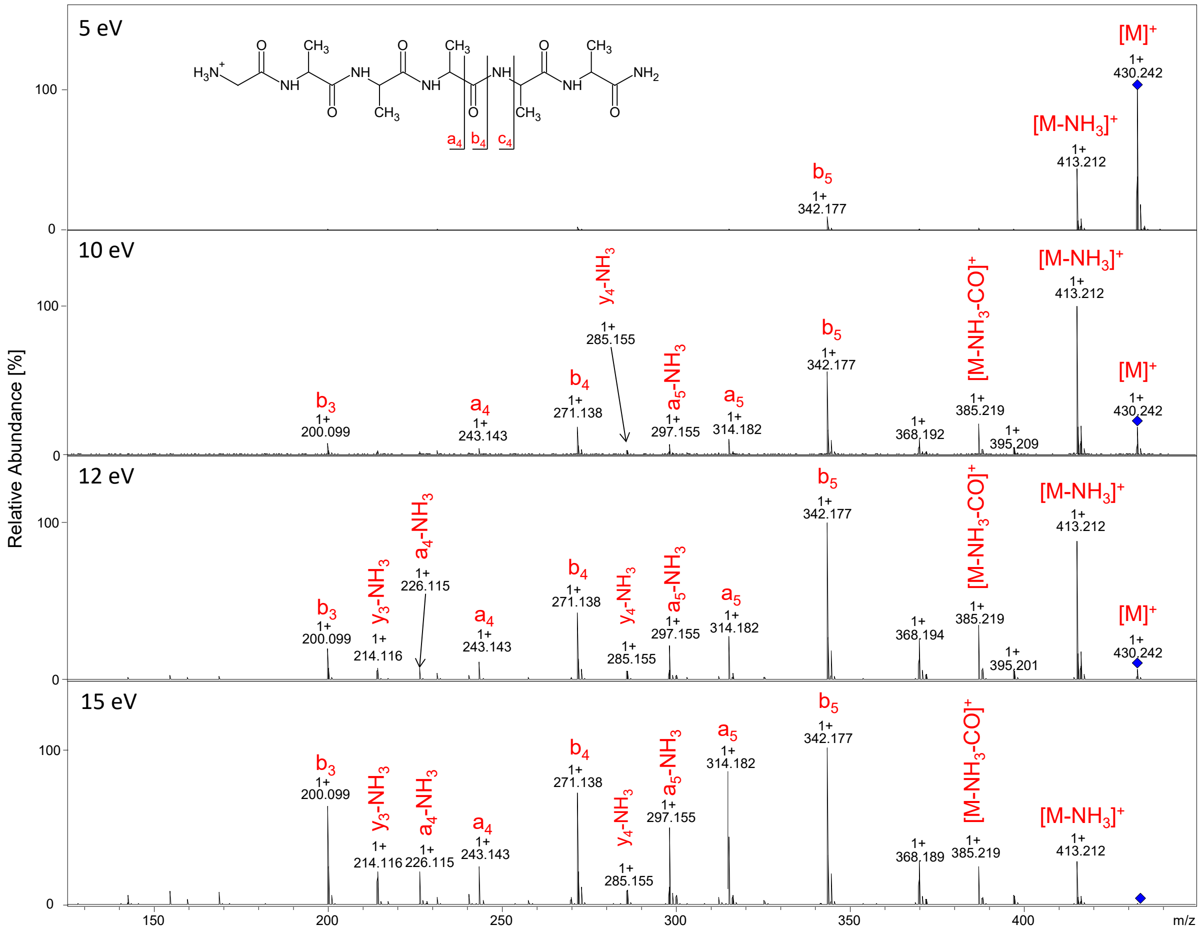

Fig S2. ESI-CID-MS/MS spectra of H-Gly-Ala-Ala-Ala-Ala-Ala-NH₂ (1). Precursor ion at *m*/*z* 430.242.

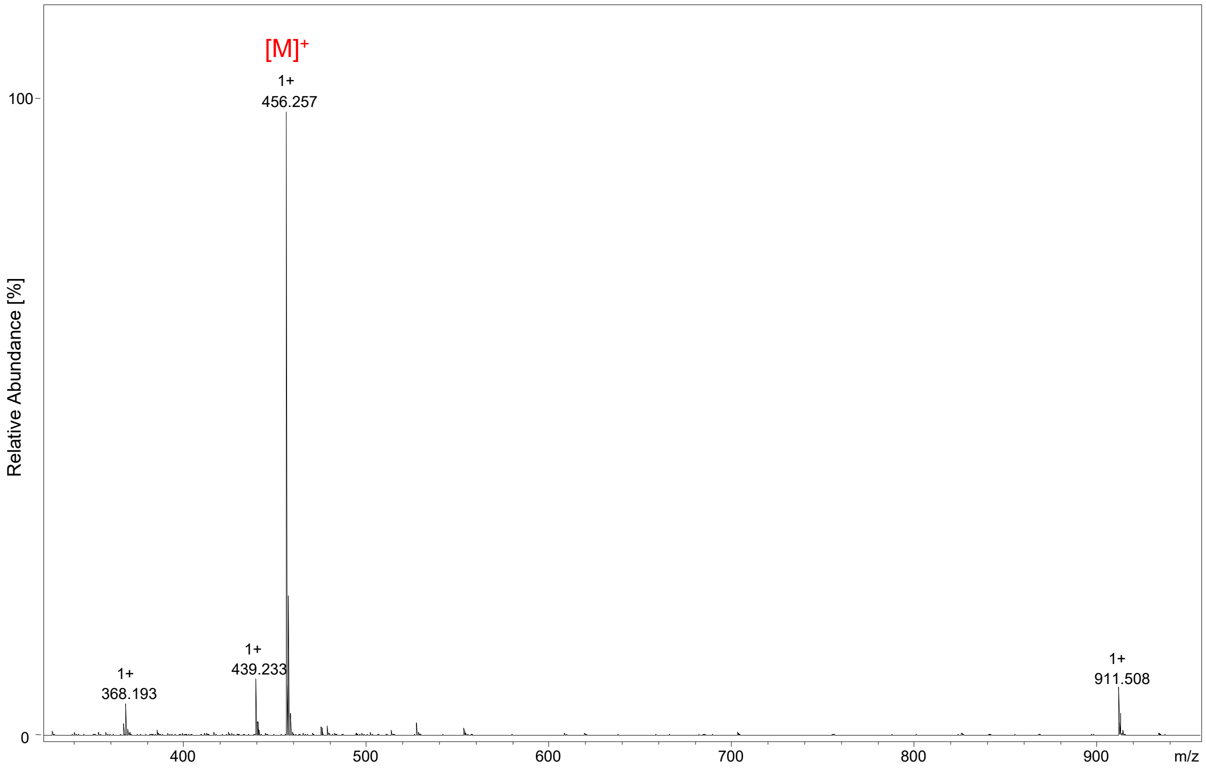

Figure S3. ESI-MS spectrum of H-Gly-Ala-Ala-Pro-Ala-Ala-NH2 (2)

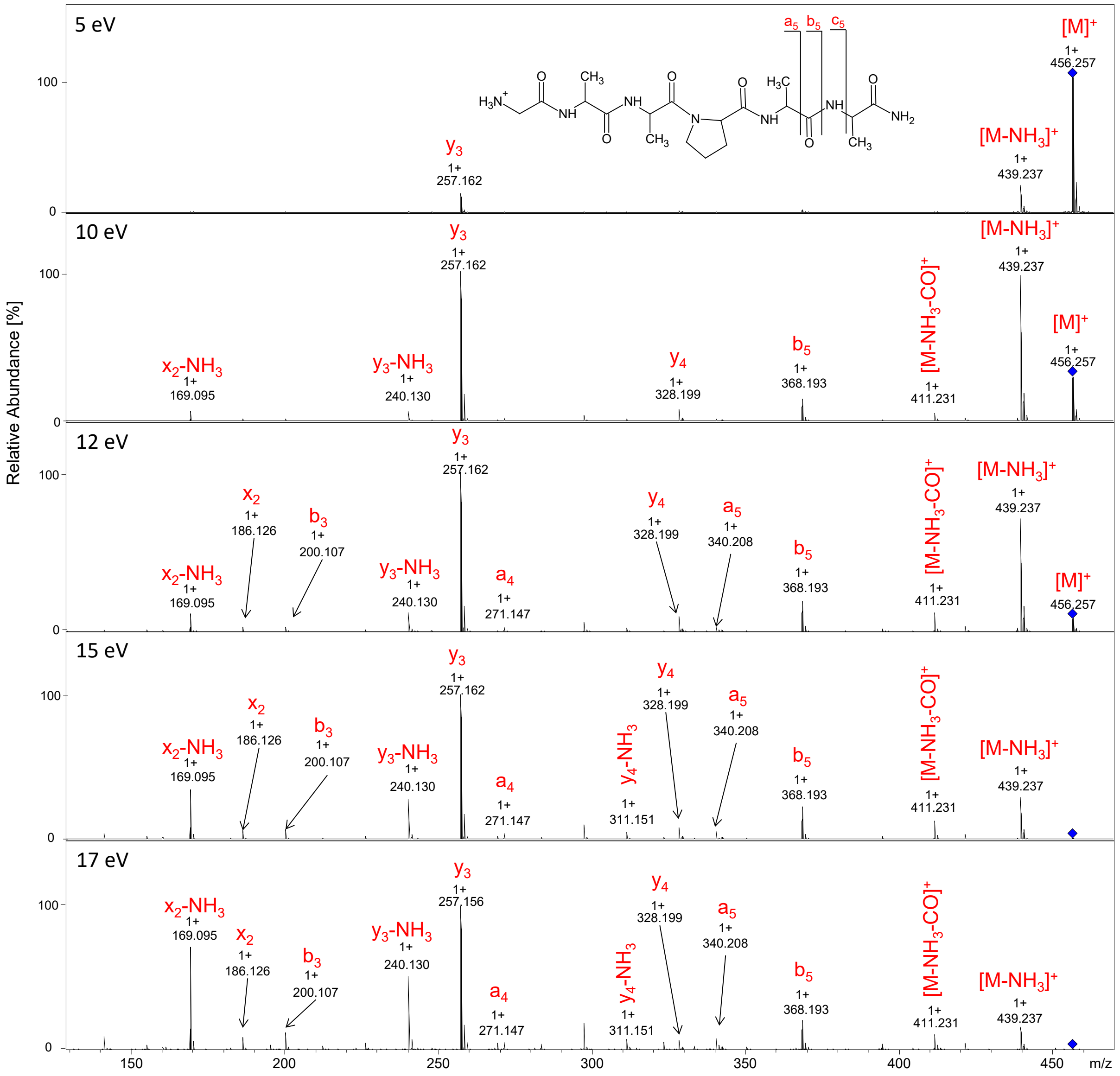

Figure S4. ESI-CID-MS/MS spectra of H-Gly-Ala-Ala-Pro-Ala-Ala-NH$_2$ (2). Precursor ion at *m/z* 456.257.

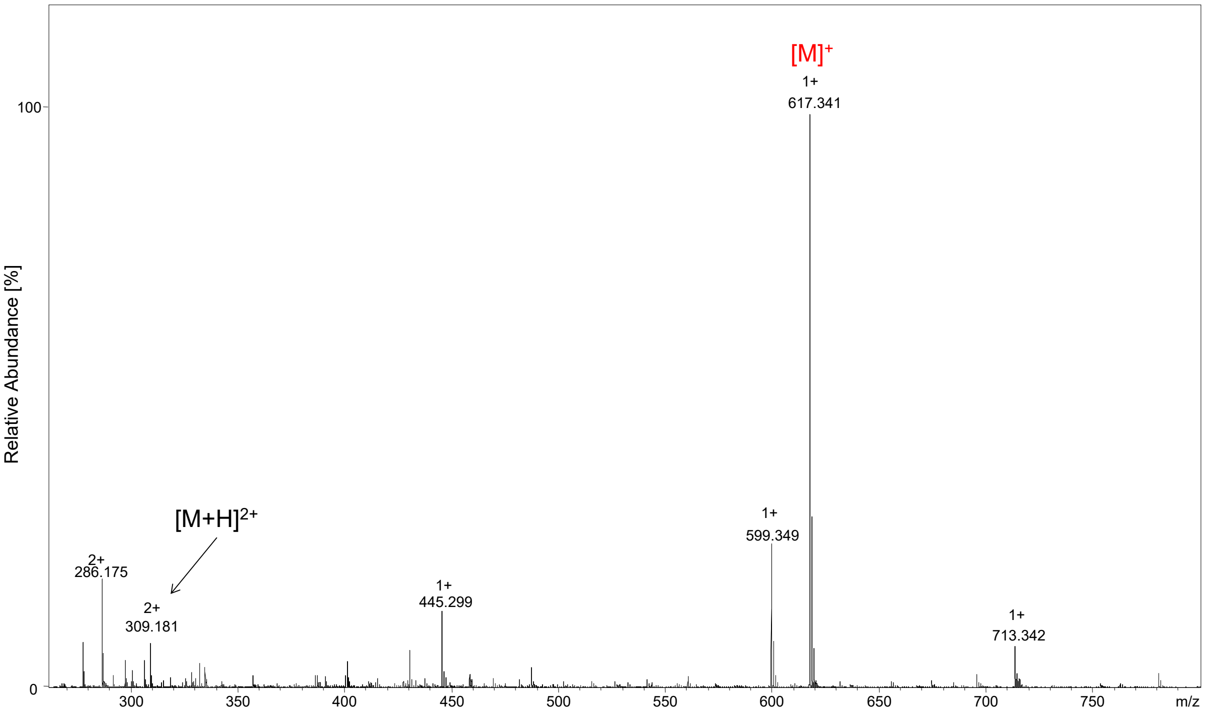

Figure S5. ESI-MS spectrum of H-Gly-Asp-Gly-Arg-Thr-Leu-NH$_2$ (3)

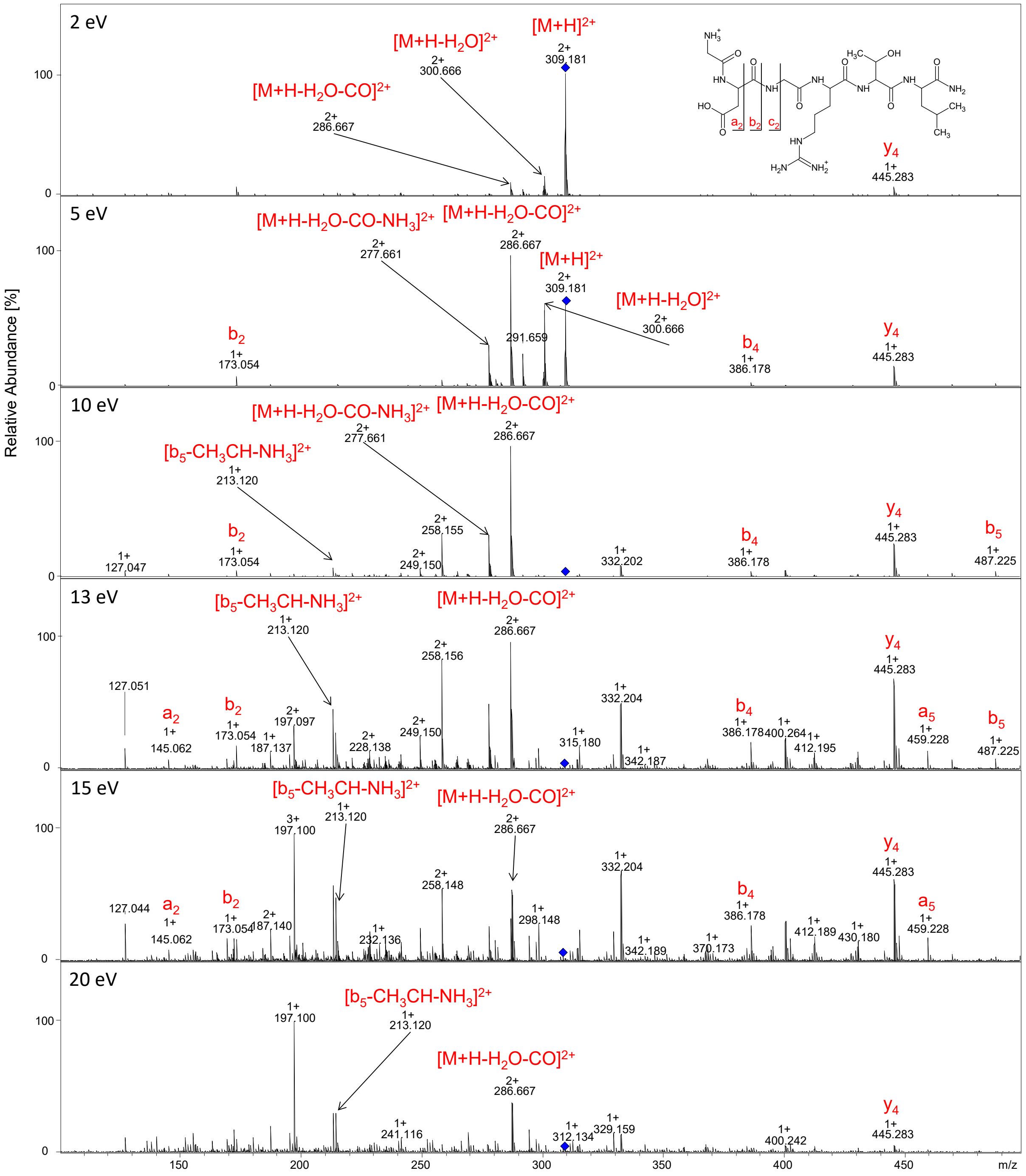

Figure S6. ESI-CID-MS/MS spectra of H-Gly-Asp-Gly-Arg-Thr-Leu-NH₂ (3). Precursor ion at *m/z* 309.181.

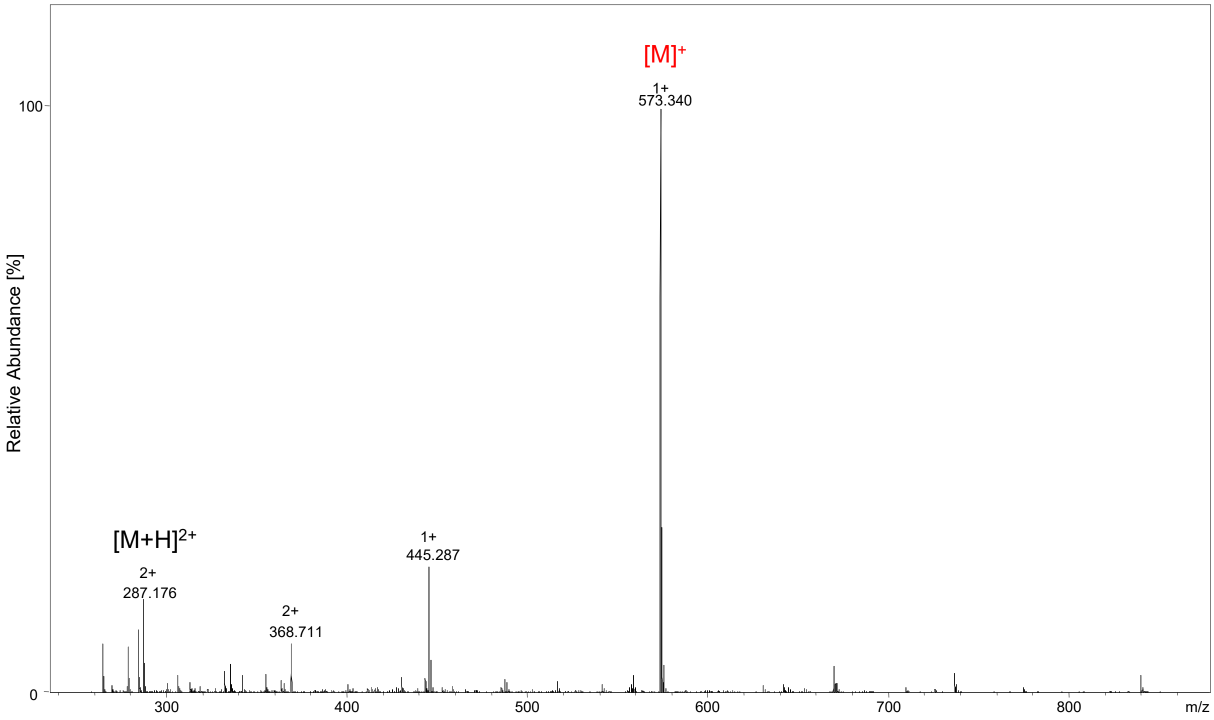

Figure S7. ESI-MS spectrum of H-Gly-Ala-Gly-Arg-Thr-Leu-NH_2 (4)

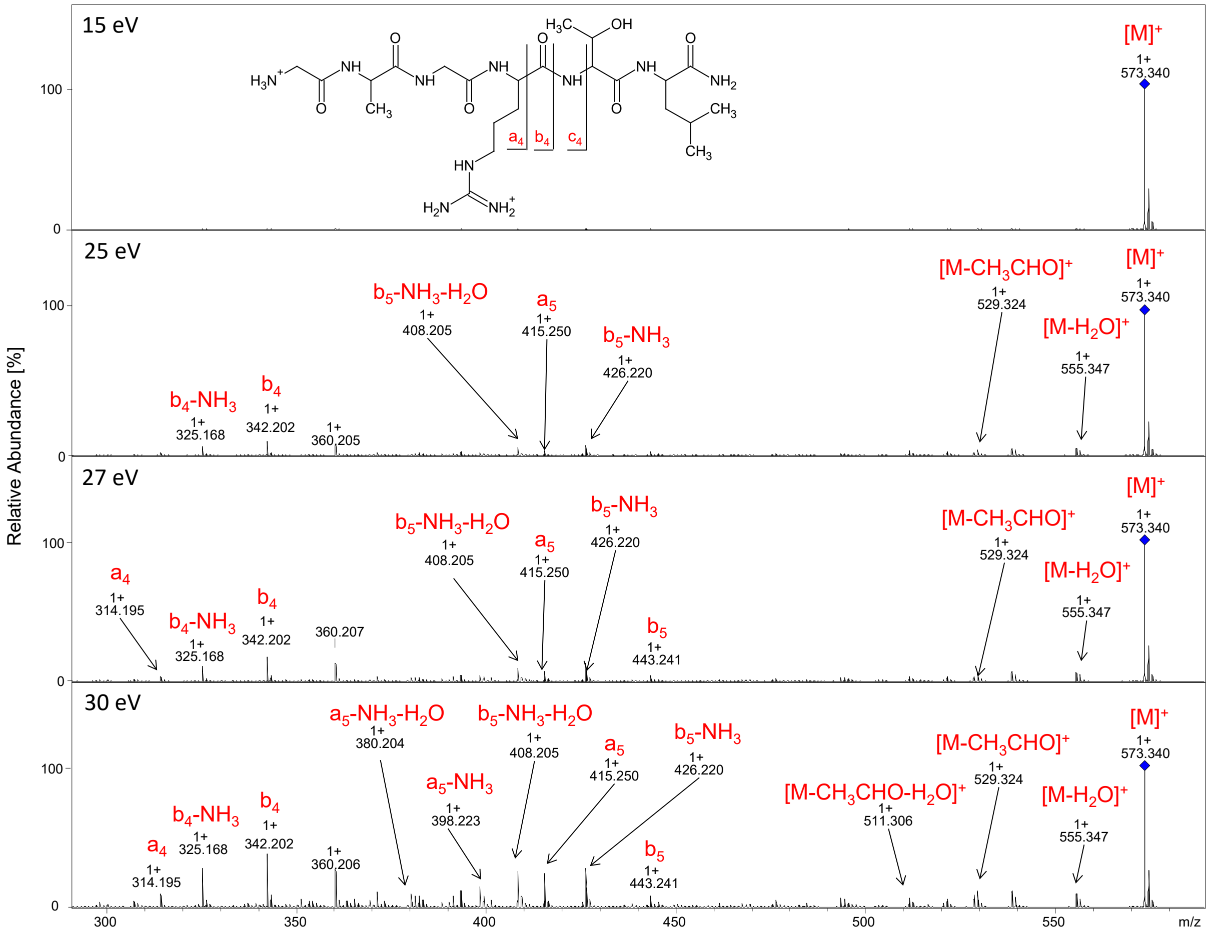

Figure S8. ESI-CID-MS/MS spectra of H-Gly-Ala-Gly-Arg-Thr-Leu-NH2 (4). Precursor ion at *m/z* 573.340.

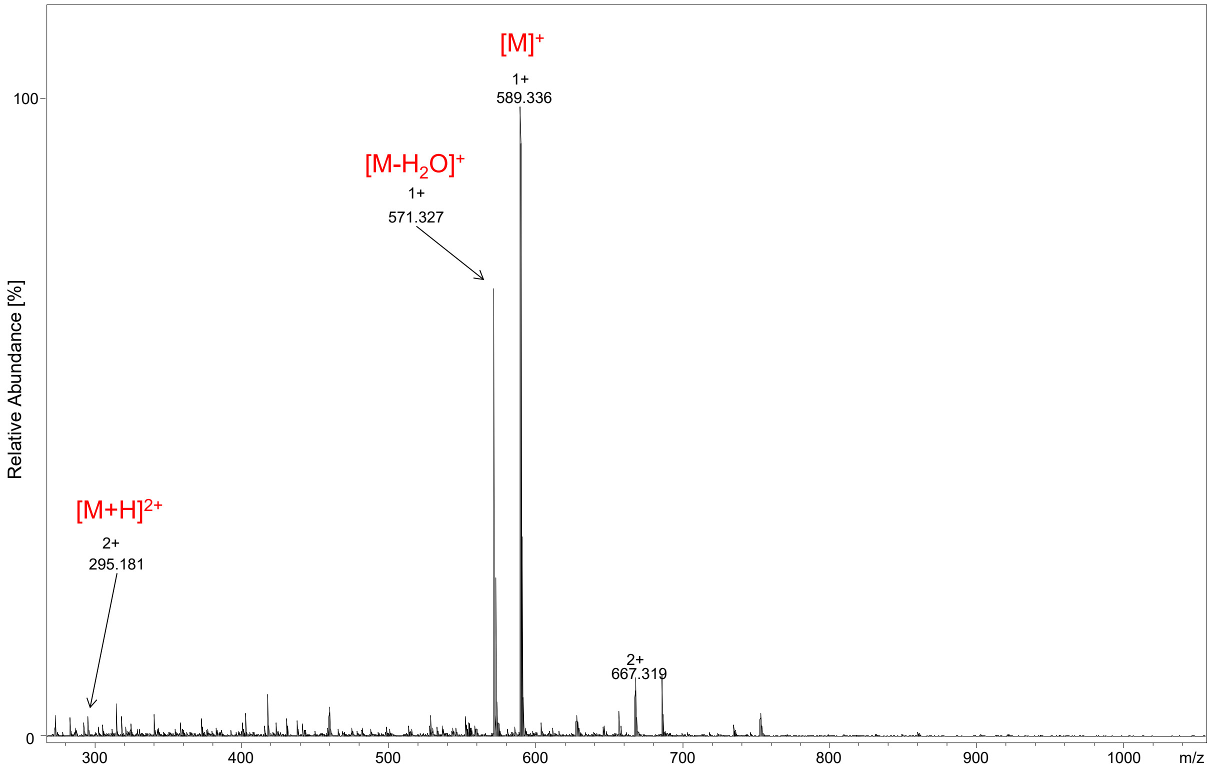

Figure S9. ESI-MS spectrum of H-Gly-Asp-Gly-Lys-Thr-Leu-NH_2 (5)

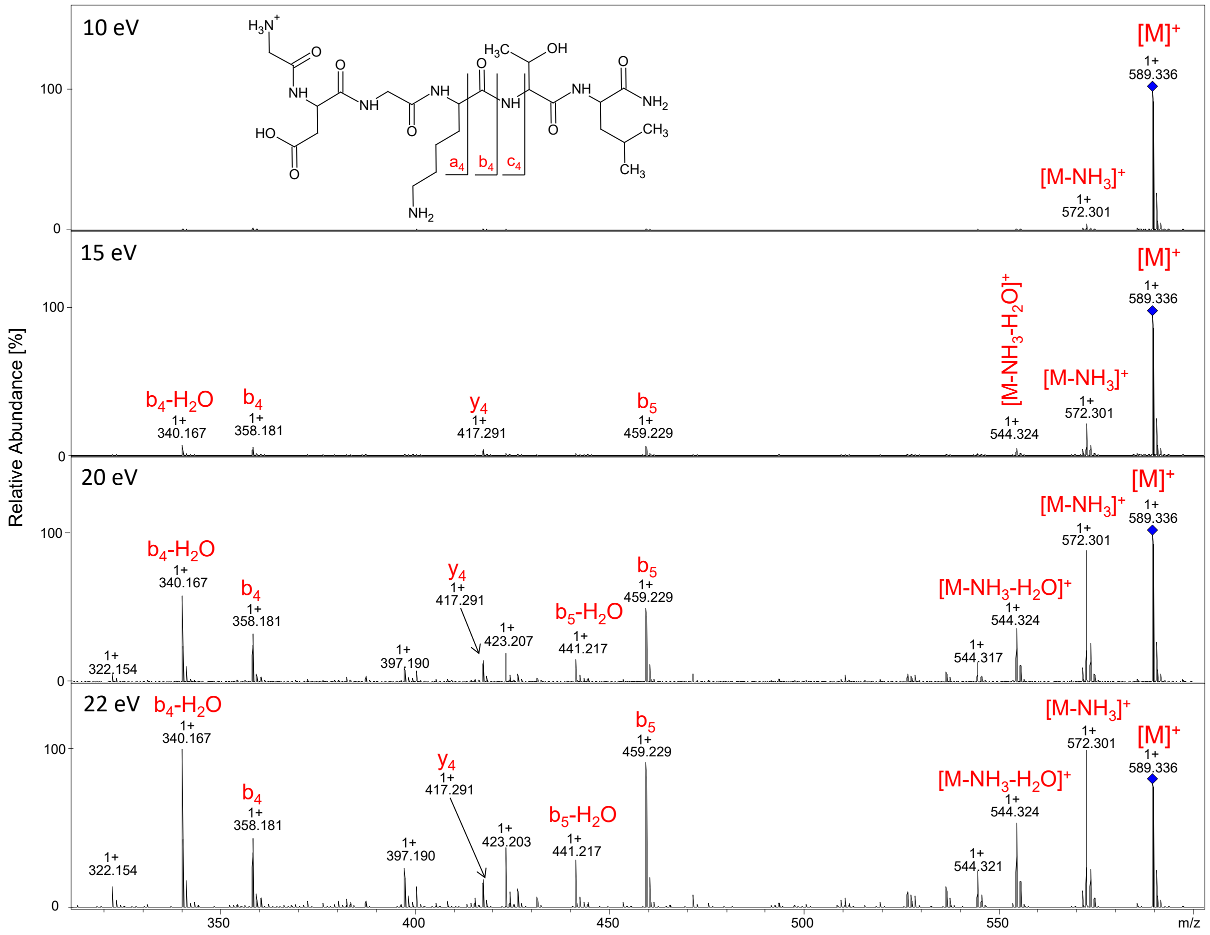

Figure S10. ESI-CID-MS/MS spectra of H-Gly-Asp-Gly-Lys-Thr-Leu-NH_2 (5). Precursor ion at *m*/*z* 589.336.

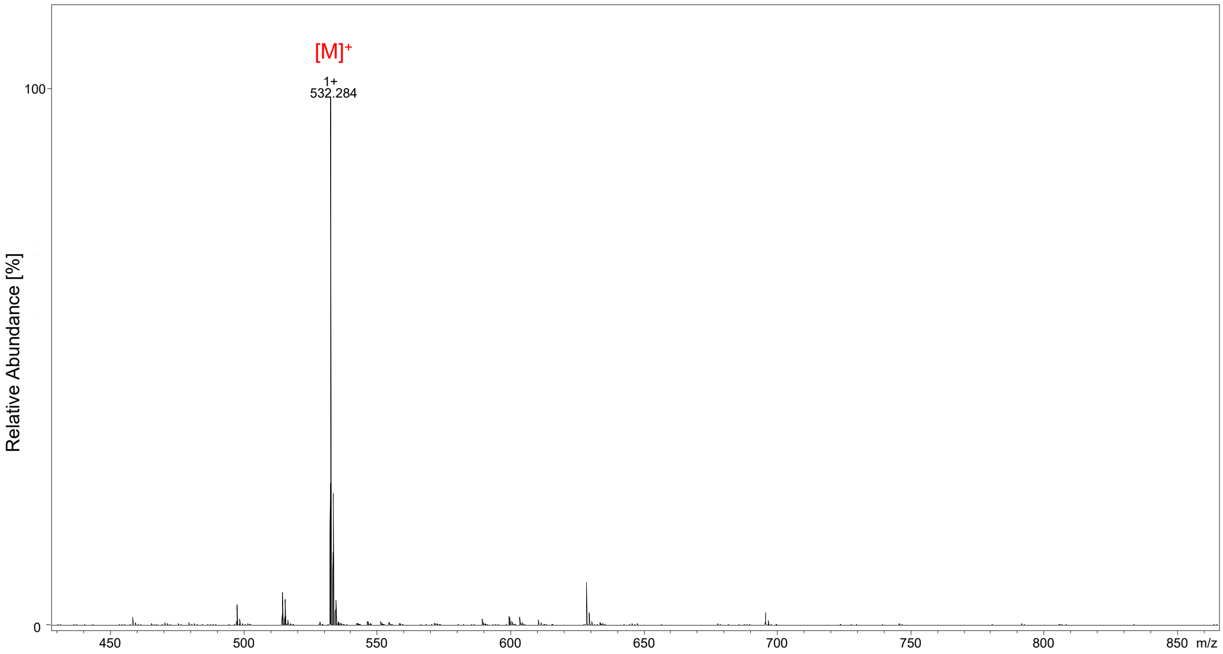

Figure S11. ESI-MS spectrum of H-Gly-Asp-Gly-Ala-Thr-Leu-NH2 (6).

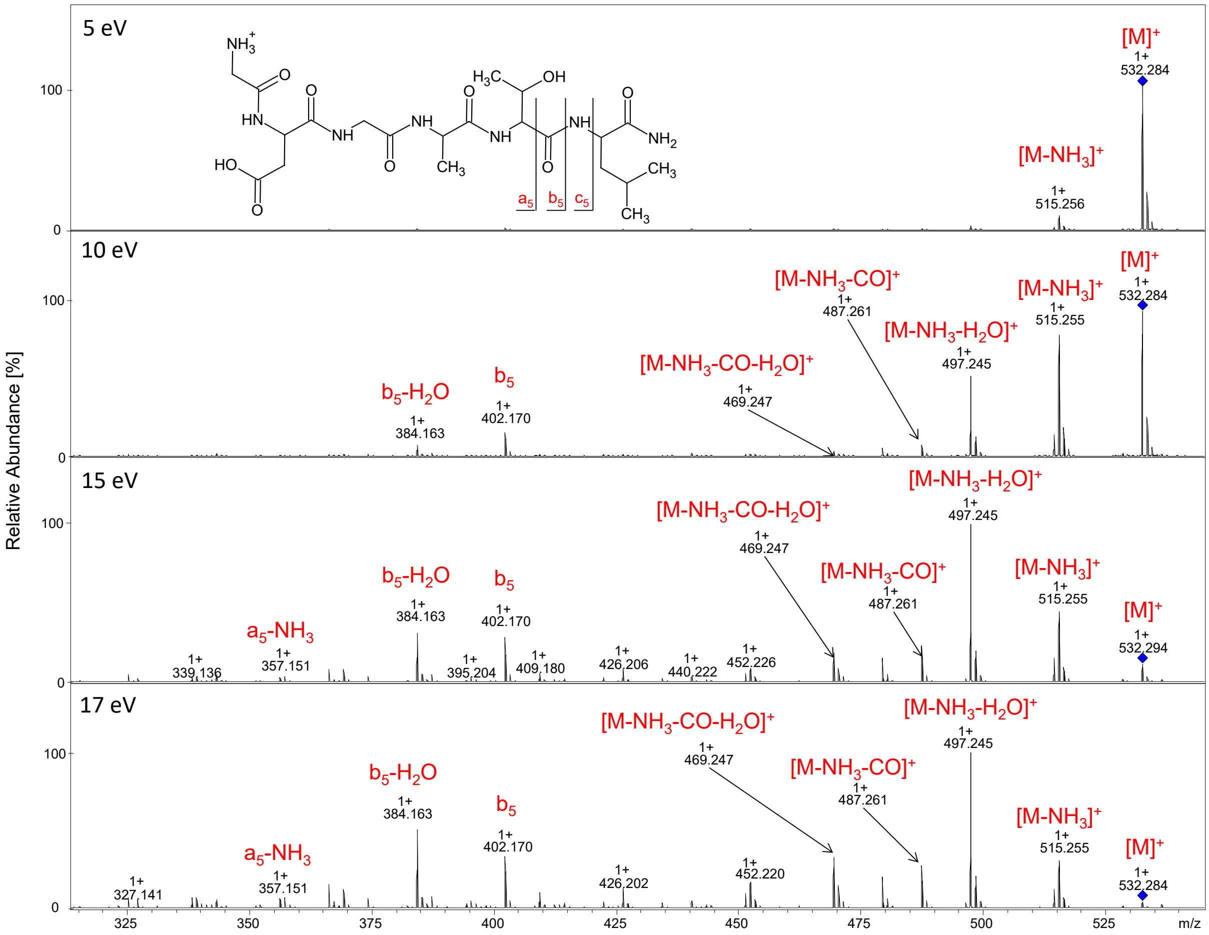

Figure S12. ESI-CID-MS/MS spectra of H-Gly-Asp-Gly-Ala-Thr-Leu-NH_2 (6). Precursor ion at *m/z* 532.294.

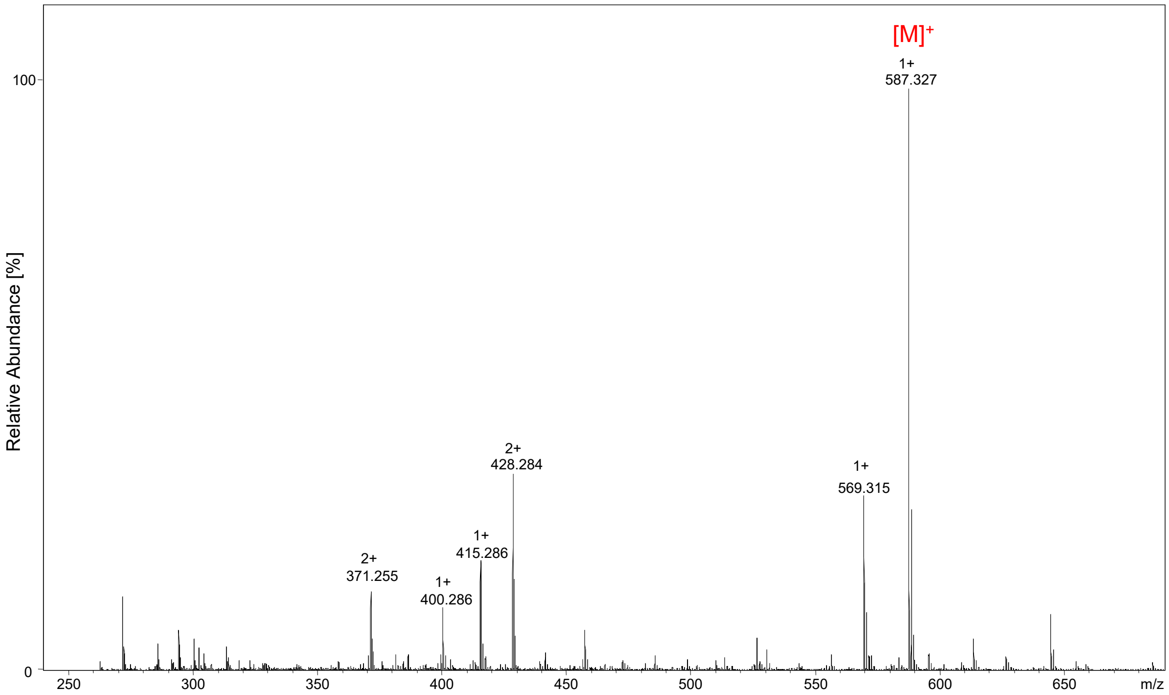

Figure S13. ESI-MS spectrum of H-Gly-Asp-Gly-Lys-Ala-Leu-NH2 (7)

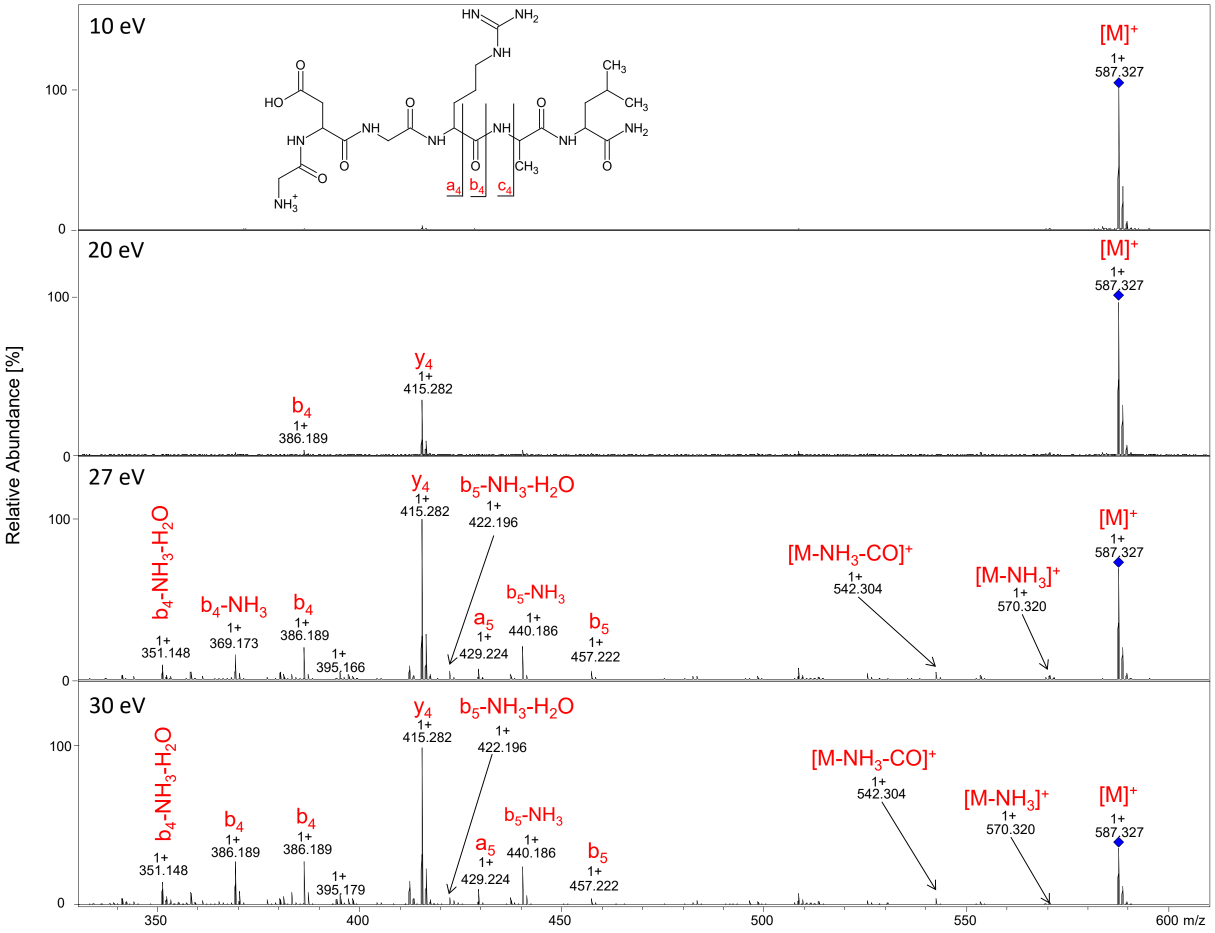

Figure S14. ESI-CID-MS/MS spectra of H-Gly-Asp-Gly-Arg-Ala-Leu-NH_2 (7). Precursor ion at *m/z* 587.327.

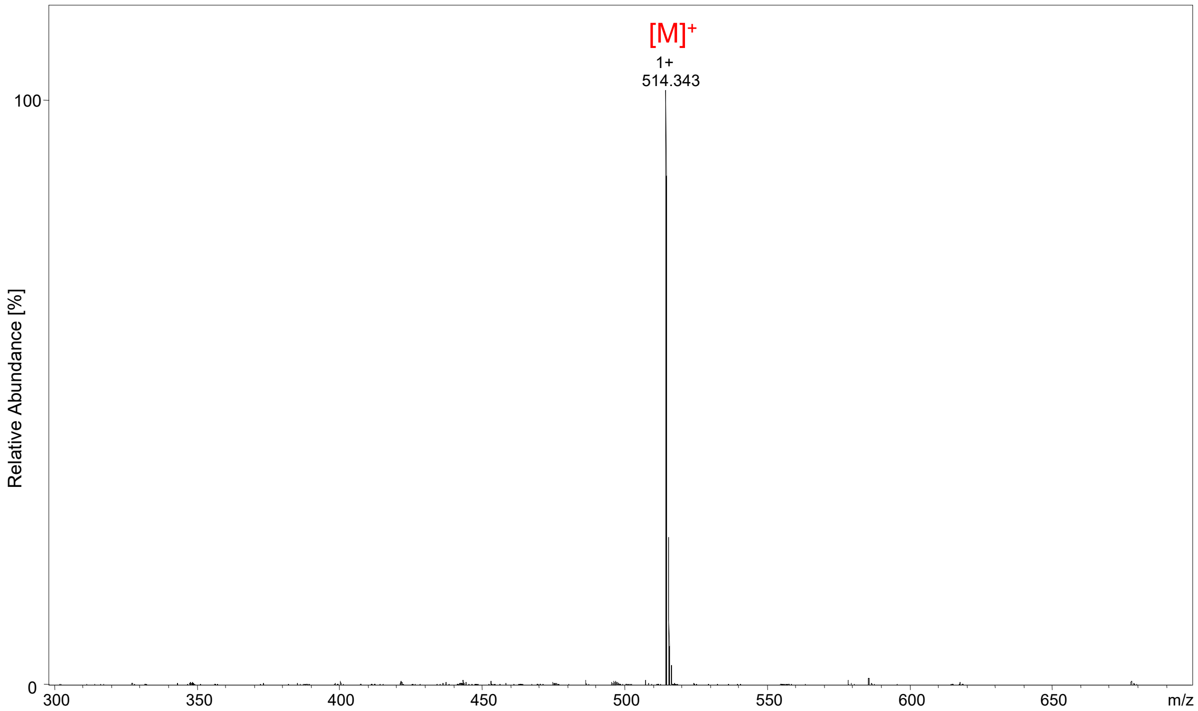

Figure S15. ESI-MS spectrum of TEA$^+$-CH_2CO-Ala-Ala-Ala-Ala-Ala-NH_2 (1a).

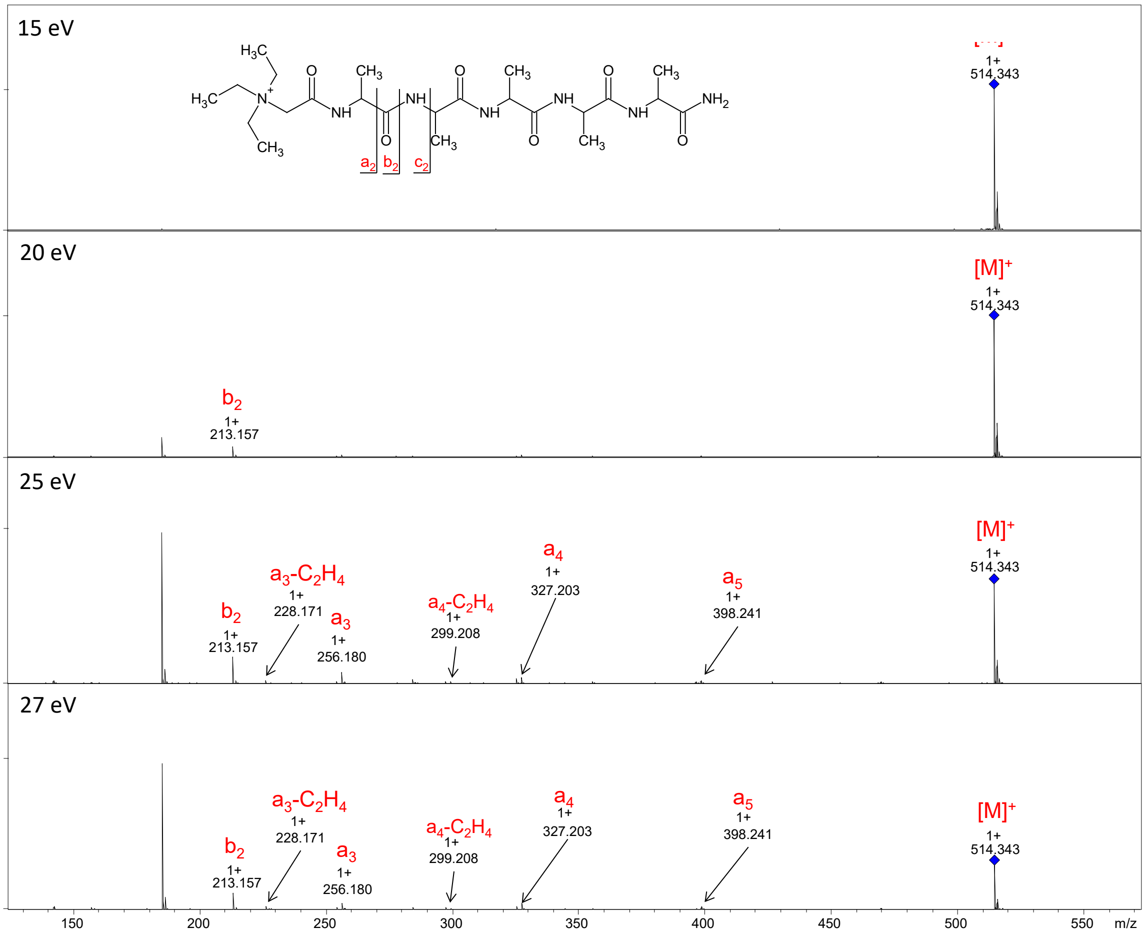

Figure S16. ESI-CID-MS/MS spectra of TEA⁺-CH2CO-Ala-Ala-Ala-Ala-Ala-NH2 (1a). Precursor ion at *m/z* 514.323

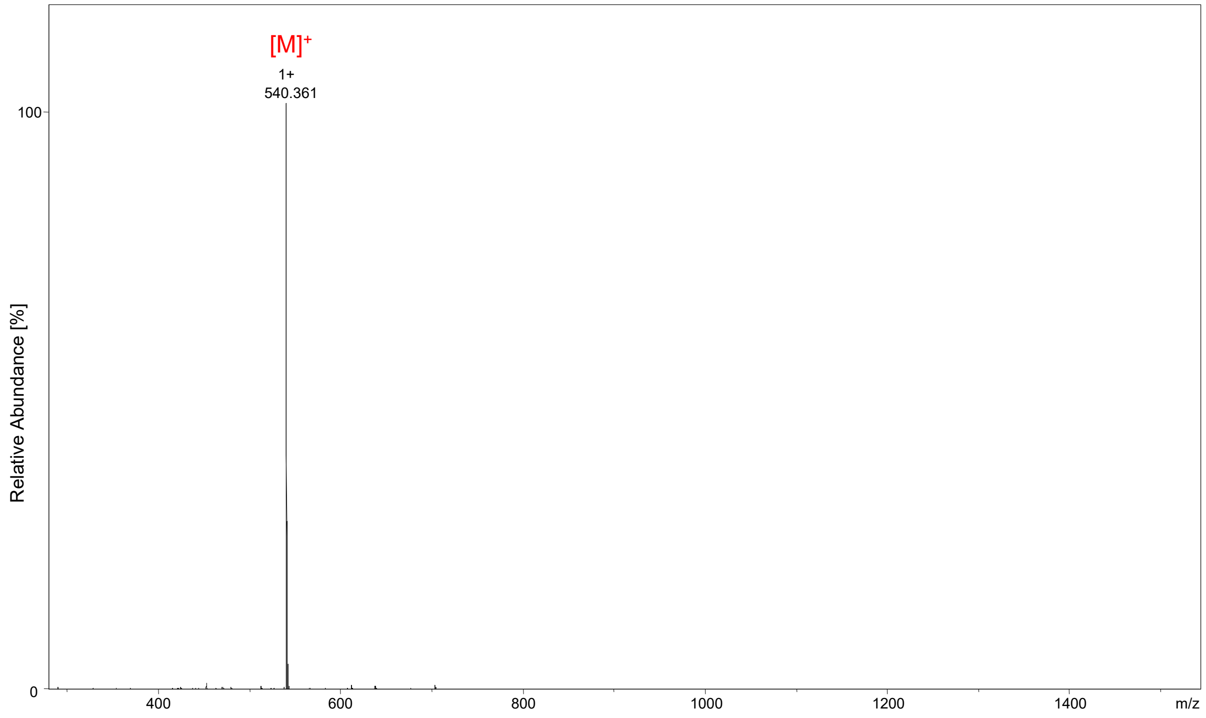

Figure S17. ESI-MS spectrum of TEA+-CH$_2$CO-Ala-Ala-Pro-Ala-Ala-NH$_2$ (2a)

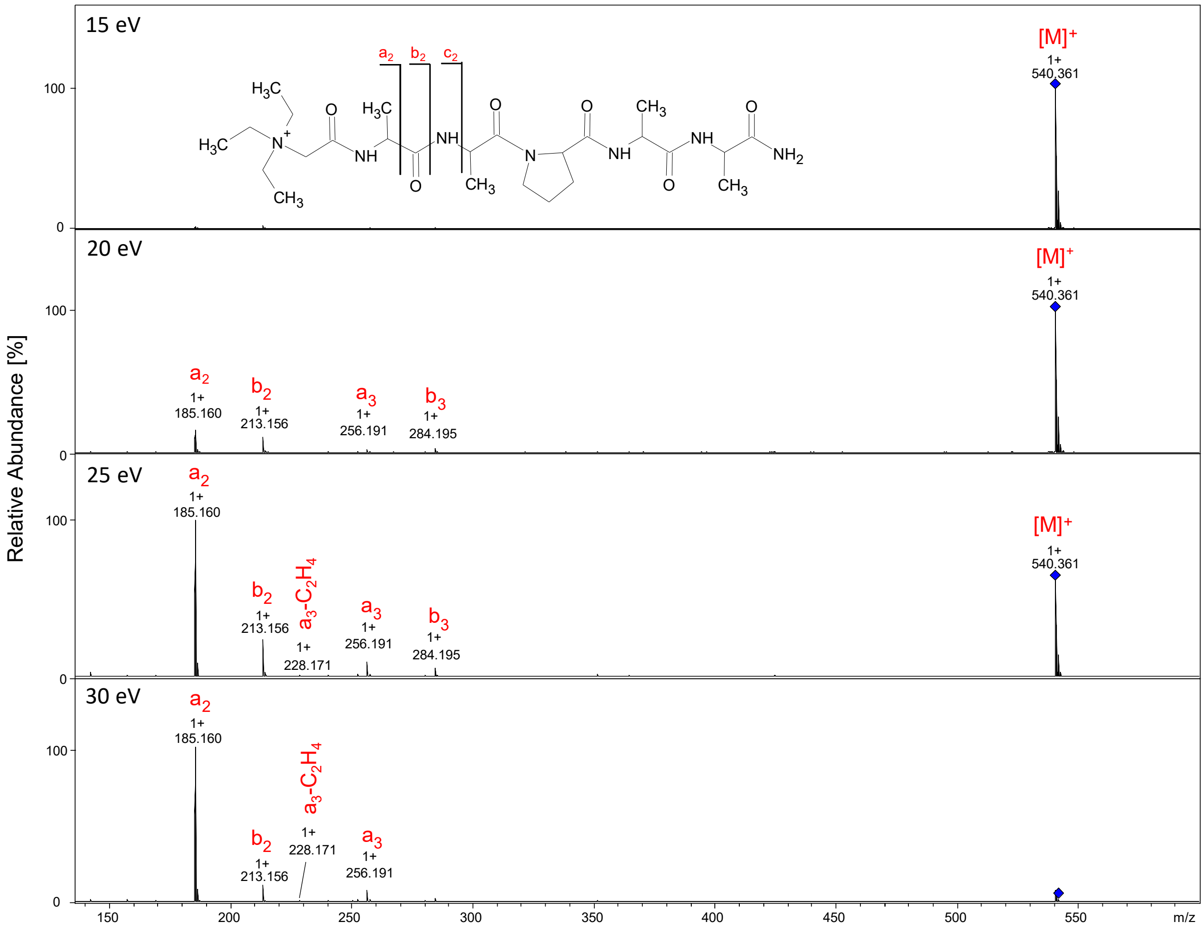

Figure S18. ESI-CID-MS/MS spectra of TEA⁺-CH2CO-Ala-Ala-Pro-Ala-Ala-NH2 (2a). Precursor ion at *m/z* 540.361

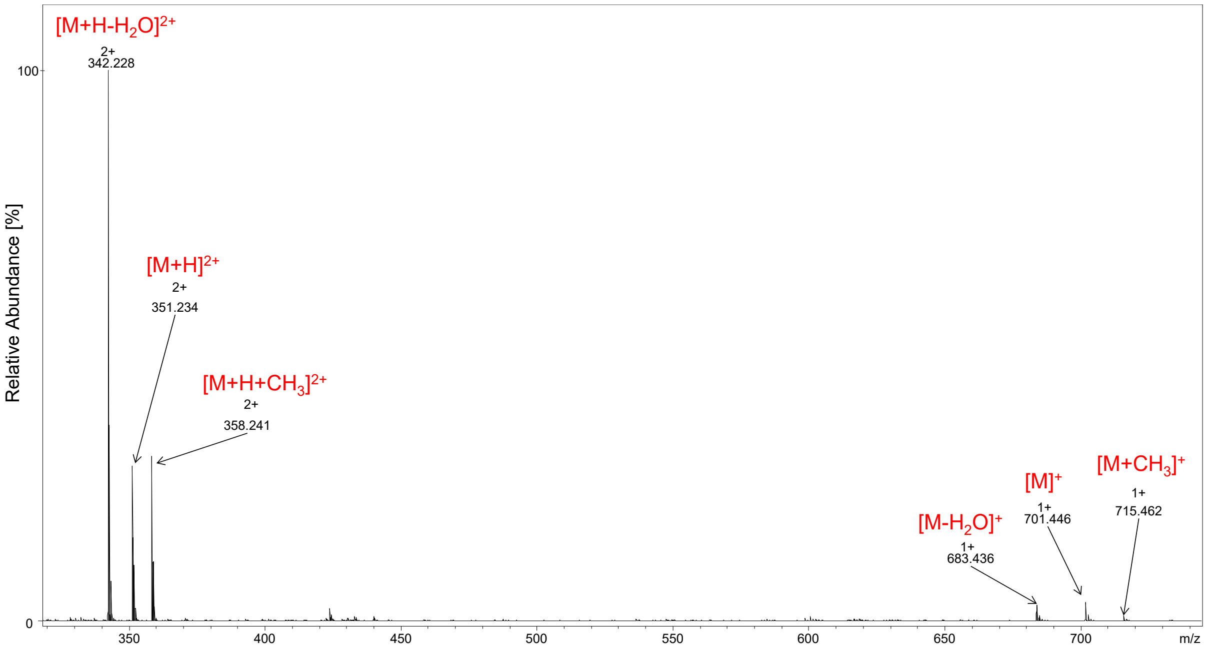

Figure S19. ESI-MS spectrum of TEA⁺-CH2CO-Asp-Gly-Arg-Thr-Leu-NH2 (3a)

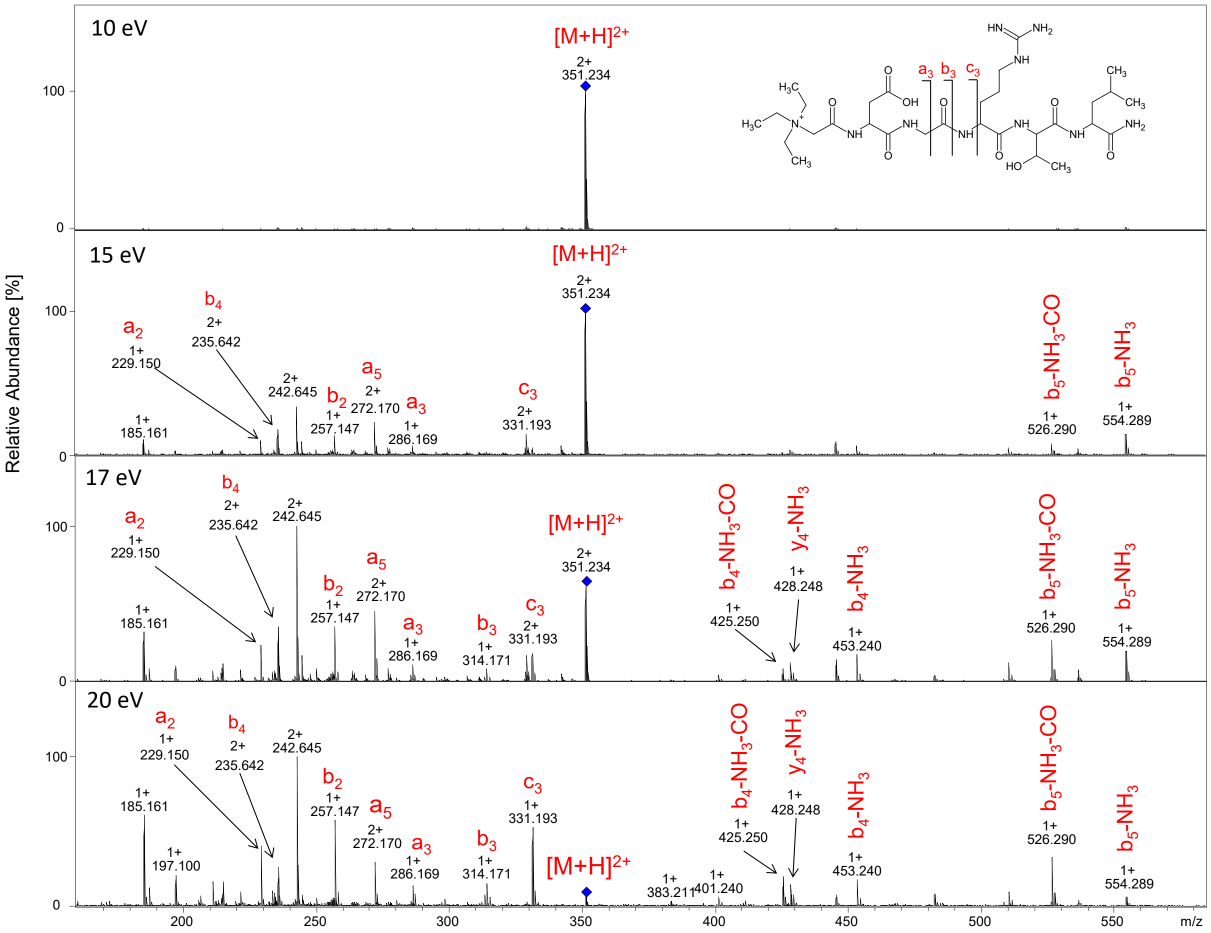

Figure S20. ESI-CID-MS/MS spectra of TEA⁺-CH_2CO-Asp-Gly-Arg-Thr-Leu-NH_2 (3a). Precursor ion at *m/z* 351.234.

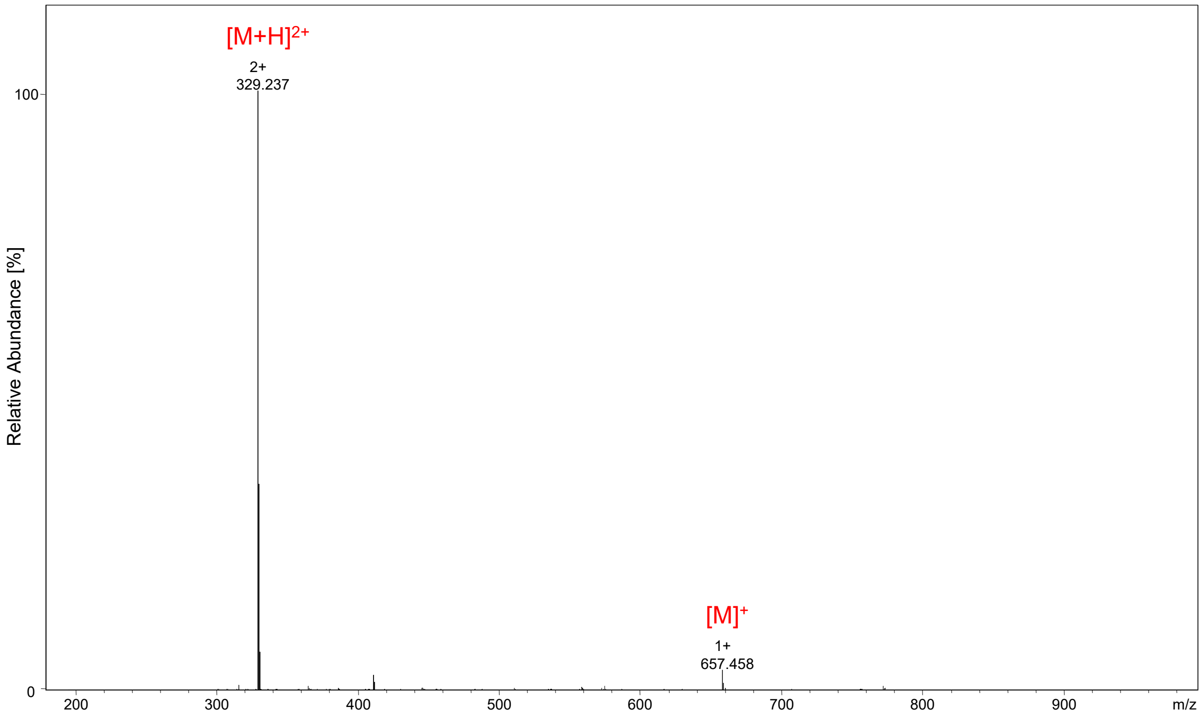

Figure S21. ESI-MS spectrum of TEA⁺-CH2CO-Ala-Gly-Arg-Thr-Leu-NH2 (4a)

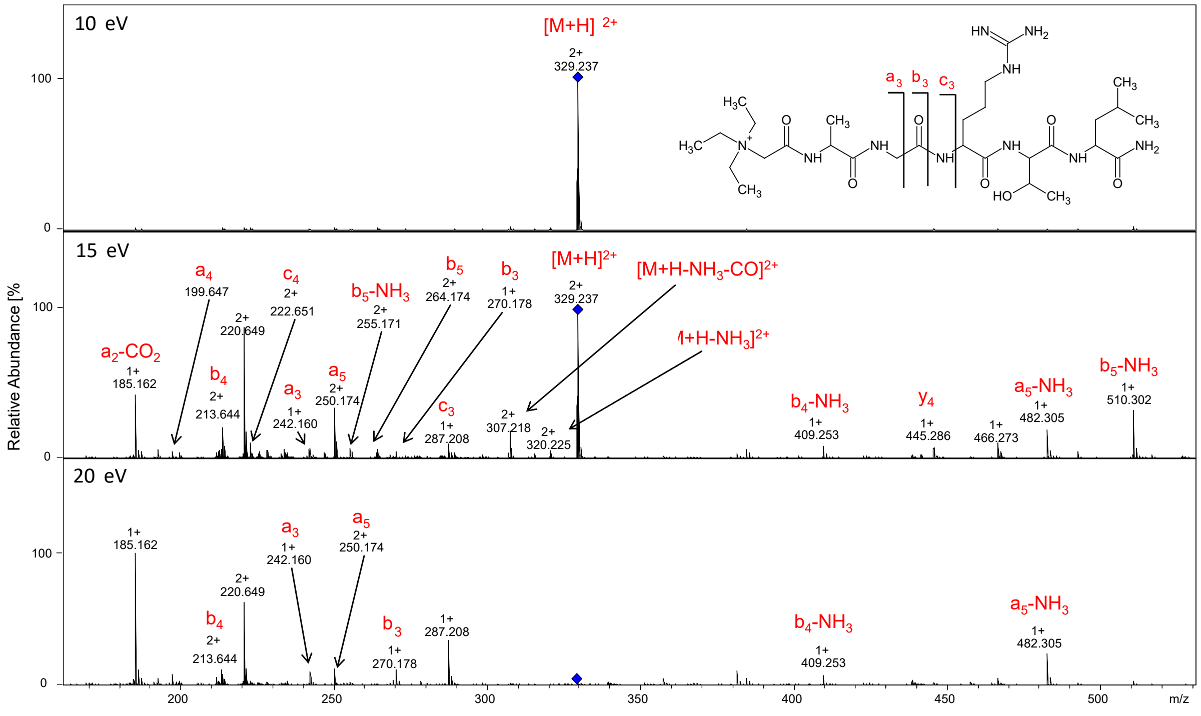

Figure S22. ESI-CID-MS/MS spectra of TEA⁺-CH2CO-Ala-Gly-Arg-Thr-Leu-NH2 (4a). Precursor ion at *m*/*z* 329.237.

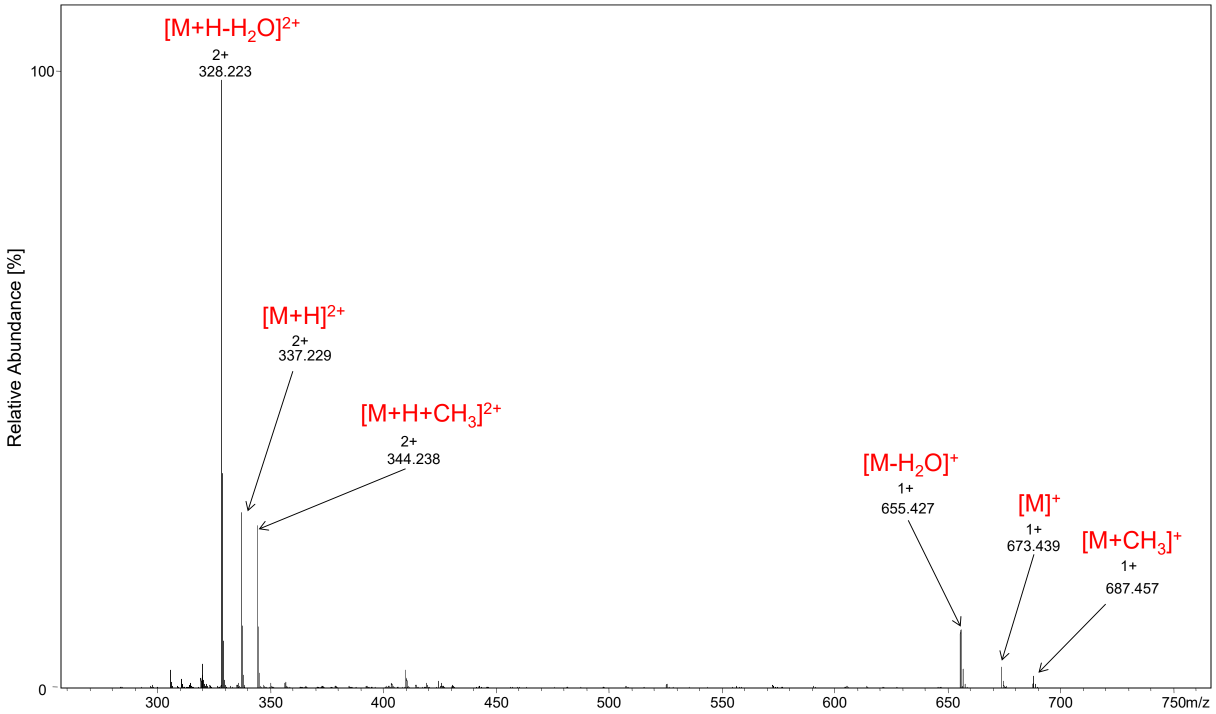

Figure S23. ESI-MS spectrum of TEA⁺-CH_2CO-Asp-Gly-Lys-Thr-Leu-NH_2 (5a)

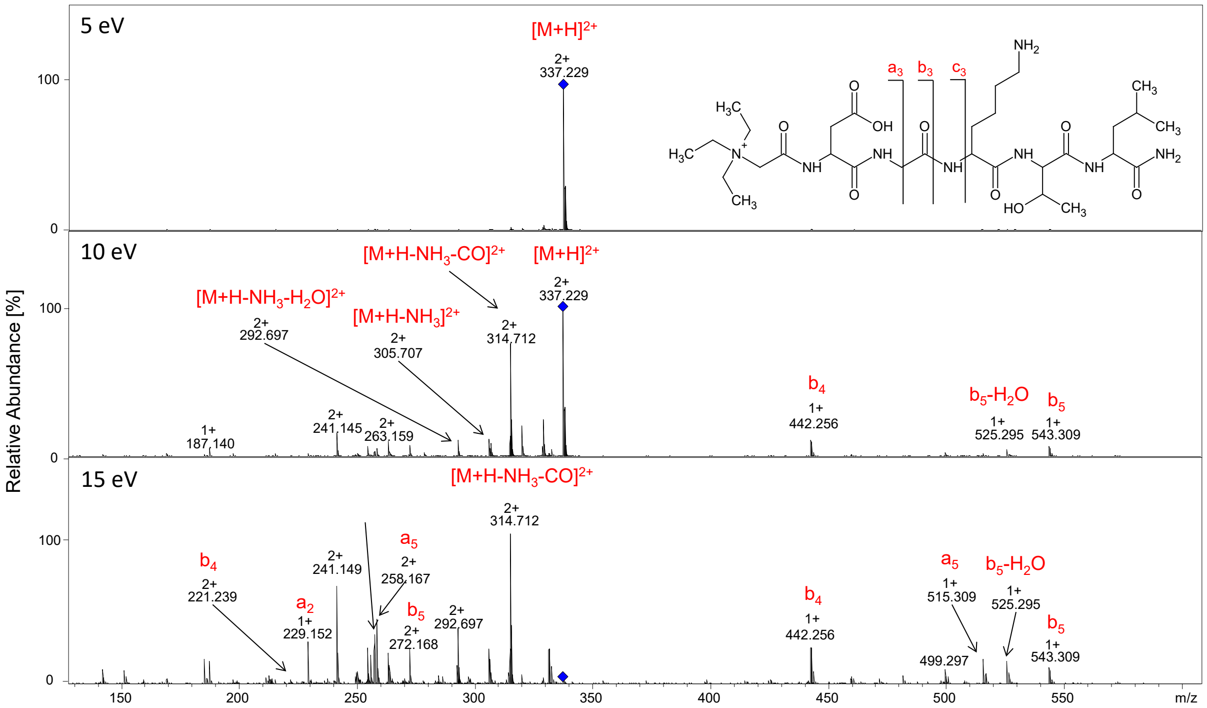

Figure S24. ESI-CID-MS/MS spectra of TEA⁺-CH_2CO-Asp-Gly-Lys-Thr-Leu-NH_2 (5a). Precursor ion at *m/z* 337.229.

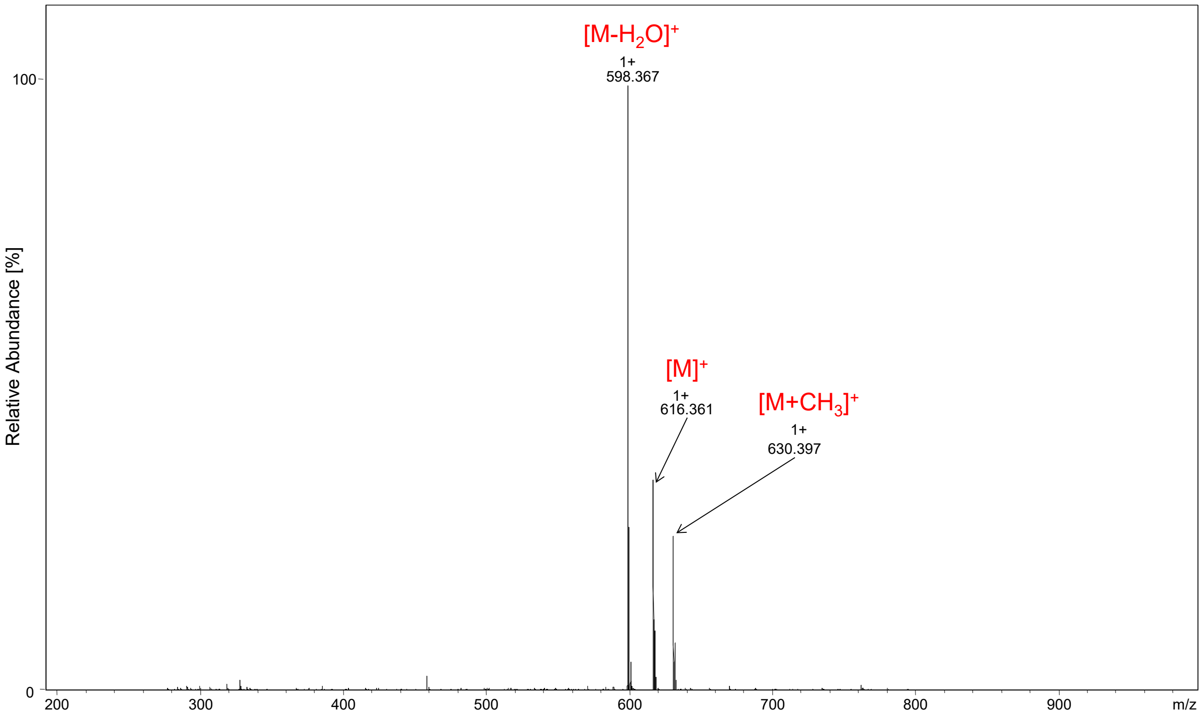

Figure S25. ESI-MS spectrum of TEA⁺-CH₂CO-Asp-Gly-Ala-Thr-Leu-NH₂ (6a)

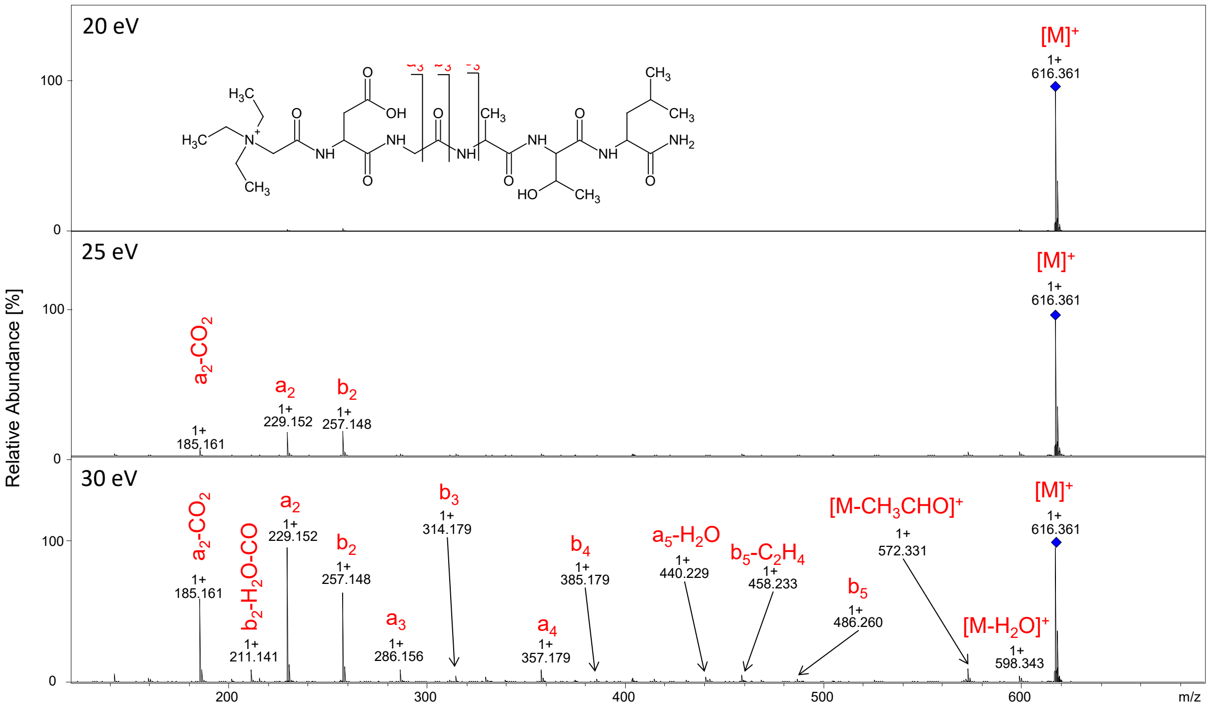

Figure S26. ESI-CID-MS/MS spectra of TEA⁺-CH₂CO-Asp-Gly-Ala-Thr-Leu-NH₂ (6a). Precursor ion at *m*/*z* 616.361.

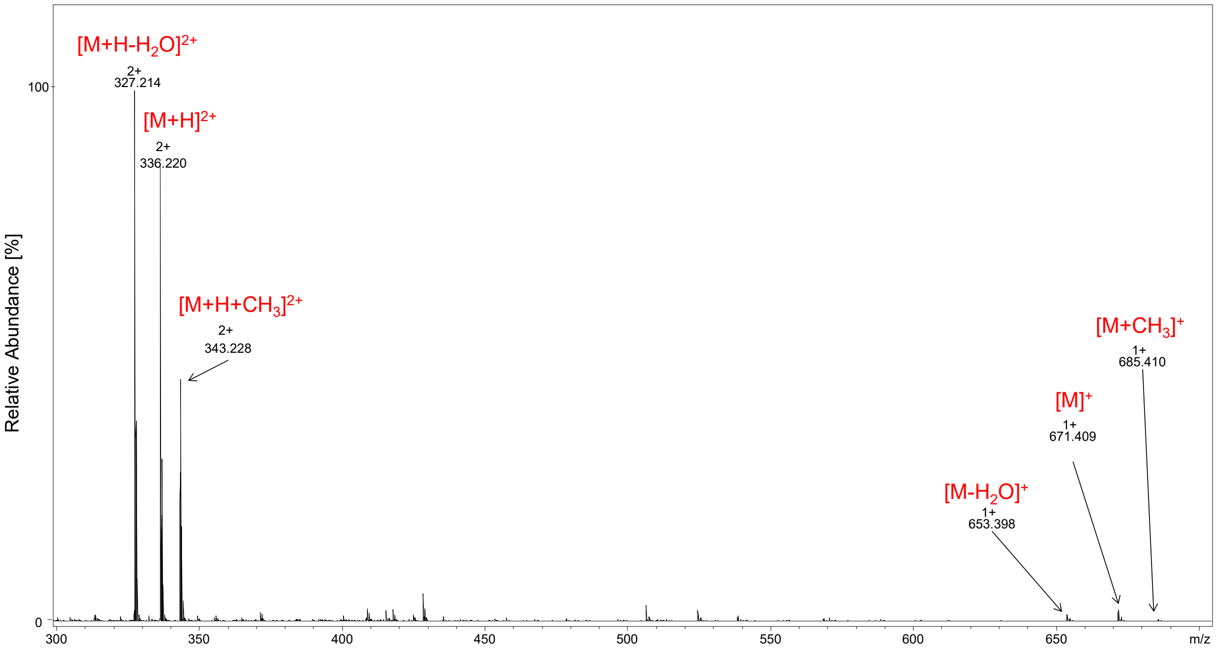

Figure S27. ESI-MS spectrum of TEA⁺-CH₂CO-Asp-Gly-Arg-Ala-Leu-NH₂ (7a)

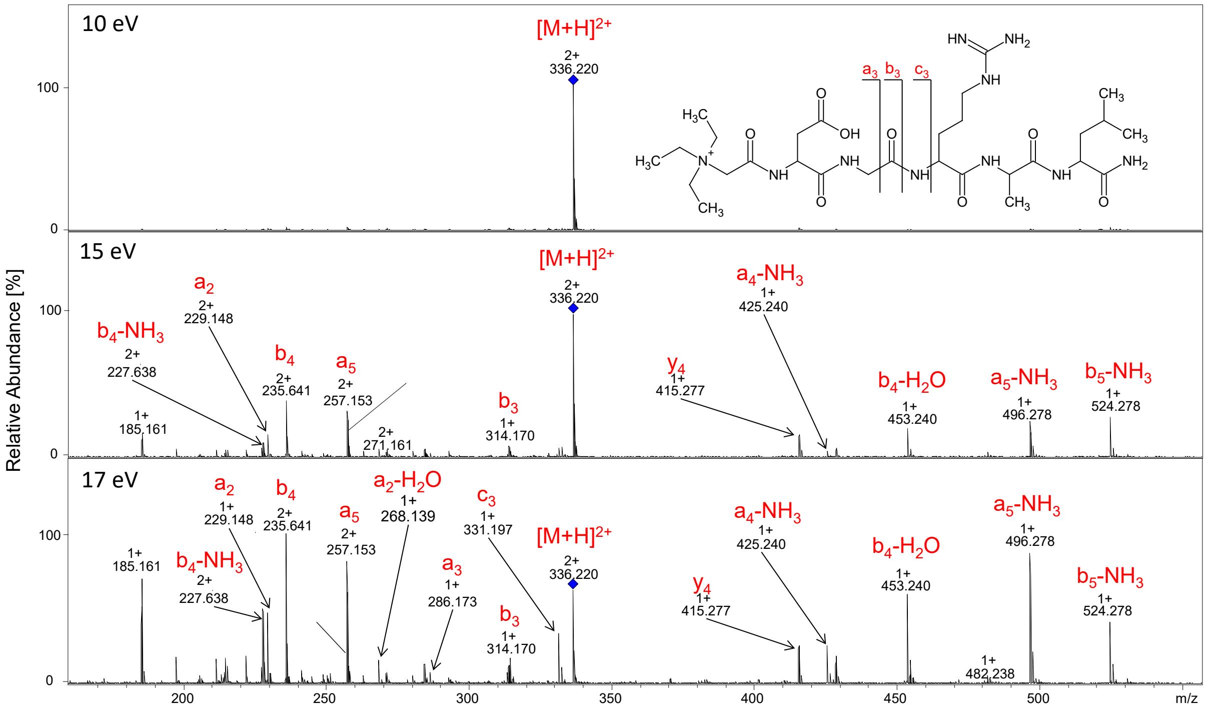

Figure S28. ESI-CID-MS/MS spectra of TEA⁺-CH₂CO-Asp-Gly-Arg-Ala-Leu-NH₂ (7a). Precursor ion at *m/z* 336.221.

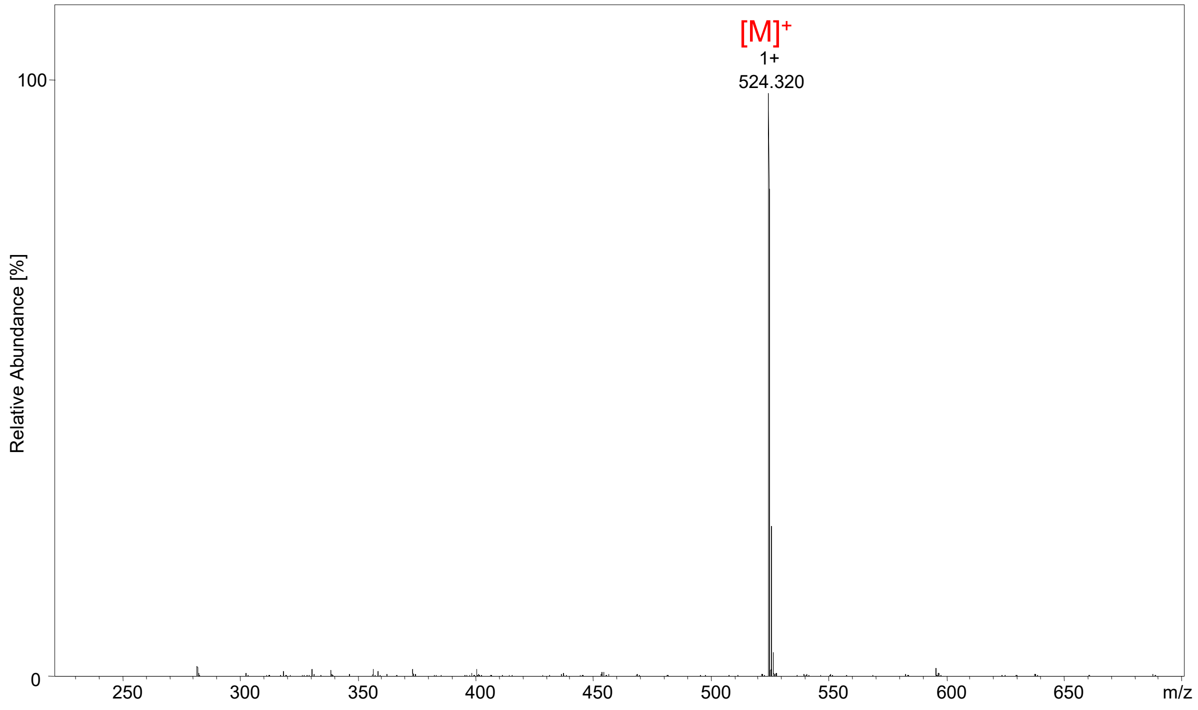

Figure S29. ESI-MS spectrum of ABCO⁺-CH2CO-Ala-Ala-Ala-Ala-Ala-NH2 (1b)

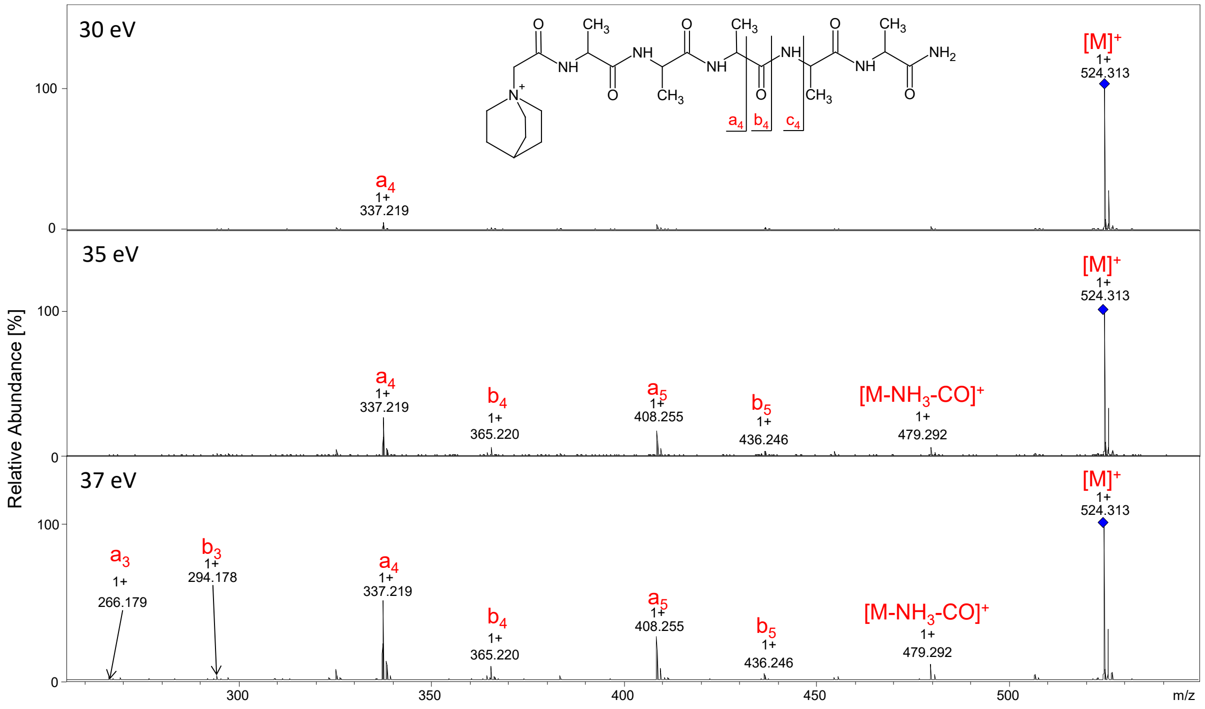

Figure S30. ESI-CID-MS/MS spectra of ABCO⁺-CH2CO-Ala-Ala-Ala-Ala-Ala-NH2 (1b). Precursor ion at *m/z* 524.313.

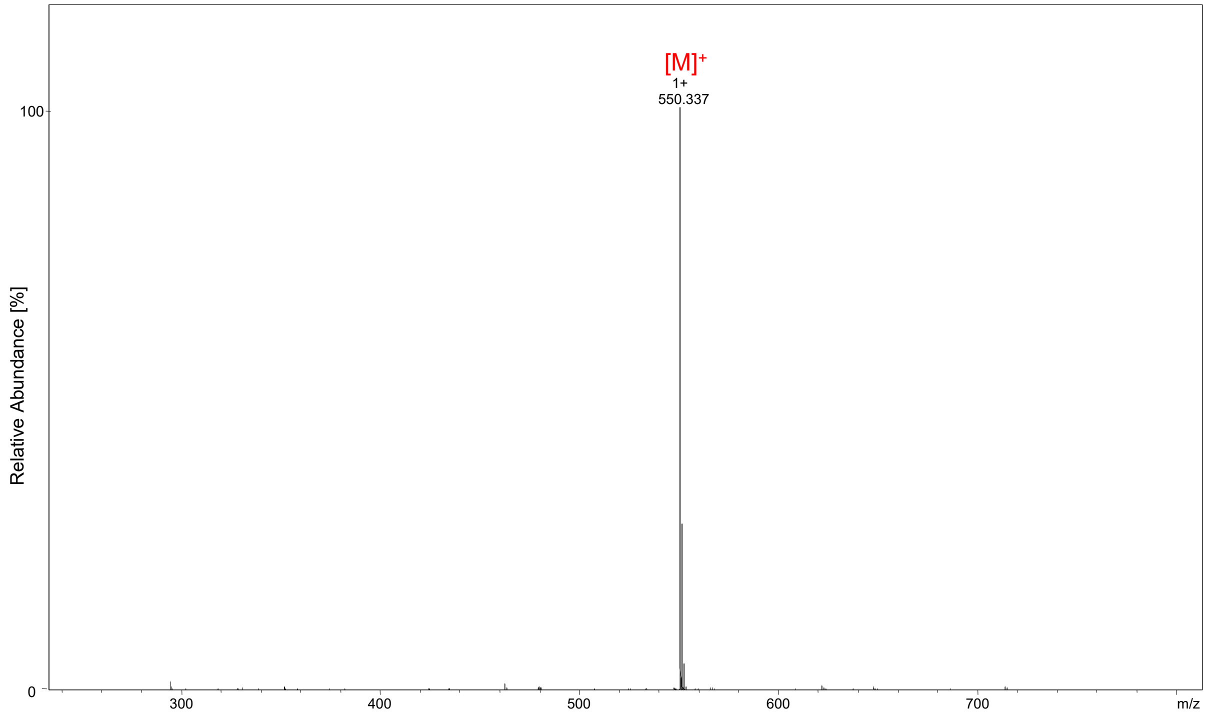

Figure S31. ESI-MS spectrum of ABCO$^+$-CH$_2$CO-Ala-Ala-Pro-Ala-Ala-NH$_2$ (2b)

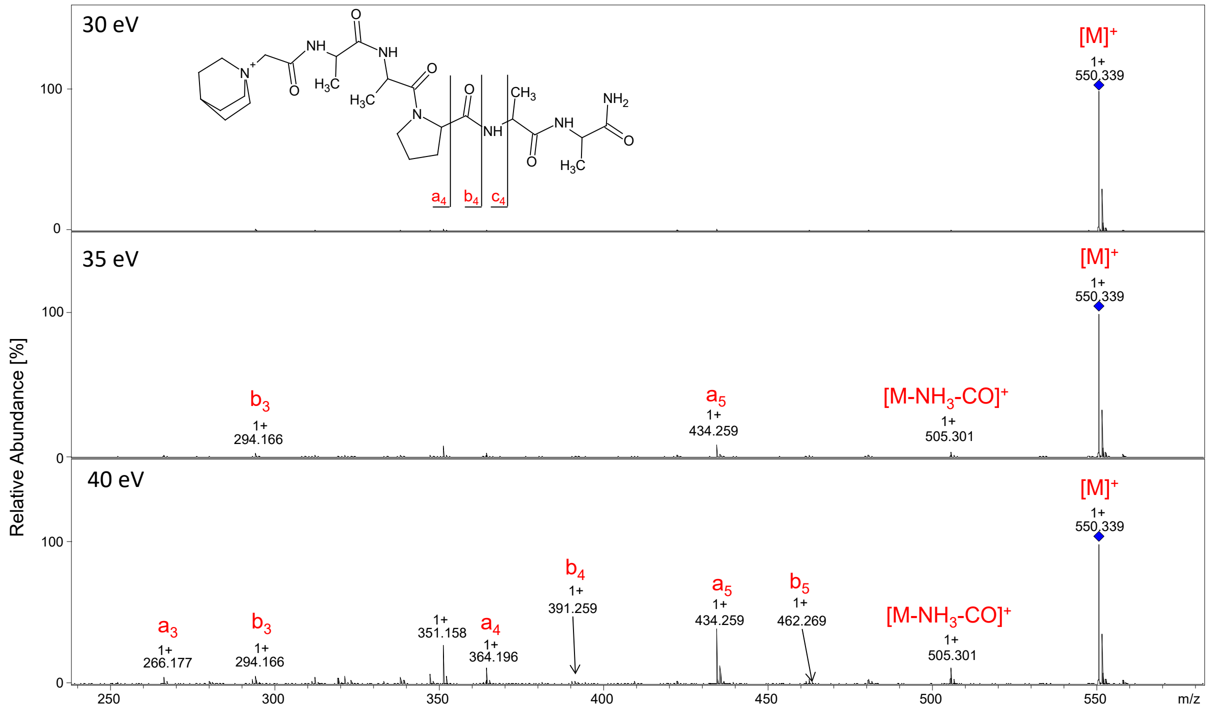

Figure S32. ESI-CID-MS/MS spectra of ABCO$^+$-CH$_2$CO-Ala-Ala-Pro-Ala-Ala-NH$_2$ (2b). Precursor ion at *m*/*z* 550.339.

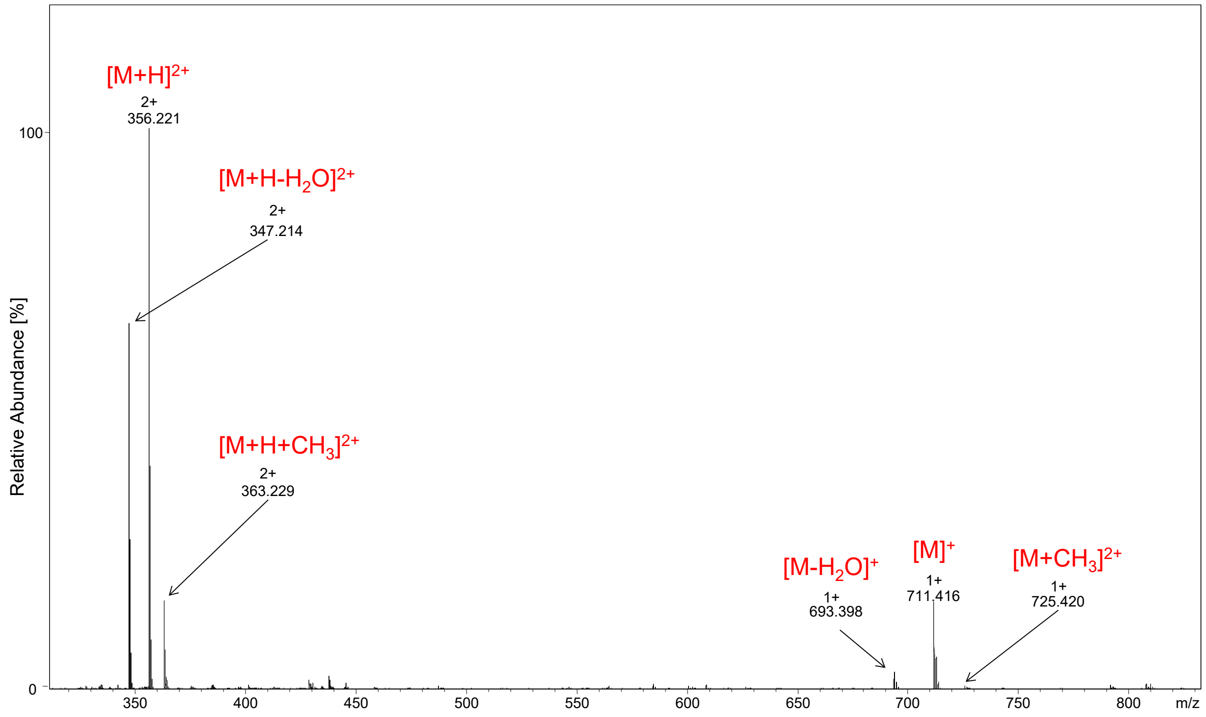

Figure S33. ESI-MS spectrum of ABCO⁺-CH₂CO-Asp-Gly-Arg-Thr-Leu-NH₂ (3b)

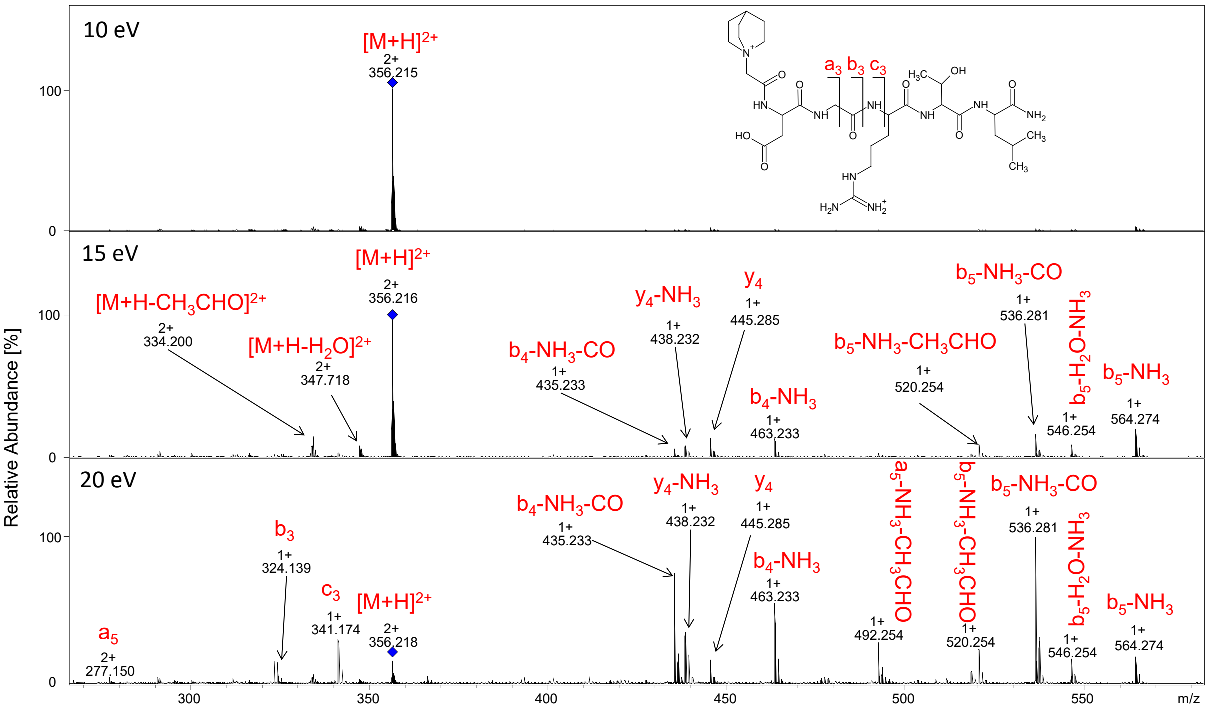

Figure S34. ESI-CID-MS/MS spectra of ABCO⁺-CH₂CO-Asp-Gly-Arg-Thr-Leu-NH₂ (3b). Precursor ion at *m/z* 356.218.

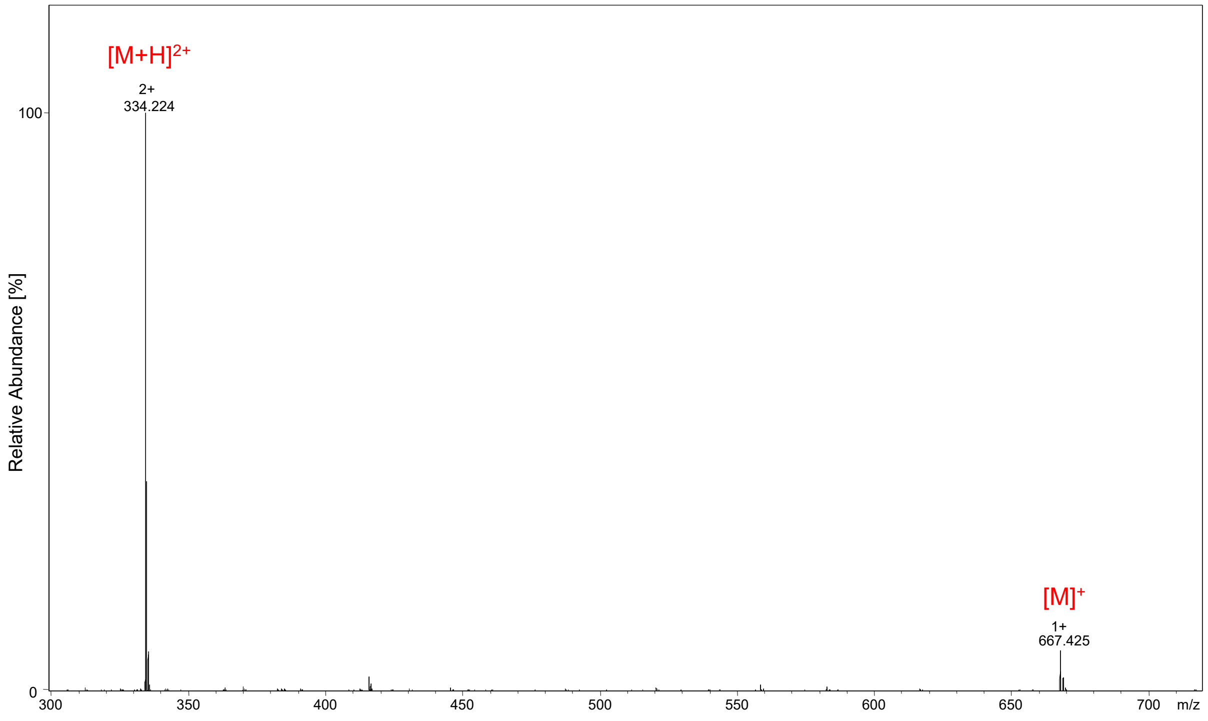

Figure S35. ESI-MS spectrum of ABCO⁺-CH₂CO-Ala-Gly-Arg-Thr-Leu-NH₂ (4b)

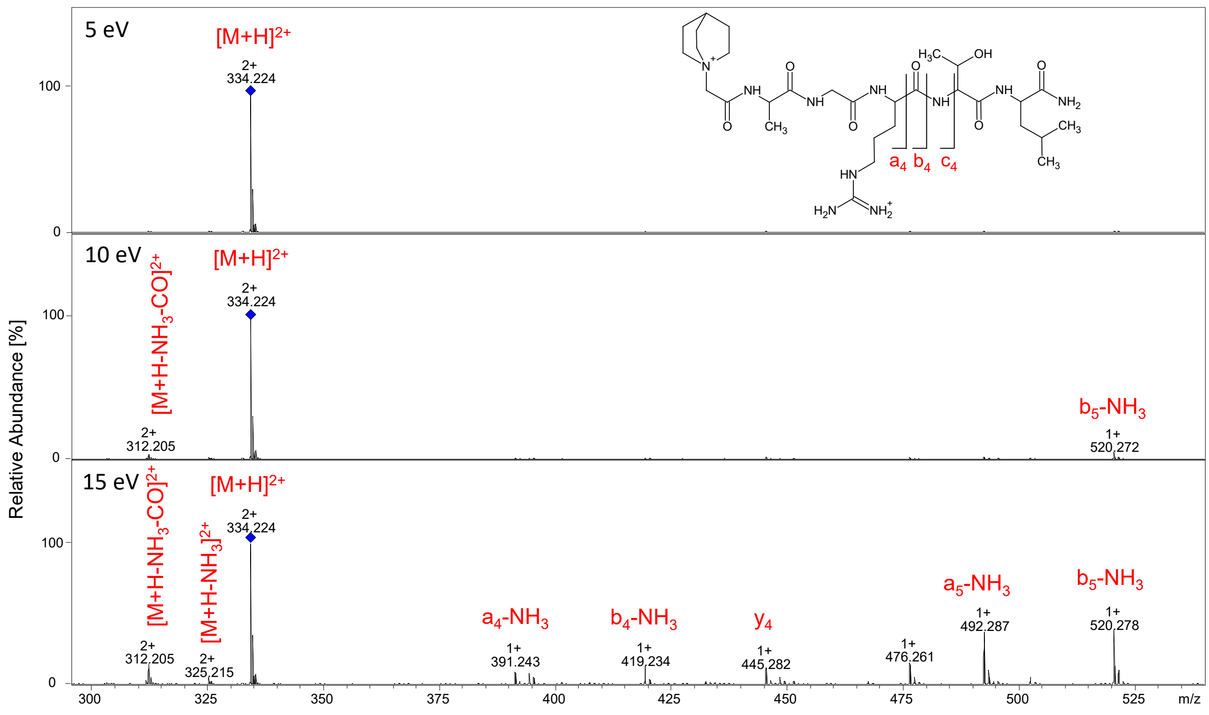

Figure S36. ESI-CID-MS/MS spectra of ABCO⁺-CH₂CO-Ala-Gly-Arg-Thr-Leu-NH₂ (4b). Precursor ion at *m/z* 334.224.

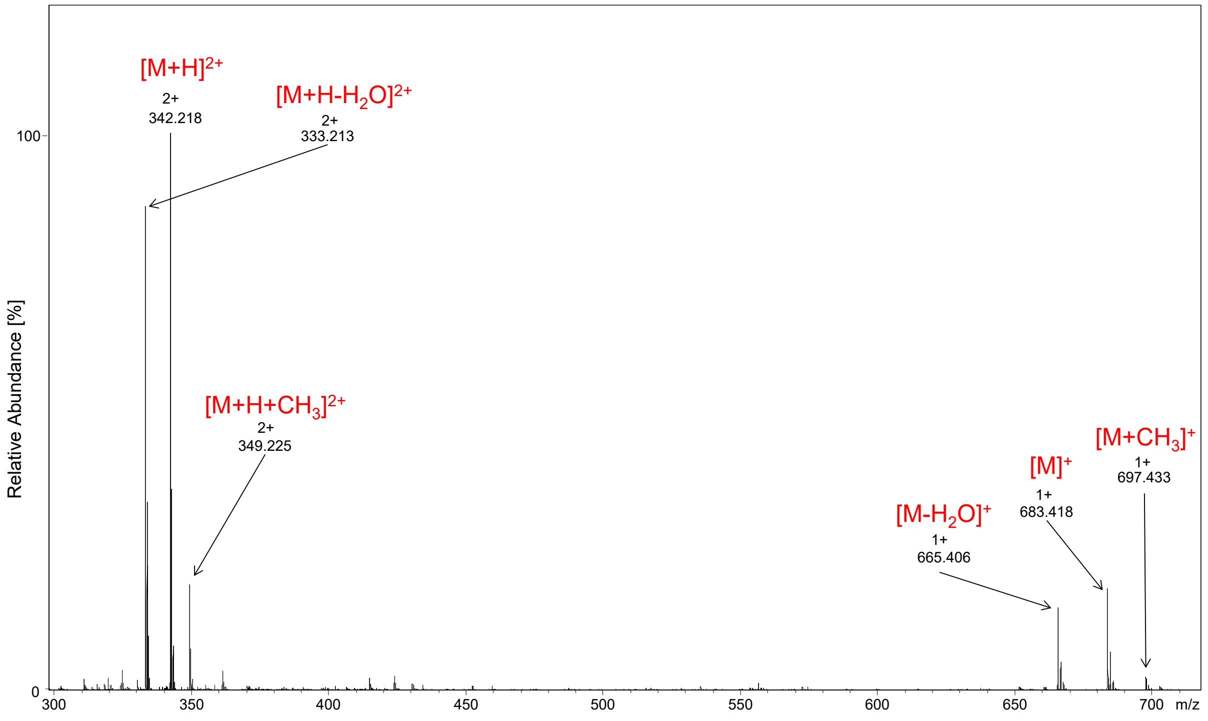

Figure S37. ESI-MS spectrum of ABCO⁺-CH2CO-Asp-Gly-Lys-Thr-Leu-NH2 (5b)

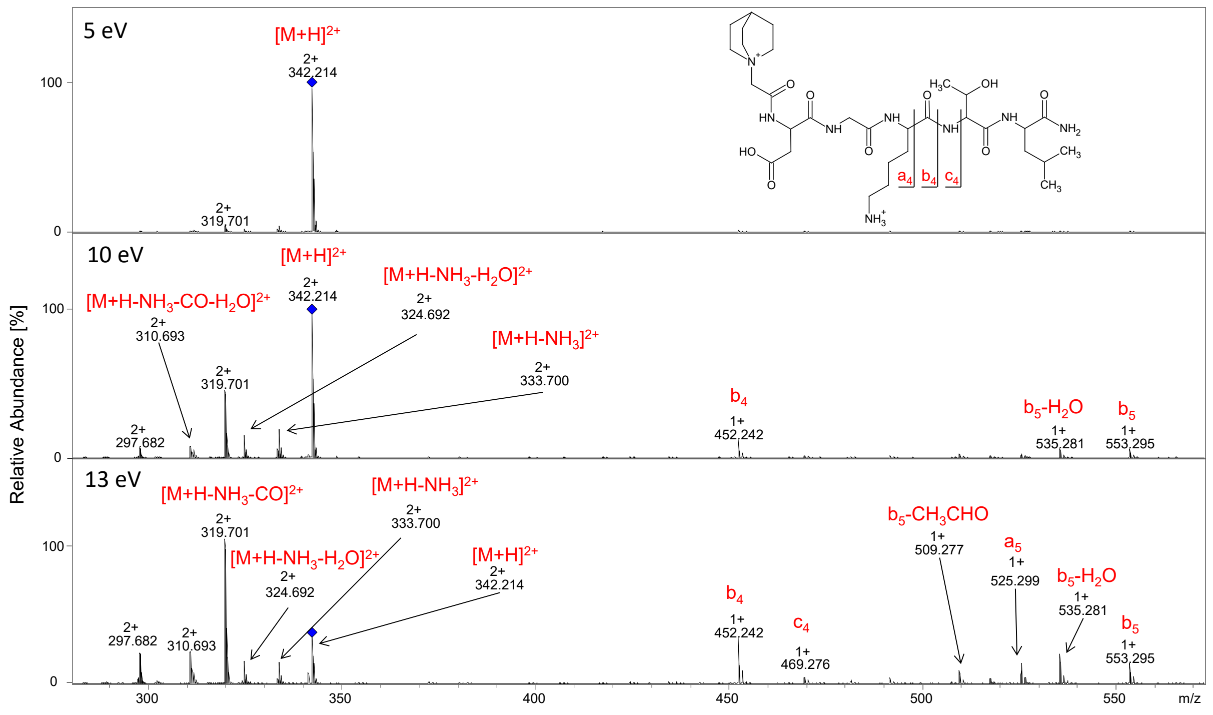

Figure S38. ESI-CID-MS/MS spectra of ABCO⁺-CH2CO-Asp-Gly-Lys-Thr-Leu-NH2 (5b). Precursor ion at *m/z* 342.214.

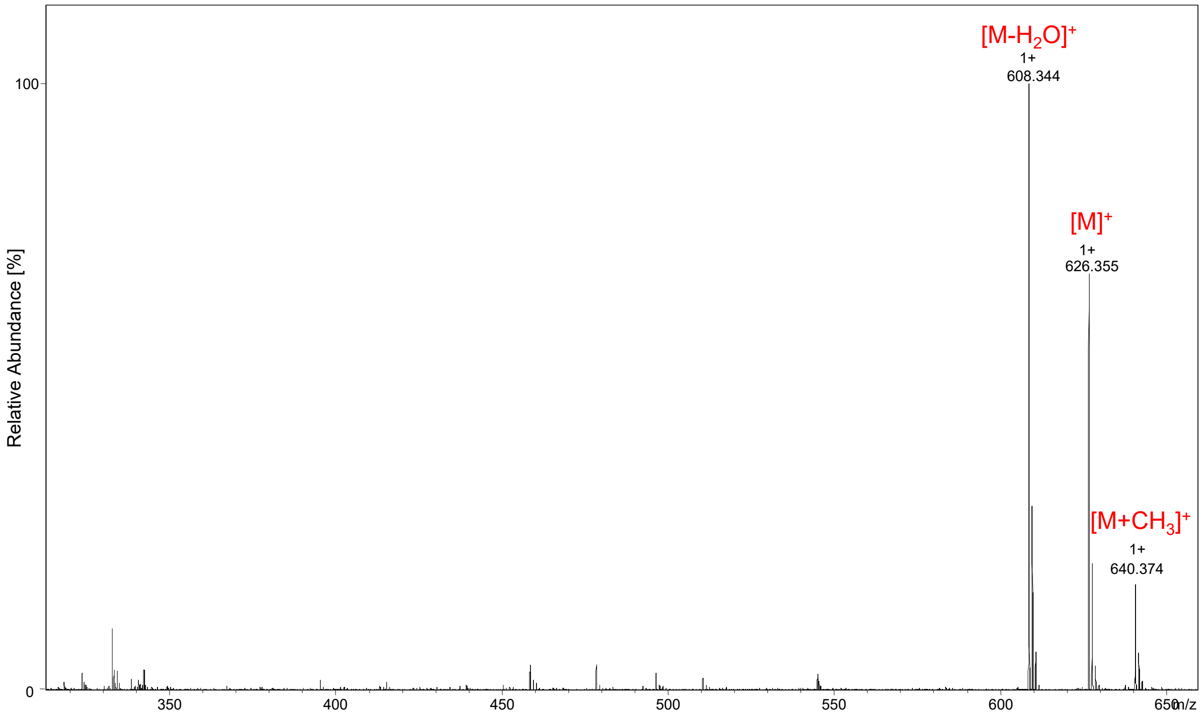

Figure S39. ESI-MS spectrum of ABCO$^+$-CH_2CO-Asp-Gly-Ala-Thr-Leu-NH_2 (6b)

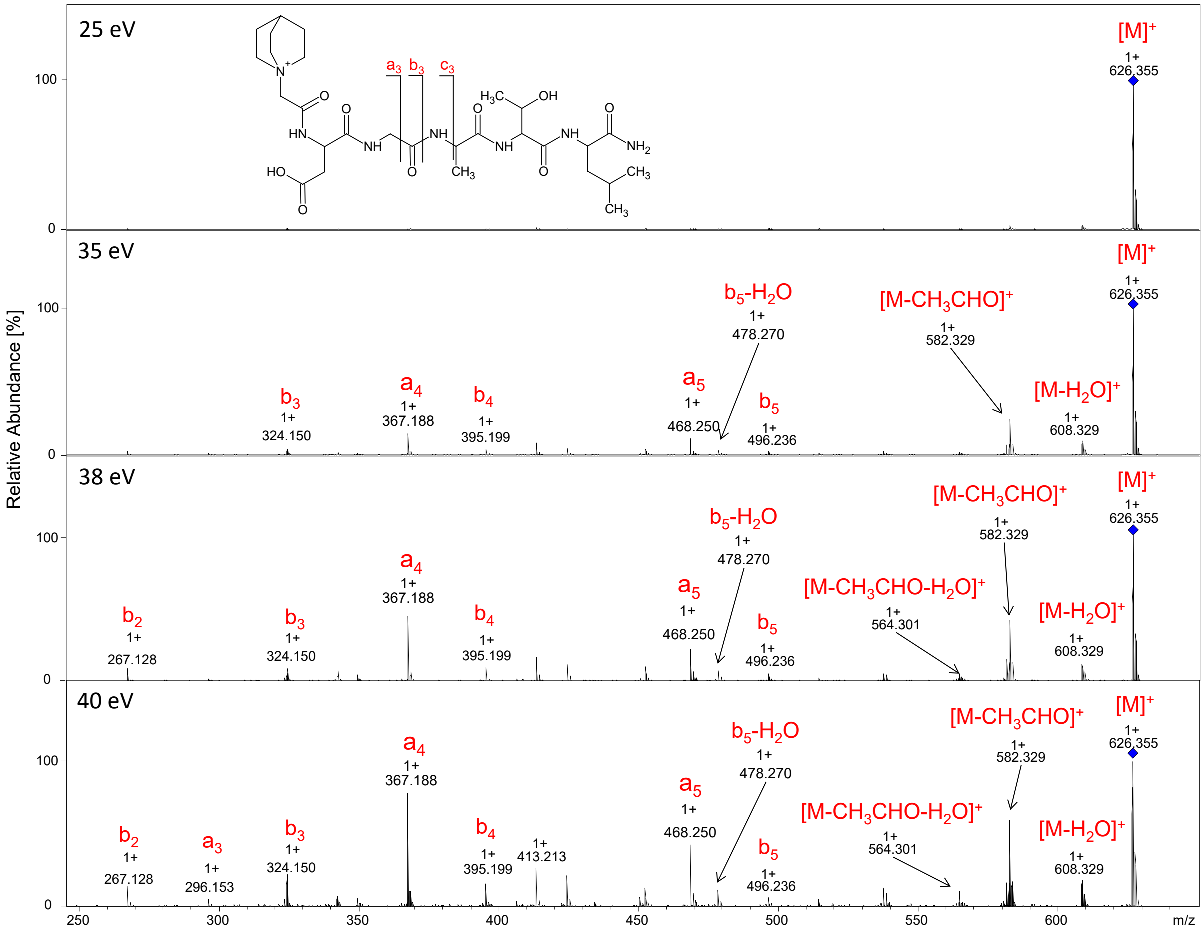

Figure S40. ESI-CID-MS/MS spectra of ABCO+-CH2CO-Asp-Gly-Ala-Thr-Leu-NH2 (6b). Precursor ion at *m*/*z* 626.355.

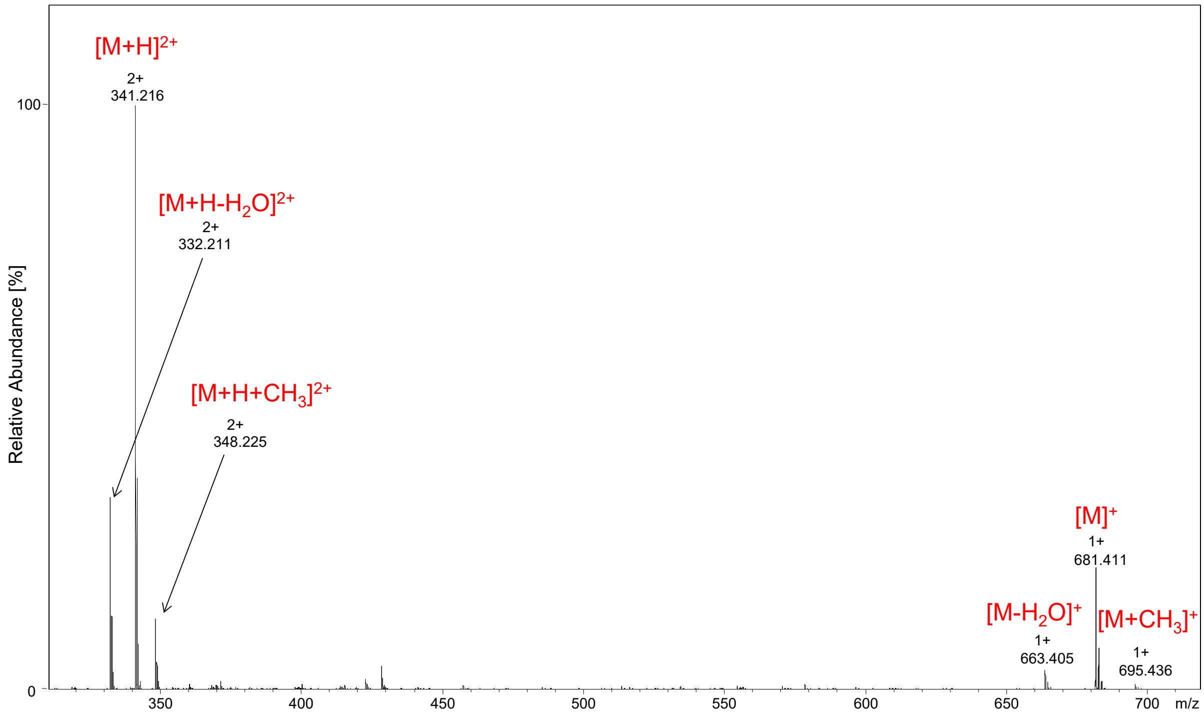

Figure S41. ESI-MS spectrum ABCO+-CH2CO-Asp-Gly-Arg-Ala-Leu-NH2 (7b)

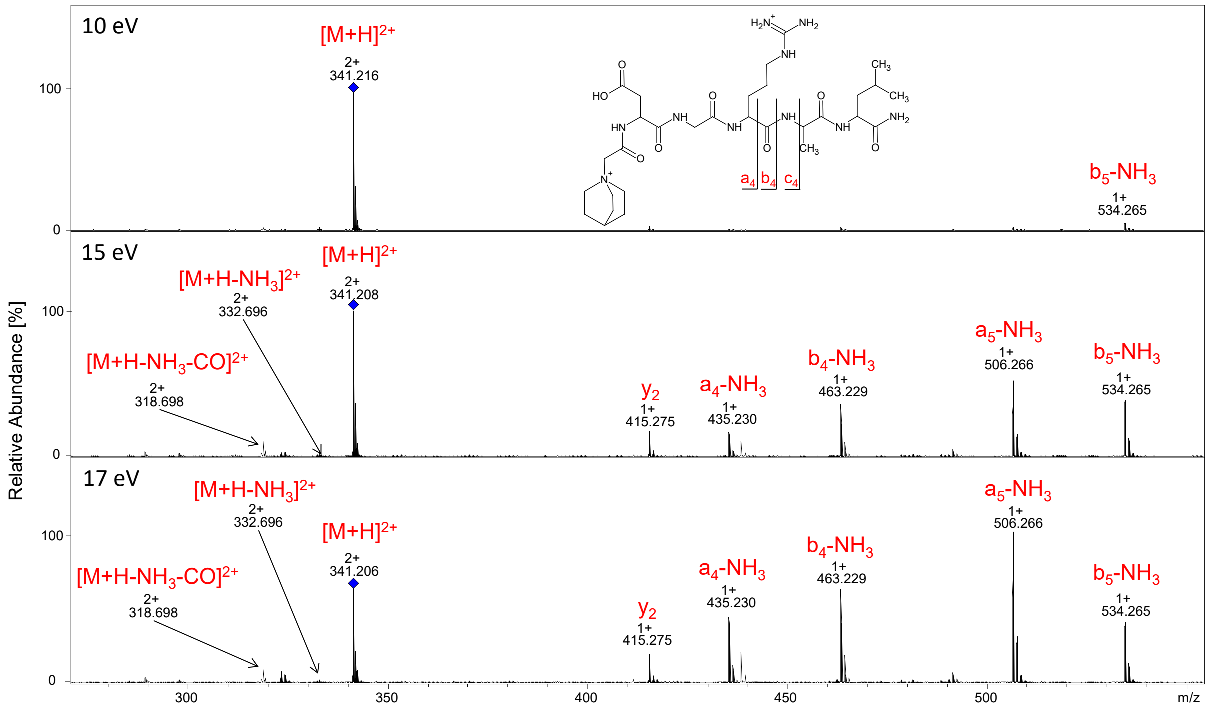

Figure S42. ESI-CIDMS/MS spectra of ABCO+-CH2CO-Asp-Gly-Arg-Ala-Leu-NH2 (7b). Precursor ion at *m/z* 341.206.

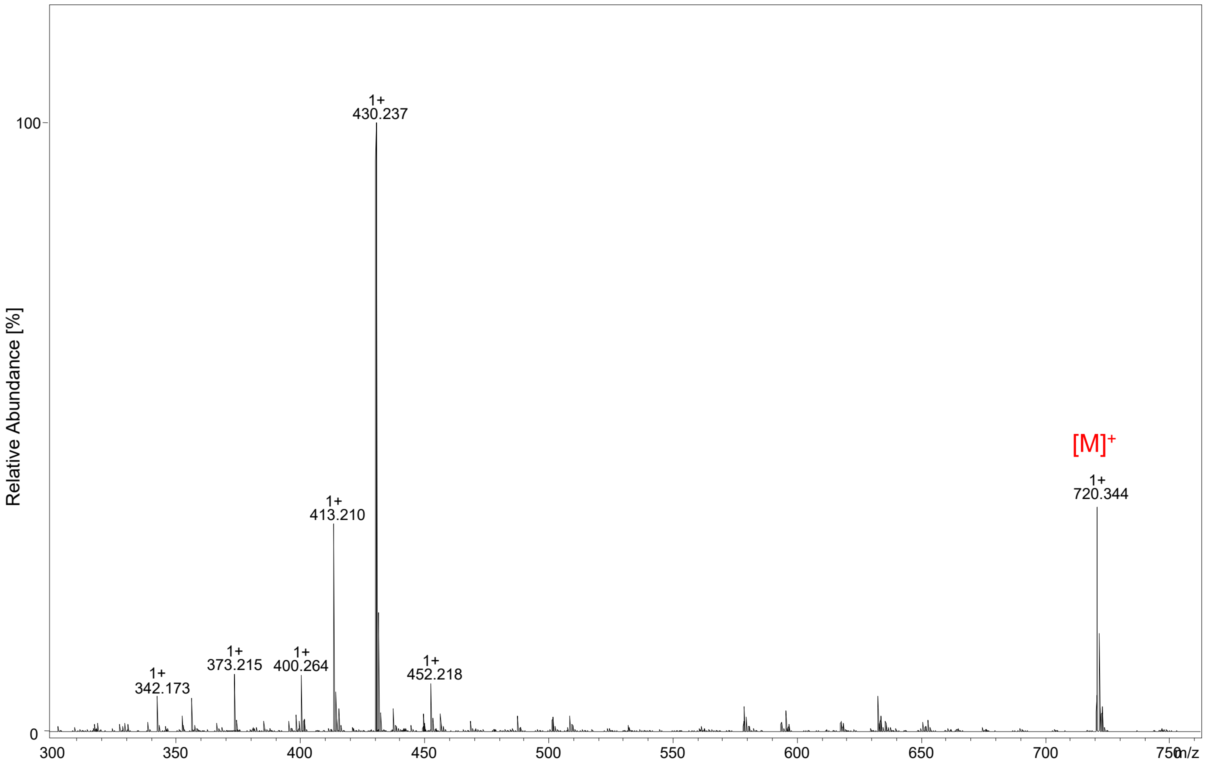

Figure S43. ESI-MS spectrum of TPP⁺-CH_2CO-Ala-Ala-Ala-Ala-Ala-NH_2 (1c)

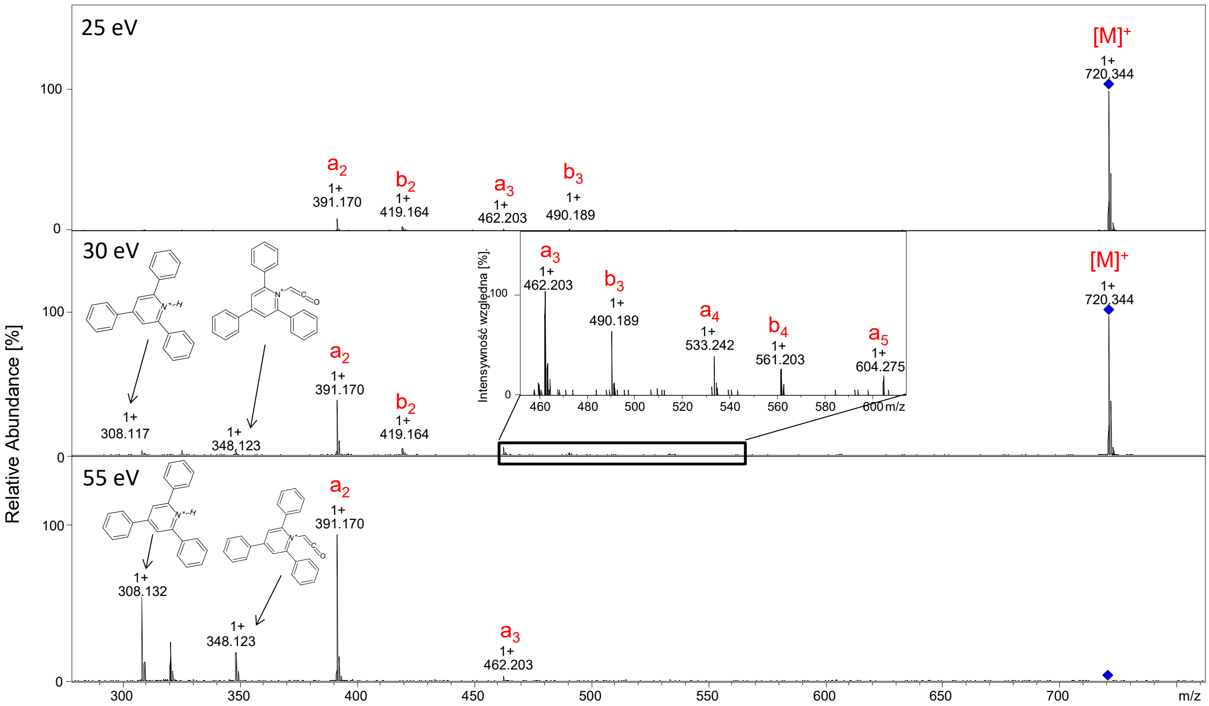

Figure S44. ESI-CID-MS/MS spectra of TPP⁺-CH_2CO-Ala-Ala-Ala-Ala-Ala-NH_2 (1c). Precursor ion at *m/z* 720.344.

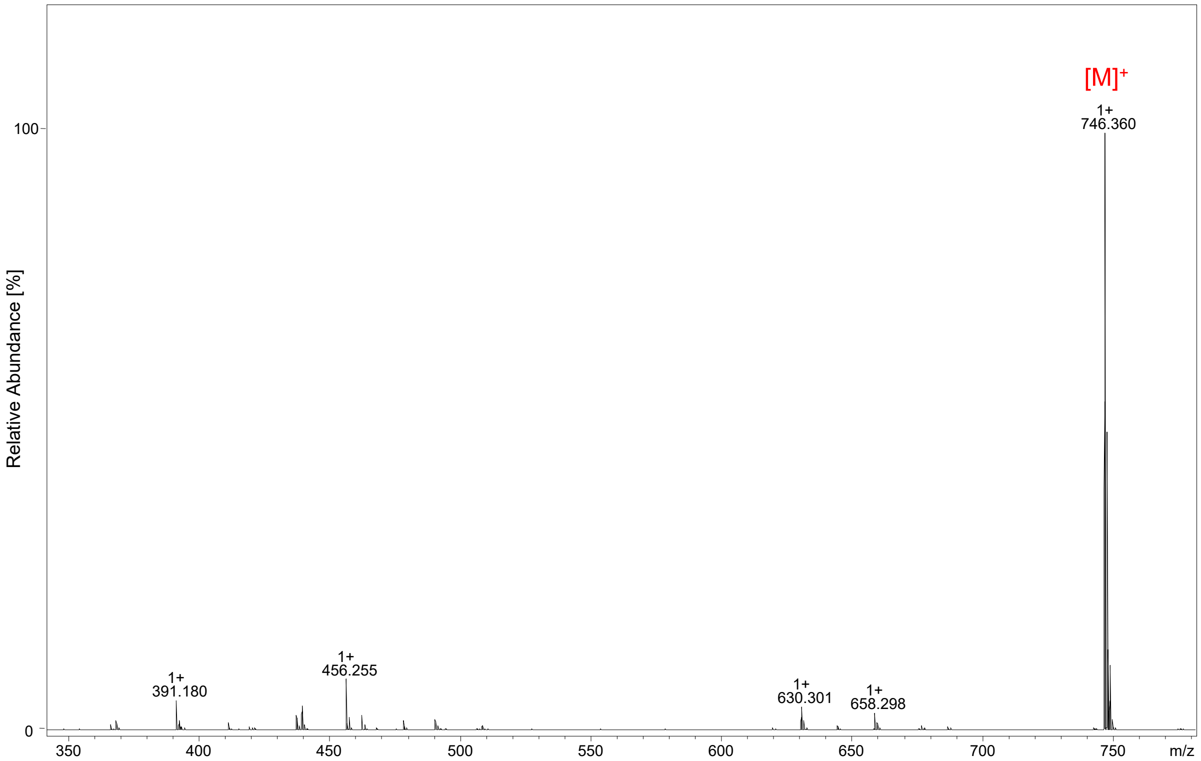

Figure S45. ESI-MS spectrum of TPP+-CH2CO-Ala-Ala-Pro-Ala-Ala-NH2 (2c)

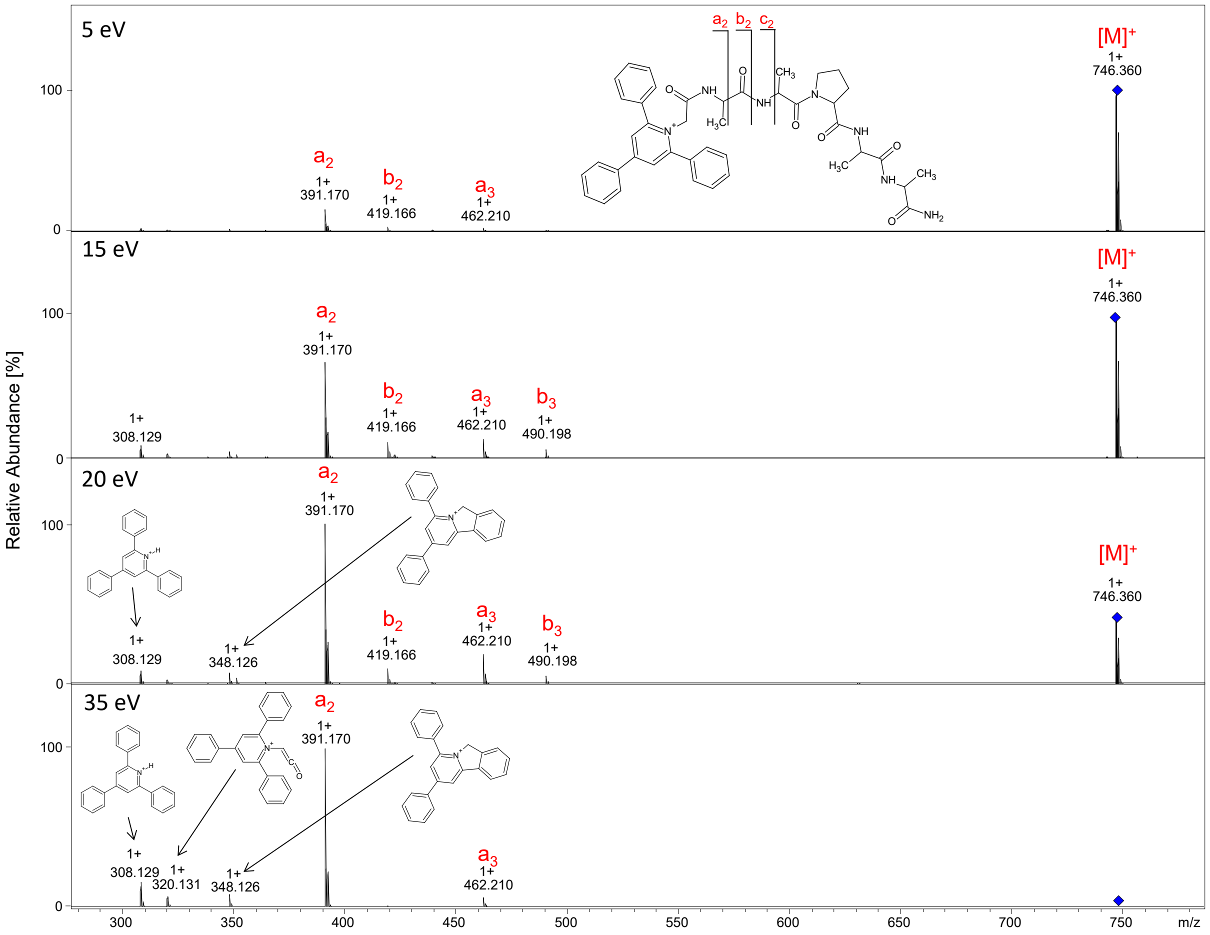

Figure S46. ESI-CID-MS/MS spectra of TPP+-CH2CO-Ala-Ala-Pro-Ala-Ala-NH2 (2c). Precursor ion at *m/z* 746.360.

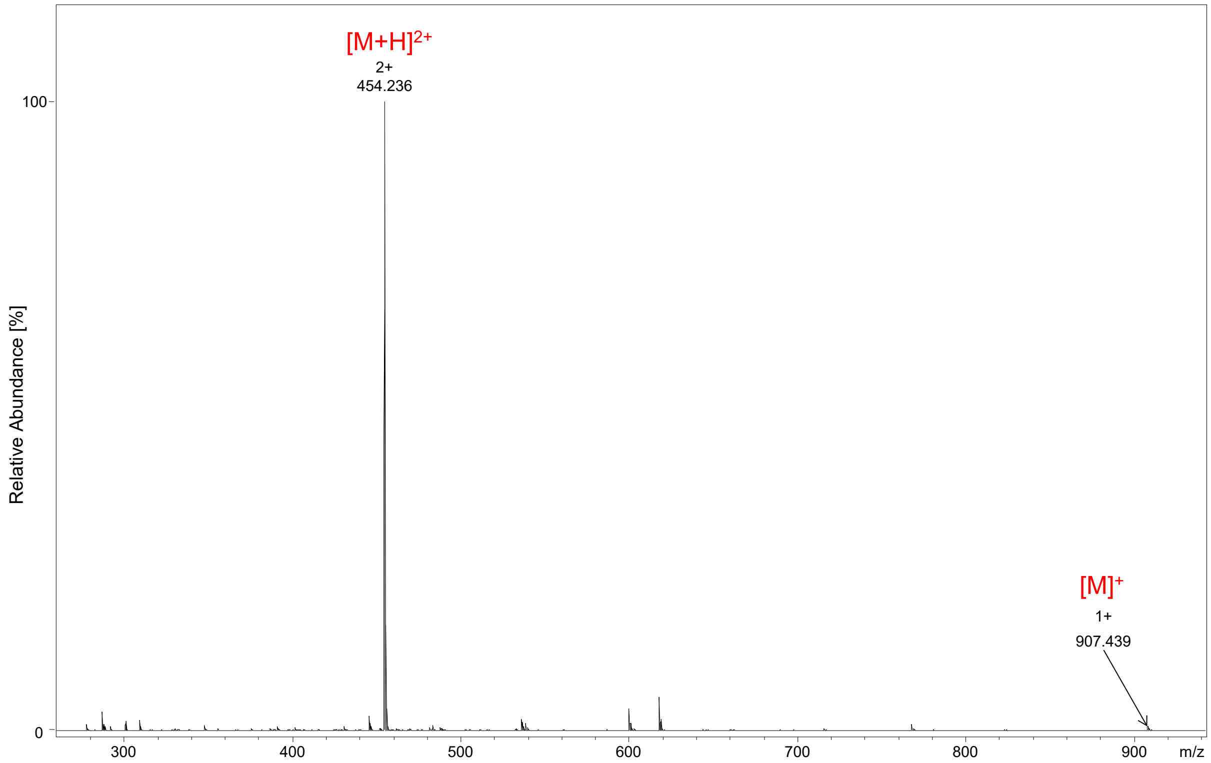

Figure S47. ESI-MS spectrum of TPP$^+$-CH_2CO-Asp-Gly-Arg-Thr-Leu-NH_2 (3c)

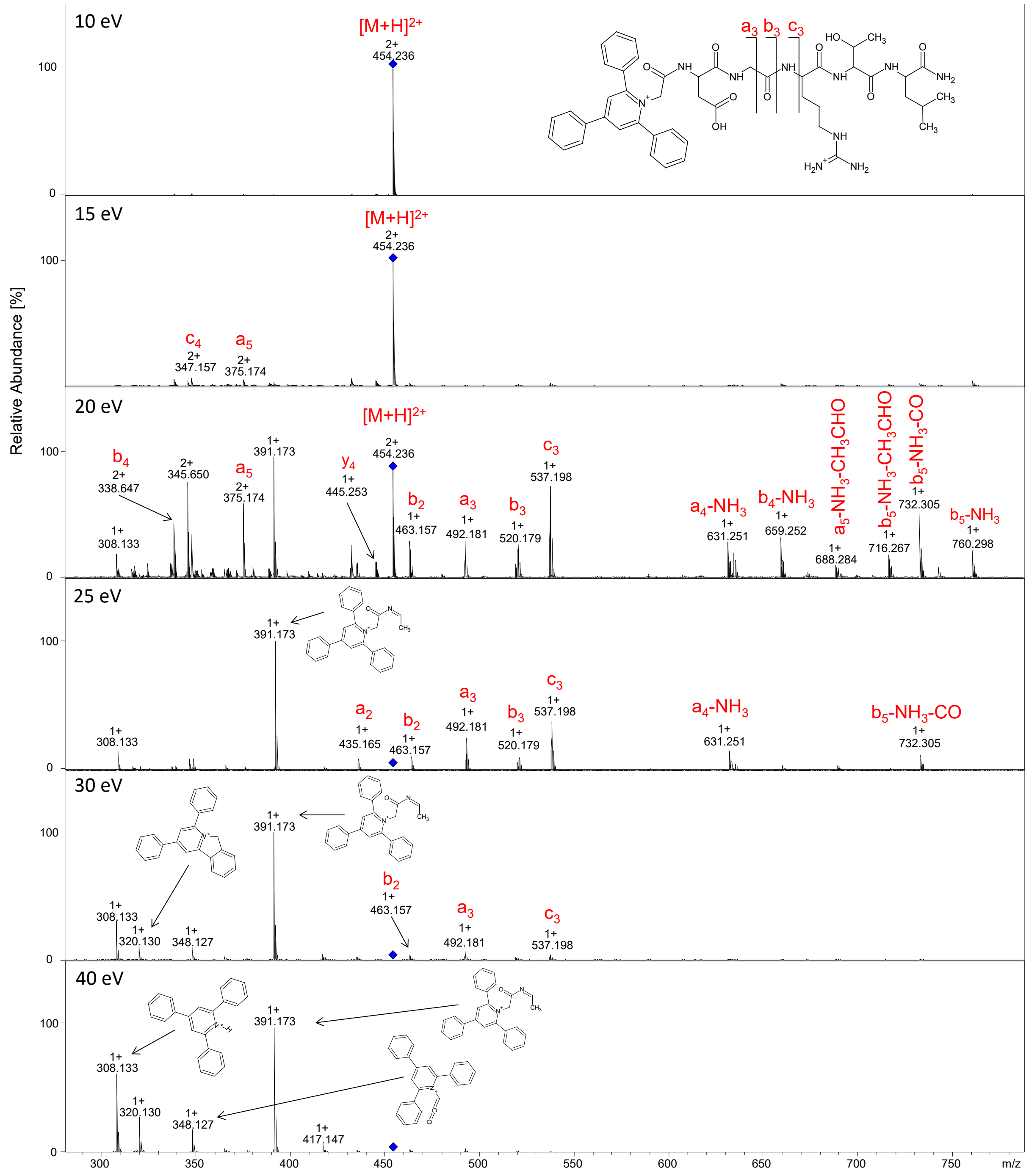

Figure S48. ESI-CID-MS/MS spectra of TPP⁺-CH₂CO-Asp-Gly-Arg-Thr-Leu-NH₂ (3c). Precursor ion at *m/z* 454.236.

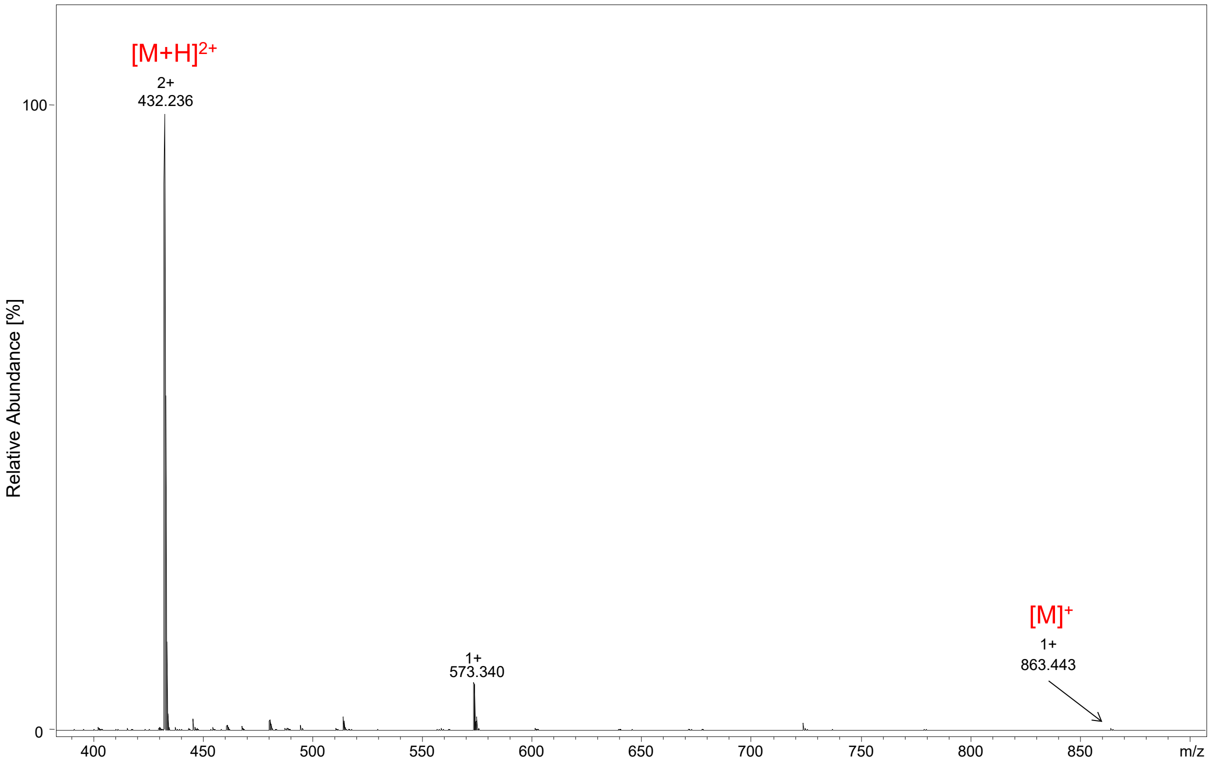

Figure S49. ESI-MS spectrum of TPP^{+}-CH_2CO-Ala-Gly-Arg-Thr-Leu-NH_2 (4c)

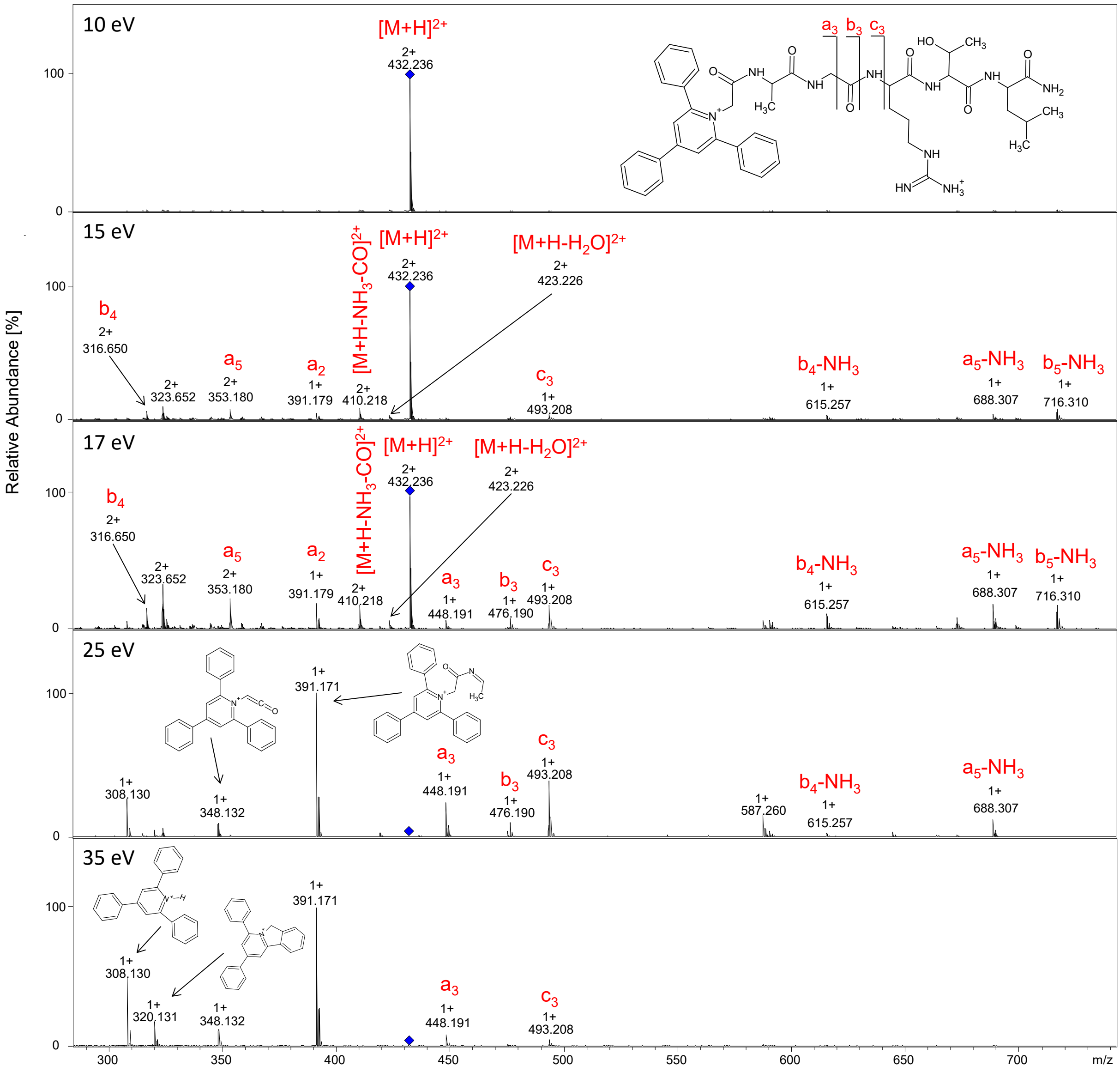

Figure S50. ESI-CID-MS/MS spectra of TPP+-CH2CO-Ala-Gly-Arg-Thr-Leu-NH2 (4c). Precursor ion at *m/z* 432.236.

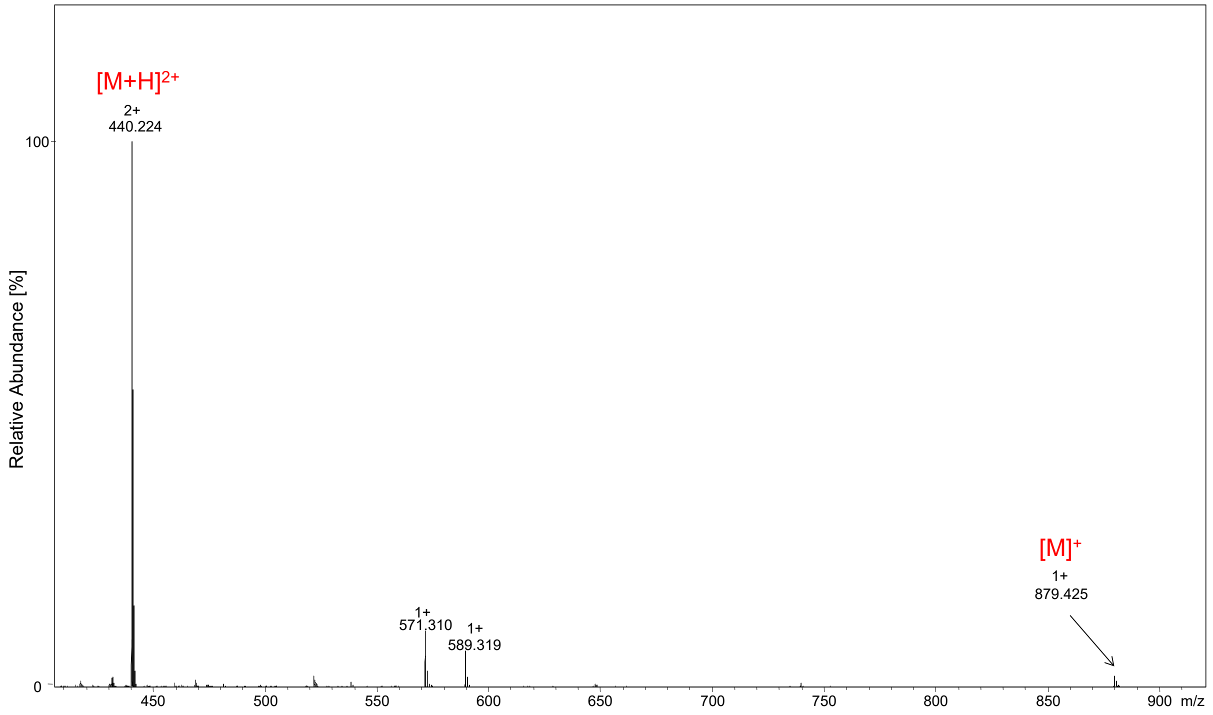

Figure S51. ESI-MS spectrum of TPP⁺-CH₂CO-Asp-Gly-Lys-Thr-Leu-NH₂ (5c)

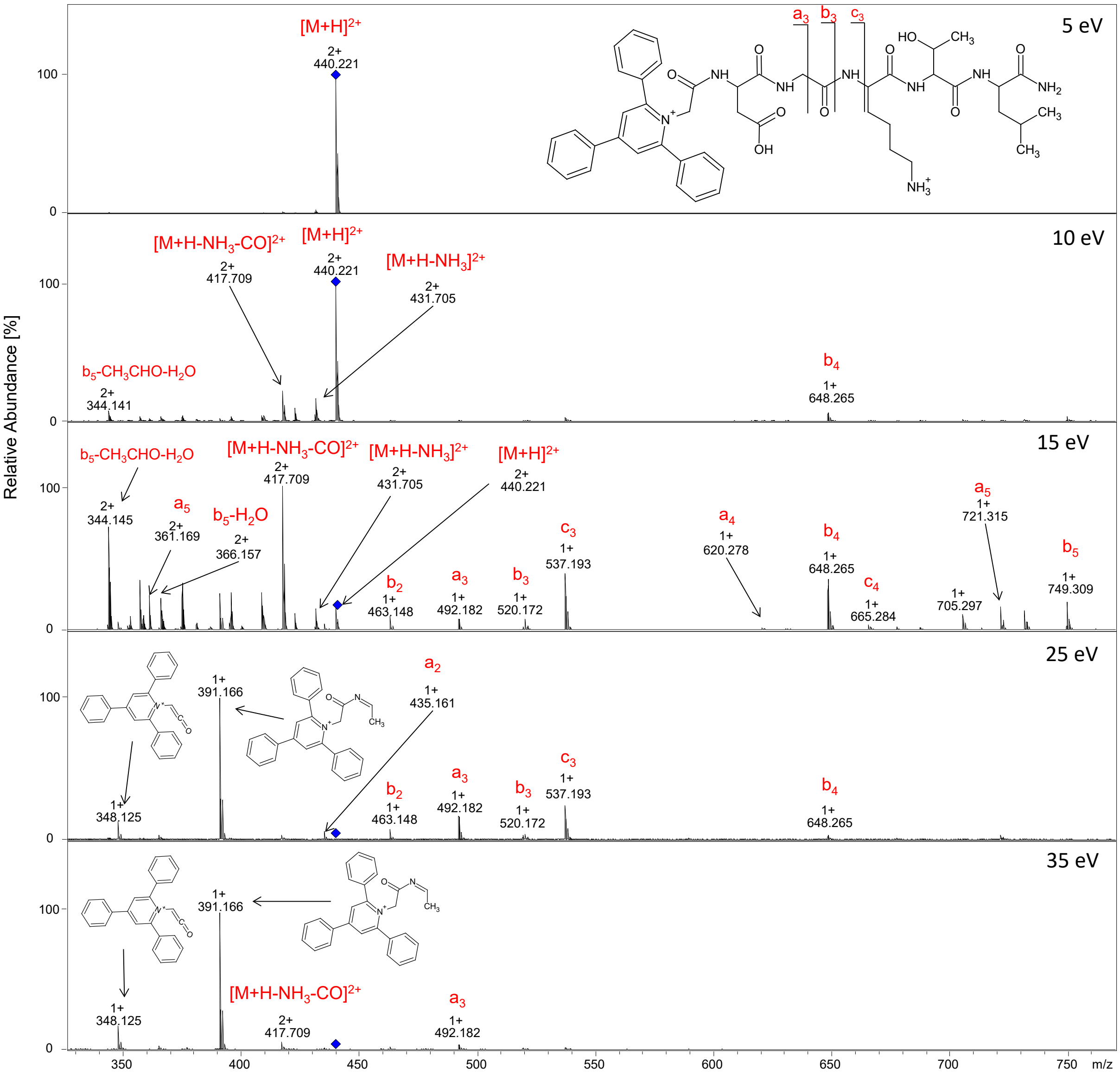

Figure S52. ESI-CID-MS/MS spectra of TPP⁺-CH₂CO-Asp-Gly-Lys-Thr-Leu-NH₂ (5c). Precursor ion at *m*/*z* 440.221.

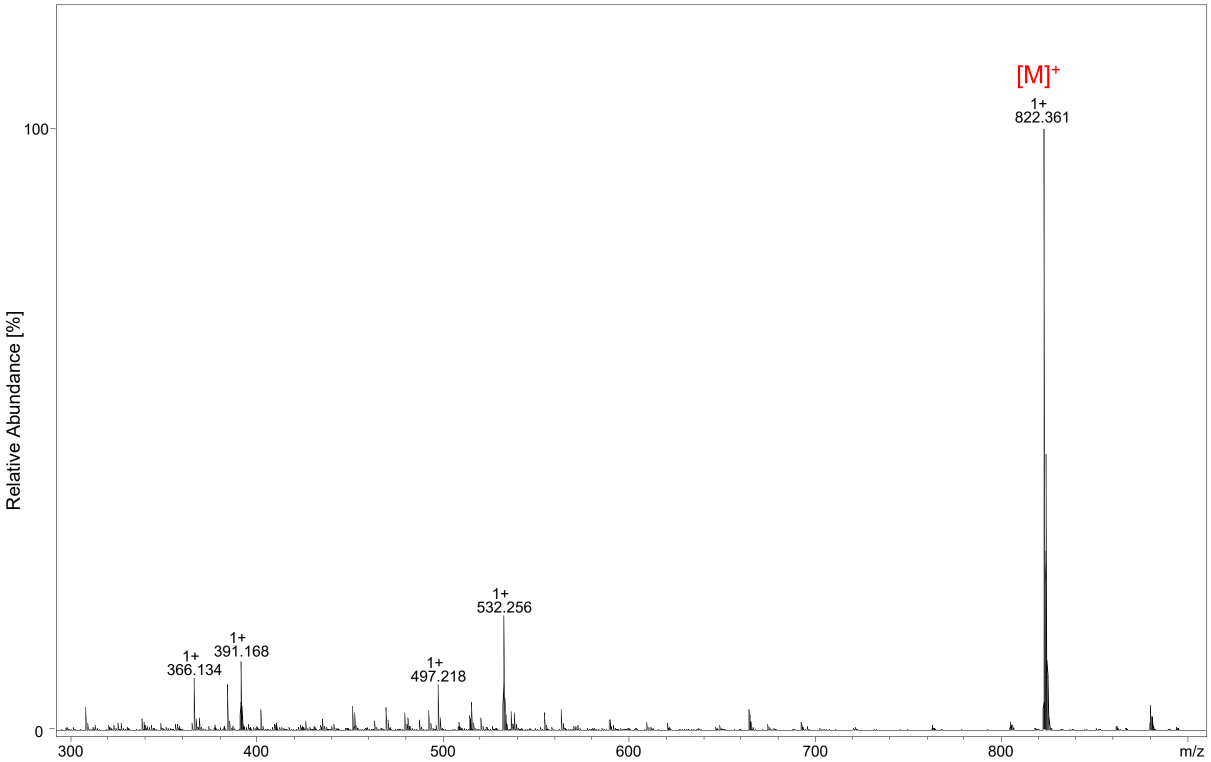

Figure S53. ESI-MS spectrum of TPP$^+$-CH_2CO-Asp-Gly-Ala-Thr-Leu-NH_2 (6c)

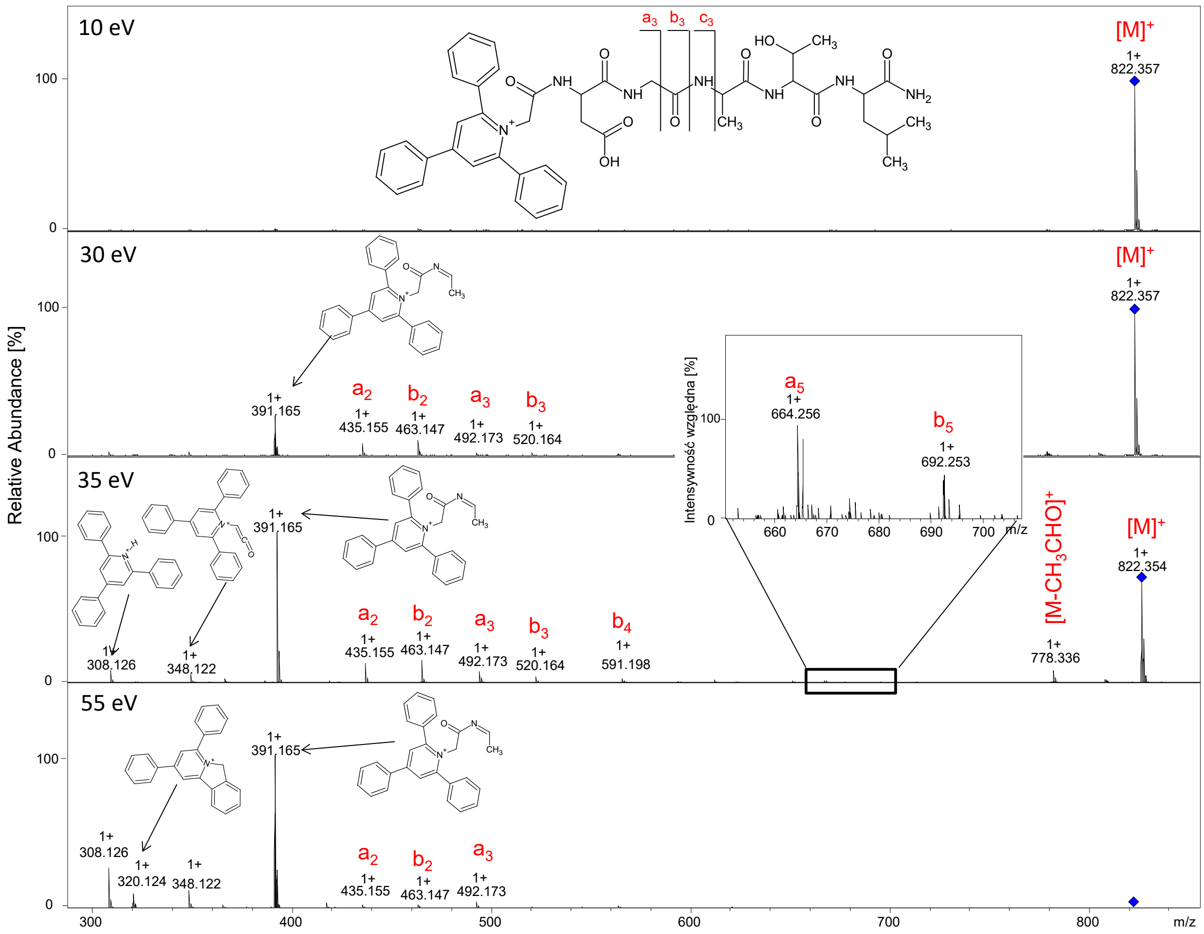

Figure S54. ESI-CID-MS/MS spectra of TPP⁺-CH₂CO-Asp-Gly-Ala-Thr-Leu-NH₂ (6c). Precursor ion at *m/z* 822.357.

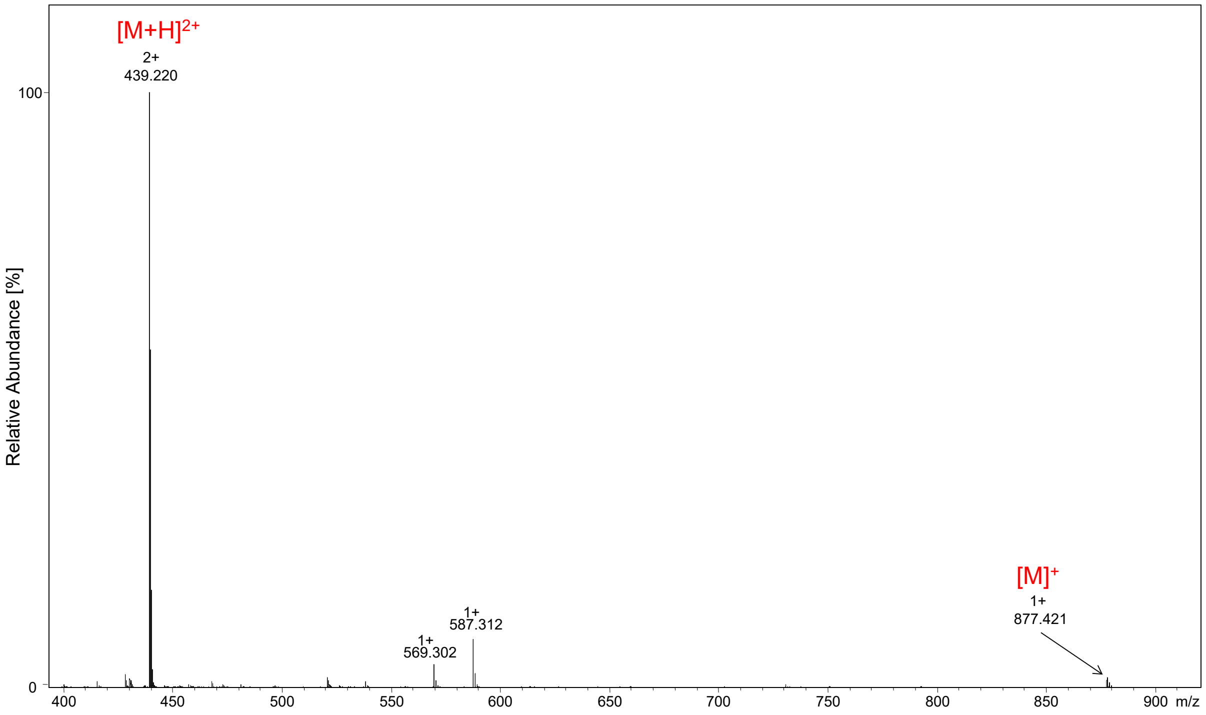

Figure S55. ESI-MS spectrum of TPP^+-CH_2CO-Asp-Gly-Arg-Ala-Leu-NH_2 (7c)

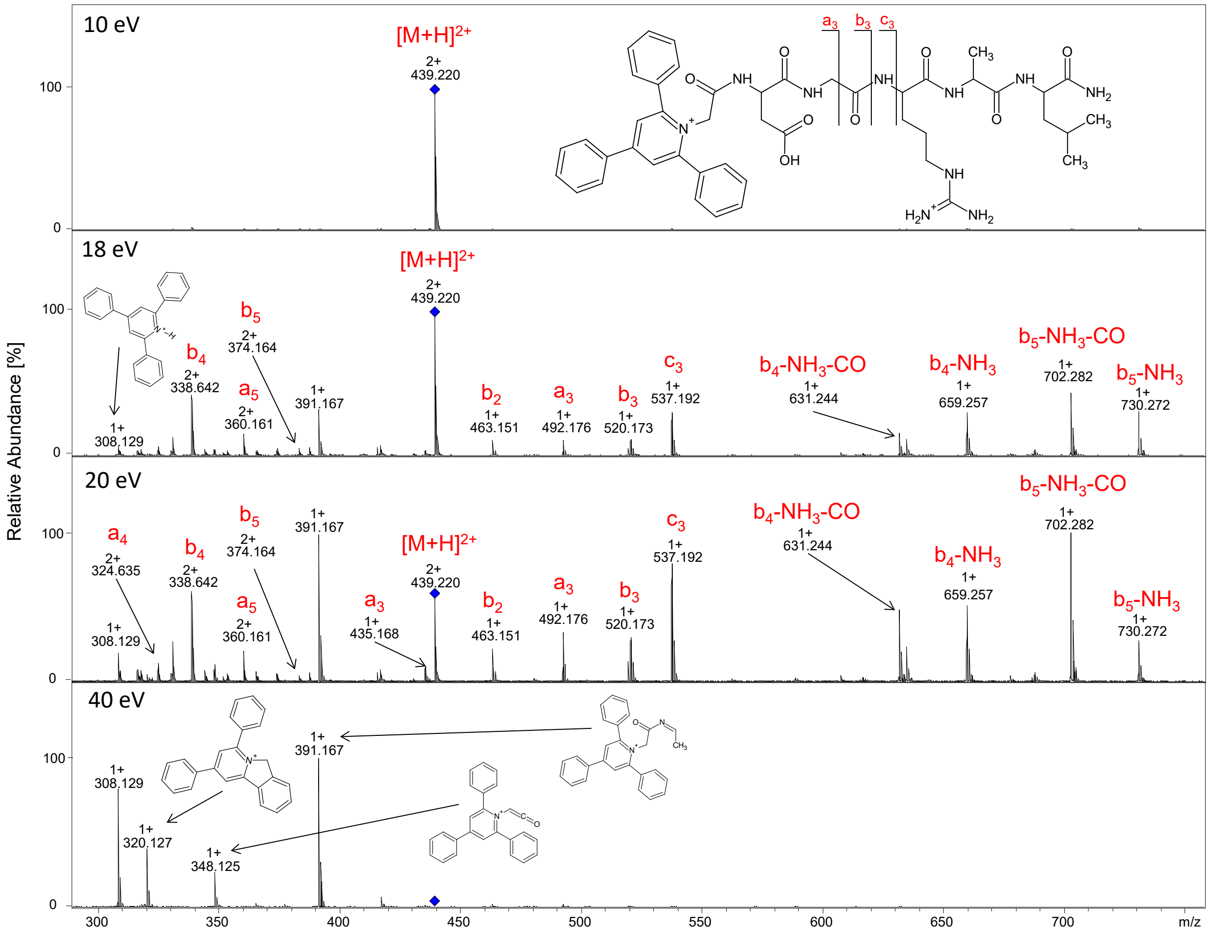

Figure S56. ESI-CID-MS/MS spectra of TPP⁺-CH2CO-Asp-Gly-Arg-Ala-Leu-NH2 (7c). Precursor ion at *m*/*z* 439.220.

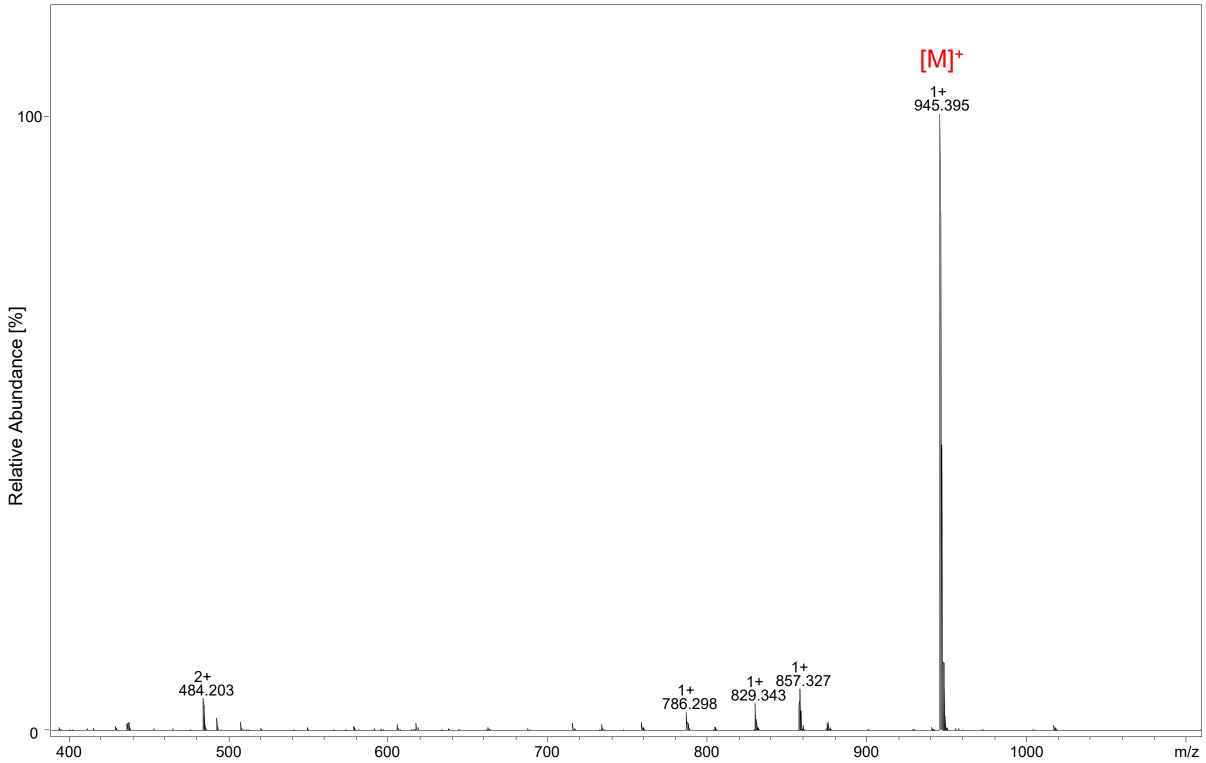

Figure S57. ESI-MS spectrum of $TMPP^{+}$-CH_2CO-Ala-Ala-Ala-Ala-Ala-NH_2 (1d)

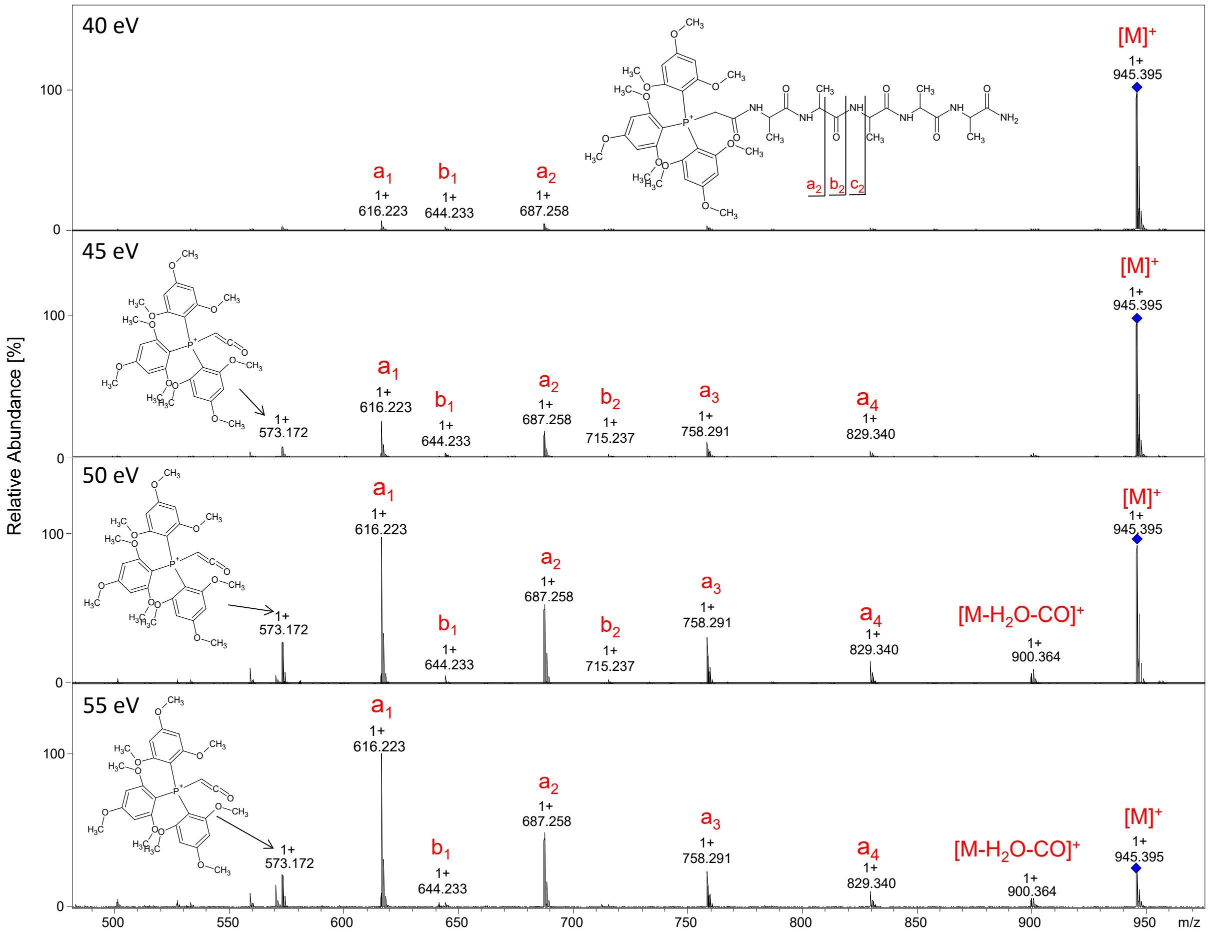

Figure S58. ESI-CID-MS/MS spectra of TMPP⁺-CH₂CO-Ala-Ala-Ala-Ala-Ala-NH₂ (1d). Precursor ion at *m/z* 945.395.

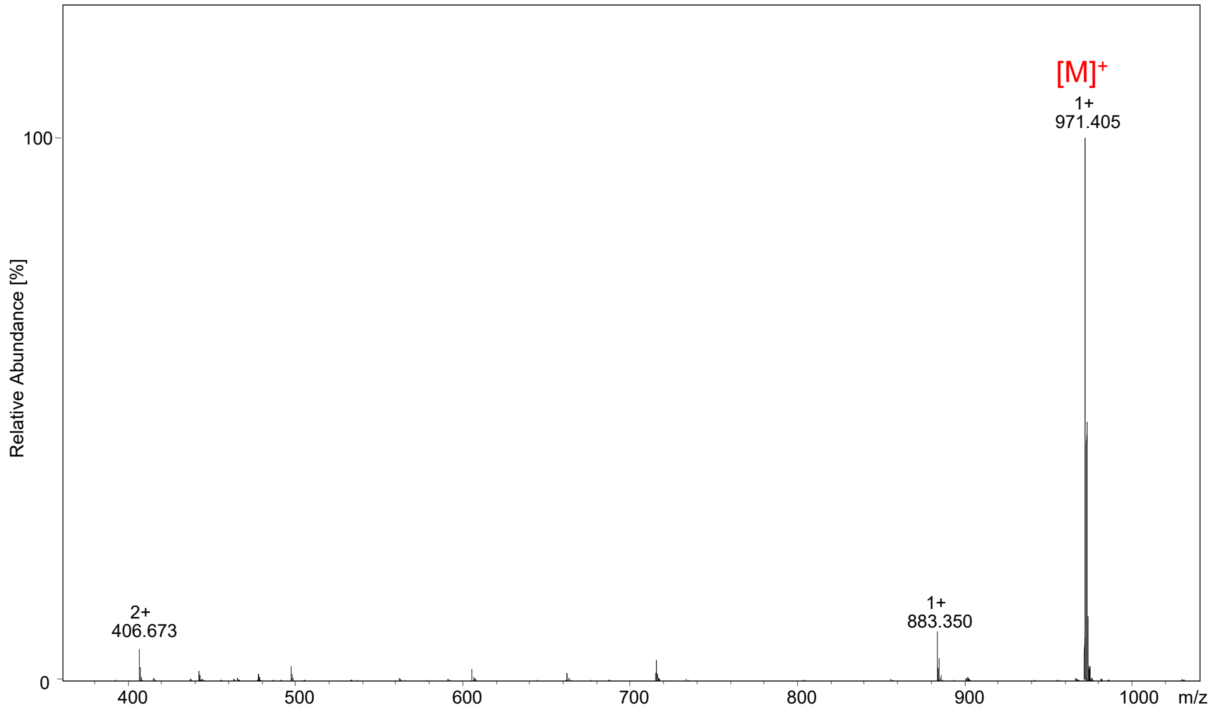

Figure S59. ESI-MS spectrum of TMPP$^+$-CH_2CO-Ala-Ala-Pro-Ala-Ala-NH_2 (2d)

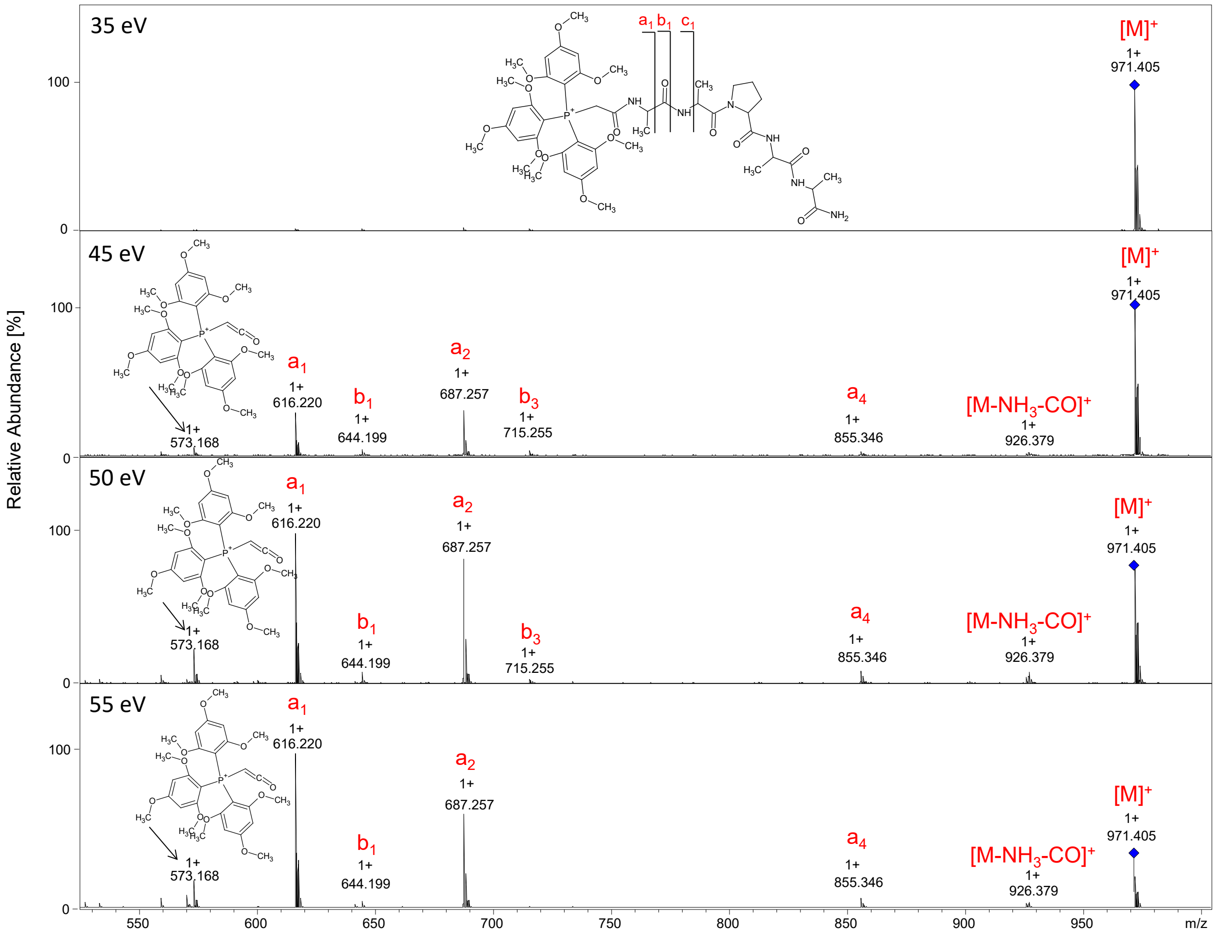

Figure S60. ESI-CID-MS/MS spectra of TMPP⁺-CH₂CO-Ala-Ala-Pro-Ala-Ala-NH₂ (2d). Precursor ion at *m/z* 971.405.

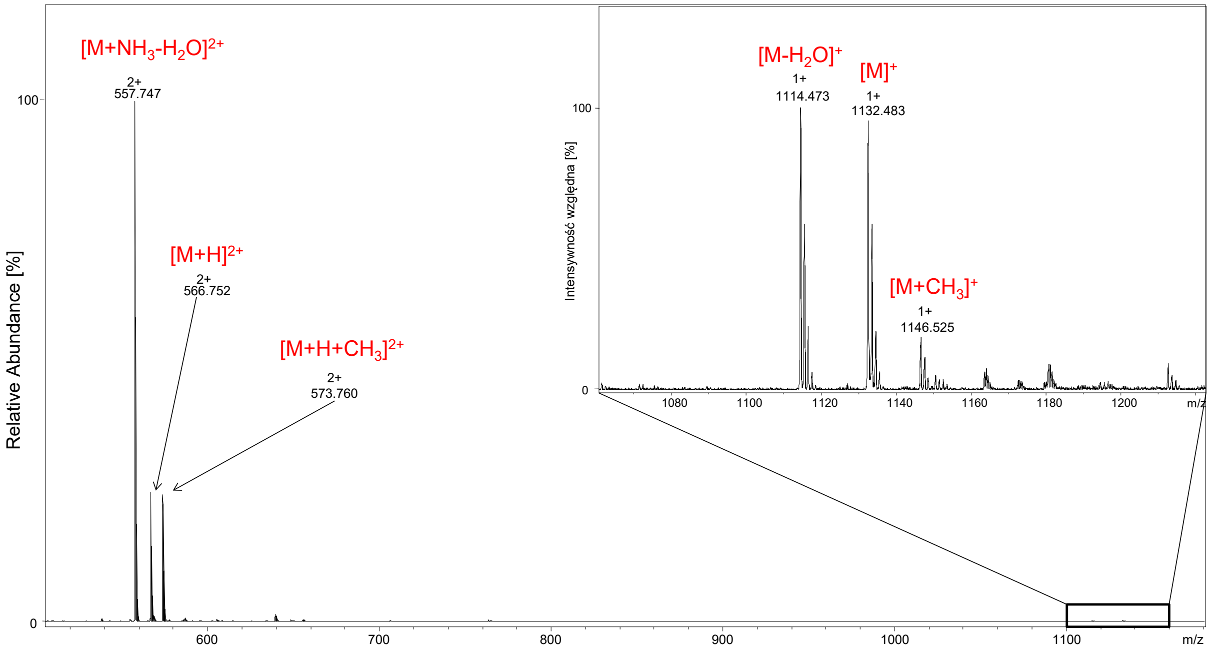

Figure S61. ESI-MS spectrum of TMPP$^+$-CH$_2$CO-Asp-Gly-Arg-Thr-Leu-NH$_2$ (3d)

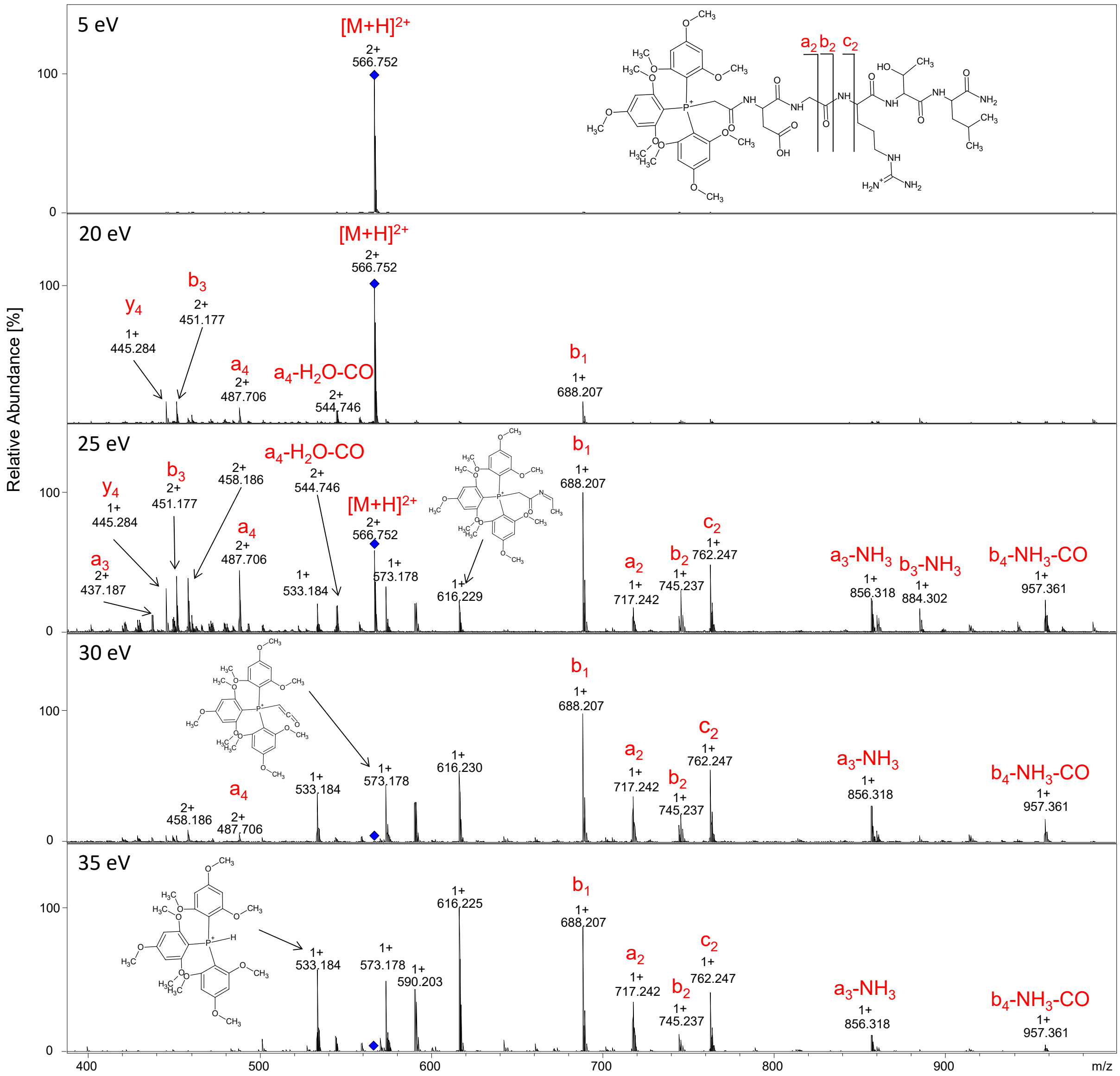

Figure S62. ESI-CID-MS/MS spectra of TMPP+-CH2CO-Asp-Gly-Arg-Thr-Leu-NH2 (3d). Precursor ion at *m/z* 566.752.

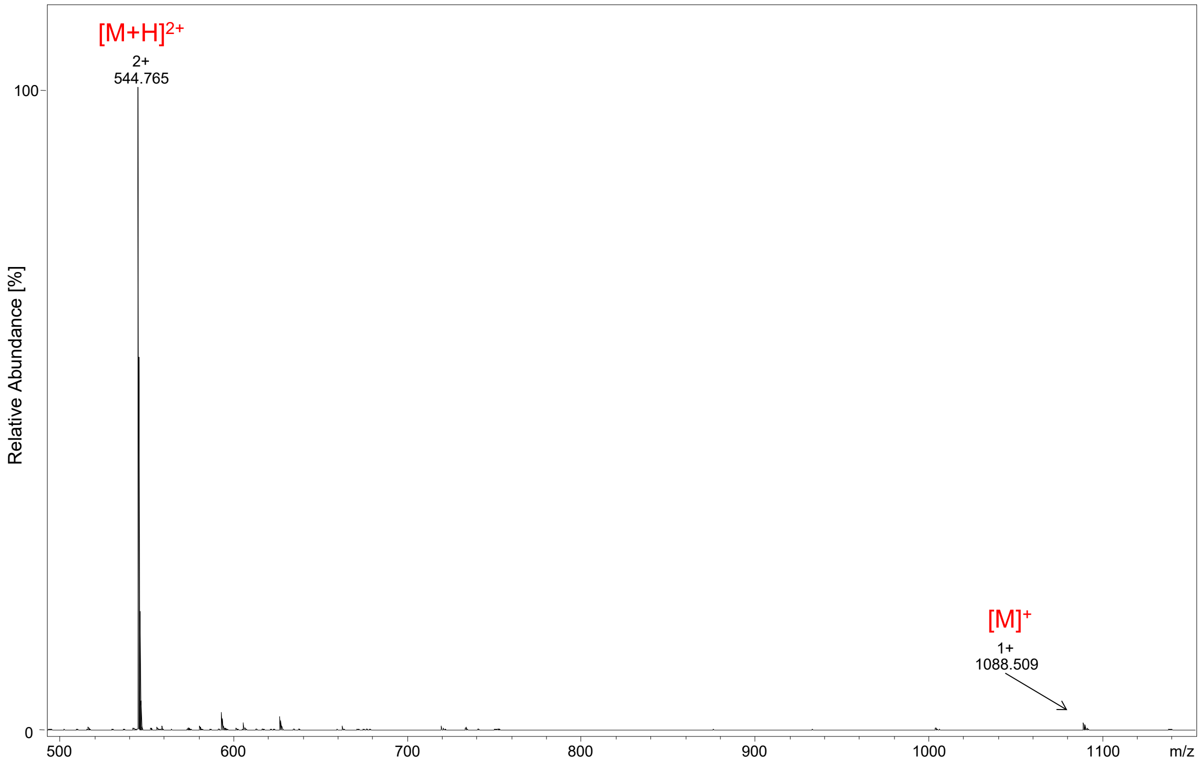

Figure S63. ESI-MS spectrum of TMPP⁺-CH_2CO-Ala-Gly-Arg-Thr-Leu-NH_2 (4d)

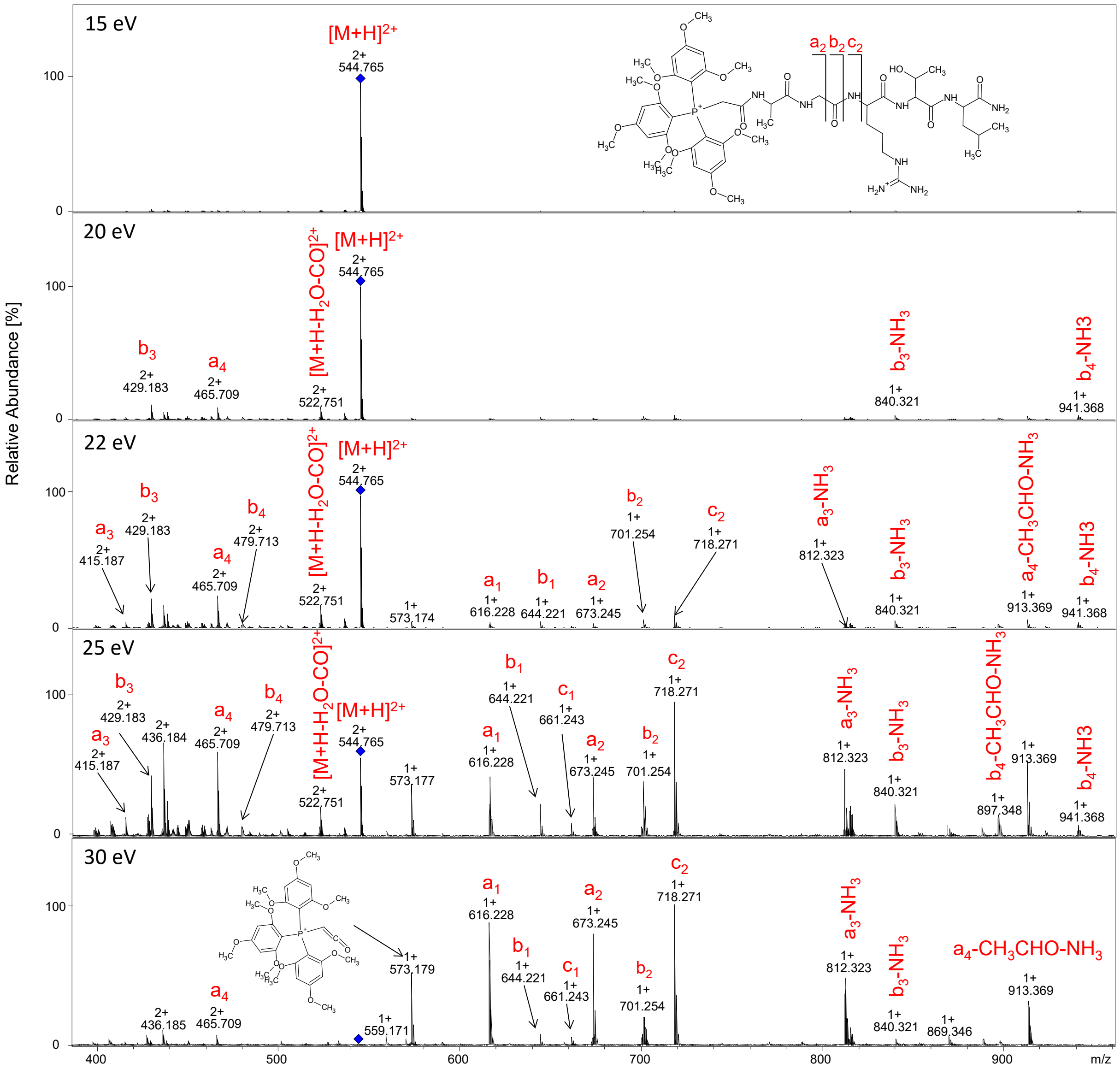

Figure S64. ESI-CID-MS/MS spectra of TMPP+-CH2CO-Ala-Gly-Arg-Thr-Leu-NH2 (4d). Precursor ion at *m*/*z* 544.765.

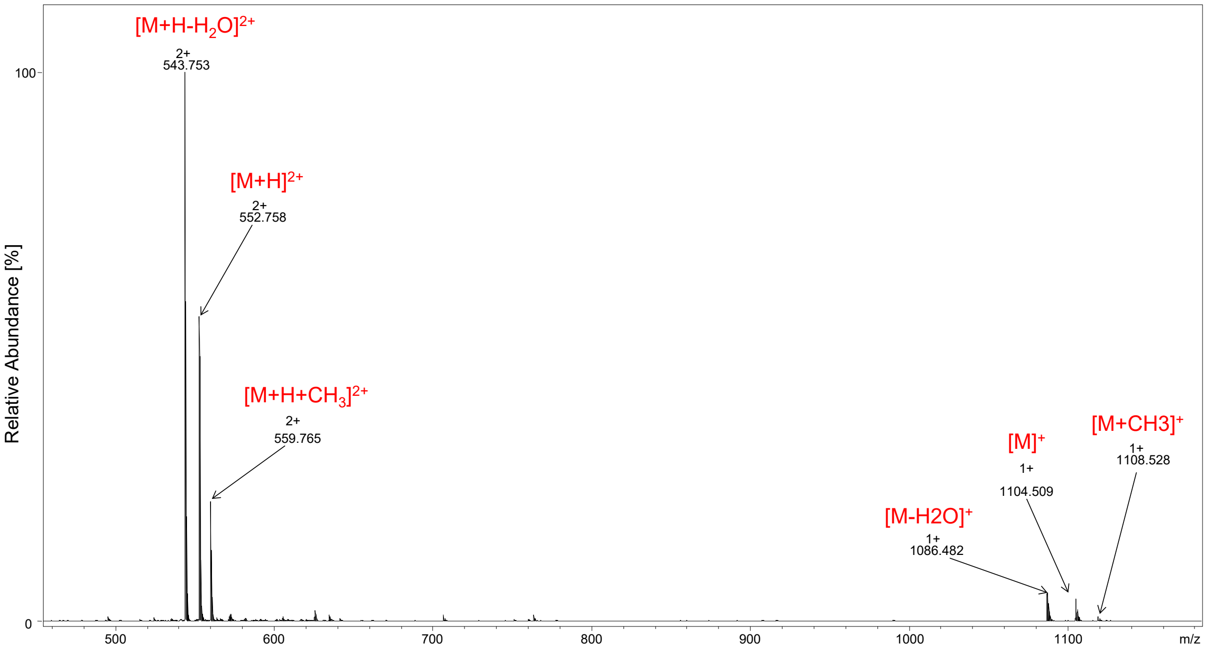

Figure S65. ESI-MS spectrum of TMPP+-CH2CO-Asp-Gly-Lys-Thr-Leu-NH2 (5d)

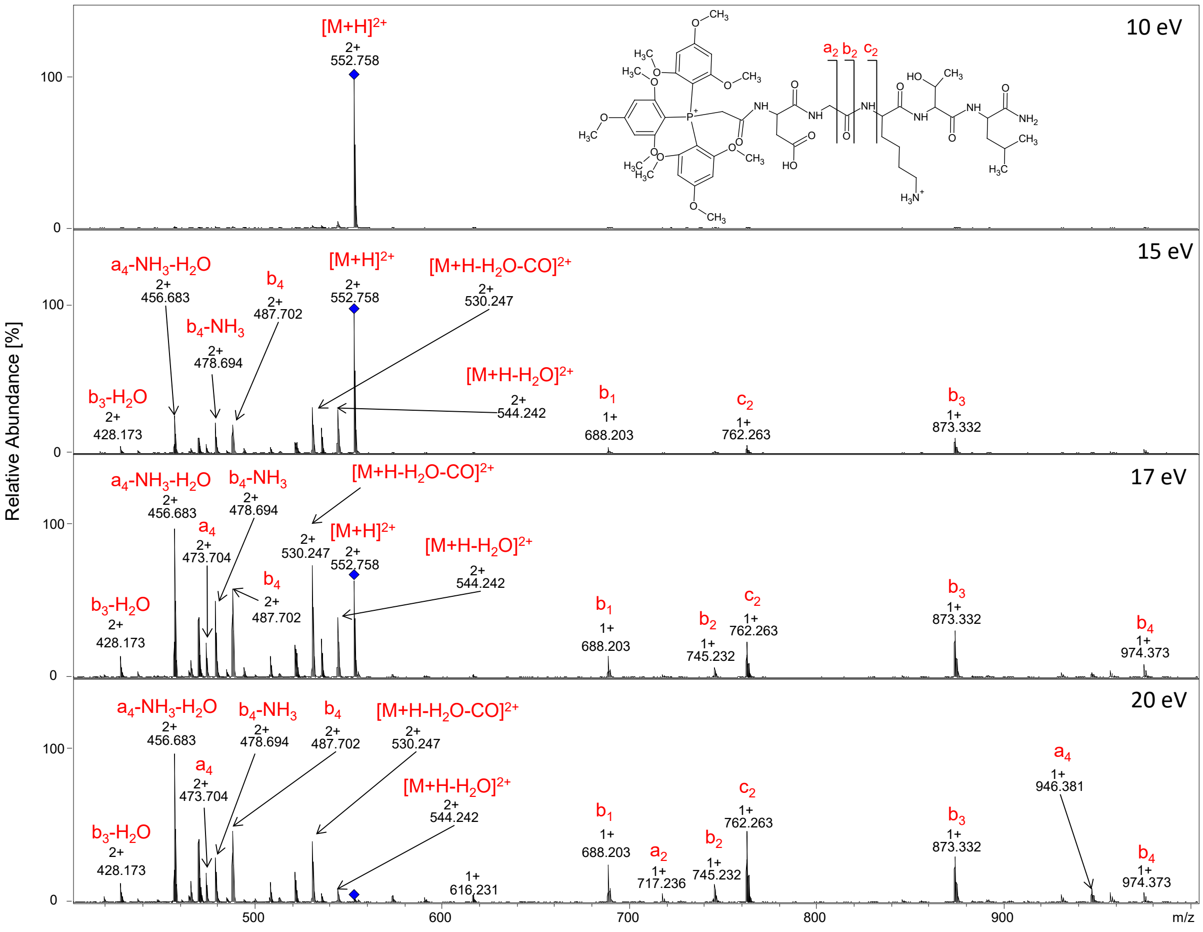

Figure S66. ESI-CID-MS/MS spectra of TMPP+-CH2CO-Asp-Gly-Lys-Thr-Leu-NH2 (5d). Precursor ion at *m/z* 552.758.

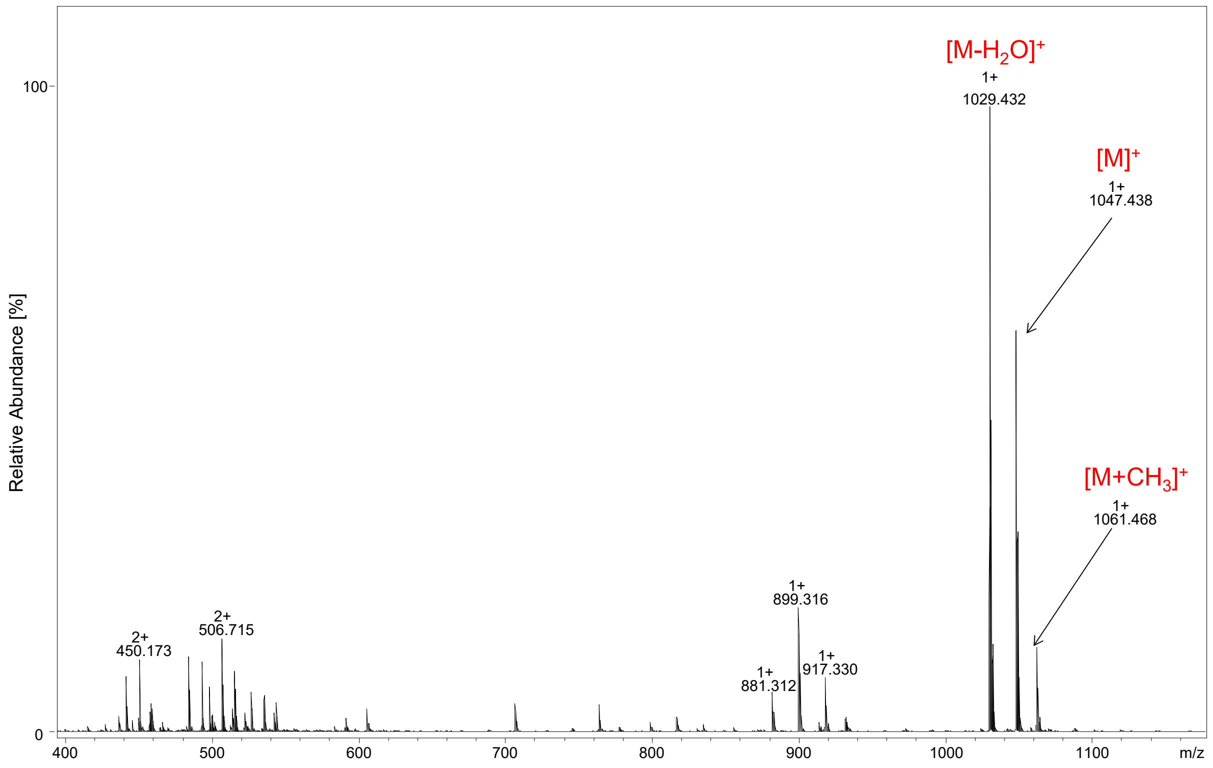

Figure S67. ESI-MS spectrum of TMPP+-CH2CO-Asp-Gly-Ala-Thr-Leu-NH2 (6d)

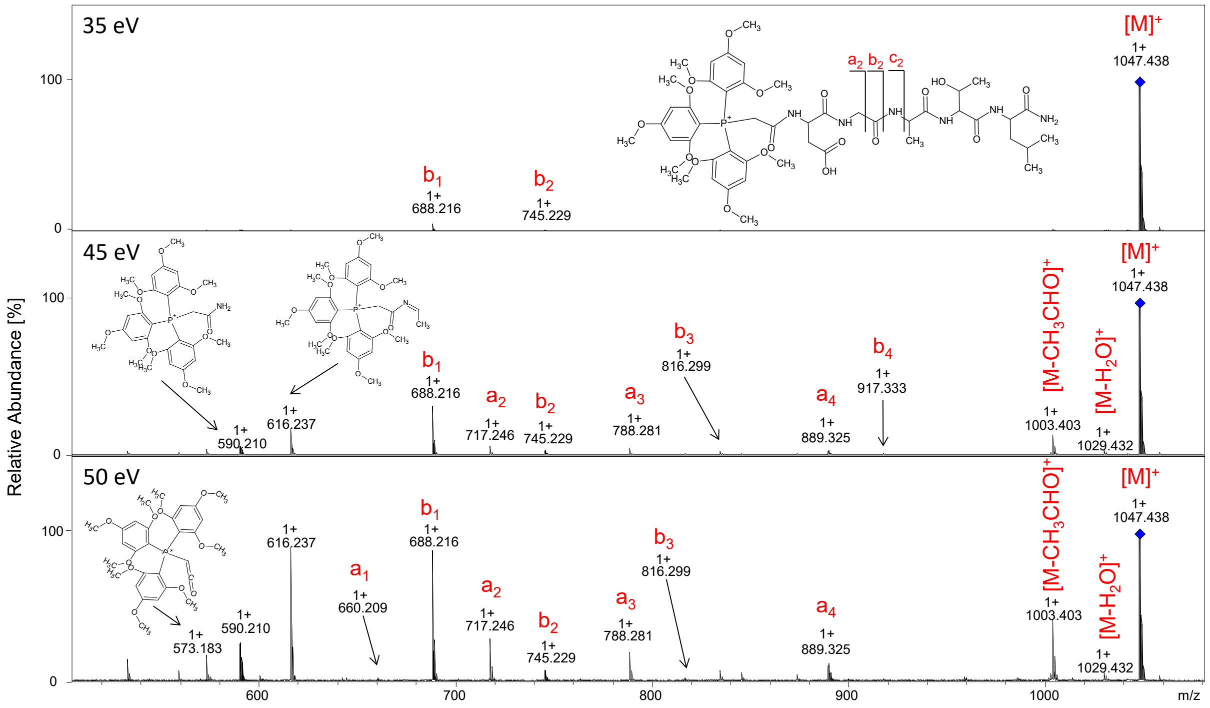

Figure S68. ESI-CID-MS/MS spectra of TMPP+-CH2CO-Asp-Gly-Ala-Thr-Leu-NH2 (6d). Precursor ion at *m*/*z* 1047.438.

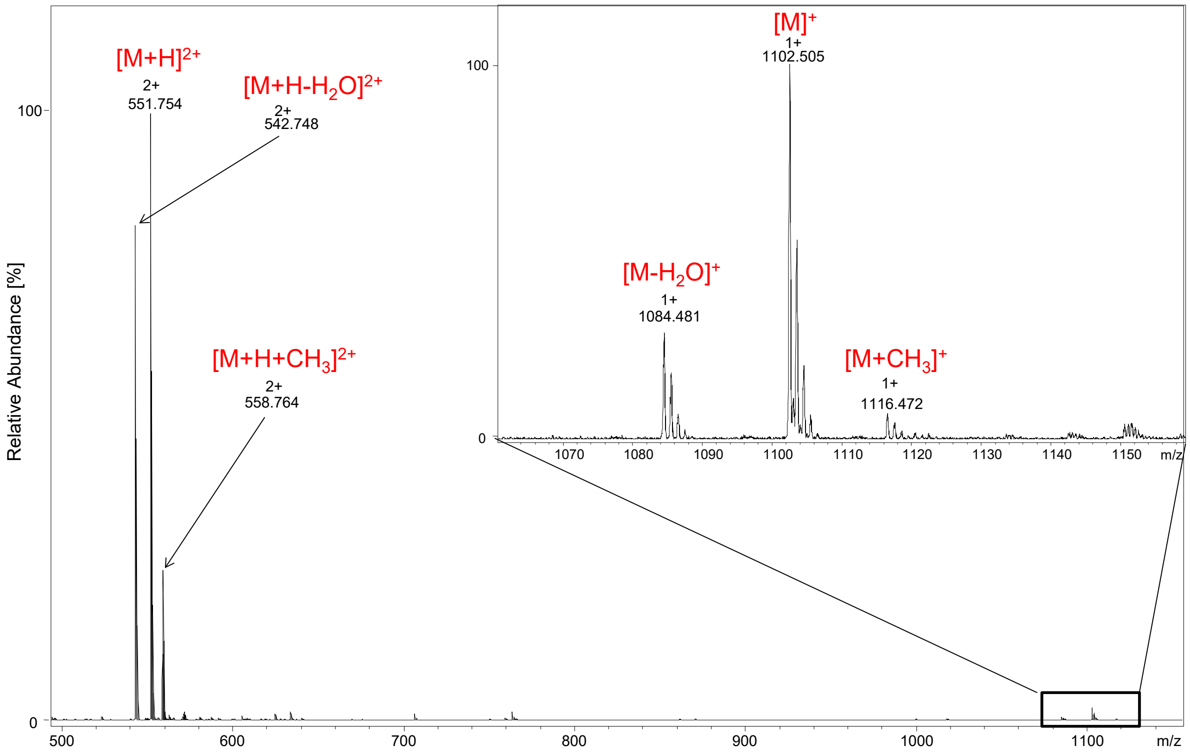

Figure S69. ESI-MS spectrum of TMPP+-CH2CO-Asp-Gly-Arg-Ala-Leu-NH2 (7d)

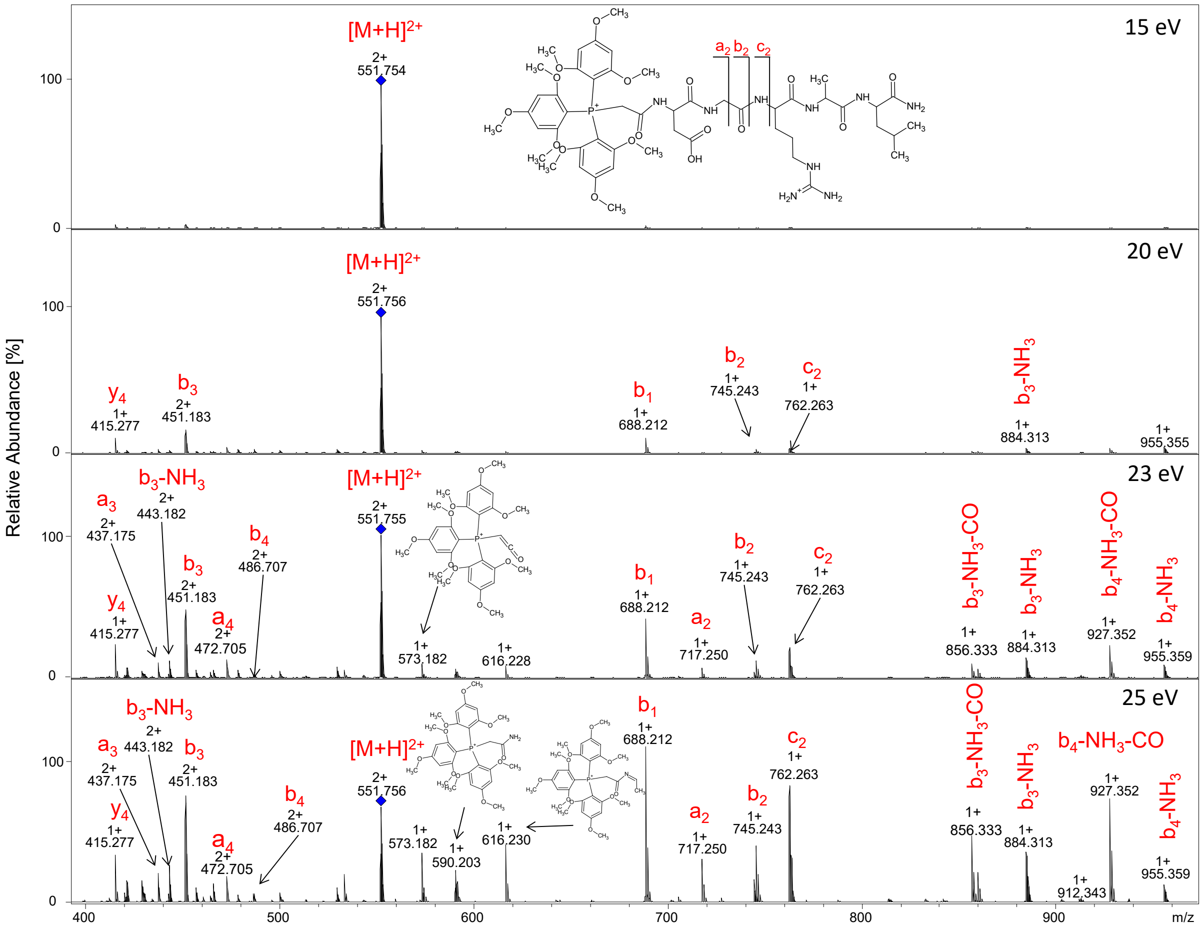

Figure S70. ESI-CID-MS/MS spectra of TMPP+-CH2CO-Asp-Gly-Arg-Ala-Leu-NH2 (7d). Precursor ion at *m*/*z* 551.756.

2.2 ESI-ECD-MS/MS spectra

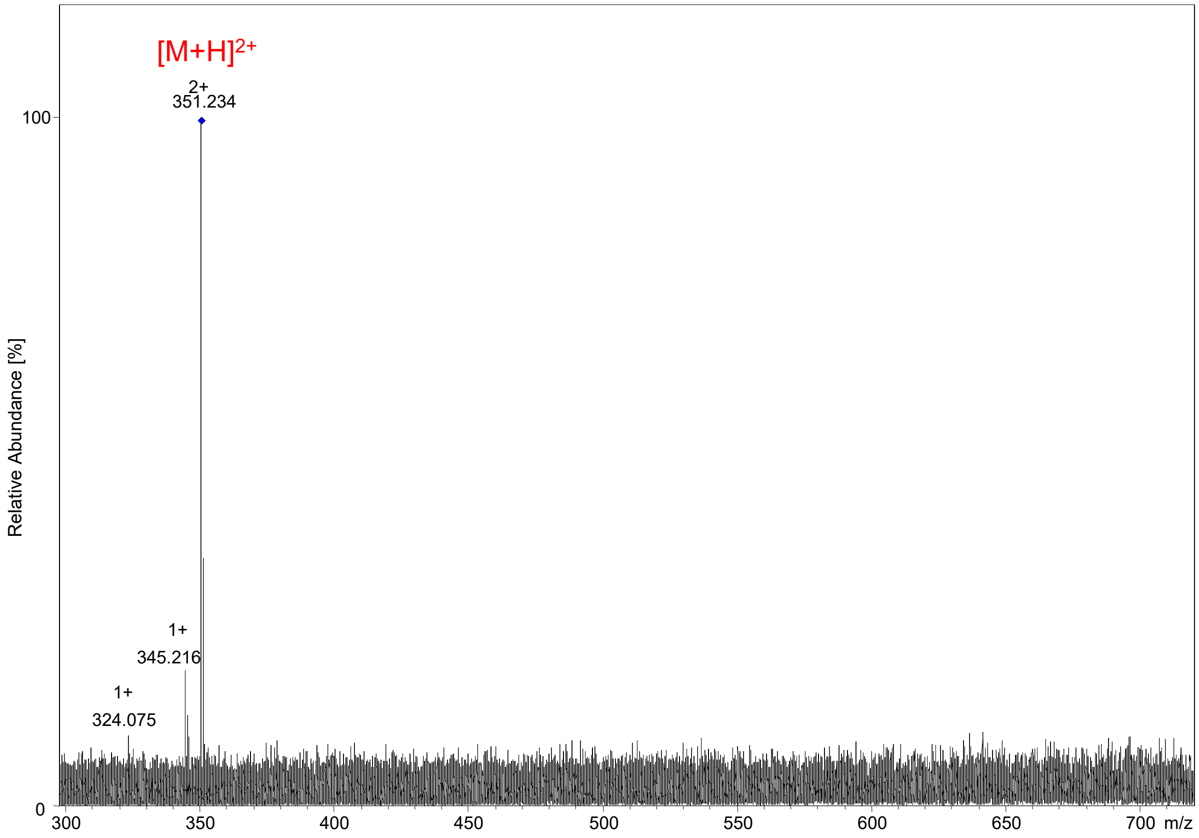

Figure S71. ESI-ECD-MS/MS spectrum of TEA$^+$-CH_2CO-Asp-Gly-Arg-Thr-Leu-NH_2 (3a)

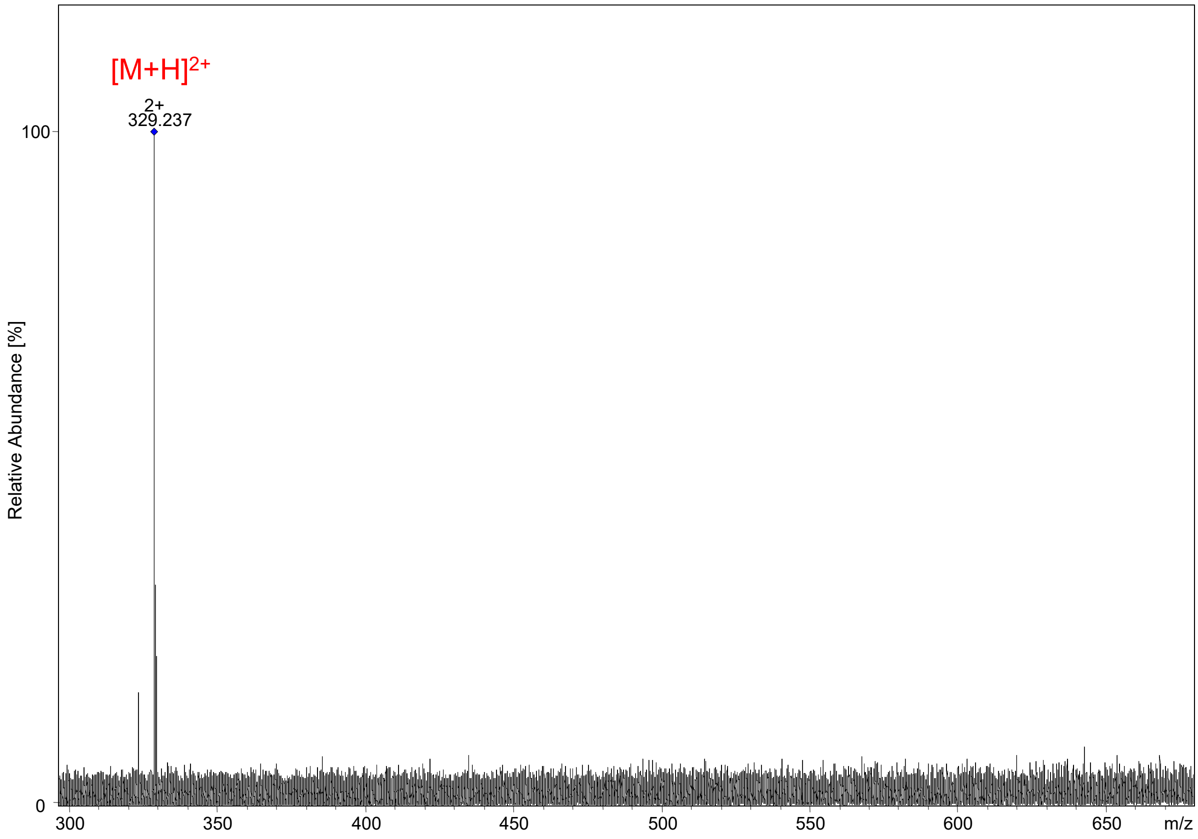

Figure S72. ESI-ECD-MS/MS spectrum of TEA⁺-CH_2CO-Ala-Gly-Arg-Thr-Leu-NH_2(4a)

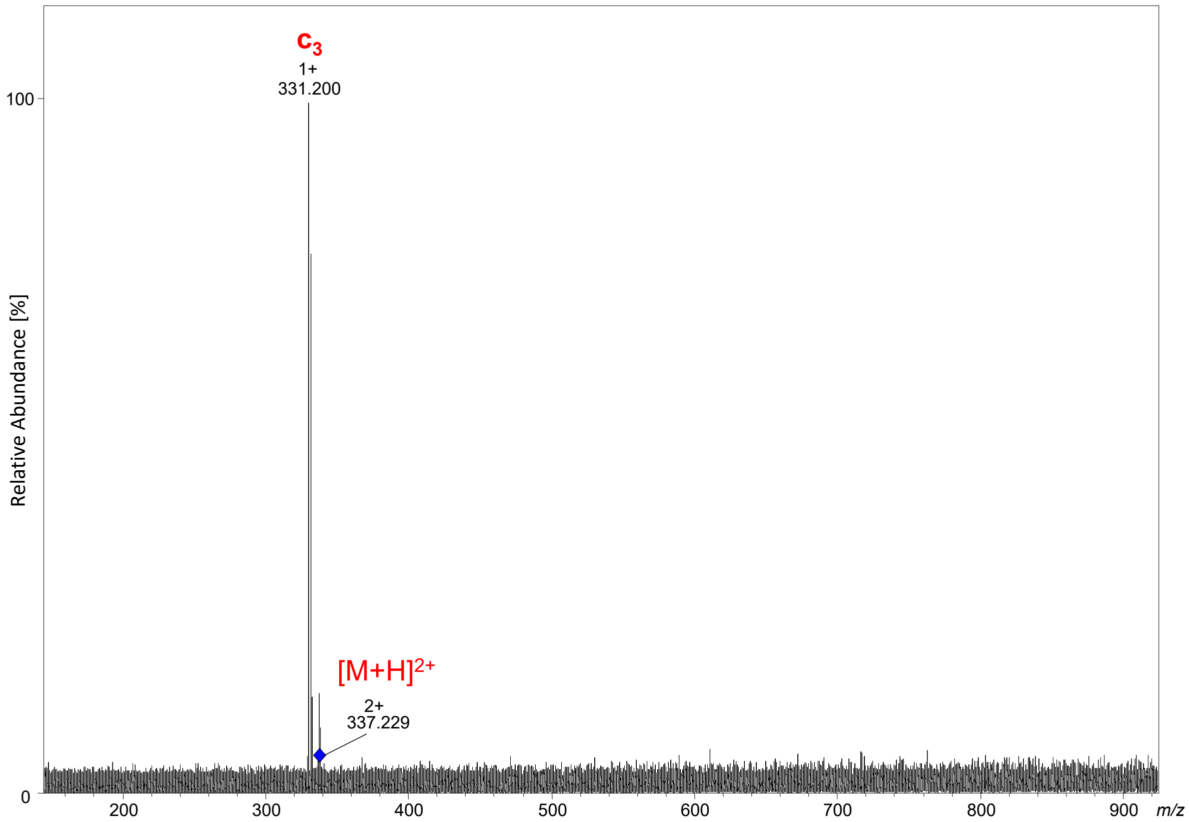

Figure S73. ESI-ECD-MS/MS spectrum of TEA⁺-CH_2CO-Asp-Gly-Lys-Thr-Leu-NH_2 (5a). Precursor ion at *m*/*z* 337.229.

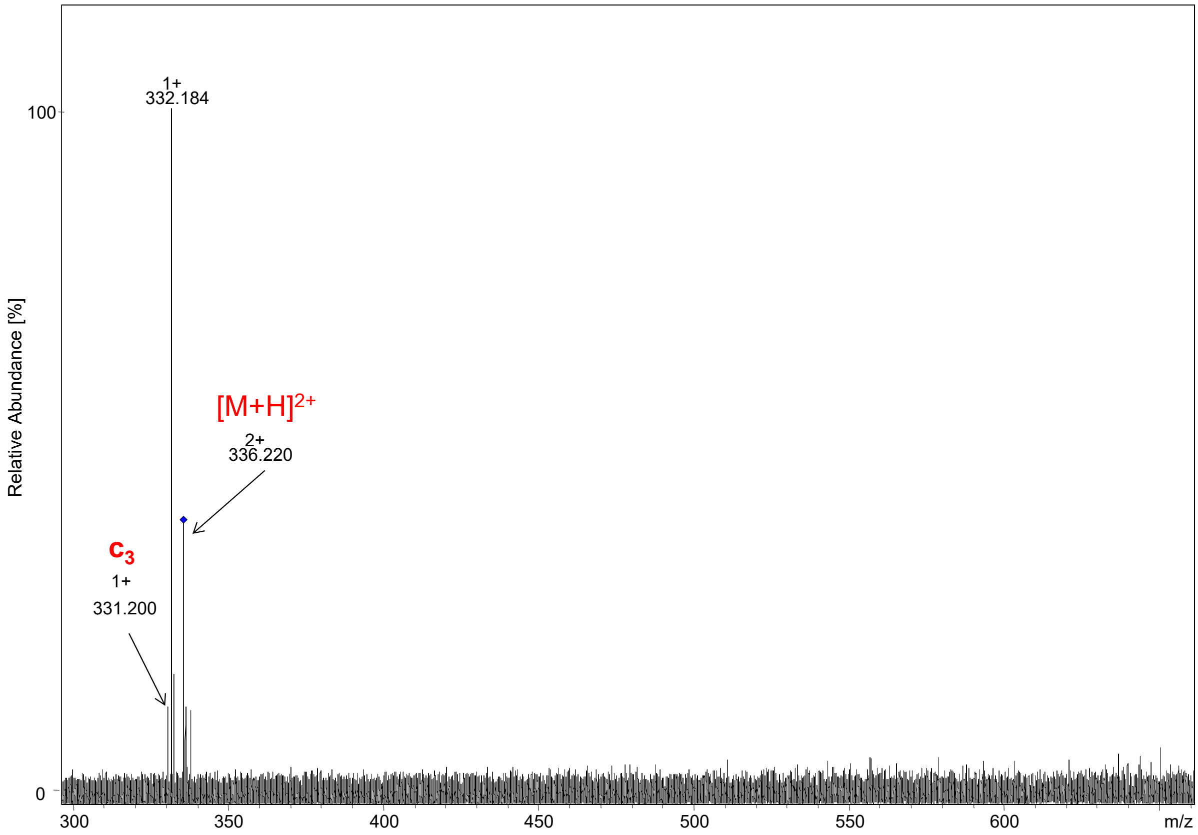

Figure S74. ESI-ECD-MS/MS spectrum of TEA⁺-CH_2CO-Asp-Gly-Arg-Ala-Leu-NH_2 (7b)

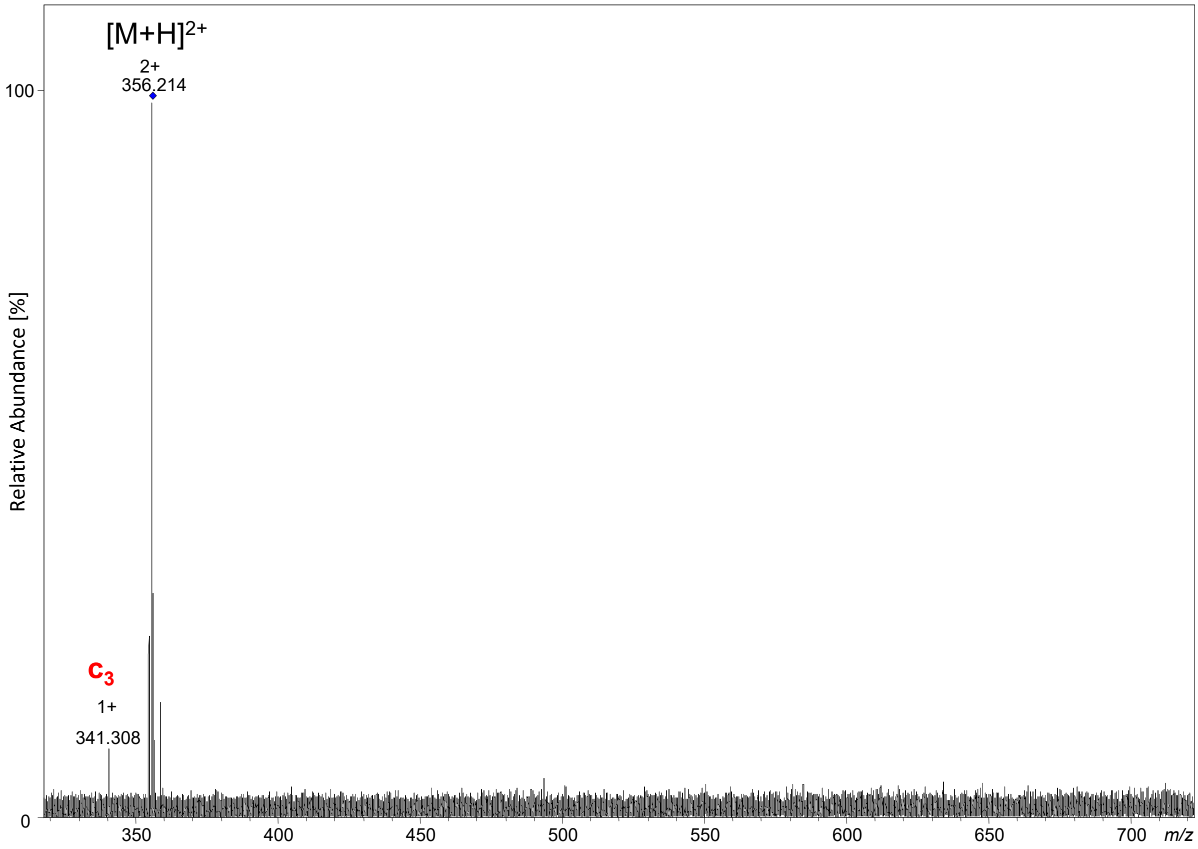

Figure S75. ESI-ECD-MS/MS spectrum of ABCO$^+$-CH_2CO-Asp-Gly-Arg-Thr-Leu-NH_2 (3b). Precursor ion at *m/z* 356.221.

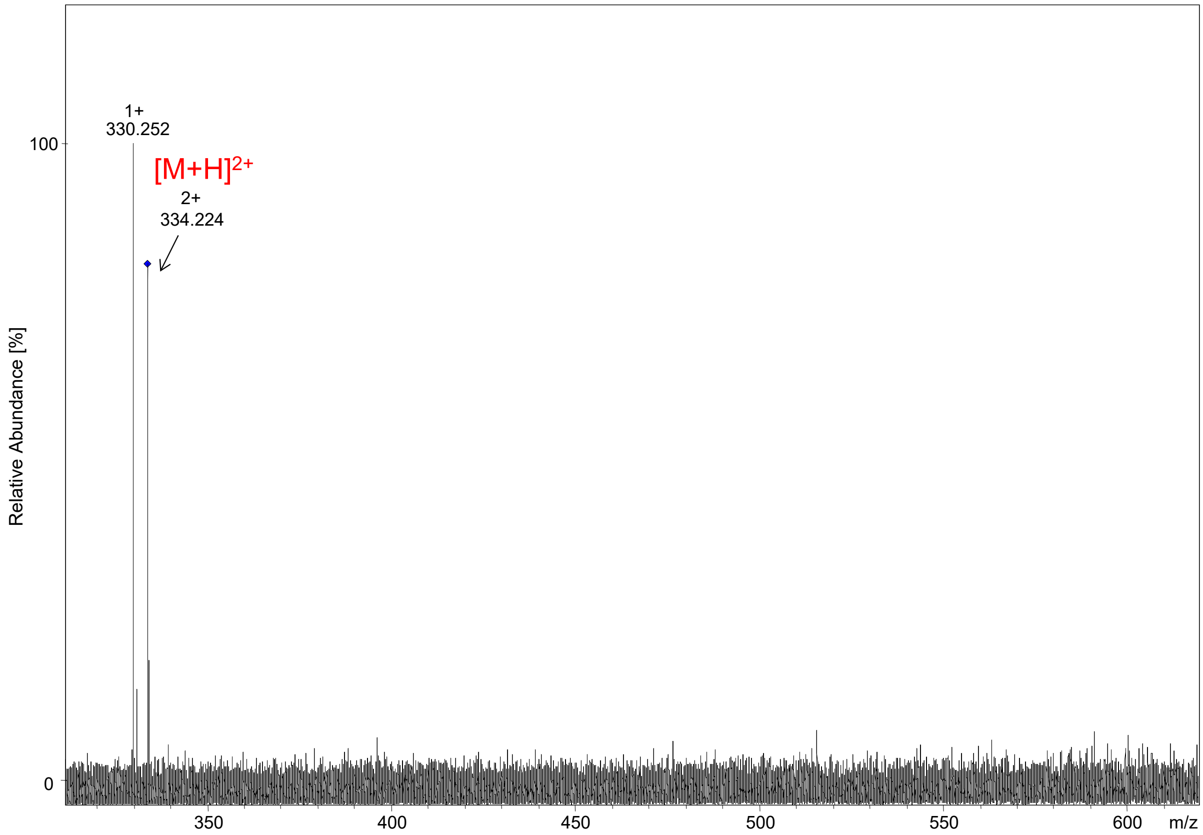

Figure S76. ESI-ECD-MS/MS spectrum of ABCO^{+}-CH_2CO-Ala-Gly-Arg-Thr-Leu-NH_2 (4b)

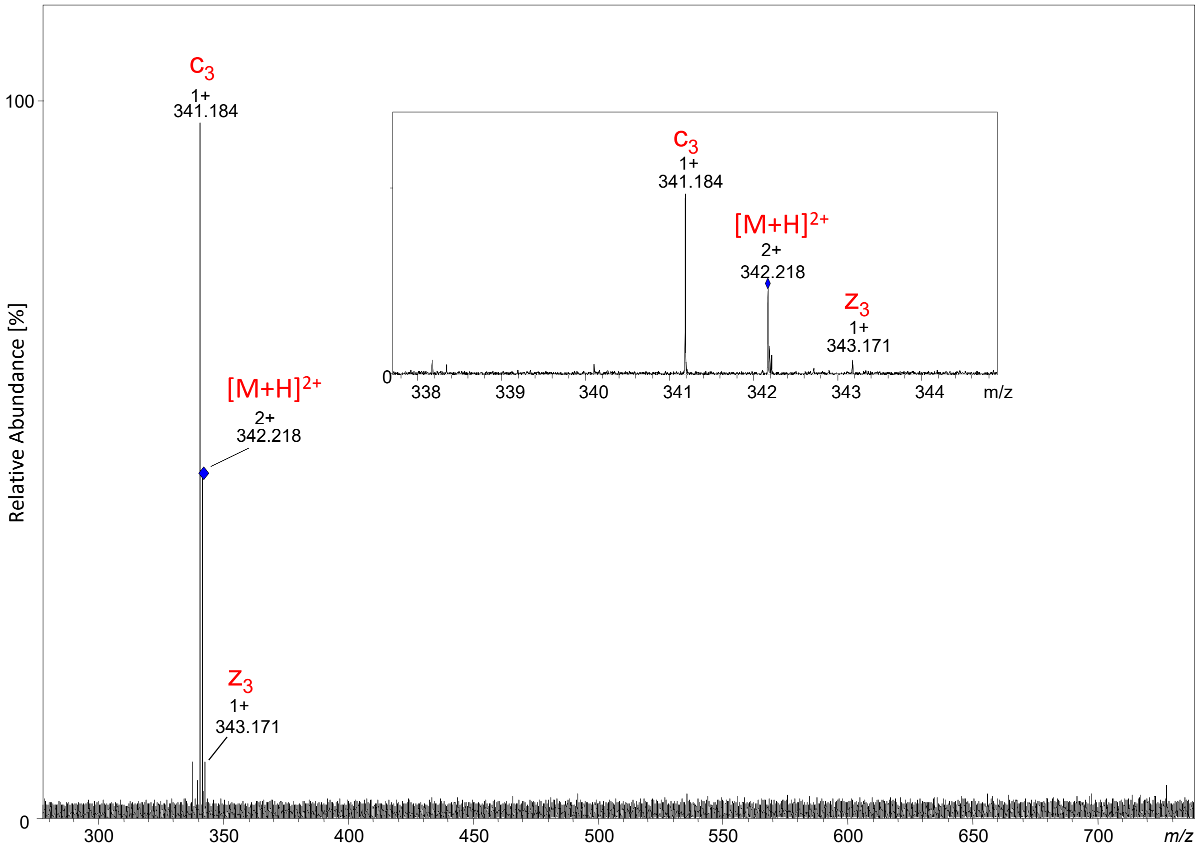

Figure S77. ESI-ECD-MS/MS spectrum of ABCO^{+}-CH$_2$CO-Asp-Gly-Lys-Thr-Leu-NH$_2$ (5b). Precursor ion at *m*/*z* 342.218.

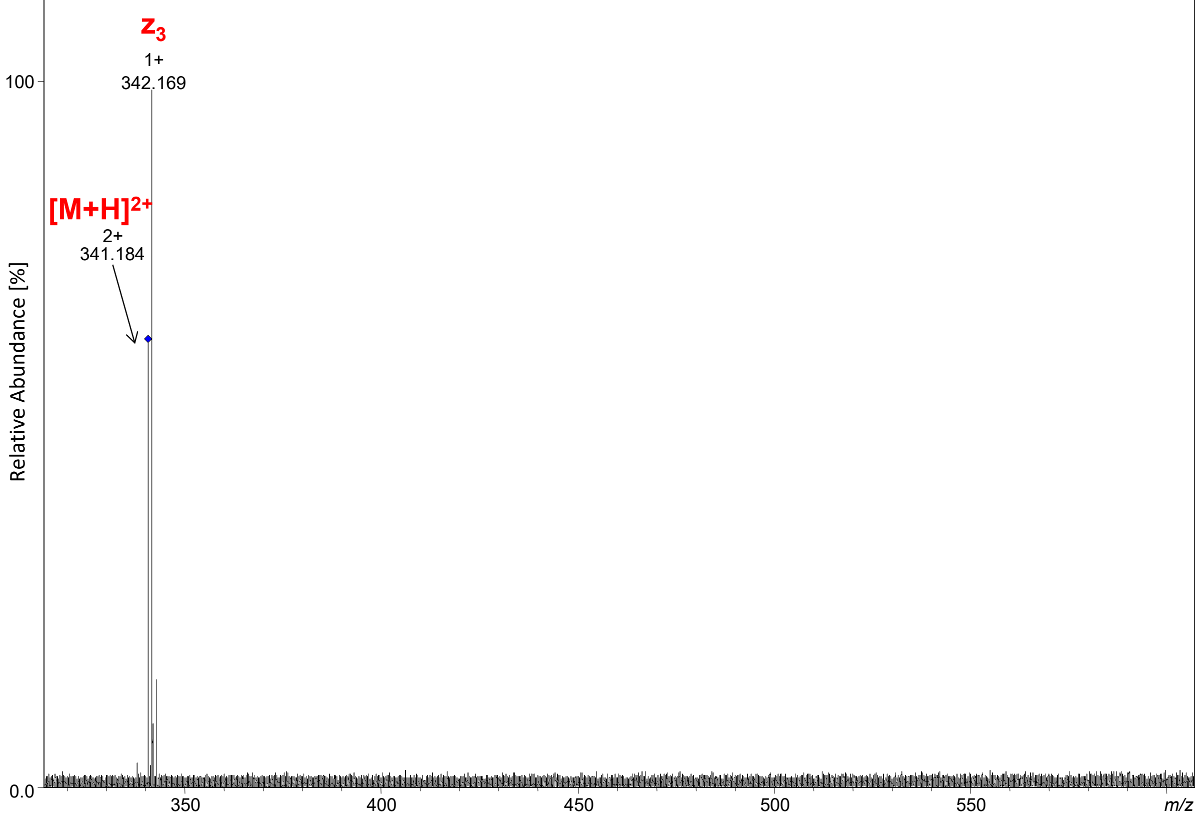

Figure S78. ESI-ECD-MS/MS spectrum of ABCO⁺-CH₂CO-Asp-Gly-Arg-Ala-Leu-NH₂ (7b)

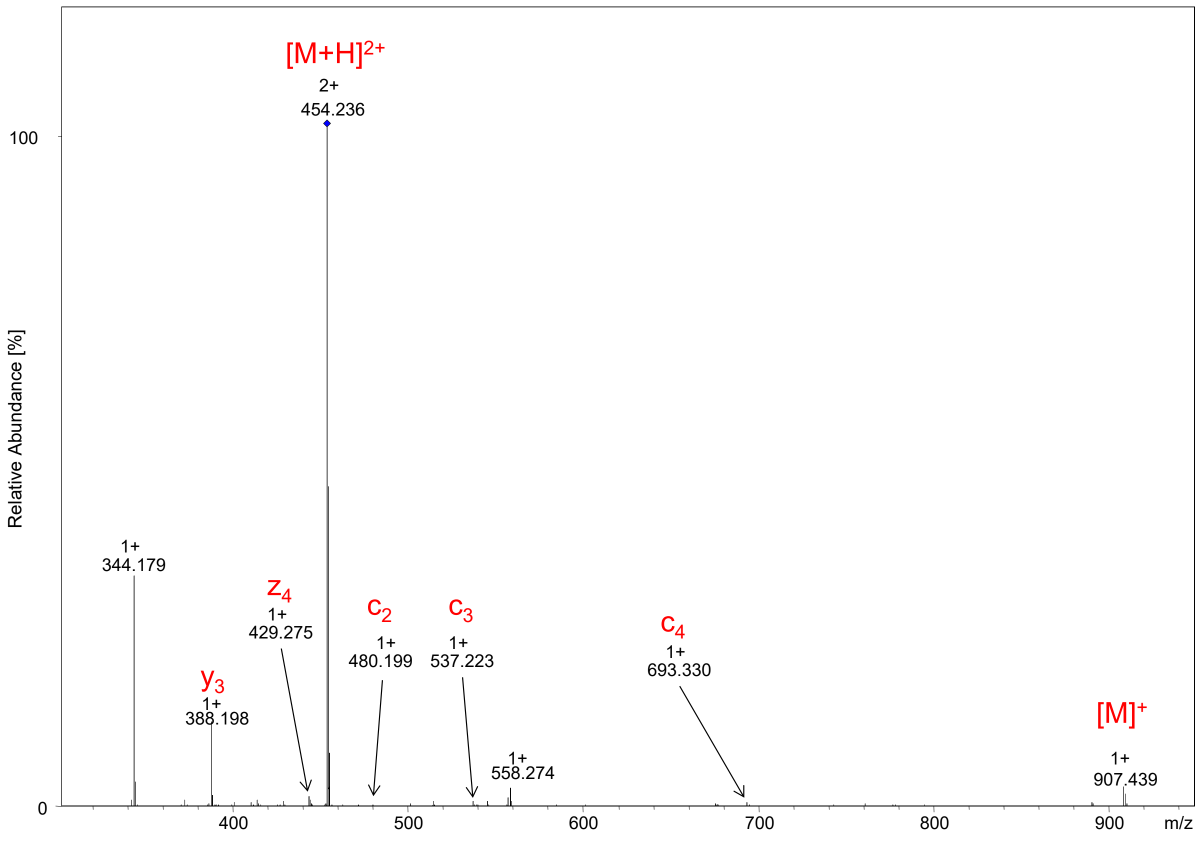

Figure S79. ESI-ECD-MS/MS spectrum of TPP⁺-CH2CO-Asp-Gly-Arg-Thr-Leu-NH2 (3c). Precursor ion at *m*/*z* 454.236.

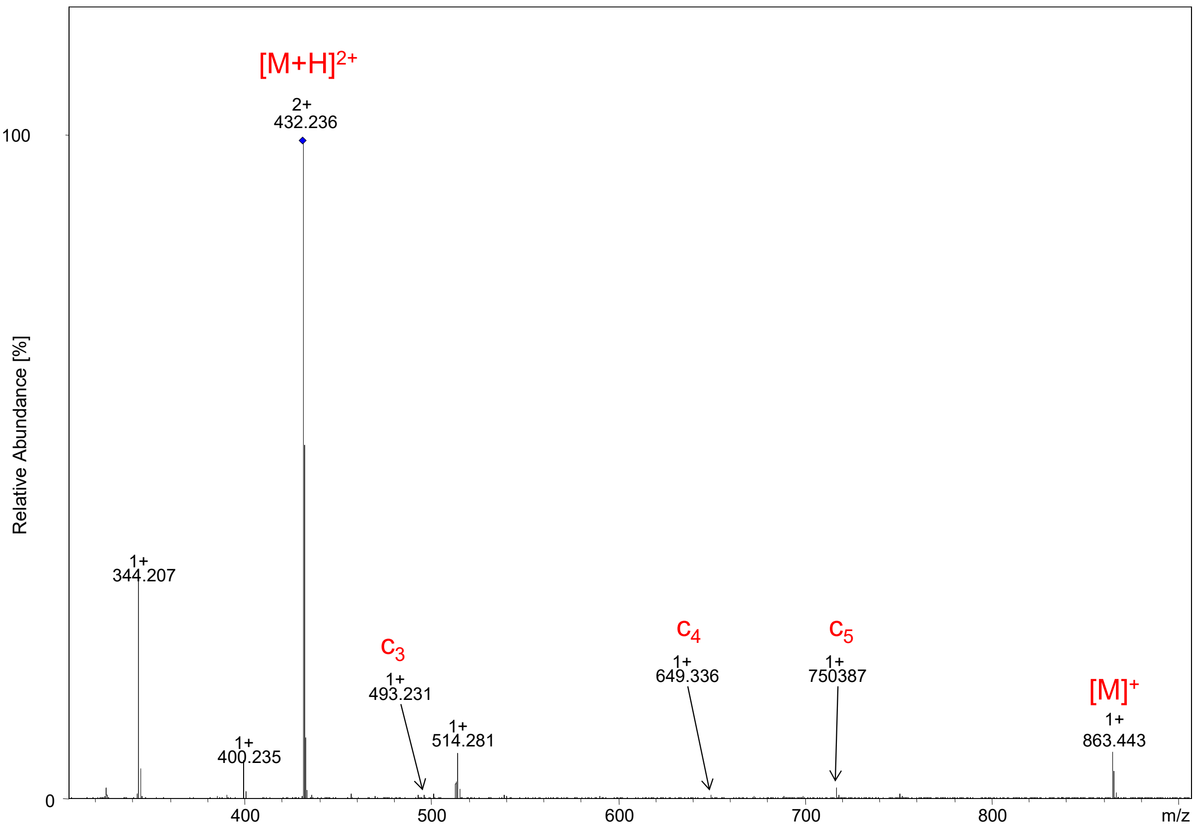

Figure S80. ESI-ECD-MS/MS spectrum of TPP+-CH2CO-Ala-Gly-Arg-Thr-Leu-NH2 (4c). Precursor ion at *m*/*z* 432.236.

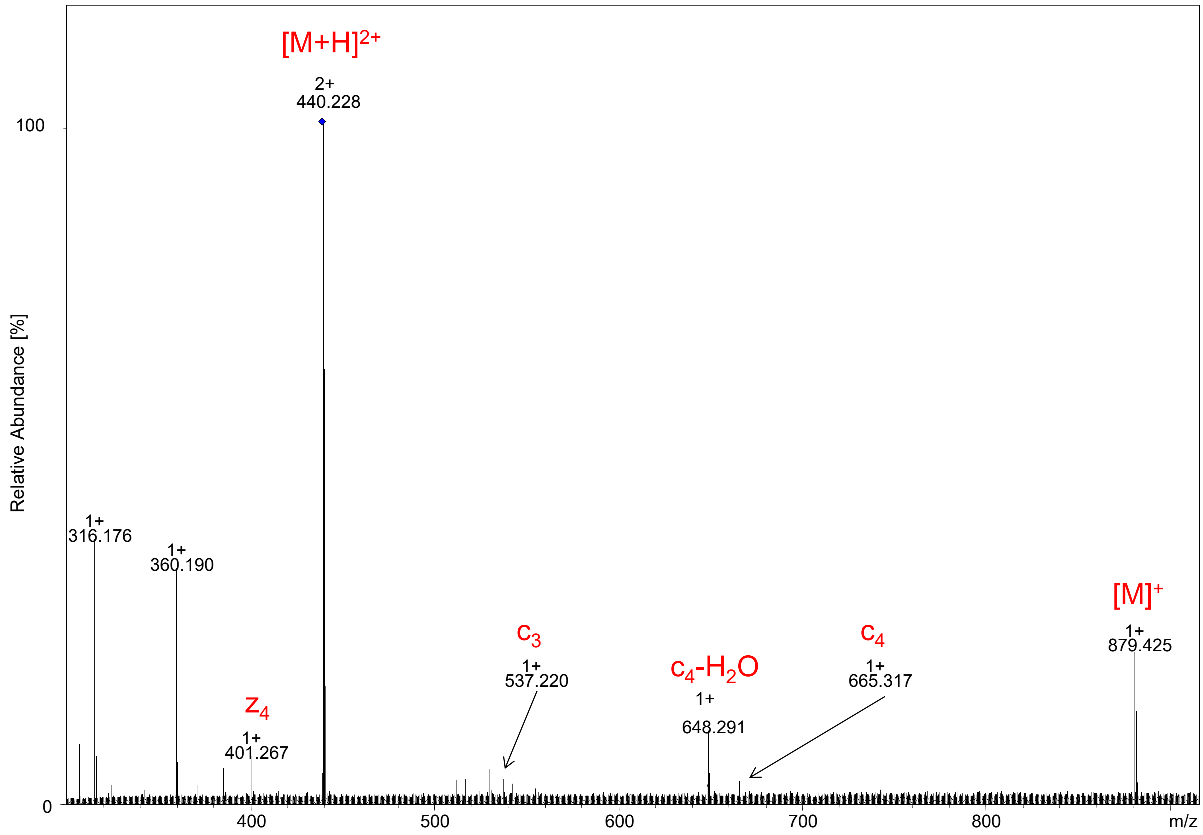

Figure S81. ESI-ECD-MS/MS spectrum of TPP⁺-CH_2CO-Asp-Gly-Lys-Thr-Leu-NH_2 (5c). Precursor ion at *m*/*z* 440.228.

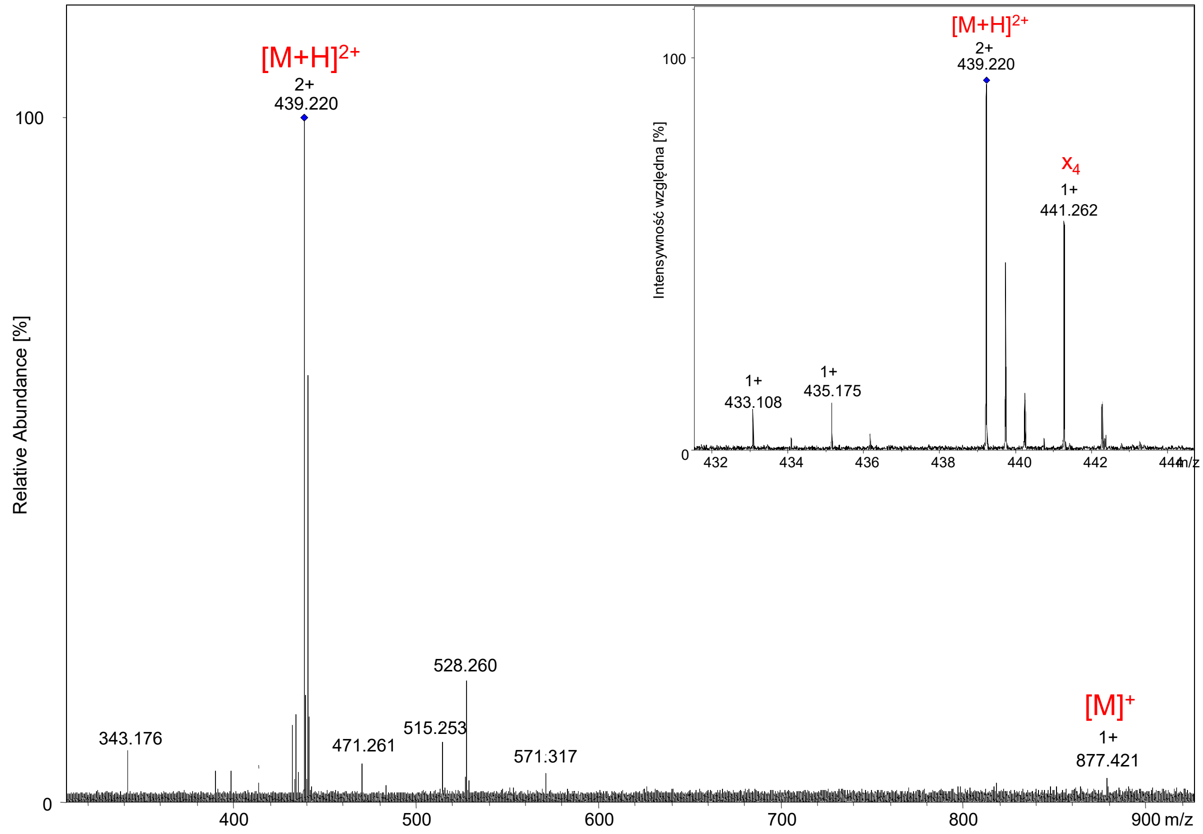

Figure S82. ESI-ECD-MS/MS spectrum of TPP+-CH2CO-Asp-Gly-Arg-Ala-Leu-NH2 (7c). Precursor ion at *m*/*z* 439.220.

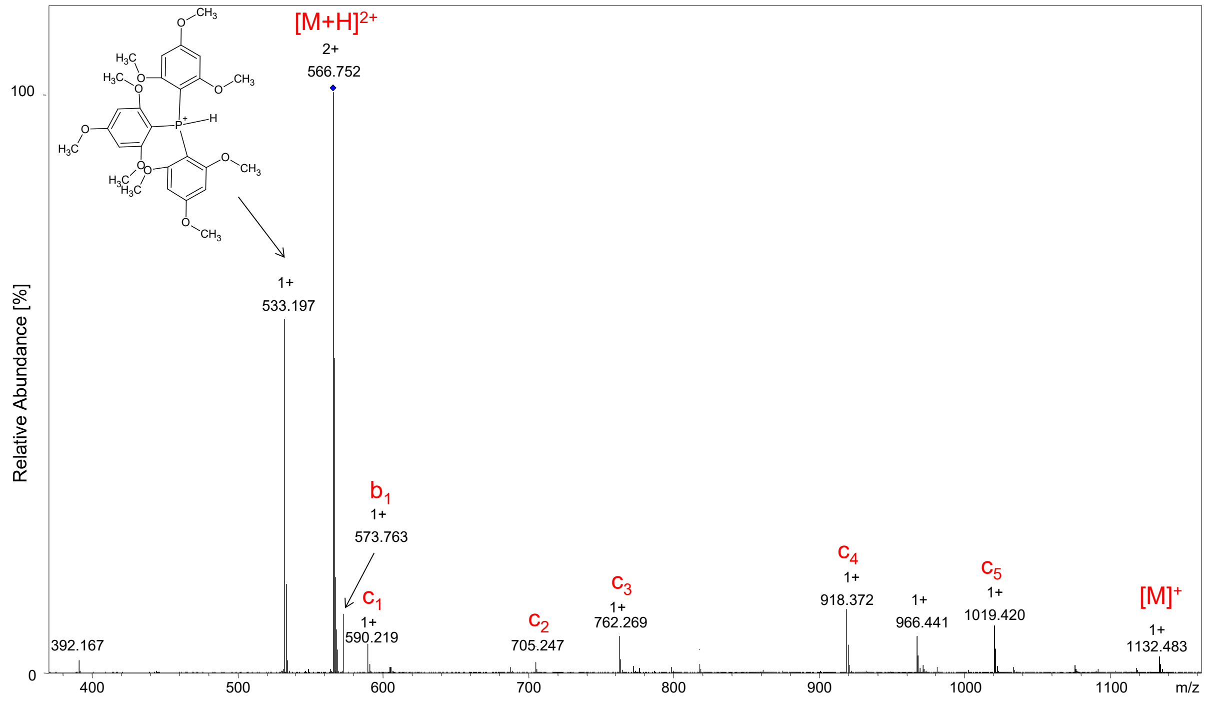

Figure S83. ESI-ECD-MS/MS spectrum of TMPP⁺-CH₂CO-Asp-Gly-Arg-Thr-Leu-NH₂ (3d). Precursor ion at *m/z* 566.752.

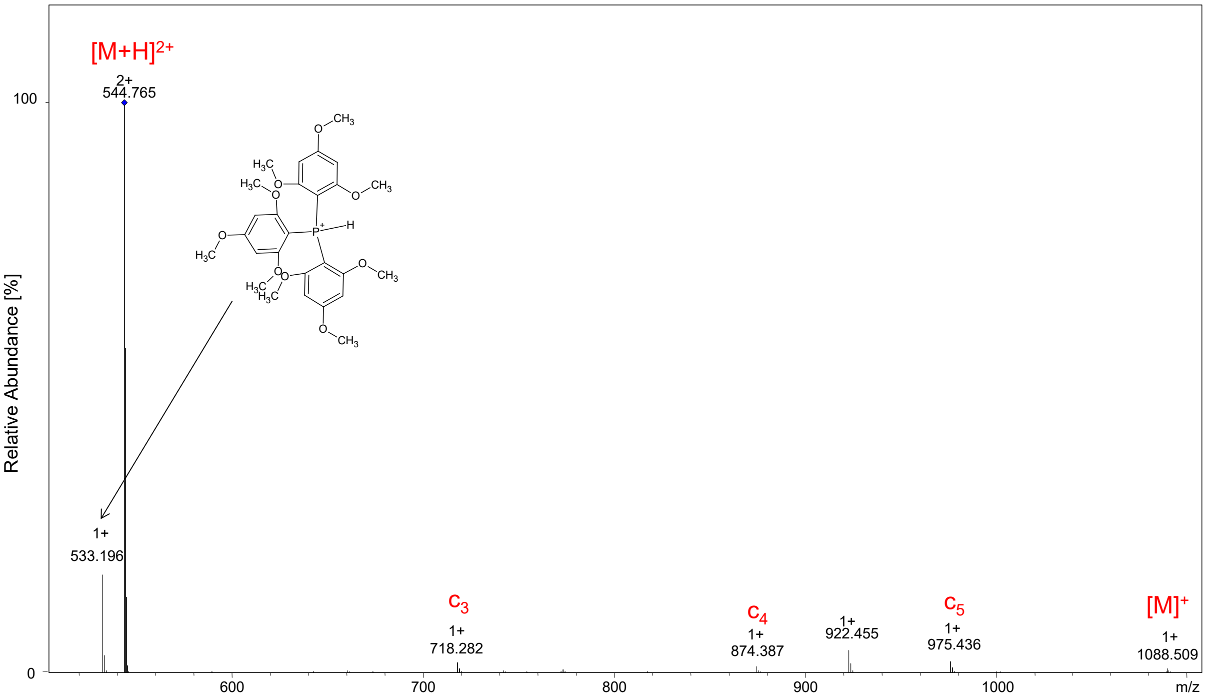

Figure S84. ESI-ECD-MS/MS spectrum of TMPP⁺-CH₂CO-Ala-Gly-Arg-Thr-Leu-NH₂ (4d). Precursor ion at *m/z* 544.765.

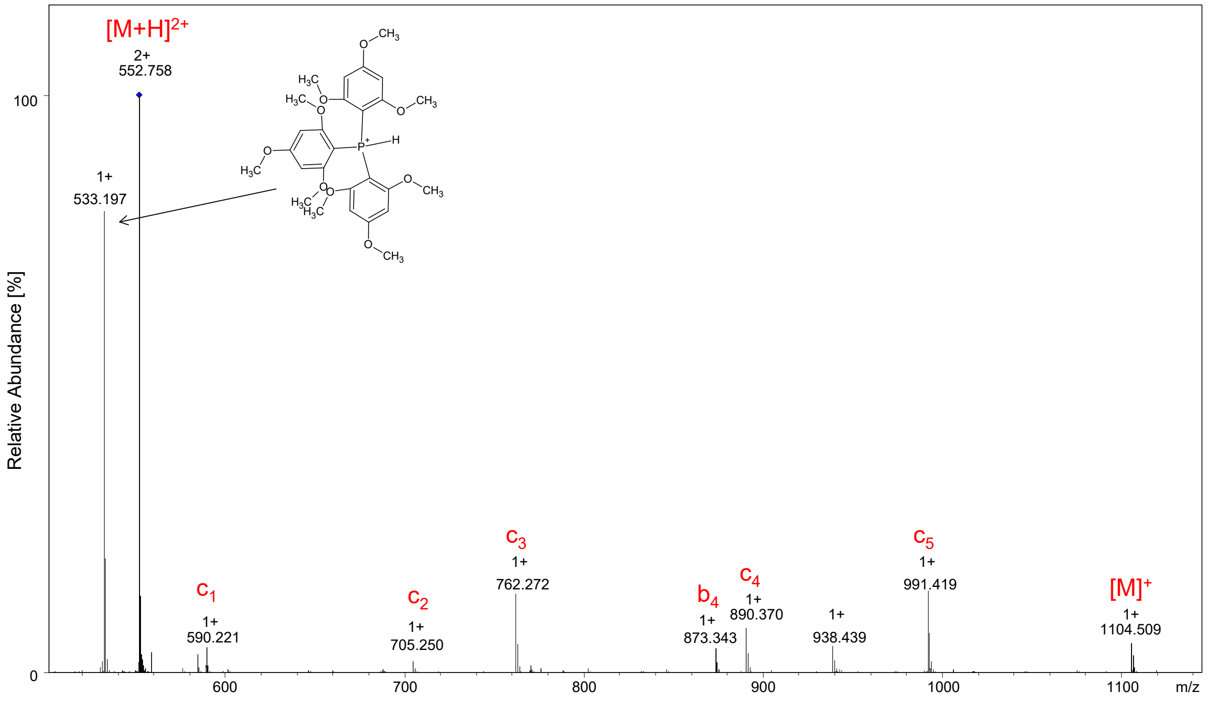

Figure S85. ESI-ECD-MS/MS spectrum of TMPP+-CH2CO-Asp-Gly-Lys-Thr-Leu-NH2 (5d). Precursor ion at *m/z* 552.758.

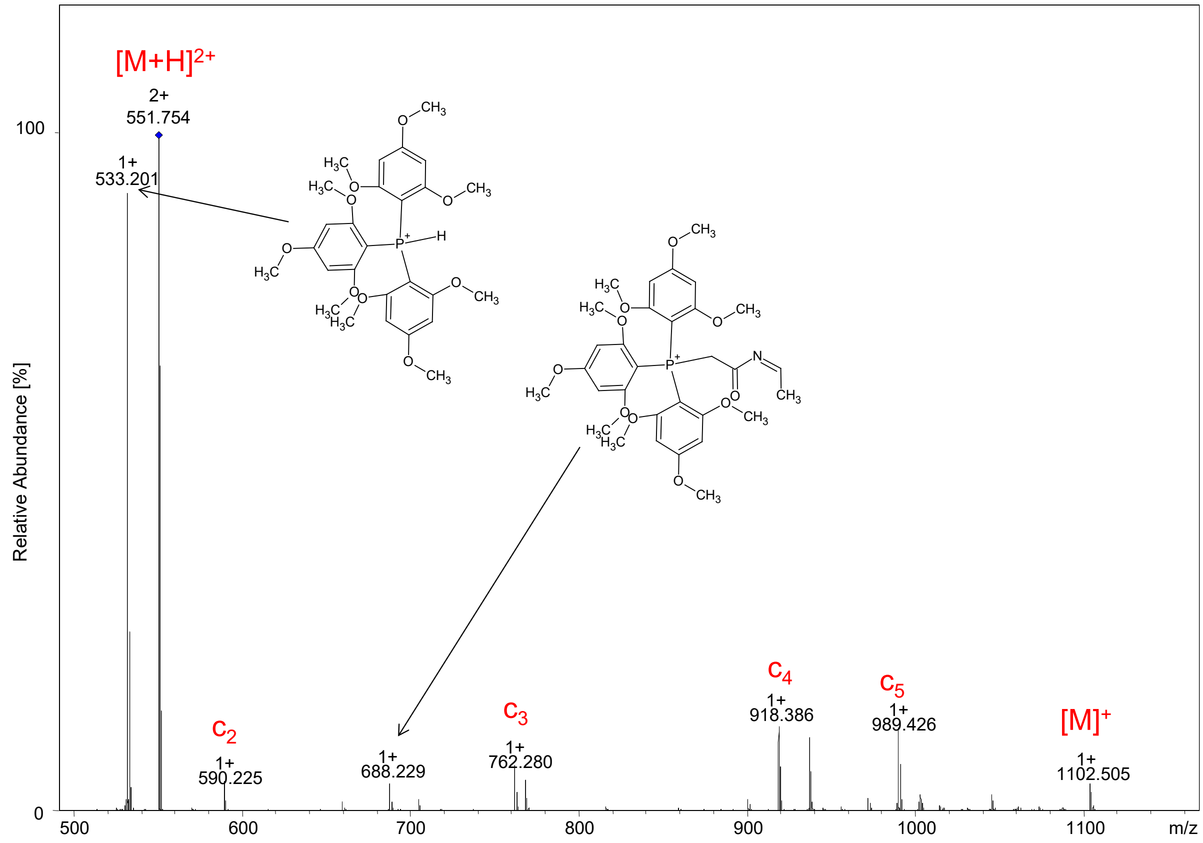

Figure S86. ESI-ECD-MS/MS spectrum of TMPP+-CH2CO-Asp-Gly-Arg-Ala-Leu-NH2 (7d). Precursor ion at *m/z* 551.754.
